# Posicionamento sobre Avaliação Multimodalidade em Imagem Cardíaca em Pacientes com Doenças Sistêmicas – 2026

**DOI:** 10.36660/abc.20260221

**Published:** 2026-04-28

**Authors:** Viviane Tiemi Hotta, Isabela Bispo Santos da Silva Costa, Renata Christian Martins Felix, Maria Estefânia Otto Bosco, Minna Moreira Dias Romano, Nathalia Conci Santorio, Simone Cristina Soares Brandão, Daniela do Carmo Rassi Frota, Alessandro Cavalcanti Lianza, Simone Nascimento dos Santos, Marcelo Dantas Tavares de Melo, Ana Clara Tude Rodrigues, Guilherme Loureiro Fialho, Karen Saori Shiraishi Sawamura, Marco Stephan Lofrano Alves, Angele Azevedo Alves Mattoso, Claudio Tinoco Mesquita, Frederico José Neves Mancuso, Marcelo Iorio Garcia, José Luiz Barros Pena, Adenalva Lima de Souza Beck, Andressa Mussi Soares, Sandra Marques e Silva, Jorge Andion Torreão, Paulo R. Schvartzman, Fábio Fernandes, Carlos Eduardo Rochitte, Silvio Henrique Barberato

**Affiliations:** 1 Hospital das Clínicas da Faculdade de Medicina da Universidade de São Paulo Instituto do Coração São Paulo SP Brasil Instituto do Coração do Hospital das Clínicas da Faculdade de Medicina da Universidade de São Paulo, São Paulo, SP – Brasil; 2 Fleury Medicina e Saúde, Grupo Fleury São Paulo SP Brasil Fleury Medicina e Saúde, Grupo Fleury, São Paulo, SP – Brasil; 3 Instituto do Câncer do Estado de São Paulo São Paulo SP Brasil Instituto do Câncer do Estado de São Paulo, São Paulo, SP – Brasil; 4 Instituto Nacional de Câncer Rio de Janeiro RJ Brasil Instituto Nacional de Câncer, Rio de Janeiro, RJ – Brasil; 5 Clínica de Medicina Nuclear Villela Pedras Rio de Janeiro RJ Brasil Clínica de Medicina Nuclear Villela Pedras, Rio de Janeiro, RJ – Brasil; 6 Universidade de Brasília Brasília DF Brasil Universidade de Brasília, Brasília, DF – Brasil; 7 Hospital DF Star Brasília DF Brasil Hospital DF Star, Brasília, DF – Brasil; 8 Universidade de São Paulo Hospital das Clínicas da Faculdade de Medicina de Ribeirão Preto Ribeirão Preto SP Brasil Universidade de São Paulo Hospital das Clínicas da Faculdade de Medicina de Ribeirão Preto, Ribeirão Preto, SP – Brasil; 9 Hospital das Clínicas da Universidade Federal de Pernambuco Recife PE Brasil Hospital das Clínicas da Universidade Federal de Pernambuco, Recife, PE – Brasil; 10 Faculdade de Medicina da Universidade Federal de Goiás Goiânia GO Brasil Faculdade de Medicina da Universidade Federal de Goiás, Goiânia, GO – Brasil; 11 Hospital São Francisco de Assis Goiânia GO Brasil Hospital São Francisco de Assis, Goiânia, GO – Brasil; 12 Hospital Israelita Albert Einstein São Paulo SP Brasil Hospital Israelita Albert Einstein, São Paulo, SP – Brasil; 13 Hospital das Clínicas da Faculdade de Medicina da Universidade de São Paulo Instituto da Criança e Adolescente São Paulo SP Brasil Instituto da Criança e Adolescente do Hospital das Clínicas da Faculdade de Medicina da Universidade de São Paulo, São Paulo, SP – Brasil; 14 Hcor - Associação Beneficente Síria São Paulo SP Brasil Hcor - Associação Beneficente Síria, São Paulo, SP – Brasil; 15 Eccos Diagnóstico CardioVascular Avançado Brasília DF Brasil Eccos Diagnóstico CardioVascular Avançado, Brasília, DF – Brasil; 16 Universidade Federal da Paraíba João Pessoa PB Brasil Universidade Federal da Paraíba, João Pessoa, PB – Brasil; 17 Hospital das Clínicas da Faculdade de Medicina da Universidade de São Paulo Instituto de Radiologia São Paulo SP Brasil Instituto de Radiologia do Hospital das Clínicas da Faculdade de Medicina da Universidade de São Paulo (INRAD HCFMUSP), São Paulo, SP – Brasil; 18 Universidade Federal de Santa Catarina Florianópolis SC Brasil Universidade Federal de Santa Catarina, Florianópolis, SC – Brasil; 19 Hospital de Clínicas da Universidade Federal do Paraná Curitiba PR Brasil Hospital de Clínicas da Universidade Federal do Paraná, Curitiba, PR – Brasil; 20 Hospital Santa Izabel Salvador BA Brasil Hospital Santa Izabel, Salvador, BA – Brasil; 21 Faculdade de Medicina da Universidade Federal Fluminense Niterói RJ Brasil Faculdade de Medicina da Universidade Federal Fluminense, Niterói, RJ – Brasil; 22 Serviço de Medicina Nuclear do Hospital Pro-Cardíaco Rio de Janeiro RJ Brasil Serviço de Medicina Nuclear do Hospital Pro-Cardíaco, Rio de Janeiro, RJ – Brasil; 23 Serviço de Medicina Nuclear do Hospital Vitória Rio de Janeiro RJ Brasil Serviço de Medicina Nuclear do Hospital Vitória, Rio de Janeiro, RJ – Brasil; 24 Hospital Universitário Antônio Pedro Health, Science & Education Lab Niterói RJ Brasil Health, Science & Education Lab - Hospital Universitário Antônio Pedro, Niterói, RJ – Brasil; 25 Universidade Federal de São Paulo Escola Paulista de Medicina São Paulo SP Brasil Universidade Federal de São Paulo - Escola Paulista de Medicina, São Paulo, SP – Brasil; 26 Hospital Universitário Clementino Fraga Filho da Universidade Federal do Rio de Janeiro Rio de Janeiro RJ Brasil Hospital Universitário Clementino Fraga Filho da Universidade Federal do Rio de Janeiro, Rio de Janeiro, RJ – Brasil; 27 Hospital Felicio Rocho Belo Horizonte MG Brasil Hospital Felicio Rocho, Belo Horizonte, MG – Brasil; 28 Instituto de Cardiologia e Transplantes do Distrito Federal Brasília DF Brasil Instituto de Cardiologia e Transplantes do Distrito Federal, Brasília, DF – Brasil; 29 Hospital Sirio Libanês Brasília DF Brasil Hospital Sirio Libanês, Brasília, DF – Brasil; 30 Hospital Evangélico de Cachoeiro de Itapemirim Cachoeiro de Itapemirim ES Brasil Hospital Evangélico de Cachoeiro de Itapemirim (HECI), Cachoeiro de Itapemirim, ES – Brasil; 31 Hospital Materno-Infantil - Unimed Sul Capixaba Cachoeiro de Itapemirim ES Brasil Hospital Materno-Infantil - Unimed Sul Capixaba, Cachoeiro de Itapemirim, ES – Brasil; 32 Hospital de Base do Distrito Federal Brasília DF Brasil Hospital de Base do Distrito Federal, Brasília, DF – Brasil; 33 Santa Casa da Bahia Salvador BA Brasil Santa Casa da Bahia, Salvador, BA – Brasil; 34 Hospital Moinhos de Vento Porto Alegre RS Brasil Hospital Moinhos de Vento, Porto Alegre, RS – Brasil; 35 Cardioeco - Centro de Diagnóstico Cardiovascular Curitiba Paraná Brasil Cardioeco - Centro de Diagnóstico Cardiovascular, Curitiba, Paraná – Brasil

**Table:** 

Posicionamento sobre Avaliação Multimodalidade em Imagem Cardíaca em Pacientes com Doenças Sistêmicas – 2026	
O relatório abaixo lista as declarações de interesse conforme relatadas à SBC pelos especialistas durante o período de desenvolvimento deste posicionamento, 2025/2026.	
Especialista	Tipo de relacionamento com a indústria
*Adenalva Lima de Souza Beck*	Nada a ser declarado
Alessandro Cavalcanti Lianza	Nada a ser declarado
Ana Clara Tude Rodrigues	Nada a ser declarado
Andressa Mussi Soares	Nada a ser declarado
Angele Azevedo Alves Mattoso	Nada a ser declarado
Carlos Eduardo Rochitte	Declaração financeira A - Pagamento de qualquer espécie e desde que economicamente apreciáveis, feitos a (i) você, (ii) ao seu cônjuge/ companheiro ou a qualquer outro membro que resida com você, (iii) a qualquer pessoa jurídica em que qualquer destes seja controlador, sócio, acionista ou participante, de forma direta ou indireta, recebimento por palestras, aulas, atuação como proctor de treinamentos, remunerações, honorários pagos por participações em conselhos consultivos, de investigadores, ou outros comitês, etc. Provenientes da indústria farmacêutica, de órteses, próteses, equipamentos e implantes, brasileiras ou estrangeiras: - GE Healthcare / Canon Medical Systems. B - Financiamento de pesquisas sob sua responsabilidade direta/pessoal (direcionado ao departamento ou instituição) provenientes da indústria farmacêutica, de órteses, próteses, equipamentos e implantes, brasileiras ou estrangeiras: - Novartis: Cleerly.
Claudio Tinoco Mesquita	Declaração financeira A - Pagamento de qualquer espécie e desde que economicamente apreciáveis, feitos a (i) você, (ii) ao seu cônjuge/ companheiro ou a qualquer outro membro que resida com você, (iii) a qualquer pessoa jurídica em que qualquer destes seja controlador, sócio, acionista ou participante, de forma direta ou indireta, recebimento por palestras, aulas, atuação como proctor de treinamentos, remunerações, honorários pagos por participações em conselhos consultivos, de investigadores, ou outros comitês, etc. Provenientes da indústria farmacêutica, de órteses, próteses, equipamentos e implantes, brasileiras ou estrangeiras: - Alnylam: Amiloidose B - Financiamento de pesquisas sob sua responsabilidade direta/pessoal (direcionado ao departamento ou instituição) provenientes da indústria farmacêutica, de órteses, próteses, equipamentos e implantes, brasileiras ou estrangeiras: - Astrazenica: Amilodose; Alnylam: Amilodose. Outros relacionamentos Financiamento de atividades de educação médica continuada, incluindo viagens, hospedagens e inscrições para congressos e cursos, provenientes da indústria farmacêutica, de órteses, próteses, equipamentos e implantes, brasileiras ou estrangeiras: - Alnylam: Amilodose; Pfizer: Amilodose.
Daniela do Carmo Rassi Frota	Declaração financeira A - Pagamento de qualquer espécie e desde que economicamente apreciáveis, feitos a (i) você, (ii) ao seu cônjuge/ companheiro ou a qualquer outro membro que resida com você, (iii) a qualquer pessoa jurídica em que qualquer destes seja controlador, sócio, acionista ou participante, de forma direta ou indireta, recebimento por palestras, aulas, atuação como proctor de treinamentos, remunerações, honorários pagos por participações em conselhos consultivos, de investigadores, ou outros comitês, etc. Provenientes da indústria farmacêutica, de órteses, próteses, equipamentos e implantes, brasileiras ou estrangeiras: - AstraZeneca do Brasil Ltda: Forxiga.
Fábio Fernandes	Nada a ser declarado
Frederico José Neves Mancuso	Nada a ser declarado
Guilherme Loureiro Fialho	Outros relacionamentos Financiamento de atividades de educação médica continuada, incluindo viagens, hospedagens e inscrições para congressos e cursos, provenientes da indústria farmacêutica, de órteses, próteses, equipamentos e implantes, brasileiras ou estrangeiras: - NovoNordis: Congresso da SBC em SP 2025
Isabela Bispo Santos da Silva Costa	Nada a ser declarado
Jorge Andion Torreão	Nada a ser declarado
José Luiz Barros Pena	Nada a ser declarado
Karen Saori Shiraishi Sawamura	Outros relacionamentos Participação societária de qualquer natureza e qualquer valor economicamente apreciável de empresas na área de saúde, de ensino ou em empresas concorrentes ou fornecedoras da SBC: - Ensino online: WavesMed.
Marcelo Dantas Tavares de Melo	Nada a ser declarado
Marcelo Iorio Garcia	Nada a ser declarado
Marco Stephan Lofrano Alves	Nada a ser declarado
Maria Estefânia Bosco Otto	Nada a ser declarado
Minna Moreira Dias Romano	Nada a ser declarado
Nathalia Conci Santorio	Nada a ser declarado
Paulo R Schvartzman	Nada a ser declarado
Renata Christian Martins Felix	Nada a ser declarado
Sandra Marques e Silva	Declaração financeira A - Pagamento de qualquer espécie e desde que economicamente apreciáveis, feitos a (i) você, (ii) ao seu cônjuge/ companheiro ou a qualquer outro membro que resida com você, (iii) a qualquer pessoa jurídica em que qualquer destes seja controlador, sócio, acionista ou participante, de forma direta ou indireta, recebimento por palestras, aulas, atuação como proctor de treinamentos, remunerações, honorários pagos por participações em conselhos consultivos, de investigadores, ou outros comitês, etc. Provenientes da indústria farmacêutica, de órteses, próteses, equipamentos e implantes, brasileiras ou estrangeiras: - Pfizer: Amiloidose; Alnylam: Amiloidose; PTC: Amiloidose; AstraZeneca: Amiloidose; Sanofi: Doença de Fabry; Takeda: Doença de Fabry. Outros relacionamentos Financiamento de atividades de educação médica continuada, incluindo viagens, hospedagens e inscrições para congressos e cursos, provenientes da indústria farmacêutica, de órteses, próteses, equipamentos e implantes, brasileiras ou estrangeiras: - Pfizer: Amiloidose; Alnylam: Amiloidose; PTC: Amiloidose; AstraZeneca: Amiloidose; Sanofi: Doença de Fabry; Takeda: Doença de Fabry.
Silvio Henrique Barberato	Declaração financeira A - Pagamento de qualquer espécie e desde que economicamente apreciáveis, feitos a (i) você, (ii) ao seu cônjuge/ companheiro ou a qualquer outro membro que resida com você, (iii) a qualquer pessoa jurídica em que qualquer destes seja controlador, sócio, acionista ou participante, de forma direta ou indireta, recebimento por palestras, aulas, atuação como proctor de treinamentos, remunerações, honorários pagos por participações em conselhos consultivos, de investigadores, ou outros comitês, etc. Provenientes da indústria farmacêutica, de órteses, próteses, equipamentos e implantes, brasileiras ou estrangeiras: - Pfizer: Amiloidose; AstraZeneca: ICFEP. Outros relacionamentos Financiamento de atividades de educação médica continuada, incluindo viagens, hospedagens e inscrições para congressos e cursos, provenientes da indústria farmacêutica, de órteses, próteses, equipamentos e implantes, brasileiras ou estrangeiras:
Simone Cristina Soares Brandão	Nada a ser declarado
Simone Nascimento dos Santos	Nada a ser declarado
Viviane Tiemi Hotta	Nada a ser declarado

**Figure:**
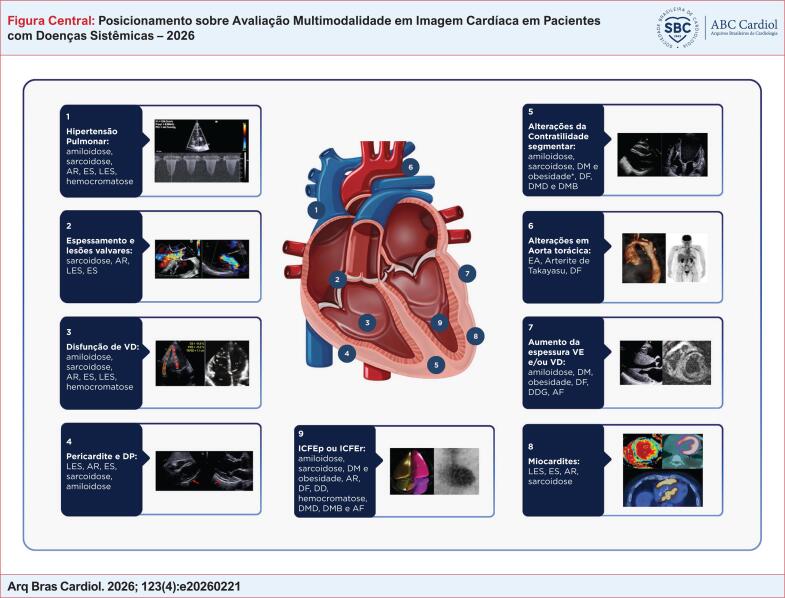
AR: artrite reumatóide; DD: Doença de Danon; DDG: doenças do depósito de glicogênio; DF: Doença de Fabry; DM: diabetes mellitus; DMD: distrofia muscular de Duchenne; DMB: distrofia muscular de Becker; DP: derrame pericárdico; EA: espondilite anquilosante; ES: esclerose sistêmica; ICFEp: insuficiência cardíaca com fração de ejeção preservada; ICFEr: insuficiência cardíaca com fração de ejeção reduzida; LES: Lupus eritematoso sistêmico; VD: ventrículo direito; VE: ventrículo esquerdo. *Na presença de doença arterial coronária ou alterações na microcirculação. Edição da figura com contribuição da Dra Thais Baptista Teixeira.

## Sumário

**1. Introdução** 8
**2. Aplicações da Multimodalidade em Imagem Cardíaca nos Distúrbios da Matriz Extracelular Miocárdica** 9
  **2.1. Amiloidose Cardíaca** 9
    **2.1.1. Ecocardiografia** 9
    **2.1.2. Ressonância Magnética Cardíaca** 12
    **2.1.3. Cintilografia Miocárdica** 14
  **2.2. Sarcoidose Cardíaca** 16
    **2.2.1. Ecocardiografia** 17
    **2.2.2. Ressonância Magnética Cardíaca** 19
    **2.2.3. Papel do PET/CT com** ^18^**F-FDG** 23
**3. Aplicações da Multimodalidade em Imagem Cardíaca nas Doenças Metabólicas** 24
  **3.1. Diabete Mellitus** 24
  **3.2. Obesidade** 26
**4. Aplicações da Multimodalidade em Imagem Cardíaca nas Doenças Reumatológicas** 28
  **4.1. Lúpus Eritematoso Sistêmico** 28
  **4.2. Esclerose Sistêmica** 31
  **4.3. Espondilite Anquilosante** 34
  **4.4. Artrite Reumatoide** 36
  **4.6. Arterite de Takayasu** 40
**5. Aplicações da Multimodalidade em Imagem Cardíaca nas Doenças de Depósito** 43
  **5.1. Cardiomiopatia por Sobrecarga de Ferro** 43
  **5.2. Doença de Fabry** 47
  **5.3. Doenças de Depósito do Glicogênio** 51
**6. Aplicações da Multimodalidade em Imagem Cardíaca as Doenças Neuromusculares** 55
  **6.1. Distrofia Muscular de Duchenne** 55
  **6.2. Distrofia Muscular de Becker** 55
  **6.3. Ataxia de Friedreich** 56
**7. Perspectivas** 59
**8. Conclusões** 60
**Referências** 60

## 1. Introdução

Doenças inflamatórias sistêmicas, imunomediadas ou não, estão frequentemente associadas a manifestações cardíacas adversas devido a alterações no ritmo, morfologia ou função cardíaca.^1,2^

Apesar da diversidade de doenças sistêmicas com acometimento cardíaco e do amplo espectro de manifestações clínicas, não há um documento que detalhe as aplicações e os achados dos métodos de imagem cardíaca por multimodalidade (MM). Este documento aborda os principais achados nas doenças sistêmicas mais relevantes, selecionadas por sua prevalência e pela importância do reconhecimento de condições que, embora raras, são frequentemente subdiagnosticadas, como a amiloidose cardíaca (AC), sarcoidose cardíaca (SC), a Doença de Fabry e as distrofias neuromusculares, entre outras.^1,2^ A proposta deste posicionamento é abordar as principais doenças sistêmicas que cursam com alterações cardiovasculares, porém, com foco nos aspectos diagnósticos aos métodos de imagem. Não é o escopo deste documento o detalhamento de cada doença em si.

O ecocardiograma transtorácico (ETT) é o exame de imagem de eleição para o diagnóstico de anormalidades estruturais, funcionais e hemodinâmicas cardíacas. Recentemente, avanços tecnológicos permitiram o aprimoramento da ecocardiografia, incluindo técnicas para a análise da mecânica cardíaca, além da avaliação convencional.^3,4^ A análise da deformação miocárdica (*strain*), especialmente por meio do *strain* longitudinal global (SLG), permite identificar disfunção subclínica mesmo com fração de ejeção preservada. Essa técnica tem ampla aplicação no diagnóstico precoce de doenças cardiovasculares.^5,6^ Estudos recentes observaram que o uso do SLG tem valor diagnóstico e prognóstico em diversas doenças cardíacas, com impacto direto no manejo clínico dos pacientes.^5–8^

A ressonância magnética cardíaca (RMC), por sua vez, é considerada o método de imagem padrão-ouro para a avaliação estrutural cardíaca. Ademais, a RMC permite a caracterização tecidual miocárdica com identificação de sinais de inflamação e edema pelas imagens de mapa T1 e T2, bem como sinais de fibrose pela técnica de realce tardio (RT), cujos padrões podem sugerir o fator etiológico das alterações detectadas. A RMC permite ainda a análise da matriz e volume extracelular (VEC), parâmetros importantes para o diagnóstico e prognóstico das alterações cardíacas nas doenças sistêmicas.^9,10^

**Figure:**
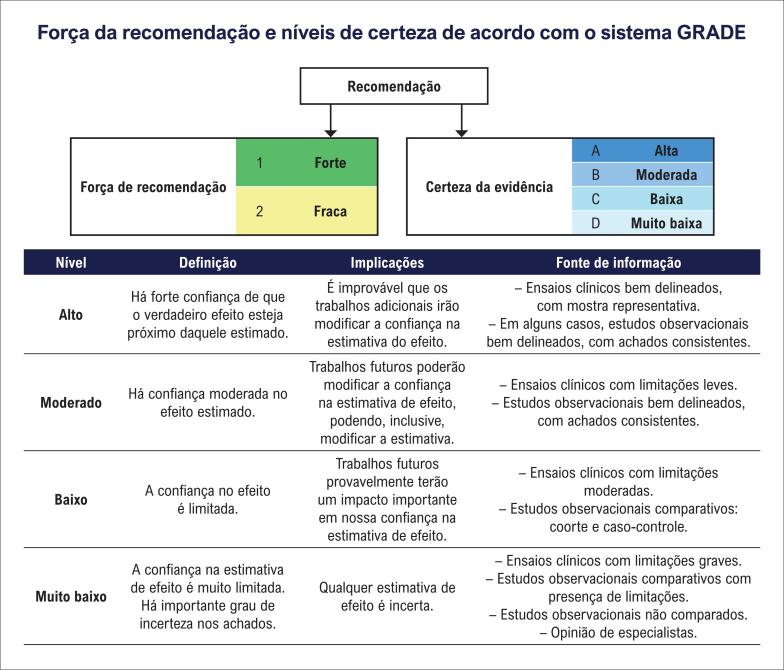
GRADE: Grading of Recommendations, Assessment, Development and Evaluation. Fonte: Elaboração GRADE Working Group: www.gradeworkinggroup.org.

As técnicas de cardiologia nuclear, por sua vez, permitem análises da fisiologia cardiovascular, incluindo metabolismo, perfusão miocárdica, inervação e função ventricular. Essas abordagens permitem a detecção precoce de anormalidades cardíacas, possibilitando intervenções antes da progressão para formas graves e irreversíveis da doença A cintilografia de perfusão miocárdica (CPM) com a injeção de marcadores como o 99mTc-sestamibi, associada ao estresse físico ou farmacológico, é um método consagrado para a avaliação de paciente com suspeita de doença coronária obstrutiva, bem como para a estratificação de risco.^11^

Assim, os métodos de imagem cardíaca – como a ecocardiografia, a RMC, a tomografia computadorizada (TC) e as técnicas de análise de metabolismo e perfusão – desempenham um papel fundamental na avaliação de pacientes com doenças sistêmicas. Dessa maneira, é importante o conhecimento das aplicações dos métodos de imagem cardiovascular por MM nas doenças sistêmicas, promovendo seu uso racional e otimizado na prática clínica, tanto por cardiologistas quanto por especialistas de áreas.^3,5,9–12^ No entanto, ainda carecem de padronização e de documentos que orientem suas aplicações e os principais achados relacionados às alterações cardíacas nesses pacientes. Portanto, este documento tem como principal objetivo fornecer um guia prático sobre as aplicações atuais e potenciais dos métodos de imagem por MM nas doenças sistêmicas – incluindo orientações sobre quando solicitar cada exame e como interpretar seus achados no contexto clínico do paciente, com foco na identificação de sinais sugestivos dessas condições (Figura Central).

Este documento foi elaborado segundo as novas normas da Sociedade Brasileira de Cardiologia (SBC), que sugerem documentos objetivos, concisos e de menor extensão, além da adoção do sistema GRADE (*Grading of Recommendations, Assessment, Development and Evaluation*) para estabelecer o nível de evidência e força da recomendação. Esse sistema consiste em um modelo de graduação mais simples (recomendação forte, fraca ou neutra; certeza de evidência alta, moderada, baixa ou muito baixa) para estabelecer o nível de evidência e força da recomendação conforme o diagrama abaixo.

É importante ressaltar que são raros os estudos diagnósticos que apresentam metodologia adequada e que avaliam desfechos clínicos, mesmo nas doenças cardiovasculares mais prevalentes, como doença coronária, insuficiência cardíaca (IC) e valvopatias. Assim, tendo em vista a menor prevalência das doenças contempladas neste documento e, muitas vezes, por se tratarem de doenças raras, os ensaios clínicos randomizados, metanálises e revisões sistemáticas avaliando os métodos de imagem cardíaca são escassos ou até ausentes. De modo que, em alguns casos, mesmo na ausência de grandes ensaios clínicos, pode haver consenso de que determinado exame de imagem é claramente benéfico.

## 2. Aplicações da Multimodalidade em Imagem Cardíaca nos Distúrbios da Matriz Extracelular Miocárdica

### 2.1. Amiloidose Cardíaca

A amiloidose é uma doença sistêmica que pode causar acometimento cardíaco com fisiologia restritiva pelo acúmulo extracelular de fibrilas de proteína amiloide no miocárdio, levando à rigidez ventricular, disfunção diastólica (DD) e, eventualmente, IC. Além dessas alterações morfofuncionais, esse depósito progressivo miocárdico associa-se com importantes alterações elétricas como fibrilação atrial e bloqueios atrioventriculares variados. É importante realçar que, dentro da AC, existem, principalmente, dois subtipos de etiologia distintas, com muita similaridade nas suas manifestações clínicas.^13,14^

- Amiloidose por cadeias leves (AL): decorrente da deposição de cadeias leves de imunoglobulinas produzidas por distúrbio plasmocitário na medula óssea.
- Amiloidose por transtirretina (ATTR): resultante do acúmulo de transtirretina dobrada incorretamente, podendo ser do tipo selvagem (ATTRwt), sem mutações detectadas, ou hereditária (ATTRv), devido a mutações genéticas.

A diferenciação entre as formas AL e ATTR é desafiadora na prática clínica. Porém, a toxicidade e a deposição miocárdica da fibrila amiloide AL são superiores às da ATTR resultando em aumento mais rápido da espessura miocárdica, maior ocorrência de derrame pericárdico (DP) e, frequentemente, mais acometimento dos parâmetros de função sistólica. A Tabela 1 resume as principais diferenças entre as duas formas de AC.^15–17^

**Tabela 1:** Amiloidose por cadeia leve vs. amiloidose por transtirretina

Aspectos	Cadeia leve	Transtirretina
Proteína precursora	Cadeias leves de imunoglobulina	Proteína transtirretina
Patogênese	Produção excessiva de cadeias leves por plasmócitos	Deposição de transtirretina anormal (mutada ou selvagem)
Formas clínicas	Primária (associada a discrasias plasmocitárias) ou associada ao mieloma múltiplo	Hereditária (mutação genética) ou selvagem
Órgãos frequentemente acometidos	Coração, rins, sistema nervoso periférico, trato gastrointestinal	Predominantemente cardíaco e sistema nervoso periférico
Idade de acometimento	Meia-idade a idosos (geralmente > 50 anos)	Idosos (> 60 anos), especialmente na forma selvagem
Diagnóstico	Presença de cadeia leve monoclonal no soro e/ou urina **e** infiltrado amiloide no tecido corado com vermelho Congo	Teste genético, cintilografia com difosfonatos (99mTc-PYP, DPD ou HMDP) **e** infiltrado amiloide no tecido corado com vermelho Congo
Tratamento	Quimioterapia, imunoterapia, transplante autólogo de células-tronco	Estabilizadores de transtirretina (tafamidis, diflunisal), agentes de RNA de interferência (patisiran e vutrisiran) terapia genética emergente

^99m^Tc: 99m-tecnécio; DPD: difosfonato de dicarboxipropano; HMDP: hidroximetileno difosfonato; PYP: pirofosfato.

**Figura 1:**
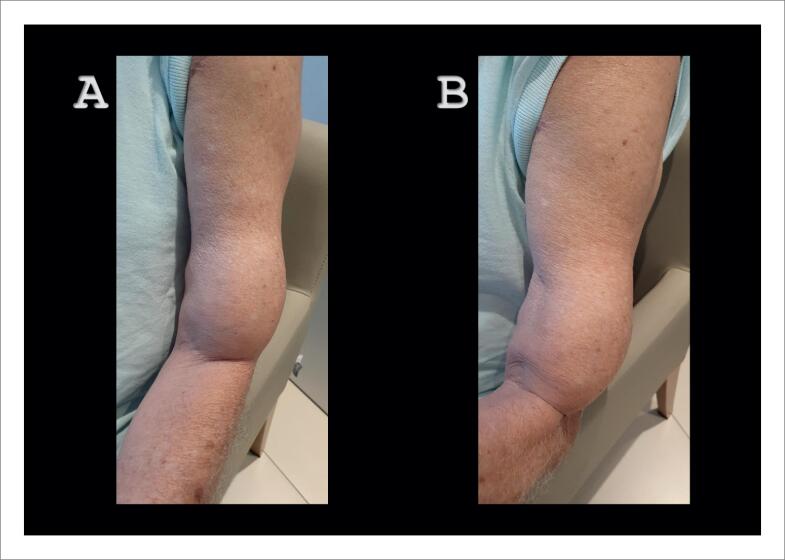
A ruptura espontânea do tendão do músculo do bíceps é um sinal de alerta na suspeita clínica de amiloidose, principalmente na forma transtirretina. A – Membro superior esquerdo relaxado com uma porção do bíceps próximo à fossa cubital. B – Contração do músculo bíceps esquerdo sem grandes alterações em relação à musculatura relaxada.

O diagnóstico precoce da AC depende de uma alta suspeição clínica devido à sua apresentação inicial inespecífica, semelhante a outras condições cardíacas.^18^ Porém, a presença de aumento da espessura ventricular em pacientes sem hipertensão arterial ou valvopatia aórtica, associada a sinais extracardíacos, como síndrome do túnel do carpo, estenose do canal medular, neuropatia periférica e disautonomia, pode servir como pistas diagnósticas importantes (Figura 1 e Tabela 2).^19^

**Tabela 2:** Sinais de alerta na suspeita de amiloidose cardíaca

Categoria	Sinais de alerta
História clínica	Síndrome do túnel do carpo bilateral História familiar de insuficiência cardíaca ou neuropatia Estreitamento do canal medular sem causa evidente Disautonomia (hipotensão ortostática, principalmente) Alterações inexplicadas do hábito intestinal Ruptura espontânea de tendão (mais comum tendão do bíceps)
Achados cardiológicos	Baixa voltagem no eletrocardiograma, desproporcional à espessura miocárdica Padrão de pseudoinfarto no eletrocardiograma Estenose aórtica com fração de ejeção preservada com baixo fluxo e baixo gradiente Marcapasso (principalmente com história familiar e aumento de espessura ventricular) Insuficiência cardíaca com fração de ejeção preservada
Achados sistêmicos	Proteinúria inexplicada Neuropatia periférica/autonômica Macroglossia (na forma AL) Equimoses, principalmente púrpura periorbitária na forma AL Elevação persistente de troponina e peptídeos natriuréticos cerebrais Síndrome edemigênica Perda de peso inexplicada Presença de paraproteína monoclonal
Exames complementares	Ecocardiograma com aumento da espessura sem causa evidente de aumento da pós-carga *Strain* longitudinal global do ventrículo esquerdo reduzido, com padrão de *apical sparing* Cintilografia óssea com captação cardíaca intensa (graus 2 e 3 de Perugini) Realce tardio difuso, elevação T1 nativo e volume extracelular na ressonância magnética cardíaca

AL: amiloidose por cadeias leves.

#### 2.1.1. Ecocardiografia

**Figura 2:**
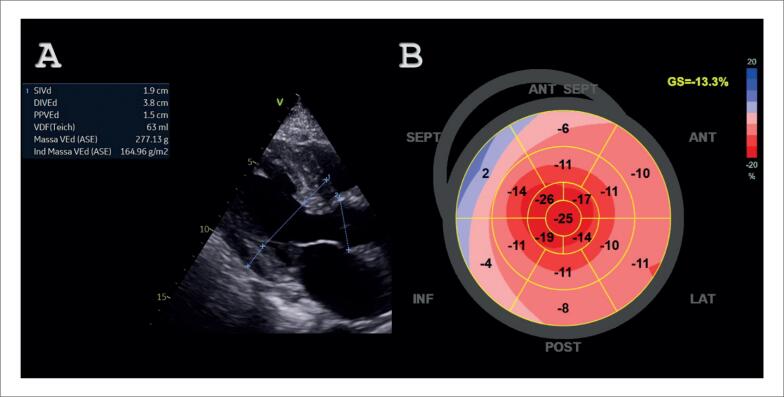
Achados ecocardiográficos sugestivos de cardiomiopatia amiloide. A – Janela paraesternal de eixo longo com aumento importante da espessura ventricular, diâmetro diastólico ventricular esquerdo reduzido e aumento do átrio esquerdo. B – Imagem paramétrica do strain longitudinal global do ventrículo esquerdo com valor global reduzido (13,3%) e preservação na região apical.

O ETT é o exame de imagem de primeira linha diagnóstica na AC.^3^ Tendo como base a fisiopatologia da doença, em que ocorre o depósito amiloide intersticial miocárdico progressivo, pode-se observar aumento da espessura biventricular, aumento da rigidez e redução da complacência ventricular, com DD, redução do volume sistólico ejetado e redução do SLG do ventrículo esquerdo (VE) que preserva as regiões apicais (*apical sparing*) (Tabela 3, Figuras 2 e 3).^14,20–23^

**Tabela 3:** Principais achados ecocardiográficos na cardiomiopatia amiloide

Parâmetro ecocardiográfico	Achados típicos na cardiomiopatia amiloide
Espessura da parede ventricular	Aumento da espessura miocárdica com padrão concêntrico, frequentemente simétrica e significativa (> 12 mm). Pode ser observado aumento predominante em parede septal com padrão assimétrico
Função ventricular esquerda	Fração de ejeção do ventrículo esquerdo inicialmente preservada, evoluindo para disfunção sistólica em estágios avançados
Padrão de enchimento ventricular	Disfunção diastólica restritiva nas fases avançadas
*Strain* longitudinal global	Redução do *strain* longitudinal global com padrão típico de *apical sparing*
Aspecto das valvas cardíacas	Espessamento valvar por infiltração das valvas atrioventriculares
Derrame pericárdico	Frequente, geralmente discreto a moderado
Dilatação atrial	Dilatação e disfunção atrial, podendo acometer ambos os átrios
Hiper Refringência septal (*sparkling*)	Presente em alguns casos, mas com baixa sensibilidade e especificidade para o diagnóstico de forma isolada
Função ventricular direita	Disfunção progressiva associada a mau prognóstico, presente em estágios avançados
Hipertensão pulmonar	Presente em estágios avançados devido à elevação das pressões de enchimento cardíacas esquerdas
Septo atrial	Espessamento do septo atrial

**Figura 3:**
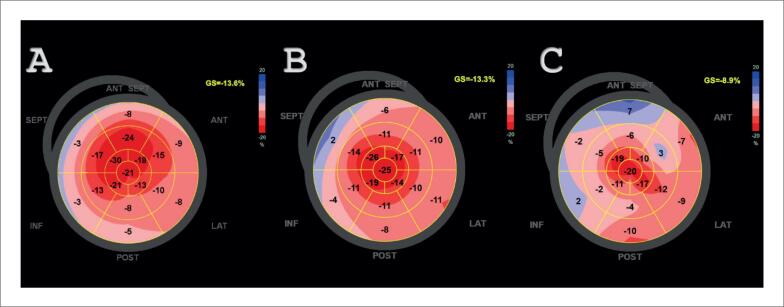
Análise paramétrica do strain longitudinal global do ventrículo esquerdo de paciente com amiloidose cardíaca AL em três momentos distintos. A – Suspeita diagnóstica inicial evidenciando redução do valor global (13,6%), preservando a região apical. B – Diagnóstico estabelecido e início do tratamento 3 meses após. C – Seguimento do tratamento com quimioterapia, mostrando importante queda do valor global do strain global longitudinal (8,9%) após 5 meses, falecendo nesse momento.

Modelos ecocardiográficos multiparamétricos foram desenvolvidos para melhorar a precisão diagnóstica.^24^ Esses modelos incorporam diversos parâmetros ecocardiográficos, como espessura relativa da parede, relação E/e’, SLG e excursão sistólica do plano do anel tricúspide (TAPSE, do inglês *tricuspid annular plane systolic excursion*), para criar escores diagnósticos mais acurados para a AC.^25^

A avaliação ecocardiográfica da resposta terapêutica na AC envolve principalmente a análise das alterações funcionais e morfológicas do miocárdio. Entre os principais indicadores de resposta ao tratamento, estão a redução da espessura da parede do VE e a estabilização ou melhora do SLG. Além disso, a razão E/e’ e a rigidez atrial esquerda são parâmetros ecocardiográficos capazes de refletir mudanças na função diastólica após o tratamento, com melhorias que se correlacionam com a resposta favorável ao tratamento.^26–50^

#### 2.1.2. Ressonância Magnética Cardíaca

A RMC permite avaliar com precisão as alterações do tecido miocárdico associadas a AC.^27^ O acúmulo de miofibrilas resulta no espessamento das paredes do VE e do septo interatrial, alterações que podem ser detectadas pelas sequências de caracterização morfológica da RMC, frequentemente sem dilatação significativa da cavidade ventricular.^28,29^ Há, ainda, aumento do volume atrial, DD e, em casos avançados, redução da fração de ejeção.^29^ É frequente a ocorrência de derrames pleural e pericárdico.^29^

Semelhantes aos achados ecocardiográficos, pacientes com AC apresentam redução mais pronunciada e difusa do SLG, com preservação relativa das regiões apicais, além de comprometimento do *strain* circunferencial, especialmente nas regiões basais.^49^ A análise biventricular do *strain* por RMC também permite detectar envolvimento precoce do ventrículo direito (VD), contribuindo para uma avaliação mais abrangente da gravidade e extensão da infiltração amiloide.^50^ Estudos com RMC demonstraram correlação significativa entre a disfunção atrial esquerda e a carga de amiloide, indicando que a avaliação funcional da câmara atrial também pode ser um marcador precoce de envolvimento cardíaco.^51^

Outra modificação tecidual observada é o aumento do teor de água total no miocárdio, o qual pode resultar da expansão do VEC em decorrência do acúmulo de proteínas e seu efeito osmótico, bem como do incremento de água intracelular em miócitos submetidos à agressão citotóxica provocada pelo depósito proteico ou, ainda, por redução da perfusão miocárdica, relacionada ao maior afastamento dos capilares ou à sua obstrução.

A elevação do conteúdo hídrico global no tecido miocárdico provoca o prolongamento dos tempos de relaxamento do hidrogênio, tanto no eixo longitudinal (T1) quanto no transversal (T2).^43^ Entretanto, a alteração tecidual mais marcante e clinicamente significativa na AC é a acentuada expansão do VEC miocárdico, atribuída não apenas ao acúmulo de fibrilas amiloides nas fases sintomáticas da doença, mas também à fibrose de reparo no miocárdio. Dessa forma, a combinação entre depósito proteico e fibrose intersticial pode ser detectada com elevada acurácia e até quantificada por meio das técnicas de RT^42,44^ e cálculo do VEC do miocárdio.^32–35^ O VEC do miocárdio normal está em torno de 25%, enquanto na AC, especialmente na forma ATTR, pode atingir valores tão altos quanto 60%.^47,48^

O RT é uma das técnicas mais clássicas da RMC na avaliação da AC. Caracteristicamente, observa-se um padrão difuso subendocárdico ou transmural de realce, que contrasta com padrões mais localizados vistos em outras cardiomiopatias.^52,53^ A distribuição global do RT está associada a maior carga de depósito amiloide e a pior prognóstico.^54^ De forma característica, a inversão da curva de recuperação do miocárdio pode ser difícil de se obter, sugerindo comprometimento difuso do interstício.

**Figura 4:**
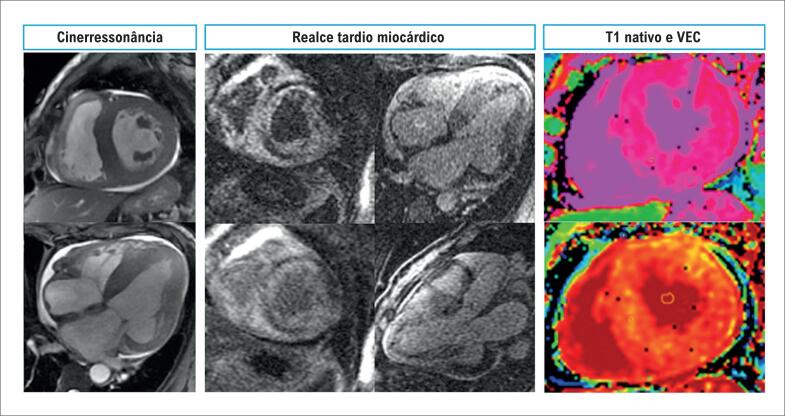
Caso ilustrativo de paciente com cardiomiopatia amiloide vista pela ressonância magnética cardíaca. A cinerressonância mostra aumento biatrial e aumento da espessura miocárdica biventricular; as imagens de realce tardio miocárdico mostram acometimento difuso; e os valores de T1 nativo e volume extracelular encontram-se elevados. VEC: volume extracelular.

Estudos demonstram que o T1 nativo e o VEC estão significativamente elevados nos pacientes com amiloidose, o que pode ocorrer mesmo na ausência de RT visível, permitindo o diagnóstico precoce do acometimento cardíaco.^52,55^ O VEC é altamente sensível à deposição de amiloide. Metanálises recentes publicadas reforçam o papel do RT, do T1 nativo e do VEC na avaliação prognóstica de pacientes com AC, sendo preditores de mortalidade.^55,56^ A Figura 4 ilustra um caso de AC avaliada pela RMC.

Alguns estudos iniciais avaliaram o papel da RMC com avaliação de *strain* miocárdico em pacientes com AC, permitindo caracterizar de forma mais sensível e precoce as alterações funcionais do miocárdio.^49,50^ Estudos recentes demonstram que, além do tradicional SLG, parâmetros adicionais como *strain* circunferencial e radial do VE, assim como os componentes de *strain* do VD, oferecem discriminação adicional entre a AL e outras condições, como a cardiomiopatia hipertrófica (CMH).^49^

A RMC também tem papel potencial no seguimento terapêutico, observado pela redução da massa ventricular.^57^ A quantificação seriada de VEC e T1 nativo pode indicar resposta à terapia específica, como estabilizadores da transtirretina ou quimioterapia para AL.^58,59^ A redução do VEC ao longo do tempo tem sido associada a melhora clínica e maior sobrevida, destacando seu valor como marcador de resposta ao tratamento.^55,59^

A RMC, junto com achados da história clínica e dados laboratoriais, permite distinguir a AC de outras causas de aumento da espessura miocárdica, como CMH ou hipertensão arterial sistêmica (HAS). A CMH geralmente apresenta espessamento assimétrico e realce localizado, enquanto a hipertrofia por sobrecarga pressórica geralmente não está associada a alterações no T1 ou VEC. O padrão difuso de RT, o aumento marcante do VEC e os valores elevados de T1 nativo são característicos da AC e ajudam a distinguir essa condição de outras hipertrofias miocárdicas.^29^

#### 2.1.3. Cintilografia Miocárdica

**Figura 5:**
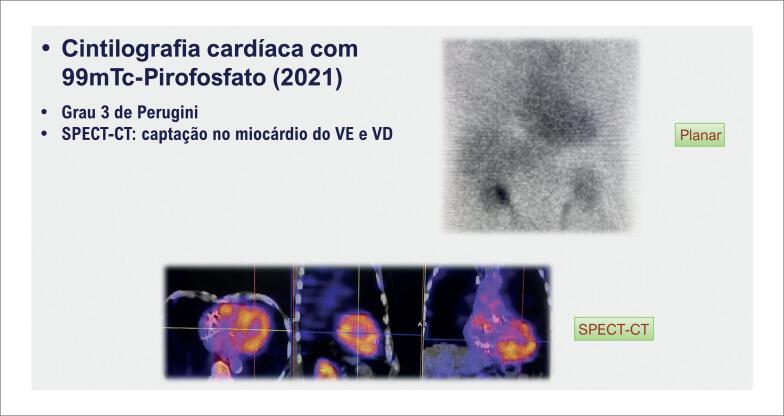
Cintilografia com pirofosfato de 99m-tecnécio demonstrando captação mais intensa do que a das costelas (imagem planar com Grau 3 de Perugini) e confirmada a captação no miocárdio em imagem tomográfica (SPECT-CT). O paciente teve a exclusão de gamopatia monoclonal realizada, sendo confirmado o diagnóstico de amiloidose por transtirretina. SPECT-CT: tomografia computadorizada por emissão de fóton único-tomografia computadorizada (Single photon emission computed tomography-computed tomography); VD: ventrículo direito; VE: ventrículo esquerdo.

As diretrizes atuais recomendam a investigação inicial da AC por meio de MM em imagem cardíaca. Na suspeita de AC, é essencial a exclusão de gamopatia monoclonal por meio da dosagem de cadeias leves livres de imunoglobulinas no sangue e na urina. Uma vez descartada a gamopatia, a cintilografia com pirofosfato de 99m-tecnécio (99mTc-PYP) torna-se um método de elevada acurácia. A captação miocárdica do traçador com intensidade igual ou superior à observada nas costelas (Graus 2 ou 3 na escala de Perugini) permite o diagnóstico não invasivo de AC por transtirretina (ATTR), sem a necessidade de biópsia endomiocárdica (Figura 5).^60^

Porém, a realização da cintilografia com 99mTc-PYP requer a observância de critérios técnicos específicos para assegurar sua acurácia no diagnóstico de AC ATTR : (a) necessidade de confirmação da captação do traçador no miocárdio, através da realização de imagens cintilográficas tomográficas (SPECT, do inglês, *single photon emission computed tomography*) para evitar falsos positivos decorrentes de acúmulo do radiotraçador no "*pool*" sanguíneo; e (b) exclusão criteriosa da presença da gamopatia monoclonal com realização de imunoeletroforese sérica e urinária e pesquisa de cadeias leves no sangue. Apenas após a exclusão da presença de cadeias leves, a cintilografia com 99mTc-PYP apresenta especificidade diagnóstica adequada para o diagnóstico não invasivo de AC ATTR, sem necessidade de biópsia, considerando-se que até 20% dos casos de AC AL podem ter captação na cintilografia.

Pacientes com captação miocárdica confirmada de baixa intensidade podem apresentar formas iniciais de doença. Nestes casos, recomenda-se a realização de novo exame em 6 meses nos casos de alta suspeição clínica. Adicionalmente, algumas formas raras genéticas, como Ser177Tyr, Tyr114Cys e Phe64Leu, também podem apresentar captação desproporcionalmente baixa de 99mTc-PYP e ATTR com ecocardiograma e RMC com achados típicos. Nos casos de alta suspeição clínica com 99mTc-PYP Graus 0 ou 1 de Perugini (ausência de captação ou captação com intensidade inferior a das costelas), o ideal é realizar o sequenciamento genético para reduzir o risco de erros diagnósticos com essas raras mutações.^61^ Diante do desenvolvimento de novos tratamentos para a AC, há interesse crescente em avaliar se os métodos de imagem, incluindo a cintilografia, podem ser utilizados para monitorar a resposta terapêutica. No entanto, essa ainda é uma área em investigação, com evidências em fase inicial de consolidação.^62^

Vários estudos observacionais demonstram, de modo consistente, a aplicabilidade da cintilografia no diagnóstico da AC e na identificação do subtipo ATTR^63–66^ (Tabela 4). As principais limitações incluem a escassez de ensaios clínicos randomizados comparando diretamente cintilografia e biópsia para desfechos de acurácia e prognóstico em grandes amostras, bem como a necessidade de validação prospectiva do papel prognóstico em diferentes contextos clínicos e populações. Há recomendação clara de que o exame de cintilografia com 99mTc-PYP, realizado com protocolo adequado e associado à exclusão de gamopatias monoclonais, pode ser suficiente para diagnóstico de ATTR, com benefícios práticos, éticos e econômicos em relação à biópsia (Tabela 5).

**Tabela 4:** Resumo dos principais estudos sobre cintilografia óssea com tecnécio-99m na investigação de amiloidose cardíaca

Estudo/ Referência	População	Sensibilidade (%)	Especificidade (%)	Valor prognóstico	Limitações
Gillmore et al.^66^	Pacientes com suspeita de ATTR sem paraproteína	99-100	86	Alta captação associada a pior prognóstico, mas limitado por dados retrospectivos	Estudo observacional; necessidade de validação prospectiva
Bokhari et al.^63^	Pacientes com suspeita de amiloidose cardíaca	91	92	Sugestão de relação entre captação e eventos cardíacos	Pequeno N; não comparou diretamente com biópsia em todos os casos
Castano et al.^65^	Pacientes com amiloidose cardíaca (diversos centros)	93	89	Captação correlacionada com mortalidade e eventos cardíacos em coorte	Observacional, sem randomização
Ahluwalia et al.^64^	Diversos estudos com suspeita de ATTR	99 (análise visual)	97 (SPECT) 96 (planar visual)	Confirma valor diagnóstico, mas com heterogeneidade devido à variação de prevalência	Heterogeneidade metodológica entre estudos

ATTR: amiloidose por transtirretina; SPECT: tomografia computadorizada por emissão de fóton único (*Single photon emission computed tomography*).

**Tabela 5:** Recomendações para o uso de imagem cardíaca multimodalidade em pacientes com cardiomiopatia amiloide

Recomendação	Força da recomendação	Certeza da evidência
Ecocardiograma transtorácico deve ser realizado como método diagnóstico inicial em pacientes com suspeita clínica de AC. ^27–30^	Forte	Alta
Avaliação do SLG do ventrículo esquerdo deve ser realizada como parte da avaliação ecocardiográfica na AC.^23^	Fraca	Moderada
Pacientes com estenose aórtica importante paradoxal devem realizar complementação com SLG do ventrículo esquerdo.^19,31,32^	Fraca	Baixa
Avaliação do *strain* do ventrículo direito é recomendada para complementação prognóstica em pacientes com AC.^33,34^	Fraca	Baixa
Ecocardiograma seriado é recomendado para acompanhamento da resposta ao tratamento e progressão da AC.^35,36^	Fraca	Moderada
Ecocardiograma com contraste deve ser considerado em casos com janela ecocardiográfica limitada na suspeita de AC.^37^	Fraca	Baixa
Avaliação da função diastólica deve ser realizada na suspeita diagnóstica e no seguimento da AC.^38^	Forte	Alta
Avaliação com Doppler tecidual (E/e') deve ser realizada para avaliação da disfunção diastólica na AC.^39^	Forte	Moderada
A RMC, com avaliação morfofuncional e técnicas de caracterização tecidual, deve ser realizada nos pacientes com suspeita de AC para fins diagnósticos.^54^	Forte	Alta
A avaliação pela RMC com a técnica de realce tardio, T1 nativo e VEC deve ser feita para avaliação prognóstica.^55,56^	Forte	Alta
A RMC seriada pode ser recomendada para acompanhamento da resposta ao tratamento e progressão da cardiomiopatia amiloide.^59^	Forte	Baixa
Para pacientes com suspeita de ATTR, recomenda-se a utilização da cintilografia óssea com 99mTc-PYP como método de diagnóstico, em detrimento da biópsia endomiocárdica, quando na ausência de gamopatias monoclonais. A positividade do exame (Graus 2 ou 3 de Perugini) equivale ao diagnóstico não invasivo de ATTR.^64^	Forte	Moderada

^99m^Tc-PYP: pirofosfato de 99m-tecnécio; AC: amiloidose cardíaca; ATTR: amiloidose por transtirretina; RMC: ressonância magnética cardíaca; SLG: *strain* longitudinal global; VEC: volume extracelular.

A periodicidade recomendada para exames de imagem cardíaca em pacientes com AC deve ser individualizada conforme o quadro clínico, o tipo de amiloidose (AL ou ATTR), resposta ao tratamento, além da disponibilidade dos métodos. Segundo o consenso atuais, recomenda-se:

- Ecocardiografia (incluindo *strain* miocárdico): a cada 6 a 12 meses para monitorar progressão da doença ou resposta ao tratamento, podendo ser antecipada em caso de piora clínica ou alteração de biomarcadores.^58,60^ O *strain* miocárdico deve ser incluído sempre que disponível, pois é sensível para detectar progressão.
- Ressonância magnética cardíaca (RMC): a cada 12 a 24 meses, principalmente se piora clínica e suspeita de progressão da doença baseada em biomarcadores ou ecocardiografia para avaliação detalhada do envolvimento estrutural ou resposta terapêutica.^58,60^ Não há consenso para intervalos menores que 12 meses em pacientes estáveis.
- Cintilografia com radiofármacos (99mTc-PYP/DPD/HMDP): não é recomendada para monitoramento seriado da resposta ao tratamento ou progressão, sendo indicada apenas para diagnóstico inicial e definição do subtipo da amiloidose.^58,60^

**Mensagens-chave**

- A avaliação MM de imagem cardiovascular na AC é imperativa dentro de uma premissa clínica;
- O ecocardiograma é o exame de imagem cardíaca de primeira linha;
- Diante de um fenótipo ecocardiográfico com aumento da espessura, o SLG do VE demonstrando um padrão de disfunção regional que preserva a região apical ("apical sparing") aumenta a possibilidade de AC, embora não seja específico;
- Mesmo sem sinais de hipertensão pulmonar (HP), os parâmetros de função do VD podem estar reduzidos;
- Apesar dos avanços, o ecocardiograma não é capaz de diferenciar a cardiomiopatia amiloide por cadeia leve da transtirretina;
- A piora clínica é esperada com aumento da espessura ventricular, dilatação atrial, redução do volume sistólico ejetado do VE, piora da DD e queda do SLG. A fração de ejeção pode se manter preservada mesmo em estágios avançados;
- Diante de uma resposta terapêutica positiva, o SLG apresenta estabilidade ou, em alguns casos, melhora mais precoce, enquanto os parâmetros morfológicos são tardiamente alterados;
- A RMC fornece, além da avaliação morfofuncional, a caracterização tecidual em pacientes com AC, quantificando a extensão do acometimento miocárdico por meio das técnicas de RT, T1 nativo e VEC. A presença de RT difuso e a elevação acentuada de T1 nativo e VEC são marcadores diagnósticos e prognósticos na AC;
- A cintilografia com 99mTc-PYP, quando realizada com protocolo adequado e associado à exclusão de gamopatias monoclonais, pode ser suficiente para o diagnóstico de ATTR e dispensar a biópsia.

### 2.2. Sarcoidose Cardíaca

A sarcoidose é uma doença inflamatória multissistêmica rara, de fisiopatologia incerta,^67^ caracterizada pela formação de granulomas não caseosos em diferentes tecidos. Possui prevalência em torno de 230 casos por 100.000 habitantes.^68^

As manifestações clínicas incluem um espectro que varia desde casos assintomáticos a uma doença progressiva e recidivante, com sequelas importantes.^69^ O envolvimento intratorácico é o mais frequente, chegando a 97% dos casos, sendo comumente por linfadenopatia intratorácica (87%) ou por infiltração parenquimatosa pulmonar (50%), com sintomas respiratórios descritos em 43% dos pacientes.^70^ Entretanto, também são descritas manifestações cutâneas, oftalmológicas, renais, cardíacas e em sistema nervoso central, entre outros acometimentos menos frequentes.^71^

O envolvimento cardíaco é clinicamente diagnosticado em cerca de 3% dos pacientes com sarcoidose,^72^ embora esteja presente em 27% a 69% dos casos submetidos a autópsia.^73,74^ O septo interventricular é a região mais acometida, seguido pela parede posterior, com expressivo envolvimento também do VD.^75^

As manifestações cardíacas clínicas mais frequentes são IC, bloqueio atrioventricular (BAV) e arritmias ventriculares, presentes em, respectivamente, 52%, 34% e 24% dos casos.^76^ Em alguns casos, a morte cardíaca súbita pode ser a primeira manifestação da doença.^77^

Pacientes com SC frequentemente apresentam envolvimento sistêmico.^72^ Todavia, o acometimento cardíaco pode ocorrer de forma isolada em 18% dos casos e é associado a maior ocorrência de taquicardia ventricular (TV) sustentada e a maior tendência ao desenvolvimento de IC, provavelmente em função de um diagnóstico mais tardio.^78^

Embora a doença pulmonar ainda seja a principal causa de mortalidade por sarcoidose no mundo, o envolvimento cardíaco assume um fator prognóstico importante, sendo a primeira causa de mortalidade no Japão.^79^ Em pacientes tratados, a sobrevida pode alcançar 90% em 10 anos,^80^ de modo que o tratamento com corticoterapia é associado a menores taxas de mortalidade, arritmias sintomáticas e hospitalização por IC, além de melhora da função ventricular esquerda.^81^ Tais dados reforçam a importância do início do tratamento imunossupressor ainda na fase inflamatória, antes do surgimento de lesões fibróticas potencialmente irreversíveis e associadas a pior prognóstico. Para tanto, o diagnóstico precoce é fundamental.

Como o acometimento miocárdico não é homogêneo, a biópsia endomiocárdica, apesar de altamente específica, possui baixa sensibilidade, inferior a 25%.^82–85^ Além disso, não podem ser desconsiderados os riscos inerentes ao procedimento, sendo reportadas complicações em torno de 10% dos casos.^85^ Com o aprimoramento da avaliação MM em imagem cardíaca e dos critérios diagnósticos,^86–88^ que permitem o diagnóstico na ausência de um exame histopatológico miocárdico positivo, o uso rotineiro da biópsia endomiocárdica não é justificado.

#### 2.2.1. Ecocardiografia

De forma geral, o ETT convencional isolado, apesar de amplamente disponível e acessível, apresenta baixa sensibilidade para diagnóstico de SC. Quando combinado ao eletrocardiograma de 12 derivações e à anamnese, apresenta sensibilidade de 64% para diagnóstico de acometimento cardíaco.^89^ Permanece, entretanto, como exame de primeira linha e deve ser realizado em todos os pacientes com diagnóstico de sarcoidose sistêmica, conforme as principais recomendações internacionais.^87,90^

Um estudo realizado na Índia, que avaliou pacientes com diagnóstico estabelecido de sarcoidose extracardíaca, evidenciou que o SLG absoluto do VE < 17,3% isolado teve sensibilidade de 80%, especificidade de 82,61% e valor preditivo negativo de 98,7% para diagnóstico de acometimento cardíaco, enquanto o SLG absoluto do VD < 21% apresentou sensibilidade de 68%, especificidade de 78% e valor preditivo negativo de 98%.^91^

Uma metanálise recente, incluindo 478 pacientes com sarcoidose, mostrou que o SLG do VE e o TAPSE foram significativamente menores em pacientes com acometimento cardíaco, sugerindo possíveis marcadores precoces para o diagnóstico da doença.^92^ Quando comparados a indivíduos saudáveis, pacientes com sarcoidose sem manifestações clínicas sugestivas de acometimento cardíaco também apresentaram redução significativa do SLG do VE em três metanálises.^93–95^ Esses achados sugerem que alterações do *strain* miocárdico podem preceder alterações estruturais ou funcionais detectadas pelo ecocardiograma convencional, favorecendo a detecção precoce de disfunção miocárdica subclínica.

**Figura 6:**
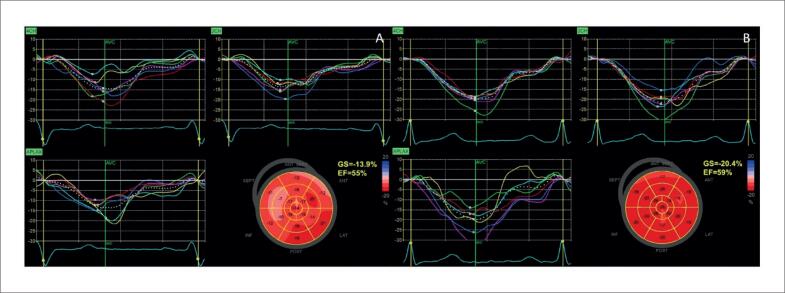
Strain longitudinal global miocárdico pela técnica de speckle tracking em dois pacientes com sarcoidose, com (A) e sem acometimento cardíaco (B). Em A, observa-se redução do strain em segmentos basais das paredes septal, inferior e segmento médio da parede lateral anterior evidenciado pelas cores mais claras.

Na Figura 6, é demonstrado um exemplo de *strain* miocárdico pela técnica de *speckle tracking* em pacientes com diagnóstico de sarcoidose, com e sem acometimento cardíaco, ambos com fração de ejeção do ventrículo esquerdo (FEVE) preservada.

Além de aumentar consideravelmente a acurácia diagnóstica, o *strain* miocárdico também apresenta implicações prognósticas. O *strain* da parede livre do VD reduzido apresentou associação com maior ocorrência de fibrilação ventricular, TV sustentada, BAV avançado e hospitalização por IC.^96^ Houve ainda correlação com RT pelo gadolínio na RMC e captação na tomografia por emissão de pósitrons com ^18^F-fluorodeoxiglicose (PET/TC ^18^F-FDG).^96^ O SLG do VE também mostrou valor incremental na avaliação prognóstica de pacientes com SC, sendo associado de forma independente a um desfecho composto por mortalidade, TV, hospitalização por IC e transplante cardíaco, além de associação com a presença de captação no PET/TC ^18^F-FDG.^97^

**Figura 7:**
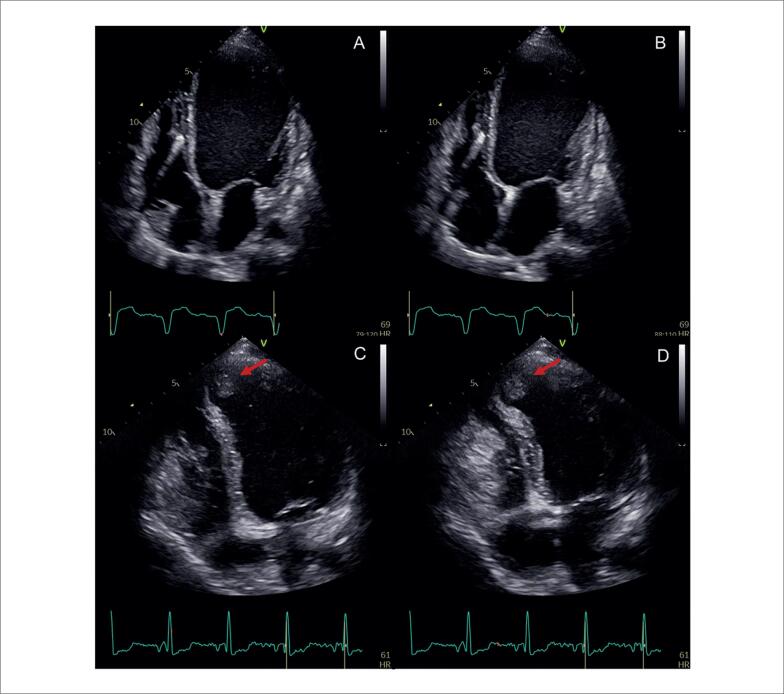
Diferentes padrões de disfunção ventricular esquerda em pacientes com sarcoidose cardíaca submetidos ao ecocardiograma transtorácico. Janela apical de quatro câmaras, em diástole (A e C) e sístole (B e D). Em A e B, evidenciado acometimento extenso do ventrículo esquerdo, com afilamentos focais e disfunção ventricular esquerda importante. Em C e D, evidenciada disfunção segmentar com formação de aneurisma apical e trombo (seta) em seu interior.

Alguns achados menos sensíveis, porém altamente específicos e com impacto prognóstico, também devem ser ressaltados. A disfunção ventricular esquerda com FEVE abaixo de 40% é descrita em 21% dos casos de SC, enquanto anormalidades segmentares são encontradas em até 36% dos pacientes,^98^ com possível evolução para áreas de discinesia e aneurismas. Ambos foram associados a maior ocorrência de mortalidade por todas as causas e de arritmia ventricular grave,^98^ sendo presentes nos critérios diagnósticos da *Japanese Circulation Society* de 2016.^88^ No entanto, somente a FEVE reduzida é contemplada nos critérios diagnósticos de 2014.^86,87^ Na Figura 7, são demonstrados exemplos de ecocardiogramas transtorácicos de pacientes com SC, com diferentes padrões de disfunção ventricular esquerda.

**Figura 8:**
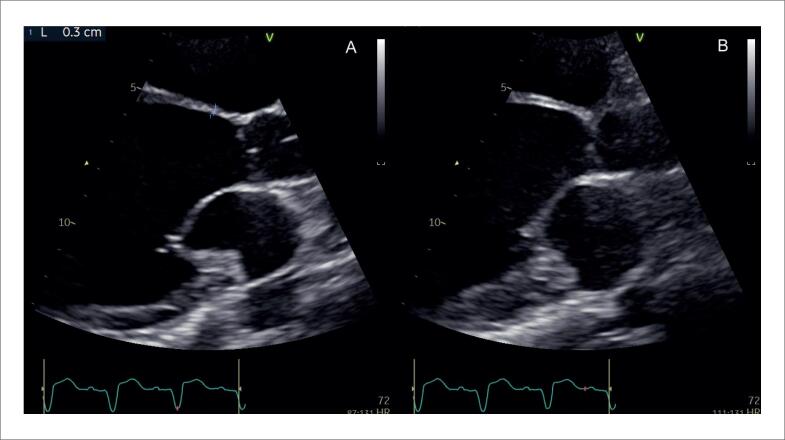
Exemplo de afilamento do segmento basal do septo interventricular medindo 3 mm, ao ecocardiograma transtorácico, na janela paraesternal de eixo longo, em diástole (A) e sístole (B).

O afilamento do segmento basal do septo interventricular (Figura 8), definido como uma espessura ≤ 4 mm, é descrito em 28% dos pacientes com SC.^99^ Foi considerado determinante, independente de um desfecho composto por mortalidade por todas as causas, arritmia ventricular sintomática, bradiarritmia com necessidade de implante de marcapasso e admissão por IC.^99^ Também foi associado ao surgimento de disfunção ventricular esquerda em um seguimento mediano de 7,5 anos.^100^

**Figura 9:**
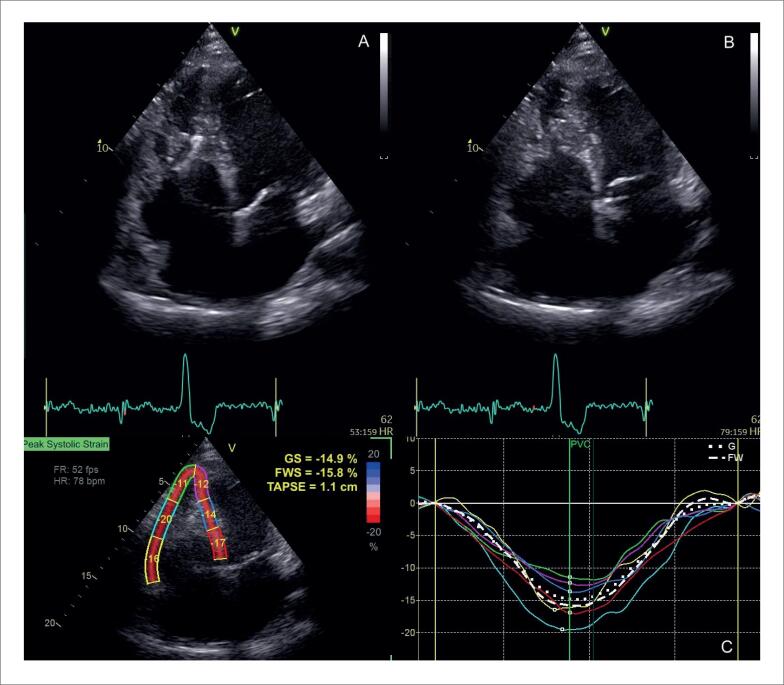
Disfunção do ventrículo direito em paciente com sarcoidose cardíaca ao ecocardiograma transtorácico, sem hipertensão pulmonar, por provável acometimento primário. Janela apical quatro câmaras focada no ventrículo direito, em diástole (A) e sístole (B). Em C, strain longitudinal global miocárdico do ventrículo direito pela técnica de speckle tracking avaliado em −14,9%.

O acometimento do VD pode ocorrer tanto de forma primária, pela infiltração granulomatosa de seu miocárdio, descrita em 34% do casos^76^ (Figura 9), como de forma secundária, pela HP associada ao acometimento primário dos pulmões ou a doença ventricular esquerda avançada. Na ausência de HP ou DD, a disfunção sistólica do VD pelo ecocardiograma possui sensibilidade de 10 a 47% e especificidade de 82 a 99% para diagnóstico de RT.^90^

**Figura 10:**
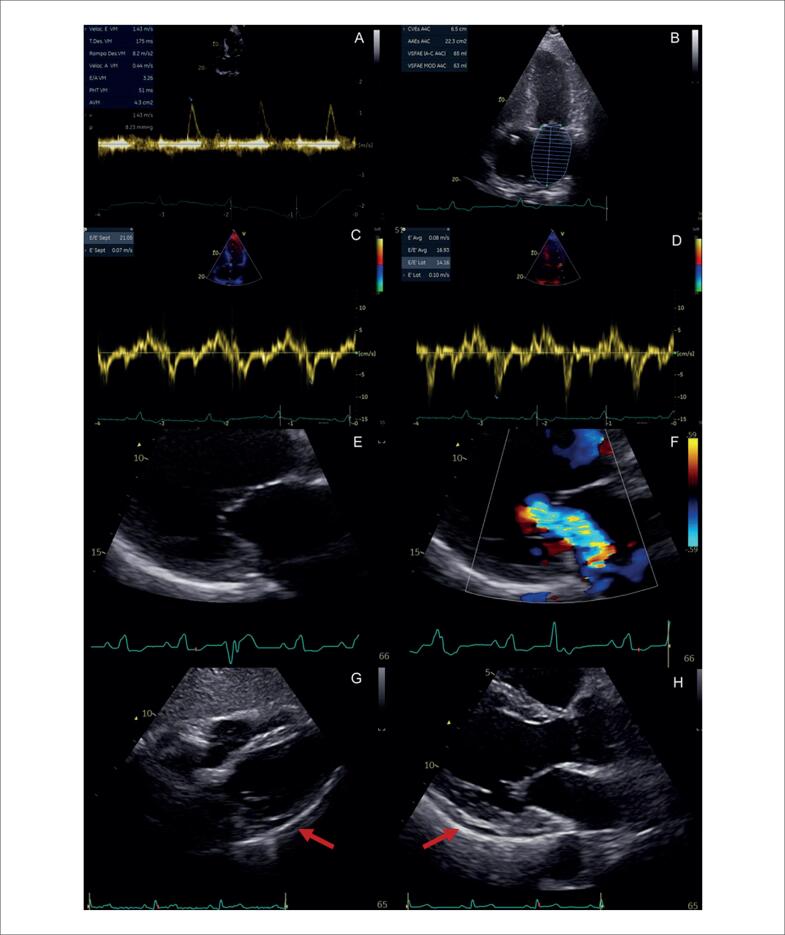
Alterações menos específicas de acometimento cardíaco por sarcoidose ao ecocardiograma transtorácico. Em A, B, C e D, é caracterizada disfunção diastólica de grau restritivo (relação E/A = 3,2, volume indexado do átrio esquerdo de 39 mL/m² e relação E/e’ = 16,9). A: Doppler espectral do influxo mitral. B: Volume do átrio esquerdo avaliado pelo método de Simpson, na janela apical 4-câmaras. C: Doppler tecidual do anel mitral medial. D: Doppler tecidual do anel mitral lateral. Em E e F, evidencia-se valva mitral e insuficiência na janela paraesternal de eixo longo focada na valva mitral. E: Modo bidimensional evidenciando falha de coaptação associada a tracionamento apical das cúspides e dilatação do anel mitral. F: Análise pelo Doppler colorido evidenciando jato de regurgitação mitral importante. G e H: observa-se derrame pericárdico localizado posteriormente, de grau discreto (setas), nas janelas subcostal de quatro câmaras (G) e paraesternal de eixo longo (H).

Outras alterações menos específicas incluem DD, insuficiência mitral e acometimento pericárdico (Figura 10). Devido à inflamação crônica no miocárdio, ocorre aumento da rigidez do VE, com alteração de relaxamento inicialmente e aumento das pressões de enchimento em casos mais avançados.^101^ Derrames pericárdicos mínimos são descritos em 20% dos pacientes com SC,^102^ mas também é descrita a evolução para pericardite constritiva e tamponamento cardíaco.^103^

Já a insuficiência mitral é secundária na maioria dos casos, com cúspides de aspecto morfológico e movimentação normais.^104^ Pode ocorrer por dilatação do anel mitral em decorrência da dilatação atrial esquerda associada à DD, mas também por tracionamento apical secundário à disfunção ventricular esquerda. Recentemente, foi demonstrada evidência de captação nos músculos papilares em 78% dos casos com regurgitação mitral moderada ou importante, avaliados pelo PET/TC ^18^F-FDG, com 37% dos pacientes apresentando redução em pelo menos 1 grau de gravidade após tratamento imunossupressor.^104^

#### 2.2.2. Ressonância Magnética Cardíaca

A RMC é um método não invasivo e altamente sensível para a avaliação da SC, fornecendo informações anatômicas, funcionais e teciduais. Na avaliação morfofuncional, nas sequências de cinerressonância (cineRM), pode ser normal em estágio iniciais, mas, progressivamente, é possível notar a presença de espessamento miocárdico, áreas de hipocinesia regional e, em casos avançados, dilatação e disfunção ventricular.^105^ A cineRM também é capaz de identificar aneurismas ventriculares, derrames pericárdicos e alterações nas valvas cardíacas – achados que podem estar presentes em indivíduos com SC.^106^

**Figura 11:**
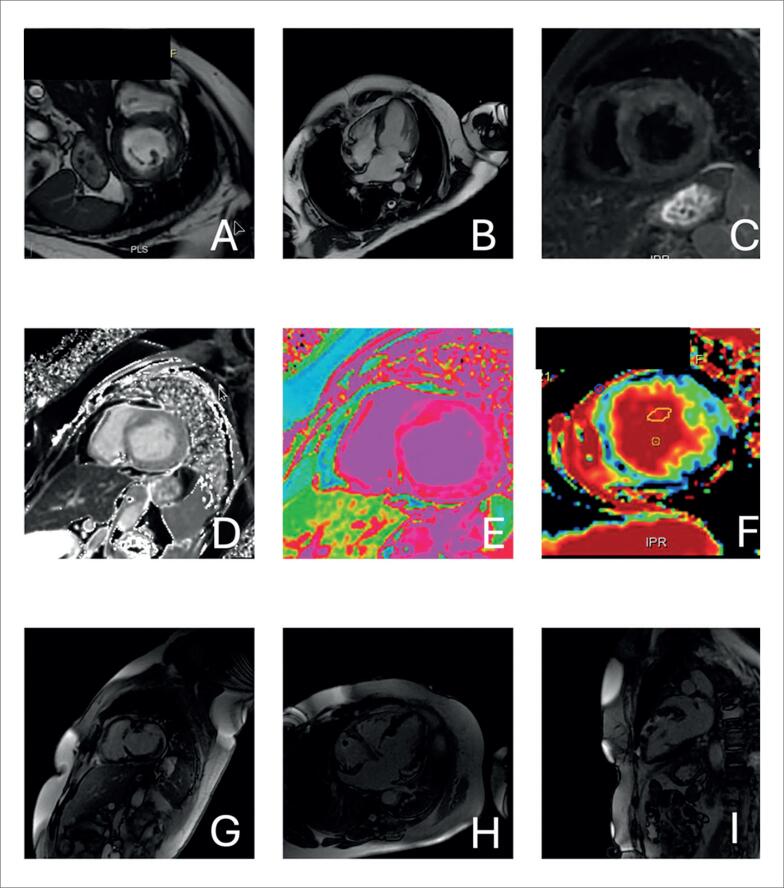
Imagens de ressonância magnética cardíaca de paciente com sarcoidose cardíaca. A e B: imagens de cinerressonância com discreto aumento da espessura miocárdica. C: imagens de sangue escura em Triple IR com hipersinal anterior que sugere edema. D, E e F: sequências de mapeamento de T1 nativo (D e E) e volume extracelular (F) com valores aumentados. G, H e I: imagens de realce tardio evidenciando fibrose multifocal, com diferentes padrões (transmural, mesocárdica e epicárdica), em diferentes segmentos do ventrículo esquerdo.

Entre os achados clássicos da RMC, estão a presença de RT em padrões não isquêmicos. A presença de RT em segmentos basais das paredes lateral e septal – especialmente de padrões mesocárdico ou subepicárdico – é considerada a manifestação mais frequente da SC.^107^ Contudo, o RT também foi identificado em outras áreas do miocárdio, podendo envolver o subendocárdio e até mesmo com padrão transmural.^105,106,108,109^ A técnica de RT foi capaz de detectar lesões focais em 20 a 30% dos pacientes com diagnóstico confirmado por biópsia, as quais foram compatíveis com granulomas não caseosos.^73,109^ A extensão do RT se relaciona com alterações estruturais cardíacas, incluindo redução da fração de ejeção, dilatação do VE e ocorrência de TV não sustentada – fatores já reconhecidos como indicadores de risco para morte súbita cardíaca^106^ (Figura 11).

O avanço dos mapas multiparamétricos, especialmente T1, T2 e o VEC, ampliou de forma significativa a acurácia diagnóstica e o monitoramento da atividade inflamatória na sarcoidose.^105,110^ O mapeamento de T2 é sensível à presença de edema miocárdico, que reflete inflamação ativa. Na SC, valores elevados de T2 estão associados à fase aguda da doença, caracterizada por granulomas ativos e infiltração celular (Figura 11). O aumento focal ou difuso dos valores de T2 pode ocorrer mesmo na ausência de RT, tornando-se um potencial marcador precoce de inflamação antes do desenvolvimento de fibrose.^111^

Na SC, valores elevados de VEC refletem tanto a inflamação quanto a fibrose crônica, e sua combinação com os mapas de T1 e T2 permite distinguir as diferentes fases da doença.^112^ A medição do VEC se mostra particularmente útil no acompanhamento longitudinal dos pacientes, permitindo avaliar a resposta à terapia imunossupressora e identificar progressão da doença mesmo na ausência de alterações morfológicas evidentes^73^ (Figura 11). A combinação dos valores de T1, T2 e VEC pode, didaticamente, auxiliar a identificar os diferentes estágios da SC:

– T2 elevado com T1/VEC normais: fase aguda inflamatória.
– T1/VEC elevados com T2 normal: fibrose crônica sem inflamação ativa.
– Todos os valores elevados: inflamação e fibrose coexistentes, sugerindo doença ativa e mais avançada.

Essa abordagem integrada dos achados clássicos da RMC (cineRM e RT) e a utilização de mapas multiparamétricos, é uma ferramenta útil tanto para o diagnóstico precoce e avaliação prognóstica, quanto para o monitoramento terapêutico, sendo particularmente benéfica em pacientes com sintomas inespecíficos ou exames complementares inconclusivos.^110,111,113,114^

#### 2.2.3. Papel do PET/CT com ^18^F-FDG

A tomografia por emissão de pósitrons associada a tomografia computadorizada com fluordesoxiglicose-flúor-18 (PET/CT com ^18^F-FDG, do inglês *18-F-Fluorodeoxyglucose Positron Emission Tomography/Computed Tomography*) revolucionou a avaliação da SC por identificar inflamação ativa no miocárdio *in vivo*, o que não é possível com exames puramente morfológicos. Esse método é recomendado por especialistas tanto para o diagnóstico quanto para o acompanhamento e ajuste da terapia imunossupressora.^115,116^

**Indicação para diagnóstico e monitoramento**: o PET/CT com ^18^F-FDG deve ser indicado em pacientes com suspeita de SC, especialmente quando houver bloqueios atrioventriculares, arritmias ventriculares ou disfunção ventricular de etiologia indeterminada.^115–118^ Além disso, ele é recomendado para monitorar a resposta ao tratamento com imunossupressor, sendo repetido em intervalos de 3 a 6 meses de acordo com o quadro clínico.^115,116,119,120^

**Importância prognóstica – envolvimento do ventrículo direito**: pacientes com captação de 18-FDG no VD apresentam risco significativamente maior de eventos adversos – incluindo arritmias e morte súbita.^121,122^ Dessa forma, o reconhecimento precoce desse achado é crucial para a intervenção terapêutica.

É importante ressaltar que a sarcoidose é uma doença sistêmica e pode afetar múltiplos órgãos. O PET/CT com ^18^F-FDG de corpo inteiro permite identificar o envolvimento extracardíaco, o que contribui tanto para a confirmação diagnóstica quanto para a escolha terapêutica.^123,124^ No entanto, é fundamental o preparo para o exame, uma vez que a acurácia desse exame depende do preparo adequado para suprimir a captação fisiológica de glicose pelo miocárdio. As recomendações incluem:^115,116,125,126^

- Dieta rica em gorduras e pobre em carboidratos nas 24 a 72 horas que antecedem o exame;
- Jejum de pelo menos 12 horas;
- Dosagem do beta-hidroxibutirato (BHB) para garantir maior especificidade metabólica (BHB ≥ 0,335 mmol/L prediz supressão adequada).^127,128^

**Figura 12:**
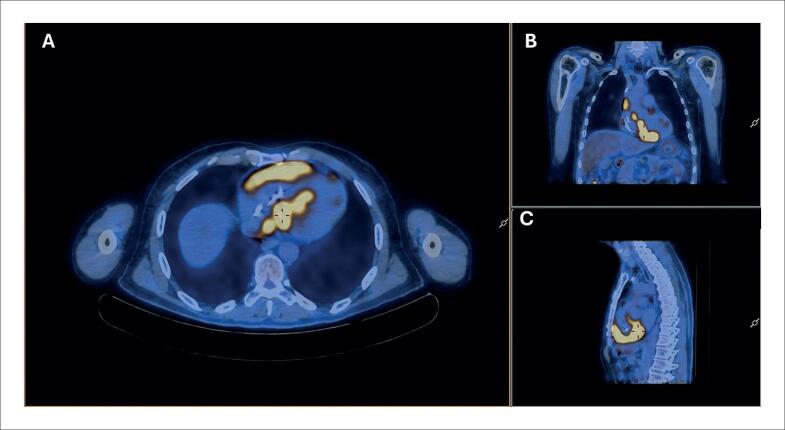
^18^F-FDG PET/CT em paciente com suspeita de sarcoidose cardíaca. (A) Corte axial demonstrando captação focal e heterogênea de ^18^F-FDG no miocárdio do ventrículo esquerdo, predominante no septo interventricular, com extensão para a parede do ventrículo direito, compatível com inflamação ativa. (B) Reconstrução coronal evidenciando a extensão da captação miocárdica. (C) Reconstrução sagital confirmando o padrão focal de hipermetabolismo miocárdico.

Em relação à avaliação da perfusão e metabolismo cardíaco relacionando esses achados ao espectro clínico da SC, uma metanálise publicada em 2020 e com 17 estudos (891 pacientes) mostrou uma sensibilidade de 84% e especificidade de 83%.^117^ A avaliação combinada de perfusão e metabolismo pelo PET/CT com ^18^F-FDG distingue inflamação ativa (alta captação de FDG) de áreas com fibrose (defeito de perfusão sem captação), permitindo uma estratificação mais precisa e uma decisão terapêutica individualizada (Figura 12).^116–118^ Quando disponível, a combinação com RMC aumenta ainda mais a acurácia diagnóstica (Tabela 6).^129^

**Tabela 6:** Recomendações para o uso de imagem cardíaca multimodalidade na sarcoidose cardíaca

Recomendação	Força da recomendação	Certeza da evidência
Recomenda-se a realização do ecocardiograma transtorácico com *strain* miocárdico por *speckle tracking* para todos os pacientes com diagnóstico de sarcoidose sistêmica.^93–95^	Fraca	Baixa
A RMC deve ser realizada em pacientes com suspeita clínica de sarcoidose cardíaca para avaliação diagnóstica.^112,114^	Forte	Moderada
O PET/CT com ^18^F-FDG deve ser realizado em pacientes com suspeita clínica de sarcoidose cardíaca para confirmação diagnóstica.^115–118^	Forte	Moderada
O PET/CT com ^18^F-FDG deve ser realizado no monitoramento da resposta ao tratamento.^115,116,119,120^	Fraca	Baixa
O PET/CT com ^18^F-FDG deve, sempre que possível, ser integrado com RMC.^129^	Forte	Moderada
O exame de PET/CT com ^18^F-FDG para pesquisa de sarcoidose cardíaca deve sempre ser feito com preparo cardíaco rigoroso do paciente.^115,116,125^	Forte	Alta

PET/CT com ^18^f-fdg: tomografia por emissão de pósitrons associada a tomografia computadorizada com fluordesoxiglicose-flúor-18 (do inglês f*luorodeoxyglucose positron emission tomography/computed tomography*); RMC: ressonância magnética cardíaca.

A periodicidade recomendada para exames de imagem cardíaca em pacientes com sarcoidose cardíaca deve ser individualizada conforme atividade da doença, sintomas, resposta ao tratamento e risco de eventos. Não há consenso internacional sobre intervalos fixos, mas as principais sociedades recomendam avaliação ecocardiográfica (com análise do *strain*) a cada 6-12 meses, ressonância magnética cardíaca anual ou à critério clínico, e PET-FDG a cada 6-12 meses em casos de doença ativa ou ajuste terapêutico.^130–133^

**Mensagens-chave**

- Recomenda-se a realização do ETT com *strain* miocárdico por *speckle tracking* para todos os pacientes com diagnóstico de sarcoidose sistêmica, com o objetivo de aumentar a acurácia diagnóstica e a avaliação prognóstica. Em pacientes com *strain* miocárdico alterado, é indicado prosseguir a investigação com métodos de imagem avançados.
- Disfunção ventricular esquerda e anormalidades de contratilidade segmentar são achados mais tardios, porém contemplados em critérios diagnósticos e associados a maior ocorrência de mortalidade por todas as causas e de arritmias ventriculares graves.
- Em pacientes com suspeita de SC, é aconselhável fazer a avaliação de possível afilamento da porção basal do septo interventricular, definido como uma espessura < 4 mm na janela paraesternal de eixo longo, como marcador prognóstico.
- O PET/CT com ^18^F-FDG é essencial para o diagnóstico e acompanhamento de pacientes com suspeita ou confirmação de SC,^115,116^ principalmente diante de BAV inexplicável, arritmia ventricular frequente ou disfunção ventricular inexplicada, além de permitir a avaliação sistêmica da doença.^58,61,65,134^
- O PET/CT com ^18^F-FDG apresenta impacto prognóstico, sobretudo em casos de envolvimento do VD.^121,122^ A avaliação do preparo do paciente é indispensável para evitar a captação miocárdica fisiológica e garantir a interpretação correta.^125–128^ Esse método possibilita ajustes na terapia de acordo com a resposta inflamatória.^119,134^
- A combinação com RMC aprimora a diferenciação entre inflamação ativa e fibrose.^129^

## 3. Aplicações da Multimodalidade em Imagem Cardíaca nas Doenças Metabólicas

### 3.1. Diabete Mellitus

O diabetes mellitus (DM) é uma das doenças crônicas mais prevalentes no mundo, contribuindo de forma expressiva para a morbimortalidade cardiovascular.^135^ O comprometimento miocárdico em indivíduos com DM, particularmente no DM tipo 2 (DM2), pode resultar de comorbidades frequentemente associadas, como a doença arterial coronariana (DAC) e a HAS. No entanto, reconhece-se a possibilidade de que mecanismos patofisiológicos próprios do DM afetem diretamente a estrutura e a função miocárdicas.^136^ Alterações glicêmicas, como resistência à insulina e disfunção no metabolismo da glicose, promovem efeitos sistêmicos e impactam diretamente o cardiomiócito, comprometendo a utilização de substratos energéticos, a função mitocondrial e o acoplamento excitação-contração.^137^ Nesse contexto, a cardiomiopatia diabética (CMD) refere-se à disfunção cardíaca observada em pacientes com DM na ausência de outras doenças cardiovasculares como DAC, HAS, doença valvar ou congênita.^137,138^

A ecocardiografia desempenha um papel importante na avaliação da CMD, identificando os três tipos de acometimento miocárdico: disfunção sistólica do VE, DD e alterações na geometria ventricular esquerda, além de auxiliar na predição de desfechos cardiovasculares.^135–138^

Embora a FEVE reduzida seja reconhecida como confirmatória de IC,^137^ em pacientes com sintomas sugestivos, quase metade dos pacientes com IC e DM2 apresentam IC com fração de ejeção preservada (ICFEp), sendo mais frequente em pacientes idosos, hipertensos e mulheres com DM2. A ICFEp normalmente está associada a complicações menos graves nas fases iniciais do DM2, enquanto a IC com fração de ejeção reduzida (ICFEr) está relacionada a complicações mais graves do DM2. Isso sugere que a gravidade e a duração da hiperglicemia são fatores importantes para o desenvolvimento da disfunção ventricular esquerda.^139^

A análise da deformação miocárdica (*strain*) pelas técnicas de *speckle tracking* bidimensional (2D-STE) e tridimensional (3D-STE) tem sido empregada para detectar disfunção sistólica subclínica em pacientes diabéticos. Estudos mostram que pacientes com DM apresentam valores de SLG do VE reduzidos, indicando disfunção sistólica, mesmo na ausência de sintomas clínicos evidentes.^135,140^ Nesse contexto, a redução do SLG do VE é um marcador sensível de disfunção sistólica, detectando comprometimento funcional mesmo com fração de ejeção preservada, e associa-se à ocorrência de IC, mortalidade e remodelamento do VE.^141,142^

A DD do VE é muito comum em pacientes com DM assintomáticos e aparentemente saudáveis, estando presente em pelo menos 50% dessa população.^137^ A DD Grau 1, sem elevação das pressões de enchimento, apresenta fraca associação com o prognóstico. No entanto, a progressão da DD, com aumento da pressão de enchimento do VE (estimada pela razão entre as velocidades das ondas E/eʹ), está associada a desfechos adversos, como progressão para IC e mortalidade, além de apresentar-se consistentemente elevada em pacientes com DM2, em comparação com controles.^143,144^

O remodelamento concêntrico do VE representa uma alteração precoce na estrutura miocárdica e está associado à disfunção sistólica sutil, decorrente de esteatose e alteração na cadeia energética miocárdica.^145^ A hipertrofia representa um aumento da massa miocárdica, sendo que parte desse aumento é atribuída ao VEC, refletindo deposição de colágeno e fibrose, fatores que, por sua vez, estão associados a piores desfechos.^146,147^ A hiperinsulinemia, decorrente da resistência à insulina, também pode induzir a hipertrofia dos miócitos na presença de aumento da pós-carga.^148^

O índice de massa do ventrículo esquerdo (IMVE) e o volume indexado do átrio esquerdo (AE) têm sido associados ao aumento do risco de mortalidade geral e cardiovascular em pacientes com DM2. Esses parâmetros estruturais, juntamente com indicadores de função diastólica (relações E/A e E/e’) são preditores importantes de mortalidade, ressaltando o valor prognóstico da ecocardiografia nessa população.^149^ Além disso, a DD é frequentemente associada à dilatação do AE, sendo a fibrilação atrial uma complicação do diabetes atribuída ao aumento da pressão de enchimento do VE e à distensão atrial, sugerindo uma possível atriopatia, de acordo com outras evidências disponíveis na literatura.^150^

A heterogeneidade dos pacientes com disfunção miocárdica assintomática e DM pode ter implicações importantes para a terapêutica. Uma análise recente identificou três padrões nessa população: 1) um grupo com função sistólica e diastólica menos comprometida (predominantemente homens), associado a prognóstico favorável; 2) pacientes com obesidade e hipertensão com DD (em sua maioria mulheres); e 3) um grupo com hipertrofia ventricular esquerda (HVE) e disfunção sistólica (principalmente homens); os dois últimos grupos apresentam prognóstico semelhante e menos favorável.^151^

A RMC permite a detecção de alterações miocárdicas e ateromatose precoces, sendo uma modalidade precisa para avaliar a função cardíaca em repouso e estresse, consistindo em método útil para análise da macro/microcirculação em pacientes com DM.^152–156^

O estudo CE-MARC (*Clinical Evaluation of Magnetic Resonance Imaging in Coronary Heart Disease*) comparou diretamente a RMC à cintilografia na detecção de DAC, utilizando a cinecoronariografia como padrão-ouro, e observou a superioridade da RMC em relação à medicina nuclear.^157^ Neste documento, não serão abordados detalhes da avaliação de DAC pelos métodos de imagem.

O RT é a técnica de escolha para a detecção e quantificação da fibrose miocárdica. No entanto, apresenta limitações para avaliar fibrose difusa, pois depende da diferença de intensidade de sinal entre o miocárdio normal e o cicatricial.^158^ Para superar essa limitação, foram desenvolvidas técnicas de imagem paramétrica, como o mapa de T1 e a quantificação do VEC.

O mapa de T1 – obtido antes (nativo) e após a infusão de contraste – permite a avaliação quantitativa dos valores de T1 no miocárdio, identificando a presença de fibrose difusa e intersticial, que não pode ser detectada por biomarcadores circulantes ou pelo RT.^158^ O mapa de T1 pós-contraste é utilizado em conjunto com o mapa pré-contraste para calcular o VEC do miocárdio. Em pacientes com DM, aqueles com VEC elevado apresentam maior risco de eventos cardiovasculares.^159^

As imagens ponderadas em T2 detectam edema miocárdico, refletindo a resposta aguda a inflamação (miocardite) ou isquemia e permitem a quantificação precisa e acompanhamento de sua evolução ao longo do tempo.^158^ Lesões no miocárdio apresentam hipersinal em T2 em comparação ao músculo esquelético e não são especificas para detecção de CMD (Tabela 7).

**Tabela 7:** Recomendações para o uso de imagem cardíaca multimodalidade em pacientes com Diabetes Mellitus

Recomendação	Força da recomendação	Certeza da evidência
O ecocardiograma transtorácico deve ser realizado como primeira linha na avaliação de pacientes com suspeita de insuficiência cardíaca.^137,138,144^	Forte	Alta
A função sistólica deve ser avaliada com o uso do *strain* longitudinal global do ventrículo esquerdo na suspeita de disfunção subclínica em janelas acústicas adequadas.^135,140–143^	Fraca	Moderada

**Mensagens-chave**

- A CMD refere-se à disfunção cardíaca observada em pacientes com DM, na ausência de outras doenças cardiovasculares, como DAC, HAS, doença valvar ou congênita.
- A ICFEp é a apresentação clínica inicial mais comum da CMD, enquanto a forma com ICFEr representa uma complicação mais tardia e grave do DM.
- O SLG do VE reduzido indica disfunção sistólica subclínica, mesmo com fração de ejeção preservada, sendo um marcador sensível associado à IC, mortalidade e remodelamento ventricular.
- A progressão da DD, com relação E/eʹ consistentemente elevada, está associada a piores desfechos.
- O aumento do IMVE e do volume do AE são preditores de mortalidade.
- Alterações metabólicas precoces dos miócitos podem ser detectadas pela RMC. A avaliação direta do miocárdio pela RMC pode ser um marcador da gravidade da doença.^160^
- A doença microvascular, presente no DM, é quantificável por RMC e PET, sendo que este último apresenta maior custo e menor disponibilidade, além de utilizar radiação.^161^
- A RMC permite a detecção precoce de alterações cardíacas em pacientes com DM, definindo precocemente a etiologia da IC (doença microvascular, fibrose subendocárdica/transmural, inflamação, infarto).
- A RMC é capaz de identificar infartos ocultos em até 28% dos pacientes com DM.^161^

### 3.2. Obesidade

A obesidade é altamente prevalente e representa um fator de risco independente para várias doenças cardiovasculares.^162^ Houve, recentemente, uma nova abordagem para a classificação de obesidade, fora do escopo deste documento, cuja leitura é recomendada.^163^ Entre as alterações hemodinâmicas e estruturais associadas a essa condição, estão a HVE, dilatação atrial e ventricular esquerda, DD e disfunção sistólica subclínica. Alterações precoces do VD em pacientes obesos, mas sem apneia do sono, foram descritas precedendo alterações do VE.^164^

Este documento apresenta recomendações práticas e atualizadas sobre o uso de ecocardiograma e RMC na obesidade, baseado nas melhores evidências científicas disponíveis e apresentadas pelo sistema GRADE (*Grading of Recommendations, Assessment, Development and Evaluation*); o resumo das principais recomendações encontra-se na Tabela 8.

**Tabela 8:** Recomendações para o uso do ecocardiograma em pacientes com obesidade

Recomendação	Força da recomendação	Certeza da evidência
O ecocardiograma transtorácico deve ser realizado como primeira linha na avaliação cardiovascular de pacientes obesos com sintomas ou fatores de risco.^162,165^	Forte	Alta
O uso de agentes de realce ultrassonográfico deve ser considerado em pacientes obesos com janelas acústicas limitadas para otimizar a acurácia diagnóstica.^165,168^	Fraca	Baixa
O ecocardiograma transesofágico é seguro e recomendado no paciente obeso que apresentar indicações para realização.^165^	Fraca	Baixa
O ecocardiograma de estresse pode ser utilizado no paciente obeso para avaliação da doença arterial coronariana.^168^	Fraca	Moderada
A função sistólica deve ser avaliada com o uso do *strain* longitudinal global na suspeita de disfunção subclínica em janelas acústicas adequadas.^171^	Fraca	Moderada
A indexação de câmaras cardíacas deve ser feita preferencialmente por altura, evitando o uso de superfície corporal em obesos.^173,174^	Fraca	Baixa
A quantificação do tecido adiposo epicárdico pelo ecocardiograma pode ser realizada como marcador de eventos cardiovasculares. Valores acima de 10 mm indicam risco alto.^175^	Fraca	Moderada
O ventrículo direito deve ser avaliado rotineiramente em obesos, mesmo na ausência de hipertensão pulmonar, devido à sua alteração ocorrer precocemente, antecedendo o ventrículo esquerdo.^164,165^	Fraca	Moderada

O ETT é essencial na avaliação cardiovascular não invasiva dessa população, por ser amplamente disponível, seguro e de custo acessível.^165^ Contudo, há limitações técnicas ocasionadas por janela acústica nesse grupo de pacientes, sendo necessário o uso de agentes de realce ultrassonográfico (ARU) ou complementação com outras modalidades como a RMC.^166^ Além da análise morfológica e funcional cardíaca, a RMC permite a caracterização tecidual, sendo especialmente útil na detecção precoce de alterações subclínicas associadas à obesidade. A RMC, por sua vez, mantém ótima qualidade de imagem e precisão diagnóstica, mesmo em pacientes com índice de massa corporal (IMC) médio de 50,3 ± 5,9 kg/m².^167^ O ecocardiograma transesofágico (ETE) também é uma alternativa válida em casos de janela limitada, sem aumento do risco causado pela sedação, desde que observadas as recomendações como elevação cefálica e monitorização com capnografia e oximetria.^166^

Outra modalidade eficaz é o ecocardiograma sob estresse farmacológico (EcoE), especialmente na suspeita de DAC.^168^ O estudo SUMO (*Stress Ultrasonography in Morbid Obesity*) avaliou 205 pacientes com obesidade mórbida e mostrou que, embora 96% necessitassem do uso de ARU para avaliação segmentar adequada, o EcoE é seguro e com valor preditivo positivo semelhante aos descritos em não obesos.^167^ Em pacientes obesos com DAC, a RMC tem também papel prognóstico. O estudo SPINS (*Stress CMR Perfusion Imaging in the United States*) demonstrou que o déficit reversível de perfusão e o RT são marcadores independentes de mortalidade em pacientes obesos.^169^ Resultados mais recentes confirmaram esses achados em pacientes com IMC > 40 kg/m².^170^

Na presença de janela acústica de boa qualidade, o SLG do VE permite análises complementares à FEVE para avaliar função sistólica e mecânica na obesidade.^171^ O SLG do VE permite detectar disfunção subclínica precoce, contribuindo para tratamento de IC precocemente ou como adjunto para o diagnóstico de ICFEp.^165,171,172^

A indexação de diâmetros e volumes das câmaras cardíacas na obesidade é controversa, sobretudo do AE. A indexação pela superfície corporal pode subestimar a dilatação. Para maior precisão, recomenda-se indexação alométrica por altura. Evidências apontam que essa métrica supera a indexação isométrica por superfície corporal.^173,174^ Valores sugeridos com as possibilidades de indexação estão descritos na Tabela 9.^173,174^

**Tabela 9:** Fórmulas para indexação do átrio esquerdo: vantagens e desvantagens

Método de indexação	Tipo	Vantagens	Desvantagens	Valor normal
**VAE/SC (m²)**	Isométrico	Padrão atual das diretrizes; fácil de aplicar	Subestima em obesos; depende de gordura corporal	< 34 mL/m².^173^
**VAE/altura**	Isométrico	Simples, melhora discreta sobre a indexação por superfície corporal	Influenciado por estatura, sem muitos estudos	Sem valor de corte definido.^174^
**VAE/altura** ^2,7^	Alométrico	Geometricamente proporcional ao volume cardíaco	Exponente empírico; não derivado de população saudável	~ < 16 m/m.^2,7,169,170^
**VAE/altura** ^1,72^	Alométrico	Derivado de estudo populacional saudável; melhor preditor de fibrilação	Cálculo mais complexo	< 22,1 mL/m.^1,72,173^
**AE/altura** ^1,56^	Alométrico	Mais sensível que o volume máximo; melhor correlação com FA	Ainda pouco utilizado na prática clínica	< 12,7 mL/m.^1,56,173^

FA: fibrilação atrial; VAE: volume do átrio esquerdo.

A espessura do tecido adiposo epicárdico (TAE), pouco usada na prática, tem boa correlação com a DAC.^175^ O TAE é visualizado como espaço sem ecos entre o miocárdio e o pericárdio visceral. Mede-se perpendicular à parede livre do VD ao fim da sístole, em cortes paraesternais longitudinal ou transverso. Valores acima de 10 mm associam-se a maior risco cardiovascular.^176^

Evidências recentes mostram impacto positivo da cirurgia bariátrica e da semaglutida na estrutura e função cardíaca de obesos resultantes da perda ponderal e controle metabólico, promovendo regressão significativa da hipertrofia miocárdica ventricular esquerda. O acompanhamento ecocardiográfico seriado é recomendado e evidencia redução da massa do VE, melhora da diástole, redução do AE e aumento do SLG, sem alteração significativa na FEVE.^177–179^ A RMC também está indicada para monitorar alterações estruturais pós-operatórias de cirurgia bariátrica.^180^

A realização de exames de imagem cardíaca em pacientes com diabetes mellitus e obesidade deve ser **individualizada**, baseada em sintomas, risco cardiovascular e alterações clínicas. Não há recomendação para exames de imagem cardíaca de rotina em pacientes assintomáticos apenas por terem diabetes ou obesidade, segundo as diretrizes da *American Diabetes Association* e da *American Heart Association*.^133,182,183^

A **Ecocardiografia** (incluindo *strain* miocárdico) podendo ser repetida conforme evolução clínica, geralmente em intervalos de 6 a 12 meses em pacientes com doença estabelecida ou sintomas persistentes.^133,183,184^ Em pacientes assintomáticos, a ecocardiografia não é recomendada de rotina, mas pode ser considerada em casos de alto risco ou alterações em biomarcadores.^137,185^

A **Ressonância magnética cardíaca** e **cintilografia** (incluindo perfusão miocárdica) não são recomendadas para rastreamento periódico em pacientes assintomáticos com diabetes e obesidade. Esses exames estão indicados em pacientes sintomáticos para avaliação de doença estrutural, ou quando o ecocardiograma é inconclusivo devido à limitação técnica pelo excesso de tecido adiposo.^137,165,186^ A periodicidade depende da indicação clínica e não existe intervalo fixo recomendado para acompanhamento em pacientes sem sintomas.

**Mensagens-chave**

- O uso de ARU é recomendado para melhorar a acurácia diagnóstica do ETT e do EcoE em pacientes com janelas acústicas limitadas, condição frequente na obesidade.^165,168^
- O SLG do VE é superior à FEVE na detecção de disfunção sistólica subclínica na obesidade e deve ser realizado sempre que a janela acústica permitir.^171^
- A indexação alométrica dos volumes do AE por altura é mais precisa e deve substituir a indexação por superfície corporal no paciente obeso.^173,174^
- O TAE, mensurável por ecocardiografia, está associado à rigidez miocárdica, DD e maior risco cardiovascular, podendo ser acompanhado.^175,176^
- A RMC tem boa qualidade, independentemente do grau de obesidade.
- Deficiência de perfusão e aumento de RT pela RMC são marcadores de mortalidade na obesidade.
- A avaliação da massa ventricular e função cardíaca é mais acurada pela RMC, tornando o método adequado para detectar alterações precoces e avaliar a melhora dos parâmetros após cirurgia bariátrica.

## 4. Aplicações da Multimodalidade em Imagem Cardíaca nas Doenças Reumatológicas

### 4.1. Lúpus Eritematoso Sistêmico

O lúpus eritematoso sistêmico (LES) é uma doença autoimune, inflamatória, crônica e multissistêmica, que frequentemente cursa com manifestações cardiovasculares, sendo essas importante causa de morbimortalidade em pacientes.

Existem quatro manifestações cardiovasculares principais no LES, em que o ecocardiograma desempenha um papel não só diagnóstico, mas também prognóstico: a doença pericárdica, a doença valvar, a HP e a miocardite.^1^

A doença pericárdica é o acometimento cardiovascular mais comum do LES, sendo, frequentemente, sua primeira manifestação, e estando presente em até 50% dos casos. A grande maioria se apresenta com um derrame pericárdico (DP) discreto e sem maiores repercussões. É importante avaliação criteriosa do quadro clínico, avaliando se estamos diante de uma polisserosite ou de um DP isolado. Na pericardite lúpica, frequentemente associada a um surto da doença, o paciente pode ter quadro clínico característico (tríade clássica: dor torácica, febre e atrito pericárdico), com alterações eletrocardiográficas e variados graus de DP. A pericardite complicada é rara e, em apenas 1 a 2% dos pacientes, o derrame é significativo resultando em tamponamento cardíaco. As pericardites constritiva ou infecciosa são de ocorrência rara.^187^

**Figura 13:**
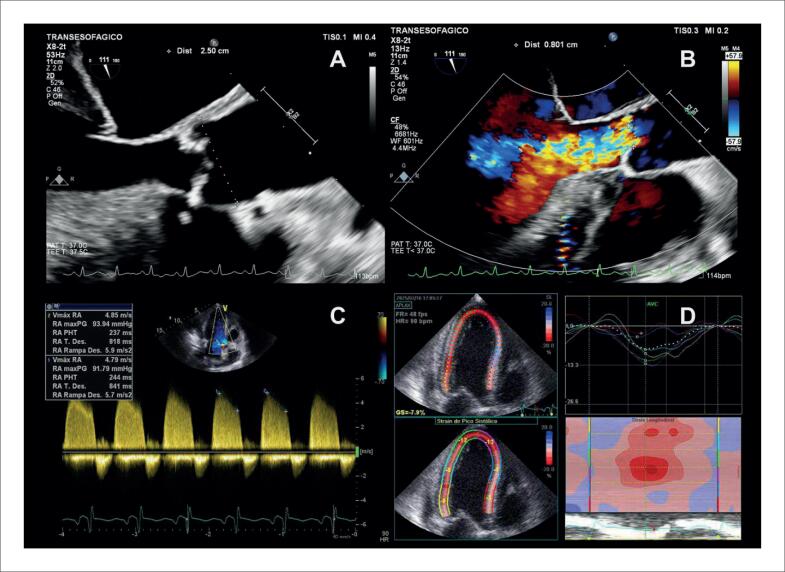
Imagens ecocardiográficas de paciente com diagnóstico de lúpus eritematoso sistêmico com valvulite aórtica e insuficiência aórtica de grau importante (A, B e C), além de miocardite evidenciada pela redução do SLG do ventrículo esquerdo ao corte três câmaras (D) (SLG = −7,9%) (D). Em A, ao ecocardiograma transesofágico, evidencia espessamento moderado e retração discreta da abertura da valva aórtica avaliada em eixo longo, associada a insuficiência de grau importante ao Doppler colorido (B). Em C, curva espectral ao Doppler contínuo com jato denso associado ao volume regurgitante significativo e redução do tempo de meia pressão (PHT = 237 ms). ETE: ecocardiograma transesofágico; PHT: tempo de meia-pressão (pressure half-time); SLG: strain longitudinal global.

O acometimento valvar no LES é muito frequente, chegando a até 75% em estudos de necropsia. As lesões normalmente encontradas são os espessamentos valvares inespecíficos e a endocardite de Libman-Sacks, com uma fisiopatologia peculiar. Trata-se de vegetações verrucosas estéreis (depósitos formados por imunocomplexos, fibrina e trombo plaquetário), mais comuns quando o LES vem acompanhado de anticorpos antifosfolípideo. O ETT auxilia o diagnóstico diferencial entre a endocardite de Libman-Sacks e a endocardite infecciosa, uma consideração importante nos pacientes imunossuprimidos. A valva mitral é a mais acometida na Libman-Sacks, com as lesões geralmente localizadas em sua face atrial e com menor mobilidade. Podem gerar distúrbios valvares (geralmente regurgitações) (Figura 12), fenômenos embólicos e serem substratos para a endocardite infecciosa.^188^ Na Figura 13, é ilustrado o caso de paciente com diagnóstico de LES com valvulite aórtica e insuficiência aórtica de grau importante, além de miocardite avaliadas pela ecocardiografia.

**Figura 14:**
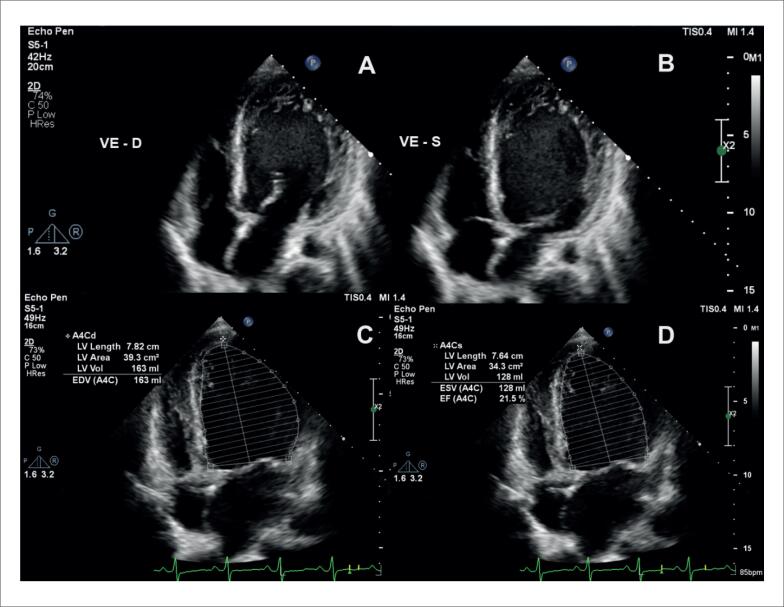
Imagens ecocardiográficas de paciente com diagnóstico de lúpus eritematoso sistêmico com disfunção ventricular importante evidenciada ao corte apical quatro câmaras pela pouca variação nos diâmetros diastólico (A) e sistólico (B) do ventrículo esquerdo, com sinais de contraste espontâneo na cavidade ventricular esquerda. Em (C) e (D), cálculo da fração de ejeção do ventrículo esquerdo (FEVE) pelo método de Simpson no corte apical quatro câmaras, evidenciando disfunção sistólica de grau importante (FEVE: 0,22).

A prevalência da HP nos pacientes com LES varia entre estudos, sendo estimada entre 0,5% e 17,5%. Tipicamente, são pacientes do sexo feminino em idade fértil, e são classificadas como Grupo I pelo consenso de HP.^189^ O ETT é fundamental na estimativa da pressão sistólica da artéria pulmonar (PSAP), calculada através da velocidade máxima da regurgitação tricúspide. Ele também avalia os sinais diretos e/ou indiretos sugestivos de HP – VD hipertrofiado e dilatado, átrio direito aumentado, VE deslocado para o seu interior (assumindo um formato em "D" e perdendo sua forma esférica), fluxo na via de saída do VD com padrão em "*notch*" (sugerindo uma alta resistência vascular pulmonar). É importante destacar dois aspectos: 1) não existe um padrão específico de HP no LES; 2) o ETT é um método que permite um diagnóstico de probabilidade, com o cateterismo direito permanecendo como padrão-ouro.^190^ A prevalência de miocardite no LES gira em torno de 10%, mesmo após grande evolução nos fármacos imunomoduladores.^191^ O ETT tem papel nesses casos, na detecção de anomalias segmentares e no grau de disfunção sistólica (Figura 14). Algumas vezes, a FEVE encontra-se preservada, porém, já com alterações no SLG, corroborando que, frequentemente, a disfunção é subclínica.^192^ Outras causas podem contribuir para a deterioração da função miocárdica, pois são pacientes que cursam com aterosclerose acelerada e arritmias.

A indicação da ecocardiografia nas doenças sistêmicas depende da prevalência de cardiopatia associada, das características peculiares ao comprometimento cardíaco em cada situação e da suspeita clínica de envolvimento cardíaco.^3^

A RMC tem se consolidado como a principal ferramenta não invasiva para a detecção precoce e caracterização tecidual do envolvimento miocárdico em pacientes com LES.^193^ Estudos demonstram que a fibrose miocárdica tem prevalência alta nesses pacientes, mesmo na ausência de sintomas clínicos, quando comparados a controles normais. Em uma das coortes mais amplas avaliadas por RMC com RT, observou-se uma frequência significativa de fibrose miocárdica, frequentemente localizada nas regiões inferolaterais e septais, associando-se com maior tempo de doença e presença de envolvimento multissistêmico.^194^ Adicionalmente, a localização e a extensão do RT foram associadas a pior função ventricular e pior prognóstico clínico, sugerindo seu potencial papel como marcador prognóstico.^195^

As técnicas quantitativas, como o mapeamento de T1 e T2, também têm contribuído para a avaliação da atividade inflamatória e do remodelamento tecidual. Estudos recentes revelaram que pacientes com LES apresentam valores de T1 e T2 elevados, particularmente naqueles não tratados com antimaláricos.^196^ O uso da RMC com *tissue tracking* permitiu, ainda, caracterizar disfunções biventriculares subclínicas em pacientes assintomáticos, contribuindo para a identificação de indivíduos com risco cardiovascular aumentado e potencial indicação de tratamento precoce.^197^

Recentemente, uma revisão sistemática com metanálise avaliou de forma abrangente o papel dos biomarcadores quantitativos da RMC em pacientes com LES, incluindo 18 estudos envolvendo um total de 955 pacientes. A análise revelou que indivíduos com LES apresentavam valores significativamente maiores de T1 nativo, VEC e T2, em comparação a controles saudáveis. Além disso, o SLG do VE estava significativamente reduzido nos pacientes com LES, sugerindo a presença de inflamação e fibrose miocárdica nesses pacientes, o que foi relacionado com o diagnóstico do acometimento cardiovascular e implicações prognósticas.^193^

Além do envolvimento miocárdico direto, a disfunção microvascular coronariana é uma preocupação em pacientes com LES, frequentemente associada à atividade inflamatória sistêmica. Um estudo de coorte com seguimento de 5 anos demonstrou que a disfunção microvascular coronariana avaliada por RMC com imagem de perfusão e a presença de placas ateroscleróticas (avaliadas por angiotomografia) estavam associadas ao desenvolvimento de eventos cardiovasculares futuros, mesmo em pacientes sem obstrução significativa das artérias coronárias. Esses achados reforçam a importância do rastreio precoce de isquemia miocárdica e da inflamação vascular em pacientes com LES.^198^

Além disso, a RMC tem emergido como a modalidade mais sensível para o diagnóstico de pericardite no contexto autoimune, permitindo a detecção de espessamento pericárdico, derrame e, sobretudo, inflamação ativa por meio de RT e aumento dos sinais em sequências ponderadas em T2.^194^ Em revisão recente sobre pericardite autoimune, a RMC foi destacada como essencial não apenas para o diagnóstico preciso, mas também para o monitoramento da resposta terapêutica e detecção de complicações, como pericardite constritiva transitória ou irreversível.^199^ Em pacientes com LES, a identificação precoce de envolvimento pericárdico ativo pela RMC pode impactar diretamente a conduta terapêutica, permitindo intervenções imunossupressoras mais precoces e potencialmente prevenindo a evolução para formas crônicas de fibrose pericárdica.

Portanto, a RMC oferece uma avaliação abrangente e sensível do envolvimento cardíaco em pacientes com LES, com potencial não apenas diagnóstico, mas também prognóstico e de monitoramento terapêutico. A incorporação sistemática da RMC no acompanhamento desses pacientes pode resultar na detecção precoce de complicações e melhor estratificação de risco.

A medicina nuclear, por sua vez, pode colaborar na avaliação desses pacientes no cenário das perimiocardites e na doença coronariana.^200^ O PET/CT com ^18^F-FDG tem sido empregado na avaliação da pericardite aguda, confirmando a presença do processo inflamatório, demonstrando a captação difusa do radiotraçador no pericárdio, geralmente em intensidade discreta a moderada. No entanto, a atividade do radiotraçador no pericárdio não é específica, não sendo capaz de diferenciar a etiologia dos diferentes processos inflamatórios ou infecciosos, nem mesmo dos casos de envolvimento neoplásico.^201,202^ Não existem estudos na literatura direcionados para o uso do PET/CT com ^18^F-FDG em pacientes com pericardite lúpica, portanto, seu uso pode ser indicado apenas em pacientes selecionados em que o método possa ser aplicado como avaliação complementar nas situações de dúvida diagnóstica.^202^

Da mesma forma, na miocardite, o ^18^F-FDG PET tem sido estudado em vários contextos clínicos.^203^ Com relação ao LES, sua utilização se limita a relatos de caso, em que o método foi capaz de auxiliar no diagnóstico e avaliar a resposta terapêutica.^204^ Infelizmente, trata-se de um procedimento de alto custo, envolve o uso de radiação e não está amplamente disponível.

Paciente com LES apresentam elevada prevalência de DAC e podem ter isquemia miocárdica demonstrada pela CPM, mesmo na ausência de obstrução significativa dos vasos epicárdicos e de sintomas, achados que estão associados a um incremento na ocorrência de eventos coronarianos.^205–207^ Assim, a CPM é um instrumento útil para identificar a presença de isquemia miocárdica e estimar o risco cardiovascular nos portadores de LES, que pode ser considerado como um equivalente de DAC.^2^

Estudos de perfusão miocárdica com PET/CT utilizando radiotraçadores como 13N-amônia e 15O-H20 demonstraram alteração da reserva de fluxo coronariano (RFC) em pacientes com LES, mesmo na ausência de sinais de inflamação sistêmica e de sintomas cardiovasculares, sinalizando a presença de disfunção microvascular persistente em até 57% dos casos.^208–210^ Infelizmente, esses radiofármacos não são produzidos no Brasil. Em nosso país, é possível a avaliação da RFC através da cintilografia miocárdica em câmaras com telureto de Cádmio e Zinco (Gama-Câmara CZT). No entanto, ainda não há estudos utilizando especificamente esse método no LES (Tabela 10).

**Tabela 10:** Recomendações para o uso de métodos em imagem cardíaca em pacientes com lúpus eritematoso sistêmico

Recomendação	Força da recomendação	Certeza da evidência
O ecocardiograma transtorácico deve ser realizada em pacientes com LES para avaliação de doença pericárdica, disfunções valvares, hipertensão pulmonar e miocardite.^1,187–190^	Fraca	Moderada
A ressonância magnética cardíaca com técnicas de cinerressonância, realce tardio, mapeamento de T1/T2 devem ser utilizadas para avaliação de acometimento cardiovascular em pacientes com LES.^188^	Fraca	Moderada
Diagnóstico de pericardite e miocardite com ^18^F-FDG.^191^	Fraca	Baixa
Detecção de isquemia miocárdica pela cintilografia de perfusão miocárdica.^3,192,193^	Forte	Moderada
Avaliação da reserva de fluxo coronariano.^195–197^	Fraca	Moderada

^18^F-FDG: fluordesoxiglicose-flúor-18; LES: lúpus eritematoso sistêmico.

Apesar da diversa gama de manifestações cardíacas em pacientes com LES, ainda não há uma rotina padrão para o rastreio em imagem cardíaca desses pacientes. Porém, o painel de especialistas deste documento recomenda a avaliação MM em imagem cardíaca para a detecção precoce de alterações cardiovasculares, estratificação de risco nesta população de pacientes que possui um muito alto risco cardiovasculares. A periodicidade dos exames deve ser individualizada baseada na avaliação clínica dos sintomas, atividade da doença, com ecocardiografia anual em pacientes de maior risco.^211–214^

**Mensagens-chave**

- As principais manifestações cardíacas no LES, em que o ETT desempenha um papel não só diagnóstico, mas também prognóstico são a doença pericárdica, a doença valvar, a HP e a miocardite.
- O acometimento valvar no LES é muito frequente, e as lesões mais encontradas são os espessamentos valvares inespecíficos.
- A prevalência da HP nos pacientes com LES varia entre estudos, estimada entre 0,5% e 17,5%. O ETT é fundamental na estimativa da PSAP, além de avaliar também os sinais diretos e/ou indiretos sugestivos de HP.
- O ETT é um método que permite um diagnóstico de probabilidade de HP, com o cateterismo direito permanecendo como padrão-ouro.
- Pacientes com LES podem ter acometimento miocárdico identificados pela RMC pela presença de fibrose miocárdica de padrão não isquêmico e elevação de mapa T1 nativo/VEC.
- A presença de inflamação e edema são adequadamente vistos pela técnica de mapeamento de T2.
- A pericardite é relativamente comum em pacientes com LES, sendo a RMC o método de escolha para avaliação de doença em atividade.

### 4.2. Esclerose Sistêmica

A esclerose sistêmica (ES) é uma doença autoimune do tecido conjuntivo rara, cuja patogênese é caracterizada por três marcadores principais: vasculopatia de pequenos vasos, produção de autoanticorpos e disfunção dos fibroblastos, levando ao aumento da deposição de matriz extracelular. O diagnóstico é fundamentalmente clínico, apoiado por testes sorológicos e exames de imagem.^215^ As principais características clínicas são: espessamento da pele, fenômeno de Raynaud e envolvimento de órgãos internos, conforme os critérios do *American College of Rheumatology/European League Against Rheumatism* (ACR/EULAR).^216^ O tipo e a gravidade do envolvimento dos órgãos determinarão o prognóstico heterogêneo da ES. Apesar da recente melhora na sobrevida desses pacientes, a ES continua sendo a doença reumatológica com a maior morbidade e mortalidade.^215^

O comprometimento cardiovascular na ES representa uma preocupação importante, sendo uma das principais causas de mortalidade entre esses pacientes.^217,218^ As complicações cardíacas podem ser tanto primárias quanto secundárias, incluindo arritmias,^219^ IC, doença pericárdica e fibrose miocárdica. A ecocardiografia é uma modalidade de imagem não invasiva, amplamente disponível, que desempenha um papel central na avaliação da estrutura e função cardíaca na esclerose sistêmica; ela é particularmente útil para detectar tanto o comprometimento cardíaco manifesto quanto o subclínico, já que, frequentemente, alterações cardíacas não são acompanhadas de sintomas nos estágios iniciais da doença. Como o comprometimento miocárdico na doença está relacionado a um pior prognóstico, é importante identificar alterações precoces para evitar a progressão do acometimento cardíaco.

**Figura 15:**
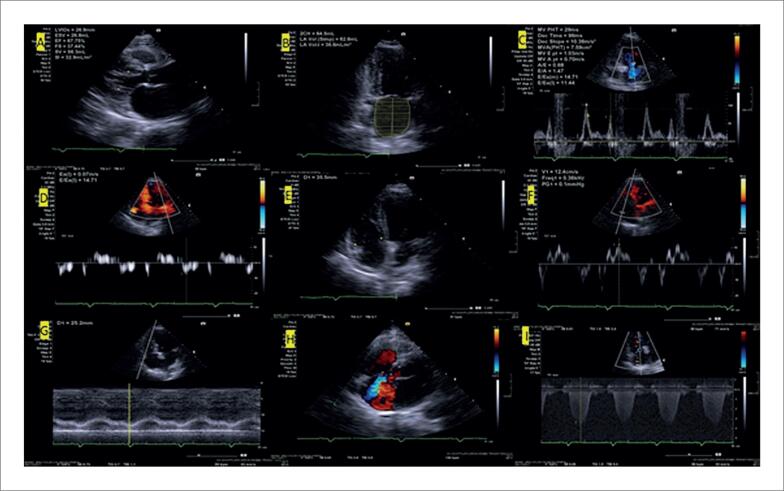
Imagens ecocardiográficas de paciente com diagnóstico de esclerose sistêmica e pneumonia intersticial. As imagens mostram função sistólica normal (A) e aumento do volume do átrio esquerdo associado à disfunção diastólica moderada (B, C e D). A avaliação do ventrículo direito mostra dimensões e função sistólica preservadas pela excursão sistólica do plano do anel tricúspide e s’ (E, F e G), porém, aumento da pressão sistólica pulmonar, avaliada pelo refluxo tricúspide, com velocidade de fluxo de 3,34 m/s (H e I).

O ETT em pacientes com ES possibilita a avaliação do envolvimento cardíaco, detectando a disfunção sistólica e diastólica, anormalidades valvares e HP^220^ (Figura 15). A ecocardiografia com avaliação de deformação miocárdica, realizada através do *strain* por *speckle tracking* é especialmente útil para identificar a disfunção miocárdica subclínica em pacientes com ES, a qual é frequentemente imperceptível através da ecocardiografia convencional. Pacientes com ES apresentam parâmetros de deformação miocárdica significativamente mais baixos quando comparados aos controles saudáveis, indicando disfunção miocárdica envolvendo tanto os ventrículos quanto os átrios.^221–226^ Mesmo quando a FEVE está preservada, os SLGs do VE e VD podem estar reduzidos,^225^ sugerindo que essa modalidade seja mais sensível do que o ecocardiograma tradicional na identificação do envolvimento cardíaco inicial.

Adicionalmente, a ecocardiografia é uma modalidade não invasiva valiosa para a avaliação inicial da pressão arterial pulmonar, especialmente considerando-se que a presença de HP está relacionada a maior mortalidade em pacientes com ES.^227^ As medidas de pressão arterial pulmonar pelo ecocardiograma podem refletir a presença de doença pulmonar intersticial, o acometimento cardíaco esquerdo com aumento da pressão capilar pulmonar e também a vasculopatia pulmonar com HAP associada.^227^ No entanto, apesar de ser útil para o rastreamento de HP, a acurácia diagnóstica da ecocardiografia é considerada moderada, com sensibilidade e especificidade em torno de 83% e 72%, respectivamente.^228^ Devido a suas limitações, o ecocardiograma não deve ser utilizado como única medida de estimativa da pressão pulmonar, necessitando da combinação com outras avaliações: o cateterismo direito é considerado o exame mais fidedigno para o diagnóstico e monitoramento da HP.^228,229^

A avaliação do VD é crucial para o acompanhamento de pacientes com ES e HP, pois a presença de disfunção do VD está associada à maior morbidade e mortalidade. Medidas ecocardiográficas, como a TAPSE, a variação fracional da área (FAC, do inglês *fractional area change*) e a velocidade sistólica do Doppler tecidual (S’), são utilizadas habitualmente para avaliar a função sistólica do VD (Figura 15), juntamente com o ecocardiograma tridimensional e o *strain* do VD.^190^ De maneira complementar, a adaptação inadequada do VD ao aumento da pós-carga arterial pulmonar é um fator determinante de desfechos adversos na ES; a medida de acoplamento VD-TP (artéria pulmonar), avaliada através de índices não invasivos como TAPSE/PSAP e FAC/PSAP, apresenta valor prognóstico na identificação de desfechos clínicos desfavoráveis.^230^ É importante destacar que os parâmetros ecocardiográficos utilizados para avaliação do VD apresentam limitações devido à complexidade anatômica dessa câmara e à influência de condições de carga. Dessa forma, esses parâmetros não devem ser utilizados isoladamente, mas, sim, em conjunto com outros indicadores, para uma avaliação mais precisa.^190^

O ecocardiograma também pode detectar o movimento assincrônico do septo, relacionado com o comprometimento miocárdico observado na RMC. Tal achado na ecocardiografia antecipa resultados anormais na RMC, como aumento do VEC e fibrose miocárdica, indicando envolvimento cardíaco na ES.^231^

Além do ETT, a ecocardiografia sob estresse físico pode ser utilizada na ES, permitindo a detecção de aumento na PSAP^229,232^ e/ou na pressão de enchimento do VE^233^ durante o exercício, indicando ICFEp e associando-se a desfechos clínicos desfavoráveis.^232^ A presença de HAP não deve se basear apenas na elevação da PSAP durante o exercício, uma vez que a presença de DD, avaliada pela E/e’, é frequentemente observada nesses pacientes.^234^ Durante a ecocardiografia de estresse em pacientes com ES, é crucial monitorar a PSAP, a função ventricular direita e esquerda e sinais de congestão pulmonar; complicações, embora raras, devem ser cuidadosamente monitorizadas.

Finalmente, o acompanhamento individualizado da ES, incluindo as complicações cardíacas, deve se basear nas características do paciente e gravidade da doença, considerando a extensão do envolvimento dos órgãos. Como o tratamento pode incluir terapias imunossupressoras, vasodilatadores e medicamentos específicos para complicações cardíacas (antiarrítmicos ou protocolos para IC), além de antagonistas do receptor de endotelina ou inibidores da fosfodiesterase-5 para manuseio da HP,^235,236^ o acompanhamento regular com o ETT é essencial para monitorizar os efeitos colaterais dos tratamentos específicos, avaliar a função cardíaca e otimizar os resultados clínicos.^235^

Em pacientes com ES, a RMC permite a caracterização tecidual detalhada, o que possibilita a detecção precoce de inflamação e fibrose miocárdica, que podem estar presentes mesmo sem manifestações clínicas.

O RT de padrão não isquêmico é um achado comum da RMC de pacientes com ES e indica a presença de fibrose miocárdica, mesmo na ausência de sintomas ou disfunção ventricular evidente. A presença de RT está associada a pior prognóstico cardiovascular (arritmia e morte súbita). No estudo multicêntrico SAnCtUS (*Scleroderma Arrhythmia Clinical Utility Study*), a presença de RT foi preditora independente de arritmias ventriculares, reforçando seu valor prognóstico nessa população.^237^ Técnicas avançadas, como a análise de entropia do RT com gadolínio, também demonstraram valor prognóstico adicional, ao quantificar a heterogeneidade da fibrose e associar-se com piores desfechos clínicos.^238^

Além da técnica de RT, a RMC permite a avaliação da fibrose difusa por meio dos mapas de T1 nativo e do VEC. Esses parâmetros se mostraram aumentados em pacientes com ES, mesmo em fases iniciais e na ausência de alterações convencionais, indicando acometimento miocárdico subclínico.^239^ O mapa de T2, por sua vez, tem se destacado na avaliação da atividade inflamatória, sendo particularmente útil na diferenciação entre inflamação ativa e fibrose crônica. Em um estudo recente, o mapeamento de T2 demonstrou maior sensibilidade que a análise visual na detecção de inflamação miocárdica associada à ES, mesmo em pacientes assintomáticos.^240^

Outro estudo que comparou pacientes com ES a indivíduos saudáveis, os valores globais de T1 e T2 miocárdicos foram significativamente mais elevados nos pacientes com ES. Notadamente, 62% dos pacientes com RMC convencional normal (ausência de RT e de edema visual) apresentaram elevação no T1 nativo e/ou T2. Além disso, 40% dos pacientes com T1 aumentado exibiram maiores volumes diastólicos do VE, maiores volumes sistólicos biventriculares, elevação do T2 global e histórico mais frequente de úlceras digitais.^240^

Outro aspecto importante é o papel emergente da RMC na avaliação da resposta terapêutica. Em estudo prospectivo, pacientes com miocardite associada à ES foram reavaliados após tratamento imunossupressor, observando-se redução nos sinais de inflamação miocárdica nos mapas de T2 e RT, demonstrando o potencial da RMC como ferramenta de monitoramento de resposta ao tratamento.^242^

Uma revisão sistemática recente avaliou o papel prognóstico da RMC em pacientes com ES. Os principais parâmetros da RMC identificados como preditores consistentes de mortalidade por todas as causas foram o RT, o valor de T1 nativo, o VEC e os índices de deformação ventricular (*strain*). Embora menos investigados, outros marcadores – como *strain* atrial esquerdo, medidas de perfusão e parâmetros obtidos por RMC sob estresse – também demonstraram valor prognóstico, mas ainda carecem de validação robusta.^243^

As evidências da utilização da medicina nuclear na avaliação cardiovascular de pacientes com diagnóstico de ES são limitadas a pequenos estudos clínicos, mas com resultados interessantes. Há mais de quatro décadas, a CPM, primeiramente, com cloreto de 201Tálio e, posteriormente, com 99mTc-sestamibi, tem demonstrado que até cerca de 80% dos pacientes com ES podem apresentar defeitos perfusionais, mesmo na ausência de doença aterosclerótica coronariana. Existe associação entre a presença de fenômeno de Raynaud e isquemia miocárdica deflagrada por estresse farmacológico com dipiridamol ou exposição ao frio em aproximadamente 50% dos casos. Esses achados indicam disfunção microvascular e vasoespasmo como um importante substrato fisiopatológico para o dano miocárdico nesse contexto.^244–248^

A avaliação de RFC com PET/CT usando 82Rubídio demonstrou que a ES é um preditor independente de redução da reserva de fluxo, que ocorreu em 89% dos casos, sendo marcador de disfunção microvascular nesses pacientes.^249^

Também tem sido observada a presença de DD através do *gated* SPECT da CPM com 99mTc-sestamibi, com prolongamento do tempo para o pico de enchimento do VE naqueles com elevado escore de espessura total da pele em comparação com grupo-controle ou com os de baixo escore. Essa alteração foi identificada mesmo quando não havia defeitos de perfusão. Os autores ressaltam que esse resultado pode se correlacionar com a gravidade da ES e ser um sinalizador inicial de acometimento miocárdico. Em um dos estudos, também encontraram alterações da função simpática cardíaca avaliada pela cintilografia com 123I-MIBG, corroborando a hipótese de que disfunção autonômica é algo comum nesse grupo de indivíduos.^250,251^

Por fim, PET/CT com ^18^F-FDG evidenciou a captação anômala miocárdica do radiotraçador em metade dos pacientes com ES e sem sintomas cardiovasculares, como indício de miocardite subclínica, que pode apontar precocemente o envolvimento cardíaco da doença.^252^

Estudos mais robustos são necessários para fortalecer esses achados e compreender seu impacto prognóstico. No entanto, a realização de CPM parece razoável nos pacientes com ES, sobretudo nos sintomáticos, para a identificação de isquemia miocárdica (Tabela 11).

**Tabela 11:** Recomendações para o uso multimodalidade em imagem cardíaca em pacientes com esclerose sistêmica

Recomendação	Força da recomendação	Certeza da evidência
ETT com DT para avaliação de função ventricular esquerda sistólica e diastólica.^217,220,221^	Forte	Moderada
ETT com análise do SLG para avaliação precoce de disfunção ventricular esquerda.^223,225,226^	Fraca	Moderada
Avaliação diagnóstica de hipertensão pulmonar pelo ETT pela estimativa da velocidade do jato de regurgitação tricúspide.^227,228^	Forte	Alta
Ecocardiograma sob estresse com exercício para avaliação da pressão pulmonar em pacientes com dispneia sem hipertensão pulmonar ao ETT.^232,233^	Fraca	Alta
ETT com medidas de TAPSE, S' e SLG do VD, FAC do VD para acompanhamento da função sistólica do VD.^190,230^	Forte	Alta
ETT com DT para avaliação de resposta terapêutica.	Fraca	Baixa
RMC com o emprego de técnicas de RT, mapeamento de T1 e T2 para o diagnóstico de acometimento cardíaco e estratificação prognóstica em pacientes com ES.^221^	Fraca	Moderada
Cintilografia de perfusão miocárdica para detecção de isquemia miocárdica.^244–249^	Forte	Alta
PET/CT com ^18^F-FDG para avaliação de inflamação miocárdica.^252^	Fraca	Baixa
Cintilografia cardíaca com 123I-MIBG para avaliação da função simpática.^251^	Fraca	Baixa

123I-MIBG: iodo-123-metaiodobenzilguanidina; DAC: doença arterial coronariana; DT: Doppler tecidual; ETT: ecocardiograma transtorácico; ES: esclerose sistêmica; FAC: variação fracional da área (do inglês fractional area change); PET/CT com ^18^F-FDG: tomografia por emissão de pósitrons associada a tomografia computadorizada com fluordesoxiglicose-flúor-18 (do inglês Fluorodeoxyglucose Positron Emission Tomography/Computed Tomography); TAPSE: excursão sistólica do plano do anel tricúspide (do inglês tricuspid annular plane systolic excursion); RMC: ressonância magnética cardíaca; RT: realce tardio; SLG: strain longitudinal global; VD: ventrículo direto.

É recomendada avaliação ecocardiográfica anual (com análise do *strain* miocárdico se disponível), ressonância magnética cardíaca conforme indicação clínica a cada 2-3 anos em pacientes de alto risco ou com sintomas, e cintilografia miocárdica apenas em casos selecionados.^253–255^

**Mensagens-chave**

- O comprometimento cardiovascular é comum e uma das principais causas de mortalidade na ES.
- O ETT é fundamental para avaliar função cardíaca e rastrear HP.
- O *strain* por *speckle tracking* detecta disfunção miocárdica precoce, mesmo com fração de ejeção preservada.
- A avaliação do VD em pacientes com ES e HP é essencial para o manejo do paciente e a estratificação de risco.
- O ETT é importante para o rastreamento da HP, mas o cateterismo direito é o padrão-ouro para diagnóstico.
- A ecocardiografia sob estresse físico pode identificar DD e ICFEp, além de HP.
- O acompanhamento regular com ecocardiografia é importante para guiar o tratamento e monitorar complicações.
- A RMC pode ser utilizada para a detecção de acometimento cardíaco em pacientes com ES em atividade.
- A RMC com avaliação morfofuncional e caracterização tecidual é uma ferramenta valiosa na avaliação diagnóstica e prognóstica da doença.

### 4.3. Espondilite Anquilosante

A espondilite anquilosante (EA) é uma espondiloartropatia soronegativa, inflamatória, autoimune, crônica e progressiva associada ao antígeno HLA B27. Caracteriza-se por acometimento das articulações sacroilíacas e da coluna vertebral. A doença também se associa a entesite, uveíte, doença inflamatória intestinal e psoríase.^256–258^

Pacientes com EA apresentam maior risco de doenças cardiovasculares do que a população geral, incluindo insuficiência das valvas aórtica e mitral, doenças da aorta, IC, acometimento do sistema de condução e DAC.^257,259^

O acometimento miocárdico e valvar é explicado pelo processo inflamatório e fibrótico desencadeado pela atividade da doença.^257,259^ Especificamente na valva aórtica, foi demonstrada ativação de fibroblastos e agregação plaquetária, resultando na fibrose valvar.^258^

A Tabela 12 demonstra a fisiopatologia do acometimento cardíaco e aórtico e sua prevalência em indivíduos com a doença.

**Tabela 12:** Acometimento cardíaco e aórtico na espondilite anquilosante^257,260–262^

Estrutura	Fisiopatologia	Prevalência
Raiz aórtica e aorta ascendente proximal	Inflamação (aortite), resultando em rigidez e dilatação.	20-60%
Valva aórtica	Inflamação e fibrose, com espessamento e retração das válvulas	40%
Valva mitral	Inflamação e fibrose, com espessamento da junção mitro-aórtica, resultando em redução da mobilidade ou coaptação incompleta ou assimétrica das cúspides.	50%
Miocárdio	Inflamação e fibrose, comprometendo o enchimento diastólico do ventrículo esquerdo.	ND
Sistema de condução	Extensão da fibrose subaórtica, com acometimento do nó atrioventricular, feixe de His proximal e ramos e fascículos, resultando em distúrbios de condução.	ND

ND: dados não disponíveis.

A ecocardiografia é a modalidade de imagem de escolha na avaliação inicial e no seguimento em longo prazo das doenças valvares. Também é a ferramenta ideal para avaliação da função diastólica.^3^

A ecocardiografia, pela avaliação do SLG do VE, pode ainda mostrar disfunção sistólica subclínica, revelando acometimento miocárdico, que pode estar presente desde as fases iniciais da EA.^259^

A RMC, além da avaliação da função ventricular e das valvas cardíacas, permite a caracterização do tecido miocárdico, demonstrando a presença de edema e/ou fibrose. Na EA, pode-se observar edema miocárdico, o qual está associado à atividade da doença, e RT focal, refletindo fibrose miocárdica.^257^ A RMC identifica a disfunção global do VE e áreas focais de RT como marcadores definitivos de envolvimento cardíaco. O VEC, quantificado por meio de mapeamento T1 miocárdico, apresenta associação com o grau de atividade da doença, destacando o potencial do VEC como biomarcador para monitoramento da atividade da doença.^257^ Tanto a RMC quanto a angioTC podem avaliar a aorta para detectar dilatação, estenoses e sinais inflamatórios.

Embora a EA esteja associada a aterosclerose prematura e aumento do risco de eventos cardiovasculares, são escassas as publicações sobre o papel da medicina nuclear nesse cenário.

Em estudo que incluiu 24 pacientes com diagnóstico de EA, os autores concluem que o PET/CT com ^18^F-FDG foi capaz de demonstrar inflamação da parede vascular em pacientes jovens com EA, além da redução da atividade inflamatória sob efeito de estatina.^256^ Outra publicação utilizou a CPM com 99mTc-sestamibi em 28 pacientes com EA, e observou-se alteração da motilidade segmentar pelo *gated* SPECT em oito pacientes, podendo inferir disfunção microvascular.^257^

Diante da escassez de evidências, não podem ser afirmadas recomendações sobre o uso de métodos de medicina nuclear especificamente no contexto da EA. Por outro lado, essa doença inflamatória compartilha fatores fisiopatológicos com outras doenças sistêmicas, como a artrite reumatoide (AR) e o LES, em que a utilização da CPM e o PET/CT com ^18^F-FDG já apresentam evidências e indicações mais consolidadas (Tabela 13).

**Tabela 13:** Recomendações para o uso da multimodalidade de imagem na avaliação da espondilite anquilosante

Recomendação	Força da recomendação	Certeza da evidência
Ecocardiografia para avaliação de acometimento da raiz aórtica e aorta ascendente proximal.^258–260^	Forte	Baixa
Ecocardiografia para avaliação das valvas mitral e aórtica.^258–260^	Forte	Baixa
Ecocardiografia para avaliação da função diastólica.^258–265^	Fraca	Alta
Ecocardiografia com avaliação do *strain* longitudinal global do ventrículo esquerdo para detecção de disfunção subclínica.^258–265^	Fraca	Moderada
RMC para avaliação de acometimento da raiz aórtica e aorta ascendente.^257^	Fraca	Baixa
RMC para avaliação das valvas mitral e aórtica em pacientes com limitação de imagem ao ecocardiograma e/ou definição de momento da intervenção valvar.^257^	Fraca	Baixa
RMC para avaliação da função ventricular esquerda em pacientes com limitação de imagem ao ecocardiograma.^257^	Fraca	Baixa
RMC para caracterização tecidual do acometimento miocárdico com a medida do volume extracelular miocárdico como marcador de atividade.^257^	Fraca	Baixa
Uso de PET/CT com ^18^F-FDG para avaliação de inflamação vascular.^263^	Fraca	Baixa
Detecção de isquemia miocárdica pela cintilografia de perfusão miocárdica.^264^	Fraca	Baixa

RMC: Ressonância magnética cardíaca; PET/CT com ^18^F-FDG: tomografia por emissão de pósitrons associada a tomografia computadorizada com fluordesoxiglicose-flúor-18 (do inglês *Fluorodeoxyglucose Positron Emission Tomography/Computed Tomography*).

Atualmente, é recomendada avaliação ecocardiográfica anual (com análise do *strain* miocárdico se disponível), ressonância magnética cardíaca conforme indicação clínica a cada 2-3 anos em pacientes de alto risco ou com sintomas, e cintilografia miocárdica apenas em casos selecionados. A realização de exames de imagem cardíaca em pacientes assintomáticos e estáveis não sejam realizados em intervalos menores que um ano, exceto diante de achados clínicos.^1,257,266–269^

**Mensagens-chave**

- Pacientes com EA apresentam maior risco de doenças cardiovasculares que incluem insuficiência das valvas aórtica e mitral, doenças da aorta, IC, acometimento do sistema de condução e DAC.
- O ETT é a método de escolha na avaliação inicial e no seguimento em longo prazo das doenças valvares e da função diastólica.
- A análise do SLG do VE pode evidenciar disfunção sistólica subclínica, que pode estar presente desde as fases iniciais da EA.
- A RMC, além da avaliação da função ventricular e das valvas cardíacas, permite a caracterização do tecido miocárdico, demonstrando a presença de edema e/ou fibrose.
- O VEC miocárdico apresenta associação com o grau de atividade da doença, destacando o potencial do VEC como biomarcador para o monitoramento da atividade da doença.
- A RMC e a TC são exames de escolha para avaliação da aorta torácica.

### 4.4. Artrite Reumatoide

A AR é uma doença autoimune, inflamatória, crônica, com períodos de remissão e surtos (exacerbações), que afeta principalmente as articulações de mãos, punhos, pés e joelhos. O sistema imunológico, por razões ainda não totalmente compreendidas, passa a acometer os tecidos do próprio corpo – especialmente a membrana sinovial que reveste as articulações, causando dor, inchaço, rigidez e, com o tempo, deformidades e destruição articular. A doença pode, ainda, afetar outros órgãos e sistemas como pulmões (nódulos reumatoides e fibrose pulmonar), olhos (esclerite e ceratoconjuntivites) e pele e vasos (vasculite).^265^ No coração, as principais manifestações da AR são pericardite, miocardite e lesão endotelial levando ao aumento do risco cardiovascular.^270^

O diagnóstico é clínico, baseado nos sinais e sintomas, apoiado por exames laboratoriais e de imagem, incluindo o fator reumatoide (FR), dosagem de autoanticorpos anti-CCP (ACPA), velocidade de hemossedimentação (VHS), proteína C reativa (PCR), radiografia e ultrassonografia para dano articular. Embora as manifestações articulares sejam o principal foco clínico, complicações cardiovasculares são uma das principais causas de morbidade e mortalidade nesses pacientes, muitas vezes de forma subclínica.^271^ Assim, a ecocardiografia é uma ferramenta diagnóstica fundamental na avaliação de pacientes com AR.

O ETT permite uma avaliação não invasiva, segura e eficaz das estruturas cardíacas e é indicado em pacientes com AR para detectar alterações precoces, como DD, espessamento e derrames pericárdicos, valvopatias (especialmente insuficiência aórtica e mitral) e alterações na contratilidade ventricular. A ecocardiografia com Doppler tecidual também contribui para avaliar a função miocárdica de forma mais sensível, mesmo na ausência de sintomas clínicos evidentes.^272^

**Figura 16:**
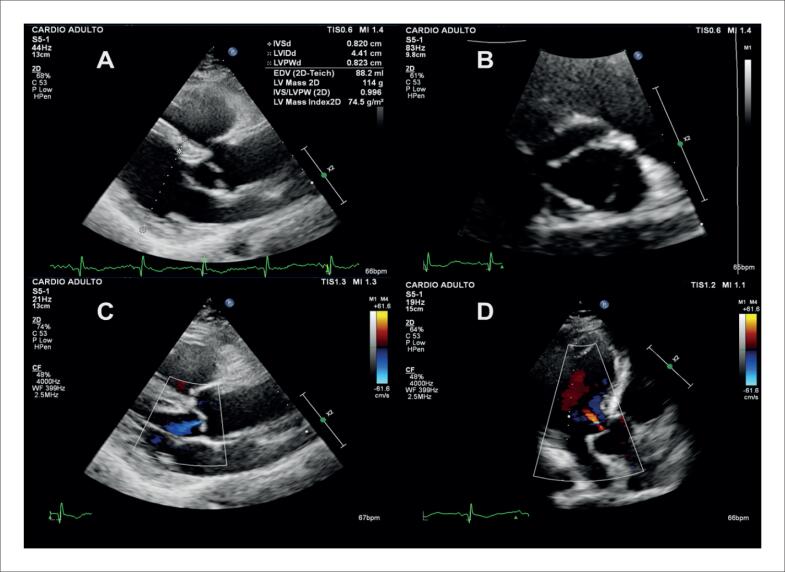
Paciente com diagnóstico de artrite reumatoide apresenta, ao ecocardiograma transtorácico, espessamento discreto valvar aórtico ao corte paraesternal longitudinal (A) e transverso (B) com refluxo de grau discreto ao Doppler colorido (C) e (D).

Além disso, o ETE pode ser indicado em casos mais específicos, quando há suspeita de endocardite ou acometimento valvar mais detalhado, particularmente em pacientes com sopros cardíacos novos ou embolias inexplicadas. A avaliação regular por ecocardiografia pode, portanto, auxiliar no diagnóstico precoce de complicações cardíacas, influenciar o manejo terapêutico e melhorar o prognóstico (Figura 16).^273^

Na AR, vários parâmetros ecocardiográficos têm sido identificados como prognósticos, ou seja, associados a maior risco de eventos cardiovasculares, pior evolução clínica ou mortalidade. Esses parâmetros auxiliam o diagnóstico do envolvimento cardíaco subclínico, permitindo intervenções mais precoces e eficazes.^270^

A redução da FEVE (< 50%) está associada a pior prognóstico, mas é um achado tardio na maioria dos casos. A DD de grau II ou superior está ligada a risco aumentado de IC e eventos cardiovasculares, sendo prevalente mesmo em fases precoces da AR, especialmente em mulheres.

Pacientes com AR frequentemente apresentam redução do SLG mesmo com FEVE preservada, o que indica dano miocárdico precoce, possivelmente relacionado à inflamação crônica, fibrose ou microangiopatia.^274^ Estudos mostram correlação entre níveis de marcadores inflamatórios (como PCR e fator de necrose tumoral alfa) e alterações no *strain*.^275^ Isso sugere que o SLG pode refletir o impacto cardiovascular da atividade inflamatória da AR, sendo útil tanto na avaliação basal quanto no acompanhamento longitudinal da doença. O *strain* reduzido em pacientes assintomáticos com AR pode indicar maior risco de eventos cardiovasculares, auxiliando na estratificação de risco e na decisão por intervenções precoces, como intensificação do controle da doença ou uso de cardioprotetores. Além do VE, o *strain* pode ser utilizado para avaliar a função do VD e dos átrios, que também podem ser afetados pela AR. Isso é particularmente útil em casos com suspeita de HP ou envolvimento pericárdico crônico.^276^

Uma metanálise demonstrou que o aumento da massa miocárdica estão associados a disfunção miocárdica progressiva e maior risco de IC. Na AR, isso pode ocorrer mesmo sem hipertensão arterial, refletindo inflamação crônica.^277^

Outra metanálise avaliou estudos caso-controle sobre o envolvimento valvar e pericárdico na AR. Espessamento ou derrame pericárdico são comuns em pacientes com AR de longa data, indicando pericardite crônica ou aguda, mesmo assintomática. Achados persistentes estão associados a maior atividade da doença e risco de complicações, como tamponamento ou pericardite constritiva.^278^

A avaliação da PSAP pela velocidade de regurgitação tricúspide (> 35-40 mm Hg) também tem valor prognóstico e está associada a aumento da mortalidade na AR, podendo refletir doença pulmonar avançada.^272^ Além disso, a presença de disfunção do VD, seja avaliada pelos métodos tradicionais (TAPSE, s’, FAC) ou pelo *strain* do VD, é preditiva de pior evolução, especialmente na presença de HP. Um estudo recente demonstrou que o acoplamento ventrículo-arterial pulmonar obtido por *speckle tracking* pode ser utilizado como um marcador de lesão miocárdica precoce na AR.^279^

A ecocardiografia tridimensional (3D) representa um avanço tecnológico significativo na avaliação cardíaca, oferecendo imagens em tempo real com maior acurácia e detalhamento anatômico do que a ecocardiografia bidimensional (2D). Na AR, essa modalidade pode trazer benefícios específicos, tanto na avaliação morfológica quanto funcional do coração, especialmente diante das manifestações cardiovasculares subclínicas comuns nessa população. A eco 3D fornece imagens realistas das valvas, facilitando a avaliação de espessamento, retração, calcificação e alterações funcionais.^280^

Ainda, diversos estudos observacionais corroboram o aumento do risco cardiovascular na AR. Uma metanálise recente validou o uso da espessura médio-intimal da carótida como marcador de risco cardiovascular elevado em pacientes com AR.^281^ A espessura médio-intimal carotídea encontra-se significativamente aumentada em indivíduos com AR em relação aos controles saudáveis, reforçando o papel da ultrassonografia vascular (USV), um método amplamente disponível, de baixo custo e que permite a detecção precoce de placas e espessamento da camada médio-intimal.^2,211,212,283,283^ Assim, recomenda-se a utilização da USV para o rastreamento de aterosclerose subclínica carotídea e femoral.

Embora a ecocardiografia continue sendo o exame de escolha inicial, a tomografia computadorizada cardíaca (TCC) e a RMC ampliam a avaliação do comprometimento cardiovascular na AR, especialmente quando há necessidade de maior precisão anatômica, caracterização tecidual ou quantificação objetiva de alterações, como na doença cardiovascular subclínica, suspeita de miocardite ou fibrose, avaliação de risco aterosclerótico precoce e nos casos em que o ecocardiograma se mostra inconclusivo. Esses exames são particularmente valiosos em centros especializados e podem orientar decisões terapêuticas mais precisas.^284^

Diversos estudos demonstraram a utilidade da RMC na caracterização precoce de alterações miocárdicas, mesmo em pacientes recém-diagnosticados com AR e sem histórico de doença cardiovascular estabelecida. Um estudo recente evidenciou elevação nos valores de T1 e VEC em pacientes com AR ativa e presença de autoanticorpos, sugerindo inflamação e remodelamento miocárdico precoce.^285^ Valores elevados de mapeamento de T2 sugerindo edema miocárdico também são uma alteração encontrada em pacientes com AR associada à atividade da doença.^286^ Além disso, há evidências de que o tratamento imunobiológico pode impactar positivamente os parâmetros funcionais do VE avaliados pela RMC, reduzindo a massa miocárdica e melhorando a FEVE.^287–289^

Sabe-se que pacientes com AR apresentam risco cardiovascular aumentado, em parte, pela inflamação crônica sistêmica, além de fatores de risco tradicionais. A TCC, incluindo a avaliação do escore de cálcio coronariano e angiografia por tomografia (angio-TC), tem se mostrado uma ferramenta importante na estratificação precoce de risco e monitoramento da progressão da aterosclerose subclínica; estudo recente demonstrou que pacientes com AR possuem escore de cálcio coronariano significativamente maior que controles saudáveis, destacando a TCC na avaliação precoce da aterosclerose subclínica.^290^ Além disso, a TCC pode detectar também calcificações valvares e arteriais com maior frequência quando comparada a indivíduos sem doenças autoimunes.^291^

A progressão da aterosclerose coronariana em pacientes com AR também parece estar associada ao grau de inflamação cumulativa, fatores de risco cardiovascular e ao tipo de exposição medicamentosa. Um estudo prospectivo utilizando angio-TC mostrou que pacientes com maior atividade inflamatória e menor controle clínico apresentaram maior progressão de placas ateroscleróticas, mesmo após ajuste para fatores de risco tradicionais.^292^

Por outro lado, estratégias terapêuticas voltadas para o controle inflamatório rigoroso têm se mostrado eficazes na redução do risco cardiovascular. Ensaios clínicos randomizados comparando imunomoduladores demonstraram que, além do controle da atividade articular, houve impacto positivo em desfechos vasculares, como redução da inflamação arterial e controle adequado do perfil lipídico.^293,294^ Tais efeitos podem ser monitorados com métodos de imagem não invasivos, como a TCC, e técnicas associadas à detecção de inflamação vascular como o PET/FDG.

Assim, esses achados ressaltam a relevância da RMC e da TCC como ferramentas diagnósticas e prognósticas em pacientes com AR, oferecendo não apenas uma avaliação morfofuncional detalhada, mas também o potencial de monitorar a resposta terapêutica. A incorporação rotineira da RMC e TCC em estratégias de rastreamento cardiovascular na AR pode permitir intervenções mais precoces e individualizadas.^295^

A medicina nuclear pode contribuir em pacientes com AR na investigação de pericardite e miocardite, demonstrando a atividade inflamatória da doença no coração através do PET/CT com ^18^F-FDG. O PET/CT e PET/MR com ^18^F-FDG também têm sido utilizados para avaliar a inflamação de vasos de grande e médio calibre e sua relação com o risco cardiovascular. Outra aplicação é na avaliação de isquemia miocárdica pela CPM ou pelo PET/CT utilizando radiotraçadores de perfusão.^2^

Um estudo interessante demonstrou captação considerada patológica de ^18^F-FDG no miocárdio em 39% de indivíduos com AR. Esse achado se correlacionou com critérios clínicos e laboratoriais de atividade da doença, sugerindo que o método de imagem possa ser considerado um marcador de inflamação miocárdica subclínica. O exame foi repetido 6 meses após o tratamento e evidenciou redução significativa da captação nos que utilizaram inibidor de fator de necrose tumoral, sinalizando resposta adequada ao tratamento.^296^ É importante mencionar que a visualização anômala de ^18^F-FDG no miocárdio não é característica de nenhum processo específico, apenas identifica a presença de inflamação ativa. O diagnóstico definitivo dependerá da integração entre as informações clínicas, laboratoriais e de outros métodos de imagem, além da eventual realização de biópsia endomiocárdica em casos selecionados, dependendo do contexto.

Publicações têm demonstrado que pacientes com AR em atividade apresentam aumento de captação de ^18^F-FDG na carótida e na aorta, como sinal de inflamação vascular. Também há evidências de que o tratamento da AR pode reduzir a intensidade da captação.^297,298^ No entanto, também já foi demonstrado que pacientes com AR de longa data podem permanecer com sinais de aumento da atividade inflamatória vascular mesmo no cenário de remissão da doença.^299,300^

A evidência mais significativa quanto ao impacto da AR no cenário da doença coronariana evidenciou redução da RFC pelo PET/CT com 82Rubídio ou 13N-amônia nos pacientes com AR de forma semelhante aos diabéticos, sem diagnóstico prévio de DAC. No seguimento de até 10 anos, a taxa de mortalidade foi maior naqueles com a RFC alterada. Os resultados corroboram que a AR é um determinante de disfunção microvascular assim como o diabetes, associando-se a maior risco de morte cardiovascular e de todas as causas.^301^ Porém, esses radiofármacos não são produzidos no Brasil. Em nosso país, é possível fazer a avaliação da RFC através da cintilografia miocárdica. No entanto, ainda não há estudos utilizando especificamente esse método na AR.

A avaliação de risco coronariano pode ser recomendada para todos os pacientes com AR a cada 5 anos ou quando houver alterações de sintomas ou na terapia anti-inflamatória. Testes não invasivos para investigação de isquemia miocárdica e/ou aterosclerose podem ser úteis nessa estimativa (Tabela 14).^2^

**Tabela 14:** Recomendações sobre os parâmetros ecocardiográficos de ressonância magnética e tomografia computadorizada cardíaca na avaliação cardiovascular da artrite reumatoide

Recomendação	Força da recomendação	Certeza da evidência
ETT para a análise do SLG para detecção de disfunção ventricular subclínica precoce.^286^	Fraca	Moderada
ETT para o cálculo da FEVE para análise da função sistólica global.^274^	Forte	Alta
ETT para o cálculo do índice de performance miocárdica para análise da função sisto-diastólica.^287^	Fraca	Moderada
ETT para a análise de disfunção diastólica precoce do VE pela avaliação das ondas E/A e relação E/e'.^274^	Forte	Moderada
ETT para análise do diâmetro do átrio esquerdo, que reflete cronicidade da disfunção diastólica.^274^	Forte	Moderada
ETT para análise do volume atrial esquerdo indexado que reflete a cronicidade da disfunção diastólica.^288^	Fraca	Moderada
ETT para análise do espessamento/derrame pericárdico indicando atividade inflamatória e risco de complicações.^279^	Fraca	Moderada
ETT para a análise do SLG do ventrículo direito que pode estar reduzido na presença de inflamação ou hipertensão pulmonar.^280^	Fraca	Baixa
ETT para a análise da massa ventricular esquerda para análise do remodelamento cardíaco por inflamação crônica.^274,278^	Fraca	Moderada
ETT para o cálculo da pressão sistólica da artéria pulmonar para análise de hipertensão pulmonar associada à artrite reumatoide.^274^	Forte	Alta
ETT para avaliação valvar (espessamento/refluxo) que identifica valvopatias inflamatórias.^273^	Fraca	Moderada
Doppler de carótidas para avaliação de espessamento médio-intimal e risco cardiovascular.^284,289^	Forte	Alta
Ecocardiografia 3D para análise de volumes do VE, FEVE e válvulas.^281^	Fraca	Moderada
RMC para avaliação prognóstica e de alterações cardiovasculares após intervenções terapêuticas.^290,291^	Fraca	Moderada
TCC para avaliação de doença aterosclerótica.^285,292^	Forte	Alta

ETT: ecocardiograma transtorácico; FEVE: fração de ejeção do ventrículo esquerdo; RMC: ressonância magnética cardíaca; SLG: *strain* longitudinal global; TCC: tomografia computadorizada cardíaca; VE: ventrículo esquerdo.

A periodicidade dos exames de imagem cardíaca em pacientes com AR deve ser guiada por sintomas, evolução clínica, fatores de risco e mudanças terapêuticas, não havendo protocolo de rastreamento periódico para pacientes assintomáticos.^2,211,212^

**Mensagens-chave**

- As principais manifestações cardíacas da AR são pericardite, miocardite e lesão endotelial levando ao aumento do risco cardiovascular.
- A redução da FEVE (< 50%) está associada a pior prognóstico, mas é um achado tardio na maioria dos casos.
- A DD de Grau II ou superior está ligada a risco aumentado de IC e eventos cardiovasculares.
- Pacientes com AR frequentemente apresentam redução do SLG, mesmo com FEVE preservada, indicando dano miocárdico precoce.
- O aumento da massa miocárdica está associado s a disfunção miocárdica progressiva e maior risco de IC, podendo ocorrer mesmo sem hipertensão arterial, refletindo inflamação crônica.
- Técnicas de medicina nuclear, como o PET/CT com ^18^F-FDG, podem ser empregadas em pacientes com AR para a avaliação de inflamação miocárdica, vascular aórtica e carotídea além da detecção de isquemia miocárdica pela CPM.
- O espessamento ou derrame pericárdico são comuns em pacientes com AR de longa data, indicando pericardite crônica ou aguda, com achados persistentes associados a maior atividade da doença e complicações.
- A RMC com avaliação morfofuncional e caracterização tecidual é útil na detecção de acometimento miocárdico, e o protocolo deve incorporar cineRM, RT e técnicas de mapeamento de T1/T2.
- A investigação de doença aterosclerótica, quando indicada, pode ser feita com precisão com angiotomografia coronariana com o escore de cálcio.

### 4.6. Arterite de Takayasu

A arterite de Takayasu (AT) é uma vasculite inflamatória rara, inicialmente descrita em 1908 no Japão, que afeta preferencialmente vasos de grande calibre, especialmente a aorta e seus ramos, podendo também afetar artérias coronárias e artérias pulmonares em menor grau.^302^ A doença possui maior incidência em pacientes com menos de 40 anos de idade e do gênero feminino.^303^

As manifestações clínicas mais comuns na fase inicial da doença incluem febre, artralgia, mialgia, sudorese noturna, inapetência e emagrecimento. No entanto, o diagnóstico costuma ser realizado em estágios mais avançados, quando já se observa fibrose dos vasos, resultando em alteração dos pulsos periféricos e diferença pressórica entre os membros superior a 10 mm Hg. Nessa fase, pode-se também detectar sopro e frêmito ao longo das artérias. Além disso, o paciente frequentemente apresenta hipertensão arterial, que é uma das causas mais comuns de diagnóstico dessa condição.^304^ Portanto, recomenda-se que todos os pacientes jovens com hipertensão arterial sem causa definida sejam investigados para AT.

Os critérios diagnósticos sugeridos pelo *American College of Rheumatology* (ACR)^305^ estão descritos a seguir. É necessário que os pacientes apresentem pelo menos três dos seis parâmetros indicados:

1. Idade < 40 anos.
2. Diminuição dos pulsos braquiais.
3. Claudicação de extremidades.
4. Diferença de 10 mm Hg na pressão arterial sistólica dos membros superiores.
5. Sopros na aorta e nas artérias subclávias.
6. Alterações angiográficas de aorta e seus arcos principais.

Entre os exames laboratoriais, recomenda-se fazer uma avaliação global, com ênfase nos marcadores de atividade inflamatória (PCR e VHS), que podem ser úteis na monitorização da doença. Embora o diagnóstico seja estabelecido através de critérios clínicos, o PET/CT tem sido empregado tanto para auxiliar na confirmação da doença como no monitoramento da atividade inflamatória, seja na avaliação da resposta ao tratamento instituído ou na suspeita de recaída do processo inflamatório, sobretudo nos casos em que os sintomas são inespecíficos e os demais exames são inconclusivos.^306,307^

**Figura 17:**
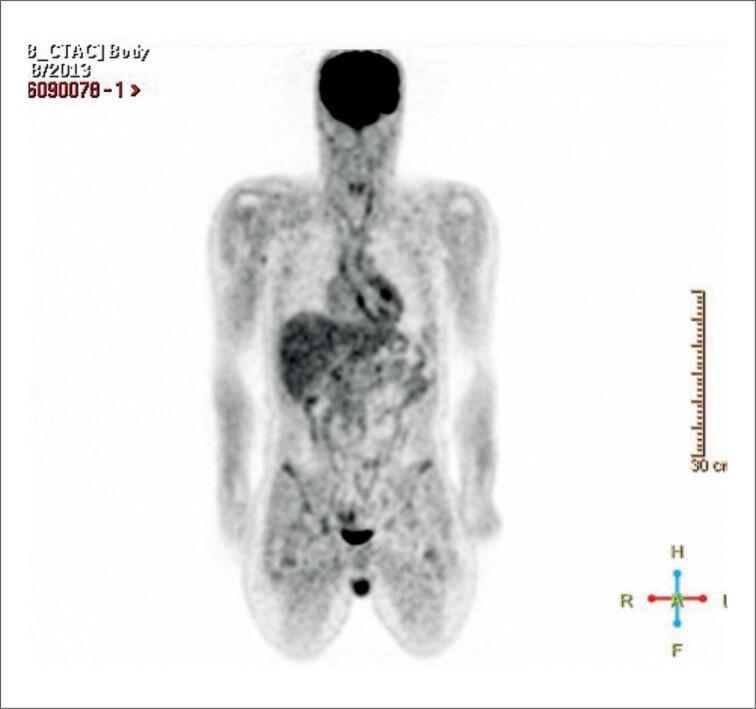
Paciente com suspeita de arterite de Takayasu, apresentando parestesia no membro superior direito aos esforços. O estudo Doppler de membros superiores evidenciou estenose de artéria subclávia direita. Corte coronal de imagem de PET com ^18^F-FDG evidencia captação heterogênea do radiotraçador na aorta ascendente e nas carótidas, sugerindo vasculite.

Assim, em pacientes com AT, recomenda-se a avaliação de toda a extensão da aorta por meio de ressonância nuclear magnética ou angiotomografia com ou sem ^18^F-FDG o mais precoce possível, para não postergar a imunossupressão (grau de evidência 1C).^307–309^ A tomografia associada ao marcador ^18^F-FDG (PET) apresenta alta especificidade na arterite de grandes células e pode ser benéfica nos pacientes com AT para determinação da atividade inflamatória além do grau de acometimento do vaso (Figura 17).^310^ As células inflamatórias ativadas apresentam avidez pela ^18^F-FDG, um análogo de glicose utilizado na realização de exames de PET/CT, capaz de avaliar doenças inflamatórias e/ou infecciosas, tendo aplicabilidade no contexto da AT.^306,311^

Algumas das vantagens do PET/CT em relação a outros métodos de imagem são o acoplamento da imagem anatômica com a imagem funcional, a estimativa da intensidade do processo inflamatório medida pelo grau de captação do radiotraçador, a realização de imagens de corpo inteiro em um único exame, com a identificação da extensão do acometimento, a ausência de prejuízo à função renal quando o exame é realizado sem contraste iodado e a baixa incidência de alergia ao radiofármaco. As potenciais desvantagens são o alto custo, a baixa disponibilidade e o uso de radiação ionizante.^312^

Em uma metanálise publicada em 2018 (10 estudos e 301 pacientes), demonstrou-se sensibilidade de 81% e especificidade de 71% para identificação de AT em comparação a critérios clínicos.^307^ Outra metanálise avaliou o valor do PET/CT na monitorização da resposta ao tratamento na vasculite de grandes vasos, incluindo a arterite de células gigantes (4 estudos e 111 pacientes), encontrando sensibilidade de 77% e especificidade de 71% na distinção entre doença ativa e remissão.^308^ Algumas das limitações que impactam a análise da acurácia diagnóstica do PET/CT na AT contemplam o pequeno número de pacientes avaliados, os critérios de inclusão diversos e os diferentes parâmetros analisados na imagem entre os estudos.^313–315^

**Figura 18:**
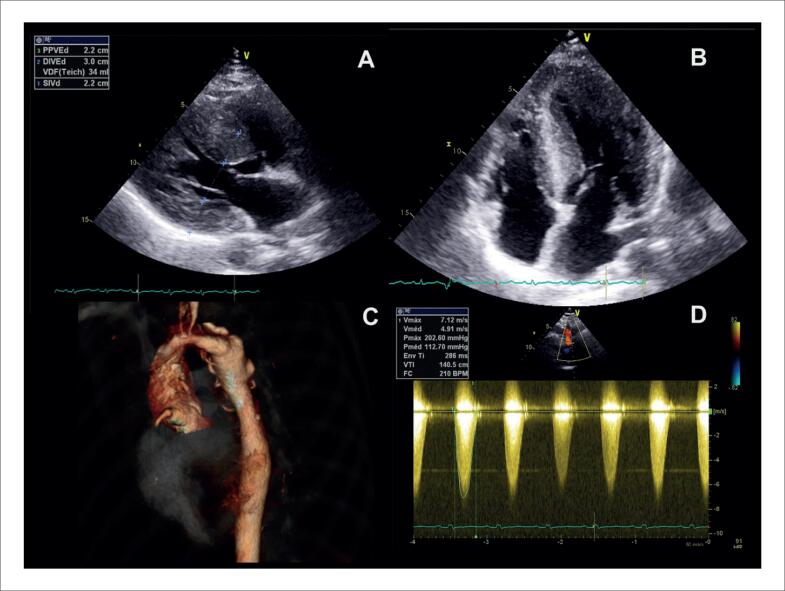
Imagens ecocardiográficas (A, B e D) e de angiotomografia de aorta torácica (C) de paciente com diagnóstico de arterite de Takayasu. A paciente apresenta hipertrofia miocárdica concêntrica de grau importante (A e B) secundária à estenose acentuada de arco aórtico, aorta torácica descendente, além de oclusão do tronco braquiocefálico e estenose acentuada da artéria carótida comum esquerda e artéria subclávia esquerda (C). Ao Doppler contínuo, foi medido gradiente máximo ao corte supraesternal em aorta descendente de 202 mm Hg.

A TC é menos invasiva que a arteriografia por cateter e pode demonstrar claramente o espessamento da parede do vaso, o que pode não ser visível no cateterismo^316^ (Figura 18). A RM também pode ser utilizada para determinar o grau de acometimento da parede do vaso, tendo como maior vantagem, em relação à TC, a ausência de radiação ionizante, o que pode ser benéfico para o acompanhamento de pacientes jovens devido à necessidade de repetição frequente da avaliação da aorta.^217^ Tanto a tomografia (com ou sem ^18^F-FDG) como a RM demonstram as lesões decorrentes da inflamação da doença com aorta de aspecto tortuoso, alternando segmentos de estenose com dilatação ou oclusão e acometimento dos ramos da aorta, em especial das artérias carótidas, subclávia esquerda e mesentérica.^318^ A atividade da doença pode ser monitorizada com provas de função inflamatória e exames de imagem (RM ou angio-TC).

**Figura 19:**
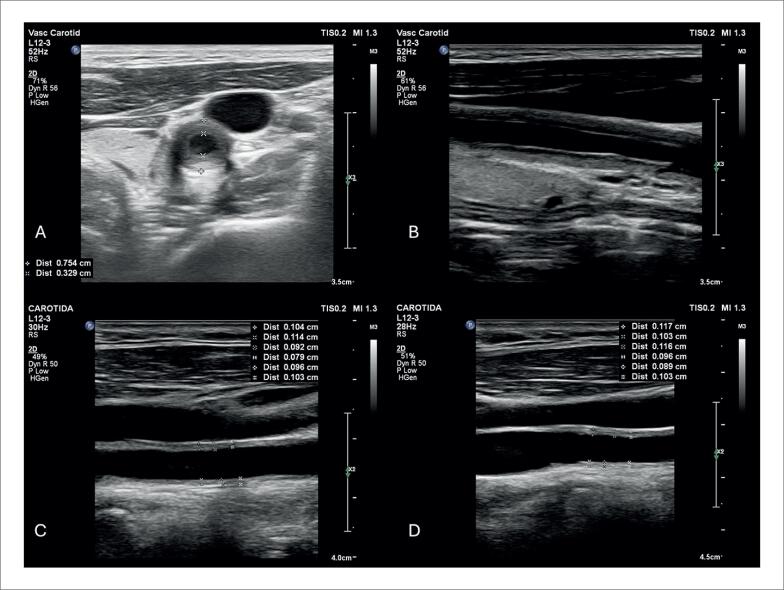
Fases do espessamento concêntrico na arterite de Takayasu. Cortes ao Modo B transverso (A) e longitudinal (B) demonstrando o grande espessamento concêntrico, de baixa ecogenicidade e com intermeios de ecolucência, da parede da artéria carótida comum, em uma paciente na fase de atividade da doença. Cortes longitudinais ao Modo B demonstrando espessamento menos acentuado e mais ecogênico da parede da artéria carótida comum (C) e poupando o ramo interno (D). Imagem cedida pela Dra. Cláudia Maria Vilas Freire.

Embora a ultrassonografia vascular (USV) tenha limitações técnicas para a avaliação do acometimento da aorta torácica na AT, é de importante utilização para a detecção do acometimento dos ramos aórticos como artérias subclávias, artérias carótidas comuns poupando os ramos carotídeos, artérias vertebrais e ramos abdominais (Figura 19). Além de auxiliar no diagnóstico da AT, a USV pode monitorar a evolução e os estágios da doença e sua resposta ao tratamento implementado.^319^ Durante o período da atividade inflamatória, a USV visualiza segmentos arteriais de espessamento concêntrico, de característica homogênea e ecogenicidade baixa ou intermediária, podendo coexistir com áreas hipoecogênicas relacionadas ao edema e/ou neovascularização no interior do espessamento concêntrico.^320^ Além da neovascularização, um aumento do calibre da artéria > 10 mm sugere atividade da doença.^321^ Esse espessamento concêntrico pode levar a obstruções e oclusões arteriais, com isquemia de órgão-alvo. Por outro lado, quando a USV detecta segmentos artérias com espessamento concêntrico hiperecogênico, por maior presença de tecido fibrótico, há maior relação com estágios crônicos da AT.^319,322^

Recomenda-se que:

- A ultrassonografia pode ser utilizada para a detecção de inflamação mural e/ou alterações luminais em ramos aórticos nos pacientes com suspeita de AT, tendo valor limitado para a avaliação da aorta torácica;
- A USV pode ser usada para o monitoramento em longo prazo de danos estruturais, particularmente para detectar estenose, oclusão, dilatação e/ou aneurismas. A frequência da triagem e o método de imagem aplicado devem ser decididos individualmente.

O ecocardiograma tem um papel secundário na doença e pode ser utilizado tanto para detectar alterações secundárias a estenoses segmentares da aorta e seus ramos, hipertensão arterial (hipertrofia ventricular) (Figura 18) e até mesmo acometimento direto de outros vasos, como as artérias coronárias (até 25% dos pacientes podem apresentar estenose) e artérias pulmonares, que podem apresentar-se dilatadas. Pode, também, ser detectada aceleração do fluxo em aorta abdominal (no plano subxifoide) e insuficiência das valvas mitral ou aórtica (esta podendo ocorrer em até 55% dos pacientes) (Tabela 15).^323^

**Tabela 15:** Recomendações para o uso do PET/CT com ^18^F-FDG na arterite de Takayasu

Recomendação	Força da recomendação	Certeza da evidência
Diagnóstico de inflamação vascular ativa, intensidade e extensão do acometimento.^307–309^	Fraca	Baixa
Monitoramento da resposta ao tratamento.^307–2,309^	Fraca	Baixa

PET/CT com ^18^F-FDG: tomografia por emissão de pósitrons associada a tomografia computadorizada com fluordesoxiglicose-flúor-18 (do inglês *Fluorodeoxyglucose Positron Emission Tomography/Computed Tomography*).

A periodicidade da realização dos exames de imagem não está muito bem estabelecida, mas pode variar de 3 a 6 meses, de acordo com a avaliação clínica de cada paciente e a atividade da doença.^316^ No entanto, de acordo com as recomendações da *European League Against Rheumatism* (EULAR), os pacientes em remissão devem realizar USV, RM ou TC anualmente, mesmo na ausência de atividade inflamatória após a introdução da medicação, visto que pode ocorrer acometimento subclínico dos vasos.^308^

**Mensagens-chave**

- A AT é uma vasculite inflamatória rara que frequentemente causa hipertensão arterial e afibrose dos vasos, resultando em sopros em aorta e artérias subclávias, alteração dos pulsos periféricos e diferença pressórica superior a 10 mm Hg entre os membros.
- Em pacientes com AT, recomenda-se a avaliação de toda a aorta, realizada com ressonância nuclear magnética ou TC com ou sem ^18^F-FDG.
- A tomografia associada ao marcador ^18^F-FDG (PET) apresenta alta especificidade na arterite de grandes células e pode ser benéfica nos pacientes com AT para determinação da atividade inflamatória além do grau de acometimento do vaso.
- A angio-TC e a angio-RM podem ser utilizadas para determinar o grau de acometimento da parede do vaso, demonstram as lesões decorrentes da AT com aorta de aspecto tortuoso, alternando segmentos de estenose com dilatação ou oclusão e acometimento dos ramos da aorta, em especial das artérias carótidas, subclávia esquerda e mesentérica.
- A USV é de importante utilização para a detecção do acometimento de ramos aórticos, como artérias subclávias e artérias carótidas comuns, poupando os ramos carotídeos, artérias vertebrais e ramos abdominais.
- A USV pode monitorar a evolução e os estágios da doença, assim como a resposta terapêutica.

## 5. Aplicações da Multimodalidade em Imagem Cardíaca nas Doenças de Depósito

### 5.1. Cardiomiopatia por Sobrecarga de Ferro

O termo cardiomiopatia por sobrecarga de ferro (CSF) foi introduzido para descrever a cardiomiopatia resultante do acúmulo de ferro no miocárdio, principalmente devido a distúrbios geneticamente determinados do metabolismo do ferro ou múltiplas transfusões.^324^ A CSF, além de ser uma das principais causas de morbidade, é responsável por um terço das mortes na hemocromatose hereditária (HH) e uma das principais causas de morte em outras condições associadas à sobrecarga secundária de ferro.^325,326^ A sobrecarga de ferro pode ser primária ou secundária (Tabela 16).

**Tabela 16:** Principais condições que levam à sobrecarga de ferro

Sobrecarga de ferro primária	Sobrecarga de ferro secundária	Outras condições
Hemocromatose hereditária	Anemias hereditárias	Doença hepática crônica
➢ Tipo I: relacionada ao gene *HFE*	Hemoglobinopatias	Ataxia de Friedreich
➢ Tipo II: juvenil	Talassemia	Aceruloplasminemia
Subtipo A: relacionada ao gene *HJV*	Anemia falciforme	Atransferrinemia congênita
Subtipo B: relacionada ao gene *HAMP*	Anemia Diamond-Blackfan	Aumento da ingestão alimentar
➢ Tipo III: relacionada ao gene *TfR2*	Anemia diseritropoiética congênita	
➢ Tipo IV: relacionada à ferroportina	Anemia sideroblástica	
	Anemias adquiridas	
	Síndromes mielodisplásicas	
	Mielofibrose	
	Anemia aplástica	
	Leucemias	
	Doenças mieloproliferativas	
	Transplante de células-tronco	
	Doença renal crônica	

A forma primária de sobrecarga de ferro é denominada HH, doença genética autossômica recessiva resultante de mutações em genes que codificam proteínas envolvidas no metabolismo do ferro, causando absorção elevada de ferro pelo epitélio intestinal, além de distúrbio adicional do seu metabolismo, o que determina acúmulo do metal e, consequentemente, lesão de vários órgãos.^327,328^

A sobrecarga secundária de ferro é causada principalmente por administração elevada de ferro parenteral e é principalmente observada em associação com anemias hereditárias ou adquiridas dependentes de transfusões sanguíneas repetitivas que saturam as células do sistema reticuloendotelial com ferro, que então se espalha para outras células parenquimatosas (Tabela 16).^329^

A deposição de ferro ocorre, inicialmente, no miocárdio ventricular e, posteriormente, no miocárdio atrial, afetando também o sistema de condução, especialmente o nó atrioventricular, mas em menor extensão em comparação com o miocárdio funcional.^329^ Deve-se notar que a sobrecarga de ferro no miocárdio, embora tenha papel fundamental como fator desencadeante no desenvolvimento de CSF, não é o único mecanismo envolvido, sendo um tipo específico de cardiomiopatia com fisiopatologia complexa envolvendo fatores imunoinflamatórios e genéticos adicionais que interferem na patogênese.

Dois fenótipos de CSF foram identificados:^325^

- Fenótipo dilatado: caracterizado por remodelamento do VE que leva à dilatação das câmaras e redução da FEVE;
- Fenótipo restritivo: caracterizado por DD do VE com enchimento restritivo, FEVE preservada, HP e subsequente dilatação do VD.

Esses dois fenótipos são seguidos por várias outras manifestações, incluindo anormalidades do sistema de condução, taquiarritmias e miopericardite. Se a causa da sobrecarga de ferro persistir e nenhuma terapia de quelação de ferro adequada for iniciada, a maioria dos pacientes com CSF desenvolverá remodelamento do VE que, em fases avançadas, leva à dilatação do VE e à redução da FEVE – fenótipo dilatado.^325,330^

Em uma minoria de casos (10%) caracterizados por sobrecarga grave de ferro, a disfunção restritiva do VE leva, em fase avançada, ao desenvolvimento de HP, dilatação do VD e IC direita sem remodelamento anatômico do VE e com FEVE preservada, mesmo nos estágios finais – fenótipo restritivo.^330–332^

A CSF apresenta algumas características clínicas distintas e requer abordagem diagnóstica e terapêutica específica, devendo ser considerada em qualquer paciente com IC de etiologia desconhecida. Os pacientes podem ser assintomáticos no início da doença. Uma vez que a IC se desenvolve, há rápida deterioração.

**Figura 20:**
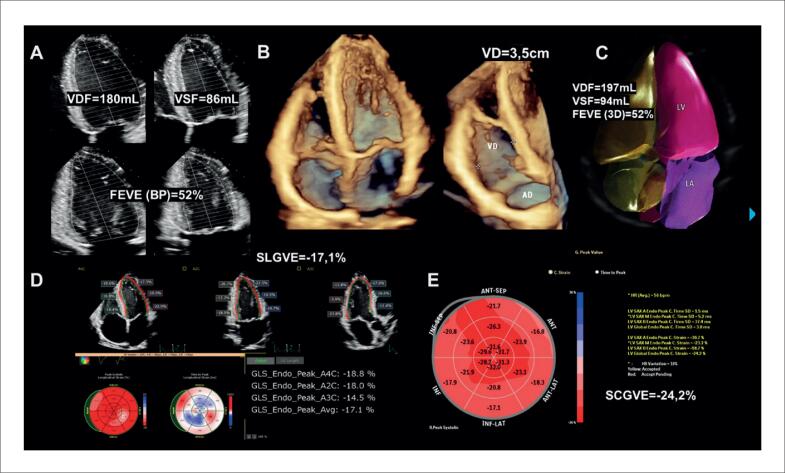
Imagens ecocardiográficas de paciente de 25 anos, portador de hemocromatose hereditária. Volumes ventriculares e FEVE avaliados pelo método de Simpson ao ecocardiograma bidimensional (A) e pelo ecocardiograma tridimensional (B e C) evidenciando função sistólica no limite inferior da normalidade e discreta dilatação ventricular. Observa-se redução discreta do SLG do VE (-17,1%) e do SCG (-24,2%) (D e E). FEVE: fração de ejeção do ventrículo esquerdo; SCG: strain circunferencial global; SLG: strain longitudinal global; VD: ventrículo direito; VDF: volume diastólico final do ventrículo esquerdo; VE: ventrículo esquerdo; VSF: volume sistólico final do ventrículo esquerdo.

A ecocardiografia, embora apresente achados inespecíficos, é a principal modalidade utilizada na triagem de pacientes com condições de sobrecarga de ferro para doença cardíaca como parte da avaliação inicial.^325^ Anormalidades da função sistólica e diastólica ventriculares, e também o envolvimento pericárdico e valvar, podem ser detectados. Em estágios iniciais, pode ser evidenciada dilatação discreta das cavidades cardíacas e redução mínima da FEVE. Nesses pacientes, a análise do SLG do VE pode auxiliar na comprovação de disfunção miocárdica incipiente (Figura 20).

Alto débito cardíaco com dilatação das câmaras, hipertrofia excêntrica do VE e FEVE normal ou aumentada também podem ser observados.^333^ Deve-se observar que a DD do VE e a FEVE reduzida podem ser mascaradas por estado de alto débito induzido por anemia em pacientes com hemoglobinopatias e outras condições hematológicas. Assim, o padrão pseudonormal pode ser frequentemente encontrado no fluxo transmitral,^325^ que pode ser desmascarado por refinamento da análise da função diastólica com estudo do padrão de fluxo da veia pulmonar ou do movimento do anel mitral por meio de imagens de Doppler tecidual.

Embora métodos de imagem sofisticados, como o *strain* longitudinal do VE, possam ser marcadores mais sensíveis de disfunção subclínica do VE, o desempenho de tais parâmetros tem obtido resultados ainda não definidos e controversos em populações com hemocromatose.^334,335^ Estudos prospectivos são necessários para avaliar melhor a disfunção subclínica do VE em indivíduos com HH tratados cronicamente.

A ecocardiografia identifica as consequências do ferro na estrutura e função miocárdicas, porém, ela não prevê com precisão o conteúdo de ferro no miocárdio. No entanto, ela consiste em um método simples para triagem de pacientes assintomáticos e acompanhamento de pacientes com patologia conhecida.^325^

A RMC com relaxometria T2* (T2-star) é um parâmetro que se encurta na presença de ferro devido ao efeito paramagnético do metal. Valores de T2* abaixo de 20 ms indicam sobrecarga clinicamente relevante, enquanto valores inferiores a 10 ms estão associados a um risco elevado de disfunção cardíaca e arritmias graves.^336,337^ Há uma forte correlação entre os valores de T2* e a concentração de ferro medida em biópsias, validando a RMC como um método confiável para a avaliação não invasiva.^338,339^ Além disso, esta técnica permite o mapeamento de todo o ventrículo, evitando erros de amostragem que podem ocorrer na biópsia.

Outra ferramenta promissora é o mapeamento T1. Na sobrecarga cardíaca de ferro estabelecida, T1 e T2* são concordantes. Na CSF, os valores de T1 nativo apresentam-se reduzidos. No entanto, na faixa de 20 a 30 ms de T2*, o mapeamento de T1 parece detectar ferro de forma mais precoce.^340–342^

Em casos avançados, o RT miocárdico pode identificar áreas de fibrose miocárdica, que estão associadas a um pior prognóstico.^343^ Embora a fibrose não seja um achado comum na hemocromatose primária, sua presença em pacientes com sobrecarga secundária de ferro (como em talassemias) pode indicar doença mais avançada.

Outra vantagem significativa da RMC é a sua capacidade de monitorar a resposta ao tratamento. Terapias como flebotomias ou uso de quelantes de ferro (desferoxamina, deferasirox ou deferiprona) podem reduzir a carga de ferro miocárdico, e a RMC permite acompanhar essa redução de forma seriada. Estudos longitudinais em talassemia mostraram que a melhora nos valores de T2* após terapia está associada à recuperação da função cardíaca e à redução de eventos adversos.^331,344^ As principais alterações encontradas na RMC em pacientes com hemocromatose estão descritas na Tabela 17.

**Tabela 17:** Principais alterações na hemocromatose avaliada pela RMC

Morfologia	Realce tardio	Mapa de T1	T2 ou T2*	VEC
HVE simétrica	Raro nos estágios iniciais	Reduzido	T2* reduzido	N/A

HVE: hipertrofia ventricular esquerda; N/A: não aplicável; RMC: ressonância magnética cardíaca; VEC: volume extracelular.

A função do VD pode ser prejudicada por disfunção ventricular esquerda e HP.^325^ Mais recentemente, a análise de dados de RMC em pacientes com talassemia maior mostrou que a fração de ejeção do VD (FEVD) diminuiu progressivamente com o aumento da carga de ferro miocárdica, seguindo padrão semelhante ao da FEVE.^345^

**Figura 21:**
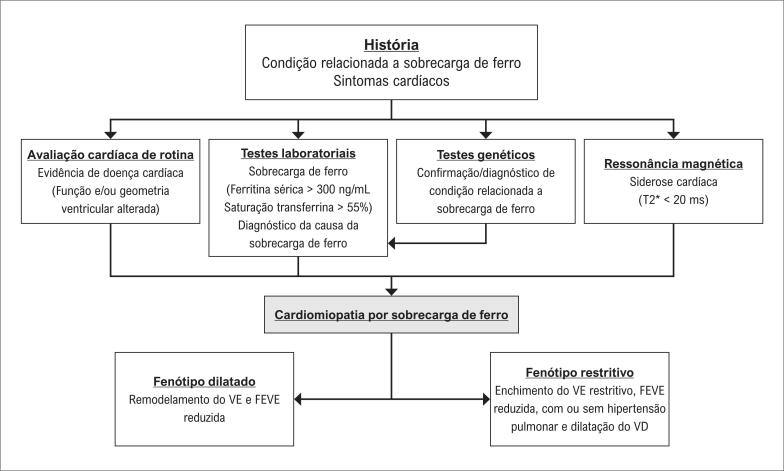
Diagnóstico de CSF com base no algoritmo proposto. O diagnóstico requer a presença de (1) sobrecarga de ferro (ferritina sérica > 300 ng/mL, saturação de transferrina > 55%), (2) siderose cardíaca (T2* < 20 ms) e (3) evidência de doença cardíaca. CSF: cardiomiopatia por sobrecarga de ferro; VE: ventrículo esquerdo; FEVE: fração de ejeção do ventrículo esquerdo; VD: ventrículo direito. Adaptado de Kremastinos e Farmakis.^331^

Finalmente, deve-se notar que níveis de ferritina sérica não se correlacionam com a gravidade da sobrecarga de ferro miocárdico. A alta deposição de ferro miocárdico pode ocorrer apesar dos baixos níveis de ferritina sérica.^346^ Portanto, o diagnóstico de CSF é feito quando a evidência de doença cardíaca, particularmente DD^324,347^ do VE com enchimento restritivo ou remodelamento do VE com dilatação das câmaras e FEVE reduzida, coexiste com sobrecarga de ferro (ferritina sérica > 300 ng/mL, saturação de transferrina > 55%) e siderose cardíaca (T2* cardíaco inferior a 20 ms) conforme esquematizado na Figura 21.

Recomendações práticas sobre o uso da ecocardiografia em pacientes com CSF conhecida ou suspeita seguindo o sistema GRADE são apresentadas na Tabela 18.

**Tabela 18:** Recomendações para o uso da ecocardiografia para pacientes com cardiomiopatia por sobrecarga de ferro suspeita ou confirmada

Recomendação	Força da recomendação	Certeza da evidência
Ecocardiograma transtorácico com análise da geometria e função sistólica biventricular deve ser realizado como primeira linha na avaliação de condições de sobrecarga de ferro suspeita ou confirmada.^324,325,331,332^	Forte	Moderada
Ecocardiograma transtorácico com análise da função diastólica do VE, incluindo imagem de Doppler tecidual do anel mitral medial e lateral, deve ser realizado como primeira linha na avaliação de condições de sobrecarga de ferro suspeita ou confirmada.^331,332,338^	Forte	Moderada
*Strain* global longitudinal pode ser usado para diagnóstico de disfunção sistólica do VE subclínica em condições de sobrecarga de ferro suspeita ou confirmada.^334,335^	Fraca	Baixa
Ecocardiograma transtorácico deve ser realizado a cada 6 a 12 meses se o tempo de relaxamento T2* medido pela RMC for inferior a 20 ms e a cada 6 meses ou menos se o paciente se tornar sintomático.^326,331,334,336^	Fraca	Moderada
A RMC com análise dos volumes e função sistólica biventricular deve ser realizada na avaliação de condições de sobrecarga de ferro suspeita ou confirmada.	Forte	Moderada
A RMC com medição do tempo de relaxamento T2* deve ser realizada em todos os pacientes em avaliação de sobrecarga de ferro suspeita ou confirmada.^336–339^	Fraca	Moderada
A RMC com medição do tempo de relaxamento T1 pode ser realizada nos pacientes em avaliação de sobrecarga de ferro suspeita ou confirmada.^340–342^	Fraca	Baixa
A RMC com medição do tempo de relaxamento T2* deve ser realizada no acompanhamento de pacientes com sobrecarga de ferro em tratamento (quelantes e flebotomia), pois a melhora nos valores de T2* correlaciona-se com a recuperação da função cardíaca e a redução de eventos adversos.^331,344^	Fraca	Moderada

RMC: Ressonância magnética cardíaca; T1: relaxamento T1; T2*: relaxamento T2 estrela; VE: ventrículo esquerdo.

Em pacientes com CSF, a Ecocardiografia deve ser realizada a cada 6 a 12 meses se o tempo de relaxamento T2* medido pela RMC for inferior a 20 ms e a cada 6 meses ou menos se o paciente se tornar sintomático. É recomendada a realização periódica de ETT com avaliação da função diastólica a cada 1–2 anos, mesmo em pacientes assintomáticos.^324,347^

**Mensagens-chave**

- A DD do VE é um achado precoce na CSF e pode ser detectada pela ecocardiografia com Doppler tecidual.
- Na presença de DD do VE e/ou redução da velocidade sistólica tecidual máxima do anel mitral detectada por ecocardiografia, deve-se realizar RMC com medição do tempo de relaxamento T2*.
- RMC com medição do tempo de relaxamento T2* deve ser realizada em todos os pacientes com cardiomiopatia idiopática.
- A RMC através da técnica de relaxamento T2* permite a avaliação da concentração de ferro no miocárdio.
- O mapeamento T1 é complementar ao T2*, sendo capaz de detectar depósito de ferro em estágios mais precoces, especialmente com T2* entre 20 e 30 ms.
- No acompanhamento do tratamento com quelantes e flebotomia, a melhora nos valores de T2* correlaciona-se com recuperação da função cardíaca e redução de eventos adversos.

### 5.2. Doença de Fabry

A doença de (DF) é uma condição rara decorrente da existência de variantes patogênicas no gene *GLA* e que determinam a deficiência da enzima alfa galactosidase A (α-Gal A), responsável pelo metabolismo de glicoesfingolipídeos (GL-3, lyso-Gb3) no interior dos lisossomos. O resultado é a formação de depósitos celulares, observados desde a vida intrauterina, causadores de intenso processo inflamatório e estresse oxidativo com evolução para apoptose celular e falência orgânica multissistêmica.^348^

As manifestações clínicas dependem do genótipo, uma vez que existem variantes patogênicas determinantes de fenótipo clássico e outras responsáveis pelo fenótipo não clássico, também conhecido pelo termo "*late onset*" ou início tardio. No primeiro subgrupo, a atividade enzimática inferior a 5% ou ausente determina sintomas muito precoces em crianças desde os 2 anos de idade. Os principais achados incluem angioqueratomas, hipohidrose, distúrbios gastrointestinais, alterações de córnea (verticilata), crises dolorosas por neuropatia periférica e proteinúria de intensidade variável. Arritmias supraventriculares e ventriculares, intolerância aos esforços e insuficiências valvares discretas podem estar presentes nessas fases iniciais. Essas alterações tendem a piorar na adolescência em casos não tratados com terapia específica, seja a terapia de reposição enzimática (TRE) ou chaperona oral. Na idade adulta, pode ocorrer insuficiência renal, aumento importante da espessura miocárdica e ICFEp, arritmias, isquemia miocárdica e doença cerebrovascular, com rápido desenvolvimento para doença avançada e necessidade de terapias substitutivas como a diálise.^349,350^

O fenótipo não clássico, por outro lado, em função da presença de níveis residuais de atividade enzimática, apresenta sintomas de início mais tardio, geralmente na idade adulta, e restritos a órgãos como o coração e o rim. Isso, no entanto, não impede o aparecimento de cardiomiopatia e nefropatia tão grave quanto os observados nas formas clássicas. ^349,350^

O diagnóstico definitivo é realizado pela identificação da variante patogênica associada à dosagem dos níveis de atividade enzimática da α-Gal A associada à presença de manifestações clínicas, ou de alterações histológicas típicas, ou pela elevação dos níveis de biomarcadores específicos (Gb3, lyso-Gb3) ou, ainda, pela existência de familiares com doença documentada.^351^

A complexidade da apresentação clínica da doença reforça a necessidade de trabalho multidisciplinar, que inclui médicos geneticistas e de especialidades como cardiologia, neurologia, nefrologia entre outros. É também importante a integração de cuidados de enfermagem, nutrição e psicologia na otimização do acompanhamento com melhora da qualidade de vida dos pacientes.^352^

Cabe ao cardiologista um papel de protagonismo, uma vez que manifestações cardiovasculares e renais são as principais causas de morbimortalidade na DF no Brasil. As queixas clínicas e as alterações em exames complementares cardiológicos podem determinar a fase de evolução da doença e fornecem informações diagnósticas e prognósticas que podem indicar a necessidade de tratamento específico.^353^

**Figura 22:**
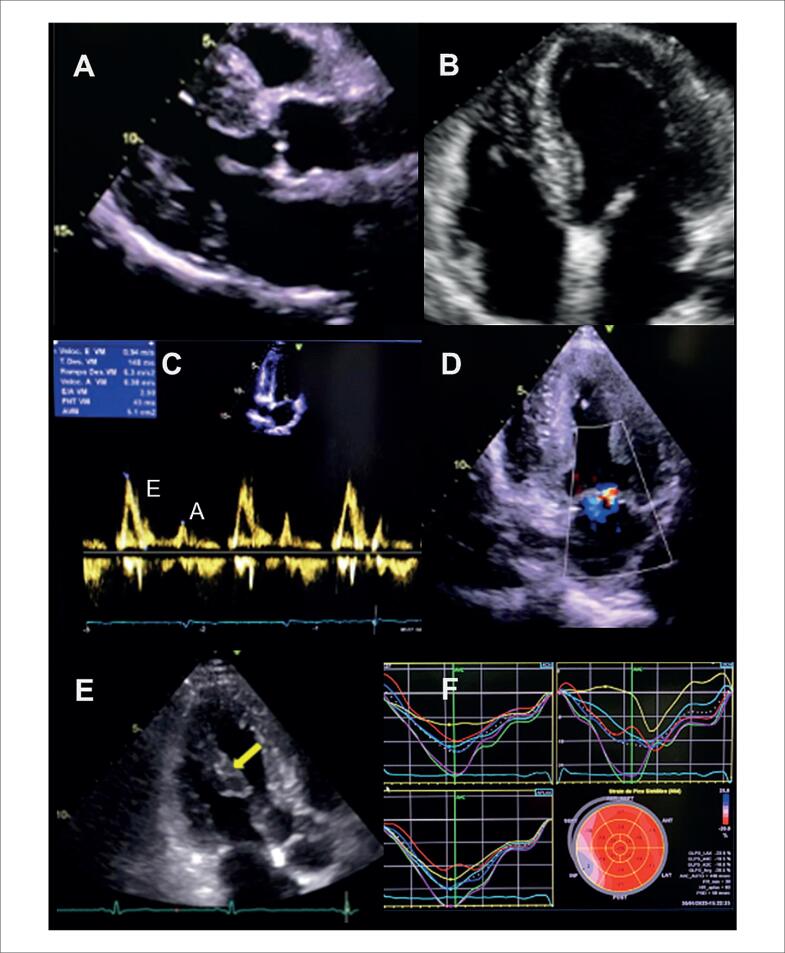
Achados ecocardiográficos na doença de Fabry (DF). A) Janela paraesternal longitudinal evidenciando aumento da espessura das paredes do ventrículo esquerdo. B) Janela apical quatro câmaras evidenciando o sinal binário endocárdico (aspecto de birrefringência do miocárdio) em parede septal inferior. C) Doppler do fluxo transmitral com padrão de disfunção diastólica importante (relação E/A > 2). D) Janela apical quatro câmaras evidenciando refluxo mitral discreto. E) Janela apical três câmaras evidenciando aumento da espessura de músculo papilar (seta amarela). F) Representação das curvas de strain longitudinal segmentar e mapa paramétrico com alteração característica da DF evidenciando redução do strain segmentar em segmento basal da parede inferior.

**Figura 23:**
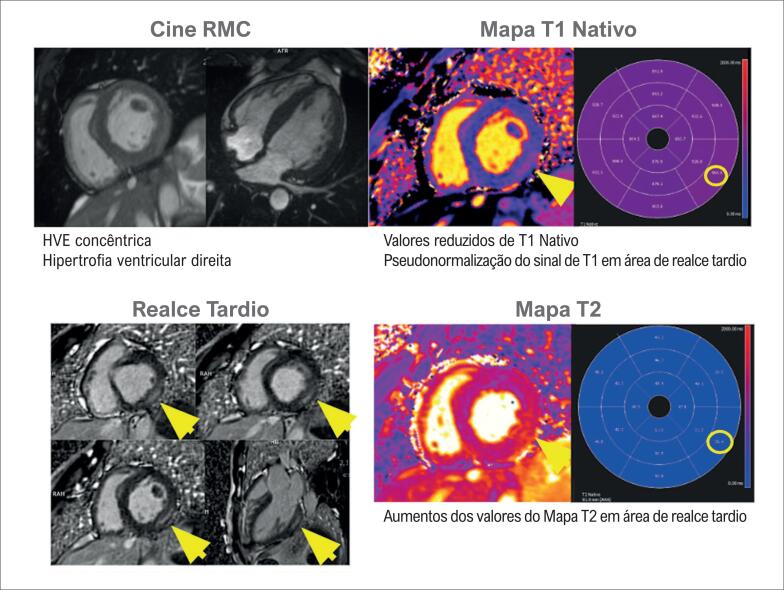
Imagens de ressonância magnética cardíaca de paciente com diagnóstico de Doença de Fabry. Imagens de cinerressonância com aumento concêntrico da espessura miocárdica biventricular. Sequências de mapeamento de T1 nativo evidenciam valores reduzidos de T1 nativo (seta amarela) e pseudonormalização do sinal de T1 em área de realce tardio. Imagens de realce tardio evidenciando fibrose em parede lateral inferior (setas amarelas). Valores aumentados ao mapa T2 em área de realce tardio indicando a presença de edema e inflamação. Imagens cedidas pela Dra Sílvia Aguiar Rosa.

O eletrocardiograma deve ser realizado para avaliação inicial do acometimento cardíaco da DF e repetido anualmente. Os achados eletrocardiográficos mais frequentes são o intervalo PR curto, os sinais de sobrecarga ventricular esquerda e intervalo QT corrigido inferior a 440 ms.^354^ O ecocardiograma é exame fundamental para a suspeita diagnóstica, principalmente em indivíduos assintomáticos ou em casos cujos sintomas sejam inespecíficos. Além disso, seu baixo custo e disponibilidade permitem a avaliação da evolução da cardiopatia no paciente índice e o diagnóstico fenotípico de familiares potencialmente acometidos. Os principais achados aos exames de imagem cardíaca estão descritos na Tabela 19, Figura 22 e Figura 23.

**Tabela 19:** Principais achados ao ecocardiograma e ressonância magnética cardíaca na doença de Fabry

Ecocardiografia	RMC
Aumento da espessura miocárdica do VE > 11 mm (mulheres) ou > 12 mm (homens)	HVE simétrica e concêntrica
Aumento da espessura de músculo papilar	RT subepicárdico e mesocárdico inferolateral e septal
Disfunção diastólica	MAPA T1 reduzido (atenção para a pseudonormalização)
Alteração de contratilidade segmentar (basal inferolateral)	MAPA T2 elevado
SLG e SRG reduzidos (segmento basal inferolateral)	

HVE: hipertrofia ventricular esquerda; RMC: ressonância magnética cardíaca; RT: realce tardio miocárdico; SLG: *strain* longitudinal global; SRG: *strain* radial global; T1: recuperação do relaxamento longitudinal, T2: decaimento do relaxamento transversal; VE: ventrículo esquerdo.

O aumento da espessura miocárdica ventricular esquerda e/ou direita (30 a 40% casos), mais frequentemente de padrão concêntrico, associado à fração de ejeção preservada, além de alterações da função diastólica, deve levantar a suspeita diagnóstica de DF. A incidência dessas alterações aumenta com o envelhecimento e tem prevalência semelhante em homens e mulheres.^354,355^

O uso de técnicas de imageamento 3D aumenta a acurácia da determinação da massa cardíaca sem a necessidade de inferências geométricas. Alterações da função diastólica em indivíduos jovens e não explicadas por doenças coexistentes costumam ser a manifestação ecocardiográfica mais precoce da doença e antecedem o desenvolvimento da hipertrofia. A FEVE costuma permanecer preservada ao longo de toda evolução natural da doença, e a piora da função sistólica está associada ao aparecimento de fibrose e/ou a coexistência de alterações tais como a disfunção renal. A presença de sinal binário endocárdico apresenta correlação histológica com a presença de depósitos endocárdicos de Gb3. Esses também podem acometer o tecido valvar, determinando a ocorrência de refluxos geralmente discretos. Ectasias da porção inicial da aorta torácica podem ser encontradas e, geralmente, não evoluem para formas aneurismáticas com potencial de dissecção.

A análise do SLG auxilia o diagnóstico diferencial com outras fenocópias e permite a identificação de alterações de contratilidade segmentar em segmento basal de parede inferolateral que podem passar despercebidas na avaliação ecocardiográfica convencional. Alterações no *strain* segmentar podem ser indicativas de alterações fibróticas que são marcadores de doença crônica e de mau prognóstico cardiovascular (Figura 22). O *strain* de parede livre do VD pode estar reduzido apesar da normalidade de outros marcadores de função sistólica, como o TAPSE e a velocidade da onda s’.^356–358^

A RMC permite caracterização tecidual miocárdica e fornece informações prognósticas, além de ter utilidade na avaliação de resposta terapêutica. A RMC permite não apenas a quantificação precisa da massa ventricular, mas também a detecção de padrões específicos de envolvimento miocárdico que auxiliam no diagnóstico diferencial de outras fenocópias.^359–362^ Uma das contribuições mais significativas da RMC no manejo da DF é a capacidade de detectar e quantificar a fibrose miocárdica através da técnica de RT miocárdico. O padrão característico de RT ocorre predominantemente nos segmentos basais inferolaterais do VE, tipicamente em localização mesocárdica ou subepicárdica, contrastando com o padrão de realce transmural observado na cardiomiopatia isquêmica ou o padrão subendocárdico difuso visto na amiloidose. Essa distribuição peculiar da fibrose reflete o processo fisiopatológico subjacente da doença, decorrente do acúmulo de globotriaosilceramida nos cardiomiócitos e nas células endoteliais.^362^ A presença e extensão do RT têm importante valor prognóstico, correlacionando-se com o risco de arritmias ventriculares e eventos cardíacos adversos.^362^

O mapeamento T1 nativo emergiu como outra ferramenta valiosa na avaliação por RMC. Devido ao acúmulo intracelular de esfingolipídios, os valores de T1 nativo estão reduzidos nessa condição, contrastando com o aumento observado na maioria das outras cardiomiopatias (Tabela 20). Deve haver cuidado especial na pseudonormalização do T1 nas áreas afetadas por RT. Esse achado é particularmente útil no diagnóstico diferencial, entre outras causas, de HVE, sendo considerado um marcador da doença (Figura 23). O mapeamento T2 pode apresentar discreto aumento global.^348^

**Tabela 20:** Recomendações sobre o uso da multimodalidade para o diagnóstico da doença de Fabry

Recomendação	Força da recomendação	Certeza da evidência
O ETT deve ser realizado como método de imagem de primeira linha na investigação de DF e repetido anualmente ou no caso de surgimento de novos sintomas cardíacos.^355,356^	Forte	Alta
O SLG alterado por diminuição da deformação miocárdica em segmento basal da parede inferolateral é característico da DF e auxilia o diagnóstico diferencial com outras doenças de fenótipo semelhante.^331,333^	Fraca	Moderada
Ecocardiograma deve ser usado para a triagem de familiares, no momento do diagnóstico de familiares de primeiro grau, quando o teste genético não é possível.^355,357^	Fraca	Moderada
A RMC é considerada o padrão-ouro de investigação cardíaca por permitir melhor caracterização do dano miocárdico e fornecer informações prognósticas e eficácia da resposta terapêutica.^358–360^	Fraca	Baixa
A tomografia computadorizada com SPECT, pode agregar informações a respeito da microcirculação pelo uso do radiotraçador 99mTc-sestamibi.^360^	Fraca	Baixa

DF: doença de Anderson-Fabry; ETT: ecocardiograma transtorácico; RMC: ressonância magnética cardíaca; SLG: *strain* longitudinal global; SPECT: single-photon emission computed tomography.

A RMC também desempenha um papel crucial na avaliação da resposta à TRE ou à terapia com chaperonas moleculares. Estudos longitudinais demonstraram que o tratamento eficaz pode levar à estabilização ou mesmo regressão da hipertrofia ventricular, além de prevenir a progressão da fibrose miocárdica.^362^ A RMC permite monitorar essas mudanças de forma objetiva e reprodutível, fornecendo parâmetros quantitativos que auxiliam na tomada de decisões terapêuticas. O mapeamento T1, em particular, mostrou-se sensível às alterações no conteúdo miocárdico de globotriaosilceramida sob tratamento, podendo servir como um biomarcador precoce de resposta terapêutica. Além da avaliação morfológica e da caracterização tecidual, a RMC fornece informações valiosas sobre a função cardíaca e o prognóstico^356,362^ (Tabela 20).

A TC com SPECT, apesar de ainda ter seu papel diagnóstico e prognóstico em investigação, também pode agregar informações a respeito da microcirculação pelo uso do radiotraçador 99mTc-sestamibi. É frequente a queixa de dor torácica de característica isquêmica associada a alterações eletrocardiográficas e de biomarcadores típicas na ausência de obstrução coronariana macrovascular. A RFC pode também ser avaliada nesses casos, pela tomografia ou por RMC.^360,361^

Na Tabela 20, são descritas as principais recomendações sobre a avaliação MM em imagem cardíaca na DF no formato GRADE.

A periodicidade recomendada para exames de imagem cardíaca em pacientes com DF é baseada em consenso de especialistas e práticas de centros de referência. Em publicação da Universidade de Washington, é recomendada a ecocardiograma anual em homens a partir dos 18 anos e a cada 2 anos em mulheres dos 18 aos 35 anos; após os 35 anos, a frequência pode ser ajustada conforme o quadro clínico.^350^

Estudos multicêntricos e revisões sugerem que, na prática clínica, ecocardiografia (incluindo *strain* miocárdico) deve ser realizada a cada 6 a 12 meses, especialmente em pacientes com maior risco ou sintomas, podendo ser antecipada em caso de piora clínica.^363^ A RMC é recomendada a cada 1 a 2 anos, conforme disponibilidade e estágio da doença.^335,362,363^

Assim, a periodicidade dos exames deve ser individualizada conforme gravidade, sintomas, resposta ao tratamento e disponibilidade dos métodos, com intensificação do seguimento em casos de maior risco ou progressão de lesão cardíaca.^363,364^

**Mensagens-chave**

- Os achados ecocardiográficos mais típicos da DF são o aumento da espessura miocárdica da parede ventricular e da musculatura papilar com função sistólica preservada, DD, sinal binário do endocárdio e refluxos valvares discretos.
- O SLG reduzido em segmento basal da parede lateral inferior é característico da DF e auxilia o diagnóstico diferencial com outras doenças de fenótipo semelhante.
- A RMC é útil para distinguir a hipertrofia ventricular da DF das outras cardiomiopatias, devido ao espessamento de padrão concêntrico simétrico, fibrose em segmentos basais inferolaterais e aos valores tipicamente reduzidos de T1 nativo em parede septal.
- A RMC permite avaliar impacto da terapia da DF com reposição enzimática ou com chaperonas moleculares, acompanhando a regressão da hipertrofia e a estabilização do RT.

### 5.3. Doenças de Depósito do Glicogênio

As doenças de depósito do glicogênio (DDGs) representam um grupo heterogêneo de doenças metabólicas genéticas, causadas por mutações em genes que codificam enzimas envolvidas no metabolismo da glicose, levando a acúmulo intracelular de glicogênio em vários órgãos. Existem mais de 12 tipos de DDGs, entre as quais, destacamos as que afetam o coração e o músculo esquelético: doença de Pompe (tipo IIa) (autossômica recessiva com variantes patogênicas no gene que codifica o ácido a-glicosidase [GAA]),^365^ doença de Danon (DDG tipo IIb) (herança ligada ao X e mutações no gene *LAMP2*, que codificam a proteína da membrana associada ao lisossomo 2)^366^ e doença PRKAG (autossômica dominante com mutações na *PRKAG2* [subunidade reguladora gama-2 da proteína quinase ativada por AMP]).^367^

As DDGs possuem diferentes fenótipos cardíacos, que variam desde uma condição assintomática até a morte súbita cardíaca. Apesar de uma ampla apresentação clínica, essas doenças se manifestam predominantemente em crianças (já no primeiro ano de vida, no caso da doença de Pompe) ou em adultos jovens e têm em comum um aumento precoce da espessura das paredes do VE.

**Figura 24:**
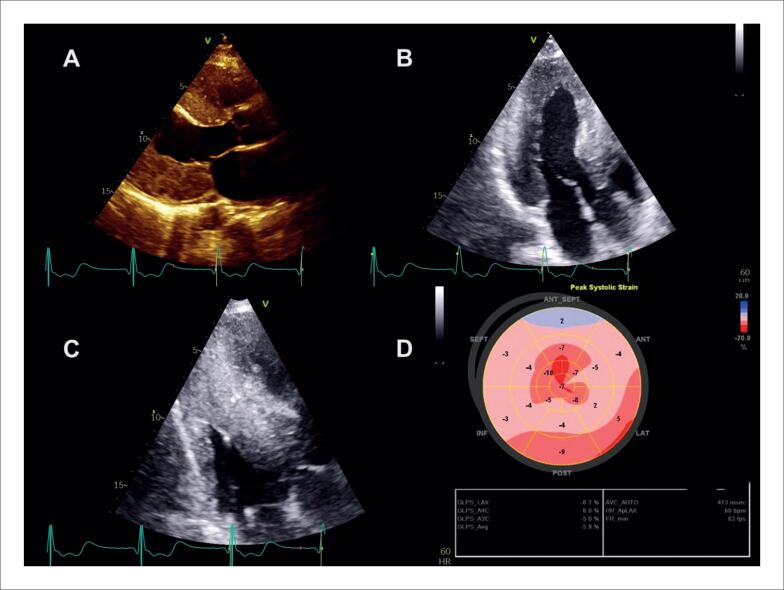
Imagens ao ecocardiograma transtorácico de paciente portador de síndrome do PRKAG2. Observa-se hipertrofia significativa concêntrica do ventrículo esquerdo ao corte paraesternal longitudinal (A) e corte apical longitudinal (B), além de hipertrofia importante ventricular direita em corte apical focado em cavidades direitas (C). Representação paramétrica em bull's-eye de paciente com comprometimento moderado da função sistólica do ventrículo esquerdo, demonstrando redução do strain longitudinal global (-5,9%) e aspecto em listras.

O ecocardiograma é o método de imagem de primeira linha e pode demonstrar, além de um fenótipo hipertrófico, um fenótipo restritivo ou dilatado.^366,368,369^ A hipertrofia é, muitas vezes, grave (> 30 mm), biventricular e rapidamente progressiva, especialmente em indivíduos com doença de Danon do sexo masculino^366^ (Figura 24). Indivíduos do sexo feminino com doença de Danon apresentam hipertrofia do VE assimétrica, hipertrofia ventricular direita ou, em 30 a 50%, fenótipos dilatados ou hipocinéticos não dilatados, de evolução mais lenta. A obstrução dinâmica na via de saída do VE pode estar presente nas DDGs, especialmente na doença de Danon (em até 25% dos casos),^366^ e deve ser ativamente pesquisada em pacientes com dispneia.

Apesar do papel fundamental do ecocardiograma, o contexto clínico é determinante para o diagnóstico diferencial com outras fenocópias hipertróficas, principalmente a CMH sarcomérica, auxiliando a indicação de testes genéticos e/ou biópsia.^368^ Alterações eletrocardiográficas, como intervalo PR curto, pré-excitação ventricular (síndrome de Wolff-Parkinson-White), fibrilação atrial, atrasos na condução intraventricular ou bloqueios sinoatriais ou atrioventriculares, com necessidade precoce de marca-passo, são consideradas sinais de alerta para o diagnóstico de DDG.^366^ Ao contrário das cardiomiopatias infiltrativas, a voltagem do QRS é normal ou aumentada, o que auxilia o diagnóstico diferencial com amiloidose. O envolvimento multissistêmico (principalmente miopatias e alterações neurocognitivas) faz parte da tríade clássica da doença de Danon (aliados à cardiopatia), apesar de ocorrer mais raramente em outras DDGs.^366^ O padrão de hipertrofia ventricular apresenta particularidades que podem ser identificadas pela RMC, conforme discutido a seguir.

Técnicas ecocardiográficas avançadas, incluindo imagens de deformação miocárdica por *speckle tracking* e 3D, são recomendáveis em outros fenótipos hipertróficos ou restritivos,^5,105^ mas carecem de validação robusta nas DDGs. O SLG reduzido com preservação dos segmentos apicais (padrão *apical sparing*, similar ao da AC) tem sido descrito em pequenas séries retrospectivas de casos de doença de Danon, em que foi associado a piores desfechos.^370,371^

Pena et al., ao analisarem 30 pacientes com *PRKAG2*, observaram, em 60% dos pacientes, um padrão regional peculiar no mapa paramétrico do SLG (Figura 24), variando de rosa claro a rosa escuro, com formato de listras.^372^ Nessa mesma série de pacientes, os autores demonstraram que a função do VD, avaliada pelo SLG e pelo ecocardiograma 3D, estava abaixo dos limites normais em mais da metade dos pacientes, confirmando uma correlação positiva e estatisticamente significativa, entre o *strain* longitudinal de parede livre do ventrículo direito (SLPLVD) e a FEVD. Esses parâmetros demonstraram maior sensibilidade para detectar disfunção miocárdica do que os índices ecocardiográficos tradicionais, como TAPSE.^373^ Em outro estudo do mesmo grupo, com sete crianças filhas de portadores dessa síndrome, Santos Neto et al. demonstraram achados ecocardiográficos indicativos de tendência à hipertrofia cardíaca.^374^

A Tabela 21 resume as principais características ecocardiográficas das DDGs abordadas.

**Tabela 21:** Principais características ecocardiográficas das DDGs

**Doença de Danon**	Hipertrofia biventricular rapidamente progressiva e de grau importante (homens) ou fenótipo variável, de hipertrofia à dilatação e disfunção sistólica (em mulheres). Em 25%, pode haver obstrução da VSVE, que deve ser ativamente pesquisada em pacientes com dispneia. O *strain* pode apresentar mapa polar com padrão *apical sparing* similar ao da amiloidose.
**Doença de Pompe**	Hipertrofia concêntrica com evolução rápida para hipocinesia difusa grave, se não houver intervenção. Pode haver obstrução da VSVE.
***PRKAG2***	HVE em diferentes graus, com padrão concêntrico na maioria das vezes; a hipertrofia do VD não é rara; a obstrução da VSVE é infrequente. O *strain* pode ter mapa polar com padrão em listras.

DDG: doença de depósito do glicogênio; HVE: hipertrofia ventricular esquerda; VD: ventrículo direito; VSVE: via de saída ventricular esquerda.

O prognóstico das fenocópias hipertróficas associadas a DDGs é geralmente pior do que o da CMH causada por variantes do gene da proteína sarcomérica. Dessas, apenas a doença de Pompe apresenta TRE,^365^ mas o diagnóstico precoce auxilia nas terapias para IC e arritmias. Porém, o benefício do controle da TRE na doença de Pompe ainda é incerto. Considerando a gravidade e rapidez de progressão das DDGs e as implicações terapêuticas, é razoável recomendar, mesmo sem evidências robustas, que crianças e adolescentes sintomáticos ou assintomáticos, com variantes patogênicas e/ou filhos de familiares com início precoce dos sinais e sintomas de DDGs realizem ecocardiograma, para rastreio da doença, a cada 1 a 2 anos. Indivíduos sem as condições descritas anteriormente podem realizar o ecocardiograma a cada 3 a 5 anos.^366^

A RMC permite a detecção de alterações miocárdicas características que podem distinguir as DDGs de outras formas de CMH, permitindo não apenas o diagnóstico precoce, mas também a monitorização da progressão da doença.^374^ Isso é particularmente valioso, considerando a natureza rapidamente progressiva de algumas dessas doenças (como a doença de Danon e a doença de Pompe infantil) e a necessidade de intervenções precoces. O padrão de RT, mapeamento T1 nativo e o cálculo do VEC auxiliam no diagnóstico diferencial com outros fenótipos hipertróficos bem como na avaliação do estágio da doença (Tabela 22). Na doença de Danon, a extensão do RT correlaciona-se com o grau de disfunção cardíaca e tem importante valor prognóstico, sendo um marcador de risco para arritmias ventriculares malignas e morte súbita cardíaca. O RT frequentemente apresenta distribuição transmural ou subepicárdica e envolve múltiplos segmentos ventriculares. A RMC também permite uma quantificação mais acurada da massa ventricular e da espessura da parede, bem como a detecção de trombos intracavitários secundários à disfunção ventricular, superando algumas limitações da ecocardiografia no que diz respeito à avaliação de segmentos cardíacos com maior dificuldade de visibilização, tais como a região apical, parede lateral e VD. Estudos demonstraram que a progressão da hipertrofia na doença de Danon ocorre de forma mais acelerada do que em outras cardiomiopatias, e a RMC fornece um método sensível para monitorar essa evolução. Os principais achados na RMC em pacientes com DDG estão descritos na Tabela 22.

**Tabela 22:** Principais alterações das doenças sistêmicas de DDG pela RMC

	Morfologia	Realce tardio	Mapa de T1	T2 ou T2*	VEC
**Normal**	Normal	Ausente	< 1050 ms	> 40 ms	< 40%
**Doença de Danon**	HVE simétrica acentuada associada a disfunção do VE	Transmural ou subepicárdica difusa	Elevado ou normal	N/A	Elevado ou normal
**Doença de Pompe**	HVE acentuada na apresentação precoce	Raro, mesocárdico inferolateral	Elevado ou normal	N/A	Elevado ou normal
***PRKAG2***	HVE difusa ou septal	Raro nos estágios iniciais	Reduzido nos estágios iniciais	N/A	N/A

DDG: doença de depósito de glicogênio; HVE: hipertrofia ventricular esquerda; N/A: não se aplica; RMC: ressonância, magnética cardíaca; T1: Recuperação do relaxamento longitudinal, T2: Decaimento do relaxamento transversal; VE: ventrículo esquerdo; VEC: volume extracelular.

No contexto do aconselhamento familiar e triagem de portadores, a RMC oferece uma ferramenta sensível para detectar envolvimento cardíaco em parentes em risco, mesmo na ausência de sintomas clínicos. Considerando o padrão de herança ligado ao X, a identificação precoce de alterações cardíacas em mulheres heterozigotas é particularmente importante, já que elas podem desenvolver manifestações cardíacas mais tardiamente do que os homens afetados, mas com igual gravidade.

Na Tabela 23, são descritas as principais recomendações sobre a avaliação MM em imagem cardíaca nas DDG no formato GRADE.

**Tabela 23:** Recomendações sobre o uso da multimodalidade de imagem em pacientes com DDG suspeita ou confirmada

Recomendação	Força da recomendação	Certeza da evidência
O ETT deve ser realizado como método de imagem de primeira linha na investigação de DDG suspeita ou conhecida.^105^	Fraca	Moderada
O uso de ARU deve ser considerado em pacientes com DDG e janela acústica limitada, para melhorar o delineamento das bordas endocárdicas e opacificação ventricular, aumentando a detecção de hipertrofia ou trombos e a acurácia na medida da FEVE.^105^	Fraca	Baixa
O *strain* longitudinal global do VE pode ser utilizado, em pacientes com janelas acústicas adequadas, como ferramenta adicional para detecção de disfunção sistólica subclínica do VE e do VD em portadores de DDG, e o padrão do mapa polar pode ajudar o diagnóstico diferencial com outras fenocópias.^370–372^	Fraca	Baixa
ETT 3D pode ser utilizado para avaliação mais acurada da FEVE em pacientes com DDG e janela acústica adequada.^105^	Fraca	Baixa
ETE deve ser indicado no caso de eventos neurológicos agudos em pacientes com DDG, para a detecção de trombos intracardíacos, no contexto de disfunção ventricular esquerda ou arritmias.^105^	Fraca	Moderada
ETT com manobras provocativas (Valsalva, ortostase) é recomendável no paciente dispneico, com hipertrofia do VE, para pesquisa de obstrução dinâmica da VSVE, que não apresente gradiente da VSVE ≥ 50 mm Hg em repouso.^366,368^	Fraca	Moderada
Ecocardiograma sob esforço é recomendável no paciente dispneico, com hipertrofia do VE, para pesquisa de obstrução dinâmica da VSVE, que não apresente gradiente da VSVE ≥ 50 mm Hg em repouso ou manobra provocativa.^366,368^	Fraca	Moderada
ETT deve ser usado para a triagem de familiares, no momento do diagnóstico de familiares de primeiro grau, quando o teste genético não for possível.^366^	Fraca	Moderada
A RMC deve ser considerada na investigação das DDGs por permitir melhor caracterização tecidual, diferenciar o fenótipo, fornecer informações prognósticas e eficácia da resposta terapêutica.^365,374^	Fraca	Moderada

ARU: agentes de realce ultrassonográfico; DDG: doença de depósito de glicogênio; ETE: ecocardiograma transesofágico; ETT: ecocardiograma transtorácico; FEVE: fração de ejeção do ventrículo esquerdo; RMC: ressonância magnética cardíaca; VD: ventrículo direito; VE: ventrículo esquerdo; VSVE: via de saída ventricular esquerda.

O painel de especialistas desde posicionamento recomenda a realização de ETT para acompanhamento clínico de pacientes com diagnóstico de DDG e rastreio de acometimento cardíaco a cada 1 a 2 anos, em crianças e adolescentes sintomáticos ou assintomáticos, com variantes patogênicas e/ou filhos de familiares com início precoce dos sinais e sintomas de DDGs. A RMC pode ser realizada a cada 1 ou 2 anos em pacientes sintomáticos, alterações ecocardiográficas e fatores de risco como arritmias, IC e complicações clínicas.^184,375^

**Mensagens-chave**

- As DDGs se manifestam, tipicamente, por HVE ou biventricular ao ecocardiograma em fases precoces de vida.
- Diante de um ecocardiograma com HVE ou biventricular, o contexto clínico é fundamental para o diagnóstico diferencial, em especial, a idade de início, o modo de herança, associação com pré-excitação ventricular e envolvimento multissistêmico.
- O uso de técnicas avançadas em ecocardiografia, como SLG e 3D, é promissor para a deteção precoce de disfunção ventricular esquerda ou biventricular, em pacientes com DDG suspeita ou conhecida. O padrão do mapa polar do *strain* pode auxiliar no diagnóstico diferencial com outras fenocópias hipertróficas.
- A RMC auxilia na distinção das DDGs de outras cardiomiopatias pela presença, extensão de distribuição da HVE, valores de T1 nativo e VEC associados à dilatação e à perda de função sistólica ventricular precoce.

## 6. Aplicações da Multimodalidade em Imagem Cardíaca nas Doenças Neuromusculares

Denominam-se doenças neuromusculares (DNMs) um grupo heterogêneo de condições raras que afetam, de forma degenerativa, a função motora, respiratória ou mesmo de deglutição. As principais condições têm origem genética, como a distrofia muscular de Duchenne (DMD), a distrofia de Becker e ataxia de Friedreich (AF). As duas primeiras doenças são condições genéticas ligadas ao cromossomo X, cursando com alterações da proteína distrofina em maior ou menor grau. O comprometimento pode envolver a função cardíaca, a qual se constitui importante causa de mortalidade nessas populações. Não há, em nenhuma delas, clara correlação temporal entre o envolvimento de musculatura esquelética e miocárdica, o que constitui um desafio maior para a determinação do rastreio da disfunção cardíaca. A avaliação de função miocárdica por métodos de imagem não invasivos, além de suas capacidades e indicações, será abordada neste texto.

### 6.1. Distrofia Muscular de Duchenne

A DMD é a forma mais grave das distrofinopatias, levando ao progressivo comprometimento de função motora, em geral, com dependência de cadeira de rodas geralmente antes dos 12 anos de idade.^376^ A cardiomiopatia, embora comum, geralmente se desenvolve mais tarde no curso da doença, com padrão de cardiomiopatia dilatada, evoluindo com redução da FEVE por volta dos 14 anos.^377^ Cerca de 1/3 dos pacientes terão cardiomiopatia dilatada aos 14 anos, 50% entre 14 e 18 anos e provavelmente todos após os 18 anos.^378^ Embora ainda haja escassez de estudos clínicos com evidências robustas, o diagnóstico precoce do envolvimento cardíaco pode possibilitar a instituição terapêutica cardioprotetora precoce (com inibidores da enzima de conversão da angiotensina [IECA] ou betabloqueadores).^378^ Um estudo retrospectivo com 69 pacientes demonstrou que a instituição de cardioprotetores permitiu a estabilização ou mesmo melhora da função miocárdica em porcentagem significativa dos pacientes acompanhados por período inferior a 4 anos.^379^

**Figura 25:**
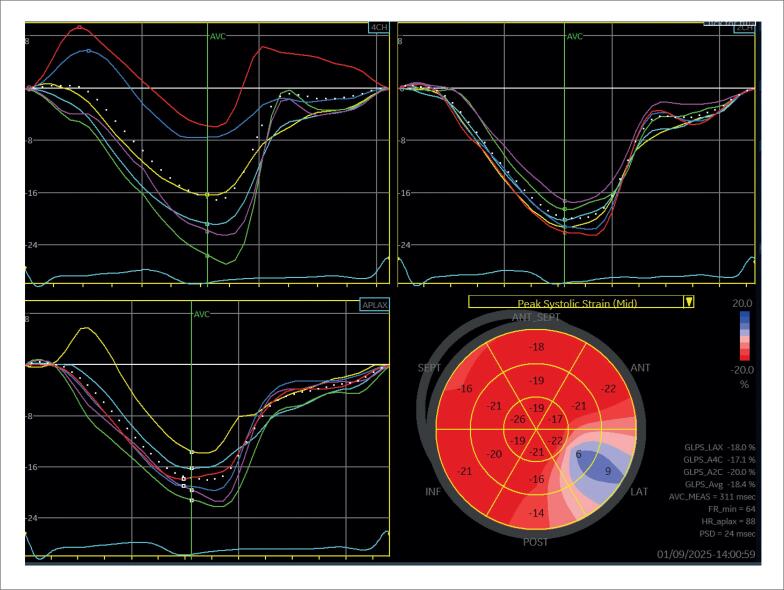
Imagem de gráfico bull's-eye e das curvas de cada segmento miocárdico nas projeções apicais de medidas de deformação miocárdica longitudinal (strain longitudinal global) em paciente com distrofia muscular de Duchenne demonstrando redução da deformação em segmentos póstero-laterais, apesar de contratilidade segmentar ventricular esquerda preservada.

O ETT é a modalidade de escolha para a avaliação e acompanhamento dos pacientes com DMD, considerando o acesso e custo. Atualmente, recomenda-se a avaliação no momento do diagnóstico da DMD ou a partir dos 6 anos de idade, com avaliações a cada 2 anos até os 10 anos, quando, então, recomenda-se a avaliação anual.^380,381^O padrão de acometimento miocárdico pode se iniciar de forma regional, principalmente na parede inferolateral do VE (Figura 25). O apoio de técnicas ecocardiográficas avançadas para a avaliação de função ventricular, tal como a análise de *strain* por *speckle tracking* em vários eixos, como os parâmetros globais SLG e *strain* circunferencial global (SCG), tem se mostrado mais sensível para o diagnóstico precoce do envolvimento miocárdico, quando comparada a voluntários normais de mesma idade.^382,383^ Trabalhos iniciais apontam para o poder do *strain* regional em identificar um comprometimento de deformação miocárdica inicialmente na região inferolateral do VE.^384^

Estudos comparando a RMC com o ETT em pacientes com DMD demonstraram que 20 a 40% dos pacientes são subdiagnosticados pelo ETT ao usar o critério da FEVE.^385,386^ Além disso, são frequentes os relatos de janela acústica limitada nessa população.^387^ Prakash et al., em 2022, mostraram em uma coorte de 38 pacientes com DMD, em que 52,6% tinham FEVE < 55% pela RMC, e apenas 12% tinham SLG anormal.^388^ Um posicionamento publicado em 2018 sugere o uso do ETT até por volta dos 6 aos 7 anos em pacientes com DMD e, assim que possível, realizar RMC para detecção precoce de cardiopatia.^389^ É preciso considerar que a RMC é um método de maior custo e menor disponibilidade e que ainda requer frequente sedação em pacientes pediátricos ou com dificuldades de posicionamento, que podem ser comuns nessa população.

### 6.2. Distrofia Muscular de Becker

**Figura 26:**
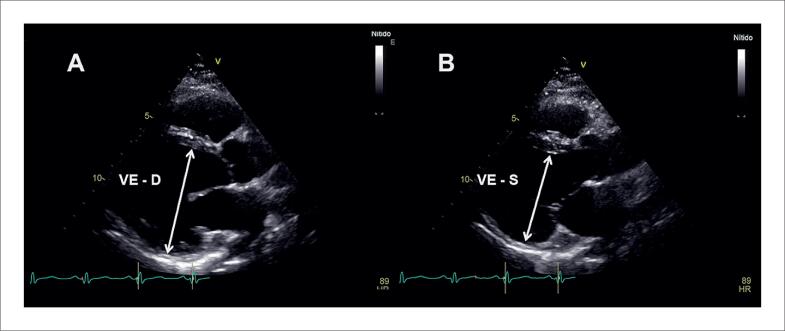
Imagens ecocardiográficas de paciente com diagnóstico de distrofia muscular de Becker, evidenciando discreta dilatação ventricular esquerda ao corte paraesternal longitudinal e pouca variação entre os diâmetros diastólico (A) e sistólico (B), com disfunção sistólica de grau moderado. Observa-se, na sístole, ausência de espessamento da parede lateral inferior (B).

Ao contrário da forma mais grave, a DMD, a distrofia muscular de Becker (DMB) apresenta evolução mais lenta e sintomas geralmente mais brandos.^377^ Estudos histopatológicos mostram que a lesão cardíaca pode preceder os sintomas clínicos, iniciando-se ainda na adolescência, mesmo em indivíduos assintomáticos ou com pouca limitação motora.^390^ O padrão de acometimento miocárdico geralmente envolve a parede inferolateral do VE, assim como na DMD (Figuras 26 e 27). Recomenda-se iniciar o seguimento cardiológico já na infância ou adolescência, com avaliações periódicas mesmo em indivíduos assintomáticos, com sugestão de periodicidade anual. A detecção de alterações funcionais precoces que indiquem a necessidade de intervenção terapêutica ainda representa um desafio clínico, no qual a ecocardiografia tem papel crescente.

**Figura 27:**
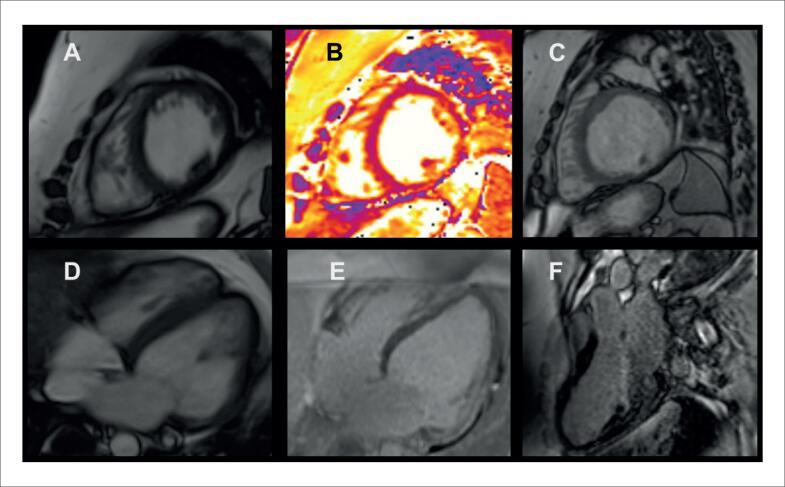
Imagens de ressonância magnética cardíaca de paciente com distrofia muscular de Becker. Observa-se dilatação ventricular esquerda com disfunção sistólica importante do ventrículo esquerdo. Observa-se afilamento dos segmentos basais e médios das paredes inferior e inferolateral (A a F). Observa-se extenso realce tardio heterogêneo nos segmentos basais e médios das paredes lateral anterior e inferolateral mediobasais (C, E e F), com aspecto habitualmente encontrado em acometimento pela distrofia muscular.

A análise de função cardíaca deve ser baseada também na quantificação da FEVE, mas também já há evidências do papel de técnicas avançadas como o s*train* miocárdico avaliada pela técnica de *speckle tracking* como ferramenta que permite o diagnóstico precoce da disfunção miocárdica global ou regional. Em um estudo observacional,^391^ 77,5% dos pacientes com DMB apresentaram SLG anormal (≥ −18%), enquanto apenas 60% tinham FEVE reduzida. Notavelmente, 17,5% apresentavam SLG alterado mesmo com FEVE normal. Após 2 anos de seguimento, observou-se deterioração significativa do SLG, enquanto a FEVE permaneceu estável, demonstrando o papel da análise do *strain* como um marcador precoce e sensível da progressão da doença. O SLG também apresentou correlação significativa com troponina I e NT-proBNP, mas não com medidas de função muscular ou respiratória. Ainda, o Doppler tecidual e a avaliação da função atrial esquerda, embora menos explorados na DMB, podem fornecer informações adicionais sobre DD precoce, sobretudo quando associados à análise de *strain* atrial. O reconhecimento precoce dessas alterações é essencial para a instituição de terapias modificadoras da doença, como o uso de cardioprotetores, que podem ter impacto na evolução da doença e morbimortalidade.

A RMC é a ferramenta diagnóstica de escolha na avaliação das alterações cardíacas como edema e fibrose miocárdica. As diferentes técnicas, como sequência de T1 e T2, técnica do RT e mapas multiparamétricos, são fundamentais na avaliação desses pacientes, além da avaliação contrátil e do cálculo dos volumes e fração de ejeção de ambos os ventrículos. Em estudos recentes, a presença de RT aumenta conforme a idade (de 17% abaixo dos 10 anos para 59% naqueles pacientes com mais de 15 anos de idade com redução da FEVE).^392^ Há correlação linear entre a presença e o número de segmentos comprometidos por fibrose e a queda da FEVE. Tipicamente o padrão de fibrose visualizado na DMD e na DMB compromete o subepicárdio ou mesocárdio, progredindo com a evolução da doença para fibrose transmural mais frequentemente na parede inferolateral do VE.^393^ Estudos recentes têm proposto que a conhecida infiltração de tecido adiposo no músculo esquelético desempenha papel importante na progressão da doença miocárdica e que talvez o RT visualizado no miocárdio seja resultado da combinação de fibrose e tecido adiposo.^394^ Portanto, a RMC apresenta papel fundamental não somente na avaliação da presença da fibrose e função ventricular, mas também na avaliação da evolução a nível celular com transformação do tecido adiposo no interior do músculo cardíaco.

### 6.3. Ataxia de Friedreich

A Ataxia de Friedreich (AF) é uma doença neurodegenerativa de herança autossômica recessiva, causada por uma expansão de repetições no gene *FXN*, que resulta em deficiência da proteína frataxina. Clinicamente, é caracterizada por ataxia progressiva, disfunção cerebelar, escoliose, DM e, frequentemente, cardiomiopatia, que é uma das manifestações mais graves e letais da doença.^376,395^

A cardiomiopatia associada à AF (CMP-AF) é marcada por HVE, principalmente septal, que pode progredir para fibrose, disfunção ventricular, arritmias e IC, sendo esta última a principal causa de óbito entre os pacientes. A relação entre CMP-AF e degeneração neurológica não é clara, mas a avaliação regular do envolvimento cardíaco é essencial na prática clínica. A CMP-AF pode ser classificada em quatro categorias com base na espessura do septo interventricular na diástole (SIVd) e na FEVE como observada na Tabela 24. No entanto, com a progressão da doença, pode ocorrer uma redução da massa de VE devido à regressão da HVE e aumento da fibrose miocárdica. Esses parâmetros podem ser avaliados pela ecocardiografia bidimensional seriada. A estimativa da FEVE é um parâmetro crucial, e sua queda está associada a maior risco de mortalidade.^396^

**Tabela 24:** Classificação da cardiomiopatia relacionada à ataxia de Friedreich

Classificação	Critério diagnóstico	Características
CMP-AF Discreta	SIVd aumentado* (aumento ≤ 18% do predito) e FEVE ≥ 50%	HVE discreta e função sistólica preservada
CMP-AF Moderada	SIVd aumentado* (aumento ≥ 18% do predito) e FEVE ≥ 50%	HVE moderada, possível início de remodelamento
CMP-AF Grave	FEVE ≤ 50% (independente da SIVd)	Dilatação ventricular, menor SLG miocárdico, possíveis sinais de fibrose

* A espessura do SIVd considerada normal baseado no normograma de Henry et al.^397^ CMP-AF: cardiomiopatia associada à ataxia de Friedreich; HVE: hipertrofia ventricular esquerda; FEVE: fração de ejeção do ventrículo esquerdo; SIVd: espessura do septo interventricular na diástole; SLG: *strain* longitudinal global.

O uso de técnicas ecocardiográficas avançadas como a análise do SLG do VE pode demonstrar alterações precoces em relação à FEVE, sendo útil para a detecção da disfunção subclínica. A DD é a alteração mais comum (84%) com pseudonormalização predominante e pode ser avaliada através da relação E/A do fluxo transmitral, tempo de desaceleração do fluxo mitral e relação E/e’.^398^

Diante da escassez de ensaios clínicos controlados e de revisões sistemáticas direcionadas ao diagnóstico da CMP-AF, sugerimos a avaliação sistemática dos parâmetros ao ETT listados na Tabela 25.

**Tabela 25:** Principais parâmetros ecocardiográficos na avaliação cardíaca da ataxia de Friedreich

Parâmetro ecocardiográfico	Anormalidade cardíaca associada
SIVd	Avaliação da hipertrofia concêntrica
Espessura da parede posterior	Avaliação da hipertrofia e geometria
Espessura relativa da parede	Remodelamento concêntrico
Índice de massa ventricular esquerda	Hipertrofia ventricular e preditor de mortalidade
FEVE	Função sistólica global e preditor de sobrevida
SLG	Disfunção subclínica precoce do miocárdio
Relação E/A mitral	Função diastólica (pseudonormalização comum)
Relação E/e'	Pressão de enchimento ventricular
Tempo de relaxamento isovolumétrico	Disfunção diastólica precoce

FEVE: fração de ejeção do ventrículo esquerdo; SIVd: espessura do septo interventricular no final da diástole; SLG: *Strain* global longitudinal.

A RMC é uma ferramenta valiosa para caracterizar o remodelamento miocárdico e a fibrose em pacientes com AF, mesmo na ausência de IC clínica, podendo detectar HVE e fibrose miocárdica. ^399^ Além disso, a RMC pode revelar remodelamento miocárdico precoce, caracterizado por hipertrofia e aumento do VEC, mesmo em pacientes com FEVE preservada.^399^ A técnica permite também detectar anormalidades na reserva de perfusão miocárdica, que são indicativas de isquemia miocárdica na ausência de DAC epicárdica.^400^

Assim, para pacientes com suspeita de CMP relacionadas à DNM, com base em pesquisa de evidências em literatura, principalmente das distrofias de Duchenne e Becker e AF, este documento recomenda a favor da adoção de uma estratégia de rastreamento de CMP através do ETT, considerando a acessibilidade e os custos. Com base em critérios da metodologia GRADE,^401^ a recomendação é forte, baseada em um nível de certeza moderado (principalmente relacionado a Duchenne) da evidência científica para o diagnóstico precoce, com possível impacto benéfico na evolução da disfunção miocárdica (nível de certeza ainda baixo sobre o impacto em sobrevida (Tabela 26).

**Tabela 26:** Recomendações para o uso do ecocardiograma em pacientes com doenças degenerativas neuromusculares

Recomendação	Força da recomendação	Certeza da evidência
O ecocardiograma transtorácico deve ser realizado como primeira linha na avaliação de pacientes com doenças neuromusculares para o rastreio de disfunção ventricular.	Forte	Moderada

Em pacientes com DNM, recomenda-se a avaliação no momento do diagnóstico ou a partir dos 6 anos de idade, com avaliações a cada 2 anos até os 10 anos, quando recomenda-se avaliação anual. Em pacientes com evidência de redução da FEVE ou na presença de alterações detectadas pelo SLG, recomenda-se a avaliação anual.^380,381^

**Mensagens-chave**

- As DNMs como a DMD, DMB e AF podem apresentar comprometimento da função cardíaca, a qual constitui-se importante causa de mortalidade nestas populações.
- A DMD é a forma mais grave das distrofinopatias. A cardiomiopatia é frequente e apresenta padrão de cardiomiopatia dilatada, evoluindo com redução da FEVE por volta dos 14 anos.
- O ETT é a modalidade de escolha para a avaliação e acompanhamento dos pacientes com DNM.
- O padrão de acometimento miocárdico na DMD e na DMB pode iniciar-se de forma regional, principalmente na parede inferolateral do VE.
- O SLG do VE tem se mostrado mais sensível para o diagnóstico precoce do envolvimento miocárdico nas DNMs.
- A cardiomiopatia associada à AF é marcada por HVE, principalmente septal, que pode progredir para fibrose, disfunção ventricular, arritmias e IC.
- Em casos avançados de doença cardíaca na AF, pode ocorrer regressão da HVE e aumento da fibrose miocárdica. A análise da FEVE pelo ETT é um parâmetro crucial, e sua queda está associada a maior risco de mortalidade.

## 7. Perspectivas

A integração MM das técnicas de imagem, não apenas em cardiologia, mas em todas as especialidades médicas, é uma necessidade e uma realidade para os pacientes. Cada método em imagem cardíaca possui suas vantagens e desvantagens, e não há um método que seja capaz de, isoladamente, fornecer todas as informações sobre o complexo funcionamento do sistema cardiovascular.

Dadas as particularidades e à interface entre fatores inflamatórios e os tradicionais fatores de risco cardiovascular em cada doença específica, as recomendações para a solicitação e frequência de cada exame de imagem cardíaca devem ser realizadas de maneira individualizada para cada doença e paciente. O racional para a solicitação dos exames consiste na redução do risco das complicações cardiometabólicas e inflamatórias dos fatores de risco tradicionais, além do diagnóstico precoce de alterações que incluem IC (com fração preservada ou reduzida), doenças coronárias, valvares, miocárdicas, pericárdicas e aórticas associadas às doenças sistêmicas. Assim, dada a complexidade de cada doença e de suas complicações cardíacas, é necessária de maneira crítica a adoção de uma abordagem estruturada envolvendo a integração MM para o diagnóstico, prognóstico e monitoramento dessas anormalidades.^4,9–11,402,403^

Com relação às novas tecnologias, apesar de seu valor bem estabelecido na detecção precoce de alterações subclínicas da função cardíaca, a análise do *strain* miocárdico pela ecocardiografia ainda não é adotada de maneira rotineira. Essa análise depende de equipamentos específicos, além de treinamento do examinador, exige maior tempo de exame e, assim, necessita de remuneração adequada, limitando que essa técnica seja incorporada à prática clínica. Entretanto, seria importante que a análise do *strain* miocárdico fosse integrada de maneira sistemática à analise ecocardiográfica convencional.^5,6^ Novas técnicas de análise da reserva de fluxo fracionada e também de perfusão associada à TCC para avaliação de isquemia miocárdica podem melhorar a acurácia diagnóstica e persistem subutilizadas.^9,10,403^ Já a RMC 4D permite a visualização tridimensional do fluxo vascular ao longo do tempo e medidas quantitativas dos fluxos transvalvares e intracavitários.^11,403^

De maneira complementar, a integração entre medicina de precisão e inteligência artificial (IA) na prática cardiovascular tem transformado a avaliação e o manejo de doenças sistêmicas com envolvimento cardíaco.^404,405^ A medicina de precisão busca adaptar o diagnóstico e o tratamento com base em características biológicas, clínicas e de imagem específicas de cada paciente. A IA potencializa essa abordagem ao processar grandes volumes de dados, identificar padrões sutis e prever trajetórias de doença com maior acurácia. Quando aplicada à imagem cardíaca multimodal, a IA é capaz de integrar informações funcionais, estruturais e de caracterização tecidual, promovendo uma estratificação fenotípica mais precisa e auxiliando na detecção precoce do acometimento miocárdico.^406,407^

Nas doenças sistêmicas, a caracterização precoce e acurada das alterações cardíacas é fundamental para a estratificação de risco e planejamento terapêutico. Em doenças como a amiloidose ou sarcoidose, por exemplo, a combinação entre RMC e PET ou SPECT aumenta a sensibilidade diagnóstica e pode identificar inflamação ou infiltração miocárdica subclínica. De modo semelhante, em doenças reumáticas autoimunes, a integração entre ecocardiografia, medicina nuclear e RMC permite a detecção de sinais iniciais de isquemia, miocardite ou fibrose, que frequentemente passam despercebidos nos estágios iniciais.

Assim, a convergência entre medicina de precisão, IA e imagem cardíaca multimodal promove uma compreensão mais profunda do impacto cardiovascular das doenças sistêmicas, permitindo rastreio e diagnóstico precoce das complicações cardiovasculares nessas doenças, além do monitoramento da função cardíaca e alterações associadas no seguimento dos pacientes com doenças sistêmicas generalizadas para uma abordagem personalizada.

## 8. Conclusões

A ampla variedade de manifestações cardiovasculares nas doenças sistêmicas torna necessária a padronização de rotina da solicitação MM em imagem cardíaca no seguimento clínico desses pacientes. Entretanto, até o momento, não há uma rotina estabelecida de solicitação de exames de imagem cardíaca nesses pacientes. O avanço das técnicas em imagem cardíaca, aliado às evidências crescentes de sua utilidade no cuidado a pacientes com doenças sistêmicas, além do trabalho multidisciplinar entre cardiologistas, reumatologistas, pneumologistas, neurologistas, entre outros, são essenciais para viabilizar a criação de uma rotina padronizada de uso da imagem por MM nesses casos.

## Data Availability

os conteúdos subjacentes ao texto do Posicionamento estão contidos no manuscrito.

## Lista de abreviaturas e siglas

2D: Bidimensional
3D: Tridimensional
99mTc-PYP: Pirofosfato de 99m-tecnécio
α-Gal A: Alfa galactosidase A
AC: Amiloidose cardíaca
AE: Átrio esquerdo
AF: Ataxia de Friedreich
AL: Amiloidose por cadeias leves
angio-TC: Angiotomografia computadorizada coronariana
AR: Artrite reumatoide
ARU: Agentes de realce ultrassonográfico
AT: Arterite de Takayasu
ATTR: Amiloidose por transtirretina
BAV: Bloqueio atrioventricular
cineRM: Cinerressonância
CMD: Cardiomiopatia diabética
CMH: Cardiomiopatia hipertrófica
CPM: Cintilografia de perfusão miocárdica
CSF: Cardiomiopatia por sobrecarga de ferro
DAC: Doença arterial coronariana
DD: Disfunção diastólica
DDG: Doença de depósito de glicogênio
DF: Doença de Fabry
DM: Diabetes mellitus
DM2: Diabetes mellitus tipo 2
DMB: Distrofia muscular de Becker
DMD: Distrofia muscular de Duchenne
DNM: Doença neuromuscular
DP: Derrame pericárdico
EA: Espondilite anquilosante
EcoE: Ecocardiografia sob estresse farmacológico
ES: Esclerose sistêmica
ETE: Ecocardiograma transesofágico
ETT: Ecocardiograma transtorácico
FAC: Variação fracional da área (fractional area change)
FDG: Fluorodeoxiglicose
FEVD: Fração de ejeção do ventrículo direito
FEVE: Fração de ejeção do ventrículo esquerdo
FR: Fator reumatoide
GRADE: *Grading of Recommendations, Assessment, Development and Evaluation*
HAS: Hipertensão arterial sistêmica
HH: Hemocromatose hereditária
HP: Hipertensão pulmonar
HVE: Hipertrofia ventricular esquerda
IA: Inteligência artificial
IC: Insuficiência cardíaca
ICFEp: Insuficiência cardíaca com fração de ejeção preservada
ICFEr: Insuficiência cardíaca com fração de ejeção reduzida
IMC: Índice de massa corporal
IMVE: Índice de massa do ventrículo esquerdo
LES: Lúpus eritematoso sistêmico
MM: Multimodalidade
PET: Tomografia por emissão de pósitrons (*positron emission tomography*)
PET/CT com ^18^F-FDG: Tomografia por emissão de pósitrons associada a tomografia computadorizada com fluordesoxiglicose-flúor-18 (*fluorodeoxyglucose positron emission tomography/computed tomography*)
PSAP: Pressão sistólica da artéria pulmonar
RFC: Reserva de fluxo coronariano
RMC: Ressonância magnética cardíaca
RT: Realce tardio miocárdico
SBC: Sociedade Brasileira de Cardiologia
SC: Sarcoidose cardíaca
SCG: *Strain* circunferencial global
SIVd: Septo interventricular na diástole
SLG: *Strain* longitudinal global
SLPLVD: *Strain* longitudinal de parede livre do ventrículo direito
SPECT: *Single photon emission computed tomography*
SRG: *Strain* radial global
STE: *Speckle tracking echocardiography*
TAE: Tecido adiposo epicárdico
TAPSE: Excursão sistólica do plano do anel tricúspide (tricuspid annular plane systolic excursion)
TC: Tomografia computadorizada
TCC: Tomografia computadorizada cardíaca
TRE: Terapia de reposição enzimática
TV: Taquicardia ventricular
USV: Ultrassonografia vascular
VD: Ventrículo direito
VE: Ventrículo esquerdo
VEC: Volume extracelular
VHS: Velocidade de hemossedimentação
VSVE: Via de saída ventricular esquerda
