# Supplementary material for: Posicionamento sobre Avaliação Multimodalidade em Imagem Cardíaca em Pacientes com Doenças Sistêmicas – 2026
Source: Arq Bras Cardiol. 2026 Apr 28;123(4):e20260221. [Article in Portuguese] doi: 10.36660/abc.20260221 (PMC13155782; doi:10.36660/abc.20260221)
Supplement: Translation

# Translation: Position Statement on Multimodal Cardiac Imaging in Patients with Systemic Diseases – 2026

**DOI:** 10.36660/abc.20260221i

**Authors:** Viviane Tiemi Hotta, Isabela Bispo Santos da Silva Costa, Renata Christian Martins Felix, Maria Estefânia Otto Bosco, Minna Moreira Dias Romano, Nathalia Conci Santorio, Simone Cristina Soares Brandão, Daniela do Carmo Rassi Frota, Alessandro Cavalcanti Lianza, Simone Nascimento dos Santos, Marcelo Dantas Tavares de Melo, Ana Clara Tude Rodrigues, Guilherme Loureiro Fialho, Karen Saori Shiraishi Sawamura, Marco Stephan Lofrano Alves, Angele Azevedo Alves Mattoso, Claudio Tinoco Mesquita, Frederico José Neves Mancuso, Marcelo Iorio Garcia, José Luiz Barros Pena, Adenalva Lima de Souza Beck, Andressa Mussi Soares, Sandra Marques e Silva, Jorge Andion Torreão, Paulo R. Schvartzman, Fábio Fernandes, Carlos Eduardo Rochitte, Silvio Henrique Barberato

**Affiliations:** 1 São Paulo SP Brazil Instituto do Coração do Hospital das Clínicas da Faculdade de Medicina da Universidade de São Paulo, São Paulo, SP – Brazil; 2 São Paulo SP Brazil Fleury Medicina e Saúde, Grupo Fleury, São Paulo, SP – Brazil; 3 São Paulo SP Brazil Instituto do Câncer do Estado de São Paulo, São Paulo, SP – Brazil; 4 Rio de Janeiro RJ Brazil Instituto Nacional de Câncer, Rio de Janeiro, RJ – Brazil; 5 Rio de Janeiro RJ Brazil Clínica de Medicina Nuclear Villela Pedras, Rio de Janeiro, RJ – Brazil; 6 Brasília DF Brazil Universidade de Brasília, Brasília, DF – Brazil; 7 Brasília DF Brazil Hospital DF Star, Brasília, DF – Brazil; 8 Ribeirão Preto SP Brazil Universidade de São Paulo Hospital das Clínicas da Faculdade de Medicina de Ribeirão Preto, Ribeirão Preto, SP – Brazil; 9 Recife PE Brazil Hospital das Clínicas da Universidade Federal de Pernambuco, Recife, PE – Brazil; 10 Goiânia GO Brazil Faculdade de Medicina da Universidade Federal de Goiás, Goiânia, GO – Brazil; 11 Goiânia GO Brazil Hospital São Francisco de Assis, Goiânia, GO – Brazil; 12 São Paulo SP Brazil Hospital Israelita Albert Einstein, São Paulo, SP – Brazil; 13 São Paulo SP Brazil Instituto da Criança e Adolescente do Hospital das Clínicas da Faculdade de Medicina da Universidade de São Paulo, São Paulo, SP – Brazil; 14 São Paulo SP Brazil Hcor - Associação Beneficente Síria, São Paulo, SP – Brazil; 15 Brasília DF Brazil Eccos Diagnóstico CardioVascular Avançado, Brasília, DF – Brazil; 16 João Pessoa PB Brazil Universidade Federal da Paraíba, João Pessoa, PB – Brazil; 17 São Paulo SP Brazil Instituto de Radiologia do Hospital das Clínicas da Faculdade de Medicina da Universidade de São Paulo (INRAD HCFMUSP), São Paulo, SP – Brazil; 18 Florianópolis SC Brazil Universidade Federal de Santa Catarina, Florianópolis, SC – Brazil; 19 Curitiba PR Brazil Hospital de Clínicas da Universidade Federal do Paraná, Curitiba, PR – Brazil; 20 Salvador BA Brazil Hospital Santa Izabel, Salvador, BA – Brazil; 21 Niterói RJ Brazil Faculdade de Medicina da Universidade Federal Fluminense, Niterói, RJ – Brazil; 22 Rio de Janeiro RJ Brazil Serviço de Medicina Nuclear do Hospital Pro-Cardíaco, Rio de Janeiro, RJ – Brazil; 23 Rio de Janeiro RJ Brazil Serviço de Medicina Nuclear do Hospital Vitória, Rio de Janeiro, RJ – Brazil; 24 Niterói RJ Brazil Health, Science & Education Lab - Hospital Universitário Antônio Pedro, Niterói, RJ – Brazil; 25 São Paulo SP Brazil Universidade Federal de São Paulo - Escola Paulista de Medicina, São Paulo, SP – Brazil; 26 Rio de Janeiro RJ Brazil Hospital Universitário Clementino Fraga Filho da Universidade Federal do Rio de Janeiro, Rio de Janeiro, RJ – Brazil; 27 Belo Horizonte MG Brazil Hospital Felicio Rocho, Belo Horizonte, MG – Brazil; 28 Brasília DF Brazil Instituto de Cardiologia e Transplantes do Distrito Federal, Brasília, DF – Brazil; 29 Brasília DF Brazil Hospital Sirio Libanês, Brasília, DF – Brazil; 30 Cachoeiro de Itapemirim ES Brazil Hospital Evangélico de Cachoeiro de Itapemirim (HECI), Cachoeiro de Itapemirim, ES – Brazil; 31 Cachoeiro de Itapemirim ES Brazil Hospital Materno-Infantil - Unimed Sul Capixaba, Cachoeiro de Itapemirim, ES – Brazil; 32 Brasília DF Brazil Hospital de Base do Distrito Federal, Brasília, DF – Brazil; 33 Salvador BA Brazil Santa Casa da Bahia, Salvador, BA – Brazil; 34 Porto Alegre RS Brazil Hospital Moinhos de Vento, Porto Alegre, RS – Brazil; 35 Curitiba Paraná Brazil Cardioeco - Centro de Diagnóstico Cardiovascular, Curitiba, Paraná – Brazil

**Table:** 

Position Statement on Multimodal Cardiac Imaging in Patients with Systemic Diseases – 2026	
The report below lists declarations of interest as reported to the SBC by the experts during the period of the development of these statement, 2025/2026.	
Expert	Type of relationship with industry
Adenalva Lima de Souza Beck	Nothing to be declared
Alessandro Cavalcanti Lianza	Nothing to be declared
Ana Clara Tude Rodrigues	Nothing to be declared
Andressa Mussi Soares	Nothing to be declared
Angele Azevedo Alves Mattoso	Nothing to be declared
Carlos Eduardo Rochitte	Financial declaration A - Economically relevant payments of any kind made to (i) you, (ii) your spouse/partner or any other person living with you, (iii) any legal person in which any of these is either a direct or indirect controlling owner, business partner, shareholder or participant; any payments received for lectures, lessons, training instruction, compensation, fees paid for participation in advisory boards, investigative boards or other committees, etc. from the brazilian or international pharmaceutical, orthosis, prosthesis, equipment and implants industry: - GE Healthcare / Canon Medical Systems. B - Research funding under your direct/personal responsibility (directed to the department or institution) from the brazilian or international pharmaceutical, orthosis, prosthesis, equipment and implants industry: - Novartis: Cleerly.
Claudio Tinoco Mesquita	Financial declaration A - Economically relevant payments of any kind made to (i) you, (ii) your spouse/partner or any other person living with you, (iii) any legal person in which any of these is either a direct or indirect controlling owner, business partner, shareholder or participant; any payments received for lectures, lessons, training instruction, compensation, fees paid for participation in advisory boards, investigative boards or other committees, etc. from the brazilian or international pharmaceutical, orthosis, prosthesis, equipment and implants industry: - Alnylam: Amyloidosis. B - Research funding under your direct/personal responsibility (directed to the department or institution) from the brazilian or international pharmaceutical, orthosis, prosthesis, equipment and implants industry: - AstraZeneca: Amyloidosis; Alnylam: Amyloidosis. Other relationships Funding of continuing medical education activities, including travel, accommodation and registration in conferences and courses, from the brazilian or international pharmaceutical, orthosis, prosthesis, equipment and implants industry: - Alnylam: Amyloidosis; Pfizer: Amyloidosis.
Daniela do Carmo Rassi Frota	Financial declaration A - Economically relevant payments of any kind made to (i) you, (ii) your spouse/partner or any other person living with you, (iii) any legal person in which any of these is either a direct or indirect controlling owner, business partner, shareholder or participant; any payments received for lectures, lessons, training instruction, compensation, fees paid for participation in advisory boards, investigative boards or other committees, etc. from the brazilian or international pharmaceutical, orthosis, prosthesis, equipment and implants industry: - AstraZeneca Brazil Ltda: Forxiga.
Fábio Fernandes	Nothing to be declared
Frederico José Neves Mancuso	Nothing to be declared
Guilherme Loureiro Fialho	Other relationships Funding of continuing medical education activities, including travel, accommodation and registration in conferences and courses, from the brazilian or international pharmaceutical, orthosis, prosthesis, equipment and implants industry: - NovoNordisk: SBC Congress in São Paulo 2025.
Isabela Bispo Santos da Silva Costa	Nothing to be declared
Jorge Andion Torreão	Nothing to be declared
José Luiz Barros Pena	Nothing to be declared
Karen Saori Shiraishi Sawamura	Other relationships Any economically relevant equity interest in companies in the healthcare or education industry or in any companies competing with or supplying to SBC: - Online teaching: WavesMed.
Marcelo Dantas Tavares de Melo	Nothing to be declared
Marcelo Iorio Garcia	Nothing to be declared
Marco Stephan Lofrano Alves	Nothing to be declared
Maria Estefânia Bosco Otto	Nothing to be declared
Minna Moreira Dias Romano	Nothing to be declared
Nathalia Conci Santorio	Nothing to be declared
Paulo R Schvartzman	Nothing to be declared
Renata Christian Martins Felix	Nothing to be declared
Sandra Marques e Silva	Financial declaration A - Economically relevant payments of any kind made to (i) you, (ii) your spouse/partner or any other person living with you, (iii) any legal person in which any of these is either a direct or indirect controlling owner, business partner, shareholder or participant; any payments received for lectures, lessons, training instruction, compensation, fees paid for participation in advisory boards, investigative boards or other committees, etc. from the brazilian or international pharmaceutical, orthosis, prosthesis, equipment and implants industry: - Pfizer: Amyloidosis; Alnylam: Amyloidosis; PTC: Amyloidosis; AstraZeneca: Amyloidosis; Sanofi: Fabry disease; Takeda: Fabry disease. Other relationships Funding of continuing medical education activities, including travel, accommodation and registration in conferences and courses, from the brazilian or international pharmaceutical, orthosis, prosthesis, equipment and implants industry: - Pfizer: Amyloidosis; Alnylam: Amyloidosis; PTC: Amyloidosis; AstraZeneca: Amyloidosis; Sanofi: Fabry disease; Takeda: Fabry disease.
Silvio Henrique Barberato	Financial declaration A - Economically relevant payments of any kind made to (i) you, (ii) your spouse/partner or any other person living with you, (iii) any legal person in which any of these is either a direct or indirect controlling owner, business partner, shareholder or participant; any payments received for lectures, lessons, training instruction, compensation, fees paid for participation in advisory boards, investigative boards or other committees, etc. from the brazilian or international pharmaceutical, orthosis, prosthesis, equipment and implants industry: - Pfizer: Amyloidosis; AstraZeneca: HFpEF. Other relationships Funding of continuing medical education activities, including travel, accommodation and registration in conferences and courses, from the brazilian or international pharmaceutical, orthosis, prosthesis, equipment and implants industry: - Pfizer: Amyloidosis.
Simone Cristina Soares Brandão	Nothing to be declared
Simone Nascimento dos Santos	Nothing to be declared
Viviane Tiemi Hotta	Nothing to be declared

**Figure:** RA: rheumatoid arthritis; DD: Danon disease; GSD: glycogen storage diseases; FD: Fabry disease; DM: diabetes mellitus; DMD: Duchenne muscular dystrophy; BMD: Becker muscular dystrophy; AS: ankylosing spondylitis; SSc: systemic sclerosis; HFpEF: heart failure with preserved ejection fraction; HFrEF: heart failure with reduced ejection fraction; SLE: systemic lupus erythematosus: FA: Friedreich ataxia. *In the presence of coronary artery disease or microvascular dysfunction. Figure edited with contribution from Dr. Thais Baptista Teixeira.

## Table of contents

**1. Introduction** 8
**2. Applications of Multimodal Cardiac Imaging in Extracellular Matrix Disorders** 9
  **2.1. Cardiac Amyloidosis** 9
    **2.1.1. Echocardiography** 9
    **2.1.2. Cardiac Magnetic Resonance Imaging** 9
    **2.1.3. Myocardial Scintigraphy** 13
  **2.2 Cardiac Sarcoidosis** 16
    **2.2.1 Echocardiography** 16
    **2.2.2. Cardiac Magnetic Resonance Imaging** 18
    **2.2.3. Role of 18F-fluorodeoxyglucose Positron Emission Tomography** 19
**3. Applications of Multimodal Cardiac Imaging in Metabolic Diseases** 24
  **3.1. Diabetes Mellitus** 24
  **3.2. Obesity** 25
**4. Applications of Multimodal Cardiac Imaging in Rheumatological Diseases** 27
  **4.1. Systemic Lupus Erythematosus** 27
  **4.2. Systemic Sclerosis** 30
  **4.3. Ankylosing Spondylitis** 33
  **4.4. Rheumatoid Arthritis** 35
  **4.6. Takayasu's Arteritis** 39
**5. Applications of Multimodal Cardiac Imaging in Storage Diseases** 42
  **5.1. Iron Overload Cardiomyopathy** 42
  **5.2. Fabry Disease** 44
  **5.3. Glycogen Storage diseases** 49
**6. Applications of Multimodal Cardiac Imaging in Neuromuscular Diseases** 53
  **6.1. Duchenne Muscular Dystrophy** 53
  **6.2. Becker Muscular Dystrophy** 53
  **6.3. Friedreich Ataxia** 55
**7. Perspectives** 57
**8. Conclusions** 57
**References** 58

## 1. Introduction

Systemic inflammatory diseases, whether immune-mediated or not, are frequently associated with adverse cardiac manifestations due to alterations in cardiac rhythm, morphology, or function.^1,2^

Despite the diversity of systemic diseases with cardiac involvement and the wide spectrum of clinical manifestations, the applications and findings of multimodal cardiac imaging methods for these conditions have not been discussed comprehensively in a single document. Here we have compiled the main findings for the most relevant systemic diseases, selected for their prevalence and importance. Although rare, these conditions, such as cardiac amyloidosis (CA), cardiac sarcoidosis (CS), Fabry disease (FD), muscular dystrophy, etc., are frequently underdiagnosed.^1,2^ Thus, this position paper was developed to address the main systemic diseases that present with cardiovascular changes, focusing on diagnostic aspects related to imaging methods, although a detailed description of each disease is not within its scope.

Transthoracic echocardiography (TTE) is the imaging test of choice for diagnosing structural, functional, and hemodynamic cardiac abnormalities. Recent technological advances have improved echocardiography, including techniques for analyzing cardiac mechanics.^3,4^ Analysis of myocardial strain, especially global longitudinal strain (GLS), can identify subclinical dysfunction even in patients with preserved ejection fraction (EF). This technique has wide application in the early diagnosis of cardiovascular diseases.^5,6^ Recent studies have observed that GLS has diagnostic and prognostic value in several heart diseases and a direct impact on clinical management.^5–8^

Cardiac magnetic resonance imaging (CMR), which is considered the gold standard imaging method for cardiac structure, allows for myocardial tissue characterization, identifying signs of inflammation and edema through T1 and T2 mapping, as well as signs of fibrosis through late gadolinium enhancement, whose patterns can suggest the etiological factor in detected alterations. CMR also allows analysis of the extracellular matrix and extracellular volume (ECV), important diagnostic and prognostic parameters for cardiac alterations in systemic diseases.^9,10^

**Figure:** GRADE: Grading of Recommendations, Assessment, Development and Evaluation. Source: GRADE Working Group: www.gradeworkinggroup.org.

Nuclear cardiology techniques allow analysis of cardiovascular physiology, including metabolism, myocardial perfusion, innervation, and ventricular function, contributing to the early detection of cardiac abnormalities and enabling interventions before the patient progresses to severe and irreversible forms of the disease. Myocardial perfusion scintigraphy (MPS), which injects markers such as 99m-technetium (^99m^Tc)-sestamibi in association with physical or pharmacological stress, is a well-established method for evaluating patients with suspected obstructive coronary artery disease (CAD) and for risk stratification.^11^

Thus, cardiac imaging methods, such as echocardiography, CMR, computed tomography (CT), and metabolism and perfusion analysis techniques, play a fundamental role in the evaluation of patients with systemic diseases. Hence, knowledge of how multimodal cardiovascular imaging methods can be applied in systemic diseases is important, promoting their rational use in clinical practice, both by cardiologists and specialists in other areas.^3,5,9–12^ Therefore, documents guiding their application and discussing the main findings related to cardiac changes in these patients are still lacking. Therefore, we set out to provide a practical guide on the current and potential applications of multimodal imaging methods in systemic diseases, including when to request each examination and how to interpret its findings in the patient's clinical context, focusing on identifying signs suggestive of these conditions (Central Illustration).

This position statement was prepared according to the new Brazilian Society of Cardiology guidelines, which call for objective, concise, and shorter documents, as well as the adoption of the format according to the Grading of Recommendations, Assessment, Development and Evaluation (GRADE) system to indicate the level of evidence and strength of recommendations. This system consists of a simple grading model (strong, weak, or neutral recommendation; high, moderate, low, or very low certainty of evidence), as outlined in the diagram below.

Of note, diagnostic studies with adequate methodology that evaluate clinical outcomes are rare, even in the most prevalent cardiovascular diseases, such as CAD, heart failure (HF), and valvular heart disease. Hence, due to their low prevalence, randomized clinical trials, meta-analyses, and systematic reviews evaluating cardiac imaging methods for the diseases covered in this document are scarce or nonexistent. Thus, in some cases, even in the absence of large clinical trials, there may be a consensus that a particular imaging examination is clearly beneficial.

## 2. Applications of Multimodal Cardiac Imaging in Extracellular Matrix Disorders

### 2.1. Cardiac Amyloidosis

Amyloidosis is a systemic disease that results in restrictive cardiac physiology due to the extracellular accumulation of amyloid protein fibrils in the myocardium, leading to ventricular stiffness, diastolic dysfunction and, eventually, HF. In addition to these morphofunctional changes, this progressive deposition is associated with significant electrical changes, such as atrial fibrillation and various atrioventricular blocks. It is important to highlight that there are 2 main subtypes of CA, which have distinct etiologies but very similar clinical manifestations.^13,14^

- Light chain amyloidosis (AL), which results from the deposition of immunoglobulin light chains produced by a plasma cell disorder in the bone marrow.
- Transthyretin amyloidosis (ATTR), which results from the accumulation of misfolded transthyretin, which can be wild-type (ie, without detected mutations), or hereditary due to genetic mutations.

Differentiating between the AL and ATTR forms is challenging in clinical practice. However, the toxicity and quantity of fibril deposition is greater in AL than in ATTR, resulting in a more rapid increase in myocardial thickness,a higher incidence of pericardial effusion, and often greater impairment of systolic function parameters. Table 1 summarizes the main differences between these forms of CA.^15–17^

**Table 1:** Light chain amyloidosis vs. transthyretin amyloidosis

Aspects	Light chain	Transthyretin
Precursor protein	Light-chain immunoglobulin	Transthyretin protein
Pathogenesis	Excessive light chain production by plasma cells	Deposition of abnormal transthyretin (mutated or wild-type)
Clinical forms	Primary (associated with plasma cell dyscrasias), or associated with multiple myeloma	Hereditary (genetic mutation) or wild-type
Frequently affected organs	Heart, kidneys, peripheral nervous system, gastrointestinal tract	Predominantly the cardiac and peripheral nervous systems
Age of onset	Middle age to older adulthood (generally > 50 years of age)	Older adults (> 60 years of age), especially wild-type
Diagnosis	The presence of monoclonal light chains in serum and/or urine **and** amyloid infiltrate in tissue stained with Congo red dye	Genetic testing, scintigraphy with diphosphonates (99mTc-PYP, DPD or HMDP) and amyloid infiltrate unstained with Congo red
Treatment	Chemotherapy, immunotherapy, autologous stem cell transplantation	Transthyretin stabilizers (tafamidis, diflunisal), RNA interference agents (patisiran and vutrisiran); emerging gene therapy

^99m^Tc-PYP: 99m-technetium pyrophosphate; DPD: dicarboxypropane diphosphonic acid; HMDP: hydroxymethylene diphosphonic acid.

Early diagnosis of CA depends on high clinical suspicion due to its initial non-specific presentation, like other cardiac conditions.^18^ Thus, increased ventricular thickness in patients without hypertension or aortic valve disease, associated with extracardiac signs, such as carpal tunnel syndrome, spinal stenosis, peripheral neuropathy, or dysautonomia, can serve as an important diagnostic clue (Figure 1 and Table 2).^19^

**Figure 1:** Spontaneous rupture of the biceps muscle tendon is a warning sign of suspected clinical amyloidosis, mainly the transthyretin form. A – Relaxed upper left arm with a portion of the biceps next to the ulnar fossa. B – Contracted left biceps muscle with no major changes in relation to relaxed muscles.

**Table 2:** Warning signs of cardiac amyloidosis

Category	Warning signs
Medical record	Bilateral carpal tunnel syndrome Family history of heart failure or neuropathy Narrowing of the spinal canal with no obvious cause Dysautonomia (mainly orthostatic hypotension) Unexplained alterations in intestinal function Spontaneous tendon rupture (generally the biceps tendon)
Cardiac findings	Low-voltage electrocardiogram, disproportionate to myocardial thickness Pseudoinfarction pattern in electrocardiogram Low-flow/low-gradient aortic stenosis with preserved ejection fraction Pacemaker (mainly with family history and increased ventricular thickness) Heart failure with preserved ejection fraction
Systemic findings	Unexplained proteinuria Peripheral/autonomic neuropathy Macroglossia (in light chain amyloidosis) Ecchymoses, mainly periorbital purpura in light chain amyloidosis Persistent elevation of troponin and natriuretic peptides in the brain Edema syndrome Unexplained weight loss Presence of monoclonal paraprotein
Complementary examinations	Echocardiogram showing increased thickness with no obvious cause in the post-loading period Reduced left ventricular longitudinal strain with an apical sparing pattern Bone scintigraphy with intense cardiac uptake (Perugini grades 2 and 3) In cardiac magnetic resonance imaging, diffuse late enhancement, elevated native T1 value and elevated extracellular volume

#### 2.1.1. Echocardiography

TTE is the first-line diagnostic imaging exam in CA.^3^ Based on the pathophysiology of the disease, in which progressive myocardial interstitial amyloidosis occurs, increased biventricular thickness may be observed, in addition to increased stiffness, and reduced ventricular compliance, with diastolic dysfunction, reduced stroke volume and reduced left ventricular (LV) GLS and an apical sparing pattern (Table 3, Figures 2 and 3).^14,20–23^

**Table 3:** Main echocardiographic findings in cardiac amyloidosis

Echocardiographic parameter	Achados típicos na cardiomiopatia amiloide
Ventricular wall thickness	Increased myocardial thickness with a concentric pattern, often symmetrical and significant (> 12 mm); a predominant increase with an asymmetrical pattern can be observed in the septal wall
Left ventricular function	Initially preserved left ventricular ejection fraction, progressing to systolic dysfunction in advanced stages
Ventricular filling pattern	Restrictive diastolic dysfunction in advanced stages
Global longitudinal strain	Reduced global longitudinal strain with a typical apical sparing pattern
Appearance of heart valves	Thickening due to infiltration into the atrioventricular valves
Pericardial effusion	Frequent, usually mild to moderate
Atrial dilation	Atrial dilation and dysfunction, which can affect both atria
Septal hyperrefringence (sparkling pattern)	Present in some cases, but with low sensitivity and specificity for diagnosis on its own
Right ventricular function	Progressive dysfunction is associated with poor prognosis; present in advanced stages
Pulmonary hypertension	Present in advanced stages due to elevated left ventricular filling pressures
Atrial septum	Thickening of the atrial septum

**Figure 2:** Echocardiographic findings suggestive of cardiac amyloidosis. A – Parasternal long-axis view showing significantly increased ventricular thickness, reduced left ventricular diastolic diameter, and left atrial enlargement. B – Parametric image of reduced left ventricular global longitudinal strain (13.3%) with apical sparing.

**Figure 3:** Parametric analysis of left ventricle global longitudinal strain in a patient with light chain cardiac amyloidosis at 3 distinct time points: A – Initial diagnostic suspicion showing a reduced global value (13.6%) with apical sparing; B – 3 months later after diagnosis and the beginning of treatment; C – Follow-up treatment with chemotherapy, showing a significant drop in global longitudinal strain (8.9%) after 5 months, at which point the patient died.

Multiparametric echocardiographic models have been developed to improve diagnostic accuracy.^24^ These models incorporate several echocardiographic parameters, such as relative wall thickness, E/e’ ratio, GLS, and tricuspid annular plane systolic excursion (TAPSE), to create more accurate diagnostic scores for CA.^25^

Echocardiographic assessment of treatment response in CA primarily involves analysis of functional and morphological changes in the myocardium. Key indicators of treatment response include reduced LV wall thickness and stabilization or improvement of GLS. The E/e’ ratio and left atrial (LA) stiffness are echocardiographic parameters that can reflect changes in diastolic function after treatment: improvement correlates with favorable treatment response.^26–50^

#### 2.1.2. Cardiac Magnetic Resonance Imaging

CMR allows for precise evaluation of myocardial tissue changes associated with CA.^27^ Myofibril accumulation results in thickening of the LV walls and the interatrial septum, changes that can be detected by CMR morphological characterization and sequence analysis, often without significant dilation of the ventricular cavity.^28,29^ Increased atrial volume, diastolic dysfunction and, in advanced cases, reduced EF also occur.^29^ Pleural and pericardial effusions are common.^29^

As in the echocardiographic findings, patients with CA present with a more pronounced and diffuse reduction in GLS with relative apical sparing, in addition to reduced circumferential strain, especially in the basal regions.^49^ Biventricular strain analysis in CMR also allows detection of early right ventricular (RV) involvement, contributing to a more comprehensive assessment of the severity and extent of amyloid infiltration.^50^ CMR studies have found a significant correlation between LA dysfunction and amyloid burden, indicating that functional assessment of the atrial chamber can reveal early markers of cardiac involvement.^51^

Another observed tissue modification is increased total myocardial water content, which could result from volume expansion of the cardiovascular system due to the osmotic effect of accumulated proteins, as well as from increased intracellular water in myocytes subject to cytotoxic aggression from protein deposition, or even from reduced myocardial perfusion due to increased capillary spacing or obstruction.

The increased overall water content in myocardial tissue causes prolonged hydrogen relaxation times, both in the longitudinal (T1) and transverse (T2) axes.^43^ However, the most striking and clinically significant tissue alteration in CA is the marked expansion of myocardial ECV, which is attributed not only to the accumulation of amyloid fibrils during the symptomatic phases of the disease, but also to myocardial fibrosis repair. Therefore, the combination of protein deposition and interstitial fibrosis can be detected with high accuracy and even quantified through late enhancement (LE) techniques^42,44^ and myocardial ECV calculation.^32–35^ Normal myocardial ECV is around 25%, while in CA, especially the ATTR form, it can reach up to 60%.^47,48^

LE is one of the most common CMR techniques for CA assessment. A diffuse subendocardial or transmural enhancement pattern is typically observed, which contrasts with the more localized patterns seen in other cardiomyopathies.^52,53^ Global distribution of LE is associated with a higher amyloid burden and a worse prognosis.^54^ Typically, inversion of the myocardial recovery curve can be difficult to achieve, suggesting diffuse interstitial involvement.

Studies have shown that native T1 and ECV are significantly elevated in patients with amyloidosis, which can occur even in without visible LE, allowing for early diagnosis of cardiac involvement.^52,55^ ECV is highly sensitive to amyloid deposition. Recent meta-analyses have reinforced the role of LE, native T1, and ECV as predictors of mortality in cancer patients.^55,56^Figure 4 illustrates a case of CA evaluated by CMR.

**Figure 4:** Cardiac MRI of a patient with cardiac amyloidosis. Cinematic MRI shows biatrial enlargement and increased biventricular myocardial thickness; late gadolinium enhancement images show diffuse involvement and elevated native T1 values and extracellular volume. ECV: extracellular volume; MRI: magnetic resonance imaging.

Some initial studies have evaluated the role of CMR in myocardial strain assessment in patients with cancer, allowing for a more sensitive and earlier characterization of changes in myocardial function.^49,50^ Recent studies have demonstrated that, in addition to traditional GLS, parameters such as circumferential and radial LV strain, as well as RV strain components, offer further discrimination between AL and other conditions, such as hypertrophic cardiomyopathy.^49^

CMR could also play a role in therapeutic follow-up, indicating reduced ventricular mass.^57^ Serial quantification of ECV and native T1 could indicate a response to a specific therapy, such as transthyretin stabilizers or chemotherapy for AL.^58,59^ ECV reduction over time has been associated with clinical improvement and increased survival, highlighting its value as a marker of treatment response.^55,59^

CMR, together with findings from clinical history and laboratory data, allows differentiation of CA from other causes of increased myocardial wall thickness, such as hypertrophic cardiomyopathy or systemic arterial hypertension. Hypertrophic cardiomyopathy usually presents with asymmetric thickening and localized enhancement, whereas pressure overload–related hypertrophy is generally not associated with changes in T1 or ECV. A diffuse LE pattern, a marked increase in ECV, and elevated native T1 values are characteristic of CA and help distinguish it from other forms of myocardial hypertrophy.^29^

#### 2.1.3. Myocardial Scintigraphy

Current Guidelines recommend initial investigation of CA using multimodal cardiac imaging. When CA is suspected, it is essential to exclude monoclonal gammopathy by measuring immunoglobulin-free light chains in blood and urine. Once gammopathy has been ruled out, ^99m^Tc-pyrophosphate (PYP) scintigraphy becomes a highly accurate method. Myocardial tracer uptake with an intensity equal to or greater than that observed in the ribs (Perugini grades 2 or 3) allows the noninvasive diagnosis of transthyretin cardiac amyloidosis, without the need for endomyocardial biopsy. y (Figure 5).^60^

**Figure 5:** Scintigraphy with 99m-technetium pyrophosphate showing more intense uptake than in the ribs (planar image, Perugini grade 3) and confirmed uptake in the myocardium in single photon emission computed tomography (SPECT). After monoclonal gammopathy was excluded, the patient was diagnosed with transthyretin amyloidosis. LV: left ventricular; RV: right ventricular.

However, performing ^99m^Tc-PYP scintigraphy requires adherence to specific technical criteria to ensure its diagnostic accuracy for transthyretin cardiac amyloidosis: (a) the need to confirm myocardial tracer uptake by performing tomographic scintigraphic imaging with single photon emission computed tomography, in order to avoid false-positive results due to radiotracer accumulation in the blood pool; and (b) careful exclusion of monoclonal gammopathy by means of serum and urine immunoelectrophoresis and assessment of blood light chains. Only after exclusion of light-chain involvement does technetium-99m pyrophosphate scintigraphy present adequate diagnostic specificity for the noninvasive diagnosis of transthyretin cardiac amyloidosis, without the need for biopsy, considering that up to 20% of light-chain cardiac amyloidosis cases may show tracer uptake on scintigraphy.

Patients with confirmed low-intensity myocardial uptake may present with early forms of the disease. In such cases, repeat imaging after 6 months is recommended when there is high clinical suspicion. In addition, some rare genetic forms, such as Ser177Tyr, Tyr114Cys, and Phe64Leu, may also present with disproportionately low ^99m^Tc-PYP uptake, despite transthyretin cardiac amyloidosis with typical findings on echocardiography and CMR. In cases of high clinical suspicion with Perugini grade 0 or 1 technetium-99m pyrophosphate uptake (absence of uptake or uptake with intensity lower than that of the ribs), genetic sequencing is recommended to reduce the risk of diagnostic errors related to these rare mutations.^61^ Given the development of new treatments for alopecia areata, there is growing interest in determining whether imaging methods, including scintigraphy, can be used to monitor treatment response. However, this area is still under investigation, with the evidence in the early stages of consolidation.^62^

Observational studies have consistently demonstrated the applicability of scintigraphy in CA diagnosis and ATTR subtype identification^63–66^ (Table 4). The main limitations include a scarcity of large randomized clinical trials that have directly compared scintigraphy and biopsy regarding accuracy and prognostic outcomes, as well as the need for prognostic validation in different clinical contexts and populations. There is a clear recommendation that, when performed using an appropriate protocol, including the exclusion of monoclonal gammopathies, ^99m^Tc-PYP scintigraphy may be sufficient to diagnose ATTR and that it has practical, ethical, and economic benefits over biopsy (Table 5).

**Table 4:** Summary of key studies on 99m-technetium bone scintigraphy for cardiac amyloidosis

Study/Reference	Population	Sensitivity (%)	Specificity (%)	Prognostic value	Limitations
Gillmore et al.^66^	Patients with suspected ATTR without paraprotein	99-100	86	High uptake is associated with worse prognosis; limited by retrospective data	Observational study, prospective validation needed
Bokhari et al.^63^	Patients with suspected cardiac amyloidosis	91	92	Relationship between uptake and cardiac events suggested	Small sample, did not directly compare with biopsy in all cases
Castano et al.^65^	Patients with cardiac amyloidosis (various centers)	93	89	Uptake correlated with mortality and cardiac events in a cohort	Observational, non-randomized
Ahluwalia et al.^64^	Several studies; suspected ATTR	99 (visual analysis)	97 (SPECT) 96 (planar)	Confirms diagnostic value, but heterogeneity due to prevalence variation	Methodological heterogeneity among studies

ATTR: transthyretin amyloidosis; SPECT: single photon emission computed tomography.

**Table 5:** Cardiac multimodality imaging recommendations for patients with cardiac amyloidosis

Recommendation	Strength of recommendation	Certainty of evidence
Transthoracic echocardiography should be the initial diagnostic method for patients clinically suspected of coronary artery disease. ^27–30^	Strong	High
LV GLS assessment should be part of the echocardiographic assessment for CA.^23^	Weak	Moderate
Patients with significant paradoxical aortic stenosis should undergo supplemental LV GLS monitoring.^19,31,32^	Weak	Low
RV GLS assessment is recommended as part of the CA prognostic evaluation.^33,34^	Weak	Low
In CA, serial echocardiography is recommended for monitoring treatment response and disease progression.^35,36^	Weak	Moderate
Contrast echocardiography may be considered in cases with an insufficient echocardiographic window when coronary artery disease is suspected.^37^	Weak	Low
Diastolic function should be assessed upon suspicion of CA and during follow-up.^38^	Strong	High
Tissue Doppler (E/e') should be used to evaluate diastolic dysfunction in CA.^39^	Strong	Moderate
For diagnostic purposes, CMR with morphofunctional evaluation and tissue characterization techniques should be performed in patients with suspected cancer.^54^	Strong	High
For prognostic evaluation, CMR with late enhancement, native T1, and ECV techniques should be performed.^55,56^	Strong	High
In CA, serial CMR is recommended for monitoring treatment response and disease progression.^59^	Strong	Low
For patients with suspected ATTR and no monoclonal gammopathies, bone scintigraphy with 99mTc-PYP is recommended as a diagnostic method rather than endomyocardial biopsy. Positive test results (Perugini grades 2 or 3) are equivalent to a noninvasive diagnosis of ATTR.^64^	Strong	Moderate

^99m^Tc-PYP: 99m-technetium pyrophosphate; CA: cardiac amyloidosis; CMR: cardiac magnetic resonance imaging; ECV: extracellular volume; GLS, global longitudinal strain; LV: left ventricular; RV: right ventricular.

The recommended frequency of cardiac imaging examinations in patients with cardiac amyloidosis should be individualized according to the clinical status, the type of amyloidosis (AL or ATTR), response to treatment, and the availability of imaging modalities. According to current consensus statements, the following is recommended:

- Echocardiography (including myocardial strain): every 6 to 12 months to monitor disease progression or response to treatment, and it may be performed earlier in cases of clinical deterioration or changes in biomarkers.[1–2] Myocardial strain should be included whenever available, as it is sensitive for detecting disease progression.
- Cardiac magnetic resonance (CMR): every 12 to 24 months, particularly in the presence of clinical worsening and suspicion of disease progression based on biomarkers or echocardiography, for detailed assessment of structural involvement or therapeutic response.58,60 There is no consensus supporting intervals shorter than 12 months in clinically stable patients.
- Radionuclide scintigraphy (99mTc-PYP/DPD/HMDP): not recommended for serial monitoring of treatment response or disease progression, and should be reserved for initial diagnosis and definition of the amyloidosis subtype.^58,60^

**Key messages**

- In CA, multimodal cardiovascular imaging is imperative in clinical settings;
- Echocardiography is the first-line cardiac imaging examination;
- For echocardiographic phenotypes with increased thickness, GLS results demonstrating LV dysfunction and apical sparing, although not specific, increase the possibility of CA;
- Even without signs of pulmonary hypertension (PH), RV function parameters may be reduced;
- Despite advances, echocardiography cannot yet differentiate between AL and ATTR;
- Clinical worsening is expected with increased ventricular thickness, atrial dilation, reduced LV stroke volume, worsening diastolic dysfunction, and a reduced GLS. Preserved EF may occur even in advanced stages;
- In good treatment response, GLS will be stable or, in some cases, improve early, while morphological parameters change later;
- CMR provides both morphofunctional evaluation and tissue characterization, quantifying the extent of myocardial involvement through LE, native T1, and ECV techniques. Diffuse LE and marked elevation of native T1 and ECV values are diagnostic and prognostic markers in CA;
- ^99m^Tc-PYP scintigraphy, when performed using an appropriate protocol and after monoclonal gammopathies have been excluded, may be sufficient to diagnose ATTR, thus eliminating the need for biopsy.

### 2.2 Cardiac Sarcoidosis

Sarcoidosis is a rare, multisystemic inflammatory disease with uncertain pathophysiology,^67^ characterized by the formation of non-caseating granulomas in different tissues. Its prevalence is around 230 cases per 100,000 inhabitants.^68^

Clinical manifestations range from asymptomatic cases to progressive and relapsing disease with significant sequelae.^69^ Intrathoracic involvement, which is quite frequent (97% of cases), is often due to intrathoracic lymphadenopathy (87%) or pulmonary parenchymal infiltration (50%), with respiratory symptoms described in 43% of patients.^70^ However, cutaneous, ophthalmological, renal, cardiac, and central nervous system manifestations, among other less frequent conditions, have also been described.^71^

Cardiac involvement is clinically diagnosed in approximately 3% of patients with sarcoidosis,^72^ although it is present in 27% to 69% of autopsied cases.^73,74^ The interventricular septum is the most commonly affected region, followed by the posterior wall, with significant LV involvement.^75^

The most frequent clinical manifestations are HF, atrioventricular block, and ventricular arrhythmias, present in 52%, 34%, and 24% of cases, respectively.^76^ In some cases, sudden cardiac death may be the first manifestation of the disease.^77^

Patients with CS frequently present with systemic involvement.^72^ However, in 18% of cases, cardiac involvement occurs in isolation and is associated with a higher incidence of sustained ventricular tachycardia and a greater chance of developing HF, probably due to later diagnosis.^78^

Although pulmonary disease remains the leading cause of sarcoidosis mortality worldwide, cardiac involvement is an important prognostic factor; it is the leading cause of death in Japanese cases.^79^ In treated patients, 10-year survival rates can reach 90%.^80^ Treatment with corticosteroid therapy is associated with lower mortality, symptomatic arrhythmias, and hospitalization for HF, as well as improved LV function.^81^ These data reinforce the importance of initiating immunosuppressive treatment during the inflammatory phase, before the development of potentially irreversible fibrotic lesions, which are associated with a worse prognosis. Hence, early diagnosis is fundamental.

Because myocardial involvement is not homogeneous, endomyocardial biopsy, despite having high specificity, has low sensitivity (< 25%).^82–85^ Furthermore, the inherent risks of the procedure cannot be disregarded, with complications reported in approximately 10% of cases.^85^ Due to improved multimodal cardiac imaging assessment and diagnostic criteria,^86–88^ which allow for diagnosis in the absence of a histopathological examination, routine endomyocardial biopsy is no longer justified.

#### 2.2.1 Echocardiography

In general, conventional TTE, despite wide availability and accessibility, has low sensitivity for diagnosing CS. However, when combined with 12-lead electrocardiogram and the patient's medical history, its sensitivity for diagnosing cardiac involvement is 64%.^89^ However, it remains the first-line test and, according to international guidelines, should be performed on all patients diagnosed with systemic sarcoidosis.^87,90^

A study conducted in India that evaluated patients with an established diagnosis of extracardiac sarcoidosis found that an absolute LV GLS < 17.3% had a sensitivity of 80%, a specificity of 82.61%, and a negative predictive value of 98.7% for diagnosing cardiac involvement, while an absolute RV GLS < 21% had a sensitivity of 68%, a specificity of 78%, and a negative predictive value of 98%.^91^

A recent meta-analysis of 478 patients with sarcoidosis found that LV GLS and TAPSE were significantly lower in patients with cardiac involvement, suggesting possible early diagnostic markers.^92^ Three meta-analyses have also found that patients with sarcoidosis without clinical manifestations suggestive of cardiac involvement have significantly lower LV GLS than healthy individuals.^93–95^ These findings suggest that changes in myocardial strain may precede structural or functional changes detected by conventional echocardiography, favoring early detection of subclinical myocardial dysfunction.

Figure 6 shows an example of myocardial strain assessed through speckle tracking in 2 patients diagnosed with sarcoidosis, with and without cardiac involvement, both with preserved LV ejection fraction (LVEF).

**Figure 6:** Global longitudinal strain assessed through speckle tracking in 2 patients with sarcoidosis: (A) with cardiac involvement and (B) without cardiac involvement. In A, lower strain is observed in the basal segments of the septal walls and the lower and middle segments of the anterior lateral wall (lighter tones).

In addition to significantly increasing diagnostic accuracy, myocardial strain also has prognostic implications. Reduced RV free wall strain has been associated with a higher incidence of ventricular fibrillation, sustained VT, advanced AV block, and hospitalization for HF.^96^ A correlation has also been found with late gadolinium enhancement in CMR and uptake in positron emission tomography with ^18^F- fluorodeoxyglucose (^18^F-FDG positron emission tomography [PET]/CT).^96^ LV GLS has also shown increasing value in the prognostic assessment of patients with CS, being independently associated with a composite outcome of mortality, ventricular tachycardia, hospitalization for HF, and heart transplantation, in addition to being associated with uptake in ^18^F-FDG PET/CT.^97^

Some less sensitive, yet highly specific, findings that can affect prognosis should also be highlighted. LV dysfunction with LVEF < 40% is described in 21% of CS cases, while segmental abnormalities are found in up to 36% of patients,^98^ with possible progression to dyskinesia and aneurysms. Both have been associated with a higher incidence of all-cause mortality and severe ventricular arrhythmia,^98^ and they were included in the 2016 Japanese Circulation Society diagnostic criteria.^88^ However, only reduced LVEF was included in the 2014 diagnostic criteria.^86,87^Figure 7 shows transthoracic echocardiograms of CS patients with different patterns of LV dysfunction.

**Figure 7:** Different patterns of left ventricular dysfunction in patients with cardiac sarcoidosis undergoing transthoracic echocardiography: apical 4-chamber view in diastole (A and C) and systole (B and D). In A and B, extensive involvement of the left ventricle is evident, with focal narrowing and significant dysfunction. In C and D, segmental dysfunction is evident, including the formation of an apical aneurysm and a thrombus (arrow) inside it.

Narrowing of the basal segment of the interventricular septum (Figure 8), defined as thickness < 4 mm, is described in 28% of patients with CS.^99^ This has been considered an independent determinant of a composite outcome including all-cause mortality, symptomatic ventricular arrhythmia, bradyarrhythmia requiring pacemaker implantation, and admission for HF.^99^ It was also associated with LV dysfunction onset a median follow-up of 7.5 years.^100^

**Figure 8:** Transthoracic echocardiography findings: narrowing of the basal segment of the interventricular septum (measuring 3 mm) in in the parasternal long-axis view during diastole (A) and systole (B).

Ventricular involvement, which can occur primarily through granulomatous infiltration of the myocardium, has been described in 34% of cases^76^ (Figure 9). Secondarily, it can be caused by PH in association with primary lung involvement or advanced LV disease. In the absence of PH or diastolic dysfunction, echocardiographic findings of RV systolic dysfunction have a sensitivity of 10%-47% and a specificity of 82%-99% for diagnosing cardiac sarcoidosis.^90^

**Figure 9:** Transthoracic echocardiography findings: right ventricular dysfunction in a patient with cardiac sarcoidosis, without pulmonary hypertension, likely due to primary involvement. Apical 4-chamber view focused on the right ventricle during diastole (A) and systole (B). In C, right ventricle global longitudinal myocardial strain (-14.9%) assessed through speckle tracking.

Other less specific changes include diastolic dysfunction, mitral regurgitation, and pericardial involvement (Figure 10). Due to chronic inflammation in the myocardium, there is increased LV stiffness, with altered relaxation initially and increased filling pressures in more advanced cases.^101^ Minimal pericardial effusion has been described in 20% of patients with CS,^102^ but the disease can progress to constrictive pericarditis and cardiac tamponade.^103^

**Figure 10:** Transthoracic echocardiography findings: less specific changes indicating cardiac involvement due to sarcoidosis. In A, B, C, and D, restrictive diastolic dysfunction is characterized (E/A ratio = 3.2, indexed left atrial volume of 39 mL/m², and E/e’ ratio = 16.9). A: Spectral Doppler of mitral inflow. B: Left atrial volume, assessed by Simpson's method, in the 4-chamber apical view. C: Tissue Doppler of the medial mitral annulus. D: Tissue Doppler of the lateral mitral annulus. In E and F, mitral valve insufficiency is evident in the long-axis parasternal view. E: 2-dimensional mode showing coaptation failure associated with apical traction of the cusps and dilation of the mitral annulus. F: Color Doppler analysis showing significant mitral regurgitation jet. In G and H, a discreet, posteriorly located pericardial effusion (arrows) in the subcostal 4-chamber view (G) and parasternal long-axis (H) views.

However, mitral regurgitation is secondary in most cases, with the cusps exhibiting normal morphology and movement.^104^ Mitral regurgitation can occur due to mitral annulus dilation resulting from LA dilation associated with diastolic dysfunction, but it can also due to apical traction secondary to LV dysfunction. Recently, uptake in the papillary muscles has been demonstrated in 78% of cases with moderate or severe mitral regurgitation that were evaluated by ^18^F-FDG PET/CT, with 37% of patients showing a reduction of ≥ 1 severity grade after immunosuppressive treatment.^104^

#### 2.2.2. Cardiac Magnetic Resonance Imaging

CMR is a noninvasive and highly sensitive method for assessing CS, providing anatomical, functional, and tissue data. Although morphofunctional assessment with cinematic magnetic resonance imaging (MRI) may be normal in the early stages, myocardial thickening, areas of regional hypokinesia and, in advanced cases, ventricular dilation and dysfunction can be progressively visible.^105^ Cinematic MRI can also identify ventricular aneurysms, pericardial effusions, and heart valve abnormalities – findings that may be present in individuals with CS.^106^

Classic CMR findings include LE in non-ischemic patterns. LE in basal segments of the lateral and septal walls, especially in mesocardial or subepicardial patterns, is considered the most frequent manifestation of CS.^107^ However, LE has also been identified in other areas of the myocardium, potentially involving the subendocardium and a transmural pattern.^105,106,108,109^ The LE technique has detected focal lesions in 20%–30% of patients with a biopsy-confirmed diagnosis, which were consistent with non-caseating granulomas.^73,109^ The extent of LE is related to structural cardiac changes, including reduced EF, LV dilation, and the occurrence of non-sustained ventricular tachycardia – factors already recognized as risk indicators for sudden cardiac death^106^ (Figure 11).

**Figure 11:** Cardiac magnetic resonance imaging of a patient with cardiac sarcoidosis. A and B: Cinematic magnetic resonance imaging showing a slight increase in myocardial thickness. C: Dark-blood images in triple inversion recovery with anterior hypersignal suggestive of edema. D, E, and F: Native T1 mapping sequences (D and E) and extracellular volume (F) with increased values. G, H, and I: Late gadolinium enhancement images showing multifocal fibrosis with different patterns (transmural, mesocardial, and epicardial) in different segments of the left ventricle.

Advancement in multiparametric mapping, especially T1, T2, and ECV, has significantly improved the diagnostic accuracy and monitoring of inflammatory activity in sarcoidosis.^105,110^ T2-weighted imaging is sensitive to the presence of myocardial edema, which indicates active inflammation. In CS, elevated T2 values are associated with the acute phase of the disease, characterized by active granulomas and cellular infiltration (Figure 11). Focal or diffuse increases in T2 values can occur even without radiotherapy, making it a potential early marker of inflammation before fibrosis develops.^111^

In CS, elevated ECV values indicate both inflammation and chronic fibrosis, and their combination with T1 and T2 maps can distinguish different stages of the disease.^112^ ECV measurement proves particularly useful in longitudinal follow-up, enabling immunosuppressive therapy response assessment and the identification of disease progression, even without evident morphological changes^73^ (Figure 11). The combination of T1, T2, and ECV values can help identify different stages of CS:

– Elevated T2 with normal T1/ECV: acute inflammatory phase.
– Elevated T1/ECV with normal T2: chronic fibrosis without active inflammation.
– All values elevated: concurrent inflammation and fibrosis, suggesting active, more advanced disease.

This integrated approach to classic CMR findings (cinematic MRI and LE) with the use of multiparametric maps, is a useful tool for both early diagnosis and prognostic evaluation, as well as for therapeutic monitoring, being particularly beneficial in patients with nonspecific symptoms or inconclusive complementary examinations.^110,111,113,114^

#### 2.2.3. Role of ^18^F-Fluorodeoxyglucose Positron Emission Tomography

The ^18^F-FDG PET technique revolutionized CS assessment through *in vivo* identification of active inflammation in the myocardium, which is not possible with purely morphological examinations. This method is recommended by specialists for diagnosis and the monitoring and adjustment of immunosuppressive therapy.^115,116^

**Recommendations on diagnosis and monitoring:** ^18^F-FDG PET/CT is recommended for patients with suspected CS, especially those with atrioventricular blocks, ventricular arrhythmias, or ventricular dysfunction of undetermined etiology.^115–118^ Immunosuppressive treatment response should also be monitored every 3 to 6 months according to the clinical picture.^115,116,119,120^

**Prognostic significance – right ventricular involvement:** Because patients with RV ^18^F-FDG uptake have a significantly higher risk of adverse events, including arrhythmias and sudden death,^121,122^ early recognition is critical.

It is important to emphasize that sarcoidosis is a systemic disease and can affect multiple organs. Through whole-body ^18^F-FDG PET/CT, extracardiac involvement can be identified, which contributes to both diagnostic confirmation and therapeutic choice.^123,124^ However, preparation for the test is crucial, as its accuracy depends on adequate preparation to suppress physiological uptake of glucose in the myocardium. Recommendations include:^115,116,125,126^

- A diet high in fat and low in carbohydrates in the 24-72 hours before the exam;
- ≥ 12-hour fasting;
- Beta-hydroxybutyrate ensures greater metabolic specificity (≥ 0.335 mmol/L predicts adequate suppression).^127,128^

A 2020 meta-analysis of 17 studies (891 patients) found that cardiac perfusion and metabolism assessment had a sensitivity of 84% and a specificity of 83% in relation to these findings on the clinical spectrum of CS.^117^ Combined perfusion and metabolism assessment with ^18^F-FDG PET/CT distinguishes active inflammation (high FDG uptake) from fibrotic areas (perfusion defect without uptake), allowing for more precise stratification and individualized therapy (Figure 12).^116–118^ When available, combining it with CMR further increases diagnostic accuracy (Table 6).^129^

**Figure 12:** ^18^F-FDG PET/CT in a patient with suspected cardiac sarcoidosis. (A) Axial slice demonstrating focal and heterogeneous ^18^F-FDG uptake in the left ventricular myocardium, predominantly in the interventricular septum, with extension to the right ventricular wall, consistent with active inflammation. (B) Coronal reconstruction showing the extent of myocardial uptake. (C) Sagittal reconstruction confirming the focal pattern of myocardial hypermetabolism.

**Table 6:** Recommendations on multimodal cardiac imaging in cardiac sarcoidosis

Recommendation	Strength of recommendation	Certainty of evidence
Myocardial strain assessment through speckle-tracking transthoracic echocardiography is recommended for all patients with systemic sarcoidosis.^93–95^	Weak	Low
CMR should be performed in clinically suspected cardiac sarcoidosis.^112,114^	Strong	Moderate
^18^F-FDG PET/CT should be performed upon clinical suspicion of cardiac sarcoidosis to confirm the diagnosis.^115–118^	Strong	Moderate
^18^F-FDG PET/CT should be used in treatment response monitoring.^115,116,119,120^	Weak	Low
Whenever possible, ^18^F-FDG PET/CT should be integrated with CMR.^129^	Strong	Moderate
Always use ^18^F-FDG PET/CT (after rigorous cardiac preparation) to investigate cardiac sarcoidosis.^115,116,125^	Strong	High

^18^F-FDG PET/CT: 18F-fluorodeoxyglucose positron emission tomography/computed tomography; CMR: cardiac magnetic resonance imaging.

The recommended frequency of cardiac imaging examinations in patients with cardiac sarcoidosis should be individualized according to disease activity, symptoms, response to treatment, and risk of events. There is no international consensus on fixed intervals; however, major societies recommend echocardiographic evaluation (including strain analysis) every 6–12 months, annual cardiac magnetic resonance or at the clinician's discretion, and FDG-PET every 6–12 months in cases of active disease or during therapeutic adjustment.^130–133^

**Key messages**

- To increase diagnostic accuracy and improve prognostic assessment, myocardial strain assessment with speckle-tracking TTE is recommended for all patients diagnosed with systemic sarcoidosis. In patients with abnormal myocardial strain, further investigation with advanced imaging methods is recommended.
- Although they are later findings, cardiac sarcoidosis dysfunction and segmental contractility abnormalities are included in the diagnostic criteria and are associated with a higher incidence of all-cause mortality and serious ventricular arrhythmias.
- In patients with suspected cardiac sarcoidosis, it is advisable to look for thinning of the basal portion of the interventricular septum, defined as thickness < 4 mm in the long-axis parasternal view, as a prognostic marker.
- ^18^F-FDG PET/CT is essential for the diagnosis and monitoring of patients with suspected or confirmed cardiac sarcoidosis,^115,116^ particularly cases involving unexplained atrioventricular block, frequent ventricular arrhythmia, or unexplained ventricular dysfunction. ^18^F-FDG PET/CT also allows for systemic disease assessment.^58,61,65,134^
- ^18^F-FDG PET/CT has a prognostic impact, especially when for RV involvement.^121,122^ The patient must be properly prepared to avoid physiological myocardial uptake and ensure accurate interpretation of the results.^125–128^ This method allows for treatment adjustments according to inflammatory response.^119,134^
- Combining ^18^F-FDG PET/CT with CMR improves differentiation between active inflammation and fibrosis.^129^

## 3. Applications of Multimodal Cardiac Imaging in Metabolic Diseases

### 3.1. Diabetes Mellitus

Diabetes mellitus (DM), one of the most prevalent chronic diseases worldwide, significantly contributes to cardiovascular morbidity and mortality.^135^ Myocardial impairment in individuals with DM, particularly type 2 DM (T2DM), can result from frequently associated comorbidities, such as CAD and systemic arterial hypertension. However, pathophysiological mechanisms specific to DM can directly affect myocardial structure and function.^136^ Glycemic alterations, such as insulin resistance and glucose metabolism dysfunction, promote systemic effects and directly affect cardiomyocytes, compromising energy substrate use, mitochondrial function, and excitation-contraction coupling.^137^ In this context, diabetic cardiomyopathy refers to cardiac dysfunction observed in patients with DM in the absence of other cardiovascular diseases, such as CAD, hypertension, valve disease, or congenital heart disease.^137,138^

Echocardiography plays an important role in diabetic cardiomyopathy assessment, identifying the 3 types of myocardial involvement: LV systolic dysfunction, diastolic dysfunction, and altered LV geometry. It can also help predict cardiovascular outcomes.^135–138^

Although reduced LVEF is considered to confirm HF^137^ in patients with suggestive symptoms, almost half of patients with HF and T2DM present with HFpEF, which is more frequent in older adults, hypertensive patients, and women with T2DM. HFpEF is usually associated with less severe complications in the early stages of T2DM, while HF with reduced EF is associated with more severe complications of T2DM, which suggests that the severity and duration of hyperglycemia are important factors in the development of LV dysfunction.^139^

Myocardial strain analysis with two-dimensional (2D) and three-dimensional (3D) speckle tracking has been used to detect subclinical systolic dysfunction in diabetic patients. Studies have shown that patients with DM present reduced LV GLS values, indicating systolic dysfunction even in the absence of evident clinical symptoms.^135,140^ In this context, reduced LV GLS is a sensitive marker of systolic dysfunction, detecting functional impairment even with preserved EF, and it is associated with the occurrence of HF, mortality, and LV remodeling.^141,142^

LV diastolic dysfunction is present in ≥ 50% of asymptomatic and apparently healthy patients with DM.^137^ Grade 1 diastolic dysfunction (ie, without elevated filling pressures) is weakly associated with prognosis. However, greater LV diastolic dysfunction, including increased LV filling pressure (estimated by the ratio of E/e′ wave velocities), is associated with adverse outcomes, such as progression to HF and mortality, and is consistently higher in patients with T2DM than controls.^143,144^

Concentric LV remodeling, an early change in myocardial structure, is associated with subtle systolic dysfunction resulting from steatosis and alterations in the myocardial energy chain.^145^ Hypertrophy (ie, increased myocardial mass) is partially attributable to ECV, reflecting collagen deposition and fibrosis, factors that are associated with worse outcomes.^146,147^ Hyperinsulinemia, resulting from insulin resistance, can also induce myocyte hypertrophy when there is increased afterload.^148^

LV mass index and LA volume have been associated with an increased risk of overall and cardiovascular mortality in patients with T2DM. These structural parameters, along with indicators of diastolic function (E/A and E/e’ ratios), are important predictors of mortality, highlighting the prognostic value of echocardiography in this population.^149^ Furthermore, LV diastolic dysfunction is frequently associated with LA dilation. Atrial fibrillation, a complication of DM, is attributed to increased LV filling pressure and atrial distension, which, according to other evidence in the literature, suggests arteriopathy.^150^

The heterogeneity of patients with asymptomatic myocardial dysfunction and DM could have important implications for therapy. A recent analysis identified 3 patterns in this population: 1) a group with less compromised systolic and diastolic function (predominantly men) that is associated with favorable prognosis; 2) obese patients with hypertension and diastolic dysfunction (mostly women); and 3) a group with LV hypertrophy (LVH) and systolic dysfunction (mainly men). The latter groups have similar, less favorable, prognosis.^151^

CMR enables detection of early myocardial changes and atherosclerosis, being an accurate technique for evaluating cardiac function at rest and under stress. It is a useful method for analyzing macro-/microcirculation in patients with DM.^152–156^

The CE-MARC study directly compared the ability of CMR and scintigraphy to detect CAD, using coronary angiography as the gold standard, finding that CMR as superior to nuclear medicine.^157^ The details of CAD assessment through imaging methods will not be covered in this document.

Although LE is the technique of choice for detecting and quantifying myocardial fibrosis, its utility in assessing diffuse fibrosis is limited, as it depends on the difference in signal intensity between normal and scarred myocardium.^158^ To overcome this limitation, parametric imaging techniques, such as T1 mapping and ECV quantification, have been developed.

T1 mapping, both before (native) and after contrast infusion, enables quantitative assessment of T1 values in the myocardium, identifying the presence of diffuse and interstitial fibrosis, which cannot be detected by circulating biomarkers or LE.^158^ In conjunction with the pre-contrast map, the post-contrast T1 map is used to calculate ECV.

In patients with DM, those with more severe CVD have a higher risk of cardiovascular events.^159^

T2-weighted images can detect myocardial edema, which indicates an acute response to inflammation (myocarditis) or ischemia, allowing for precise quantification and monitoring over time.^158^ In T2-weighted images, the signal intensity of myocardial lesions is higher than skeletal muscle, although they are not specific enough to detect diabetic cardiomyopathy (Table 7).

**Table 7:** Recommendations on multimodality cardiac imaging in patients with diabetes mellitus

Recommendation	Strength of recommendation	Certainty of evidence
Transthoracic echocardiography should be performed as a first-line assessment for patients with suspected heart failure.^137,138,144^	Strong	High
^S^ystolic function should be assessed in appropriate acoustic windows using left ventricular global longitudinal strain when subclinical dysfunction is suspected.^135,140–143^	Strong	High

**Key messages**

- Diabetic cardiomyopathy refers to cardiac dysfunction in patients with DM in the absence of other cardiovascular diseases, such as CAD, systemic hypertension, valve disease, or congenital disease.
- HFpEF is the most common initial clinical presentation in DM, while HF with reduced EF is a later and more serious complication of DM.
- Reduced LV GLS indicates subclinical systolic dysfunction, even with preserved EF, and is a sensitive marker of HF, mortality, and ventricular remodeling.
- Progressive LV diastolic dysfunction with a consistently high E/e′ ratio is associated with worse outcomes.
- Increased LV mass index and LA volume are predictors of mortality.
- Early metabolic changes in myocytes can be detected by CMR. Direct CMR assessment of the myocardium can determine disease severity.^160^
- Microvascular disease, present in DM, is quantifiable through CMR and PET scans, although the latter is more expensive, less available, and involves radiation.^161^
- CMR allows for early detection of cardiac changes in patients with DM, providing early identification of HF etiology (microvascular disease, subendocardial/transmural fibrosis, inflammation, or infarction).
- CMR can identify hidden myocardial infarctions in up to 28% of patients with DM.^161^

### 3.2. Obesity

Obesity, which is highly prevalent, is an independent risk factor for several cardiovascular diseases.^162^ New approaches to classifying obesity have been developed, but they are beyond the scope of this document; further reading is recommended.^163^ Among the hemodynamic and structural changes associated with this condition are LVH, LA and LV dilation, diastolic dysfunction, and subclinical systolic dysfunction. In obese patients without sleep apnea, early RV changes have been reported to precede LV changes.^164^

This document presents practical and up-to-date recommendations on the use of echocardiography and CMR in obesity, which are based on the best available scientific evidence and are presented according to the GRADE system. A summary of the main recommendations can be found in Table 8.

**Table 8:** Echocardiography recommendations for obese patients

Recommendation	Strength of recommendation	Certainty of evidence
Perform transthoracic echocardiography as a first-line cardiovascular assessment in obese patients with symptoms or risk factors.^162,165^	Strong	High
Ultrasound contrast agents should be considered in obese patients with insufficient acoustic windows to optimize diagnostic accuracy.^165,168^	Weak	Low
Transesophageal echocardiography is safe and encouraged for indicated obese patients.^165^	Weak	Low
Stress echocardiography can be used in obese patients to assess coronary artery disease.^168^	Weak	Moderate
Systolic function should be assessed through global longitudinal strain when subclinical dysfunction is suspected in good acoustic windows.^171^	Weak	Moderate
In obese patients, cardiac chamber indexing should be according to height, rather than body surface area.^173,174^	Weak	Low
Epicardial adipose tissue can be quantified by echocardiography as a marker of cardiovascular events. Values > 10 mm indicate high risk.^175^	Weak	Moderate
The right ventricle should be routinely assessed in obese individuals, even in those without pulmonary arterial hypertension, given that these changes occur early, preceding the left ventricle.^164,165^	Weak	Moderate

TTE, which is widely available, safe, and affordable, is essential for noninvasive cardiovascular assessment in this patient population.^165^ However, this method involves technical limitations due to their often insufficient acoustic window, requiring ultrasound contrast agents or supplementation with other modalities, such as CMR.^166^ In addition to morphological and functional cardiac analysis, CMR allows for tissue characterization, being especially useful for early detection of subclinical changes associated with obesity. Furthermore, CMR provides excellent image quality and high diagnostic accuracy, even at a mean BMI of 50.3 (SD, 5.9) kg/m².^167^ For cases involving an insufficient acoustic window, transesophageal echocardiography is a valid alternative, since it reduces the risks associated with sedation, provided that head elevation and monitoring with capnography and oximetry are observed.^166^

Another effective method is pharmacological stress echocardiography, especially when CAD is suspected.^168^ The Stress Ultrasonography in Morbid Obesity (SUMO) study found that although 96% of 205 patients required ultrasound contrast agents for adequate segmental assessment, pharmacological stress echocardiography was safe and had a positive predictive value similar to non-obese individuals.^167^ CMR also plays a prognostic role in obese patients with CAD. The Stress CMR Perfusion Imaging in the United States (SPINS) study found that reversible perfusion deficit and LE are independent markers of mortality in obese patients.^169^ More recent results have confirmed these findings in patients with BMI > 40 kg/m².^170^

In obese patients with good acoustic windows, LV GLS allows for complementary analysis of LVEF to assess systolic and mechanical function.^171^ LV GLS can detect early subclinical dysfunction, contributing to early treatment for HF or as a diagnostic adjunct in HFpEF.^165,171,172^

The indexing of cardiac chamber sizes and volumes in obesity is controversial, especially for the LA. Indexing according to body surface may underestimate dilation. For greater accuracy, allometric indexing according to height is recommended. Evidence suggests that this metric outperforms isometric indexing by body surface.^173,174^ Suggested values with indexing possibilities are described in Table 9.^173,174^

**Table 9:** Formulas for left atrial indexing: advantages and disadvantages

Method	Type	Advantages	Disadvantages	Normal value
**LAV/BS (m²)**	Isometric	Current standard guidelines; easy to apply	Underestimates obese individuals; depends on body fat	< 34 mL/m².^173^
**LAV/height**	Isometric	Simple, a slight improvement over body surface area indexing.	Influenced by height, not supported by much research	No defined cut-off value.^174^
**LAV/height** ^2,7^	Allometric	Geometrically proportional to cardiac volume.	Empirical exponent; not derived from a healthy population.	~ < 16 m/m.^2,7,169,170^
**LAV/height** ^1,72^	Allometric	Derived from a study on a healthy population; best predictor of atrial fibrillation.	More complex calculation	< 22.1 mL/m.^1,72,173^
**LA/height** ^1,56^	Allometric	More sensitive than maximum volume; better correlation with atrial fibrillation.	Still underutilized in clinical practice	< 12.7 mL/m.^1,56,173^

BS: body surface; LAV: left atrial volume.

Epicardial adipose tissue thickness, which is rarely used in clinical practice, has a good correlation with CAD.^175^ Epicardial adipose tissue is visualized as an echo-free space between the myocardium and the visceral pericardium. It is measured perpendicular to the RV free wall at end systole in longitudinal or transverse parasternal sections. Values > 10 mm are associated with higher cardiovascular risk.^176^

According to recent evidence, both bariatric surgery and semaglutide have a positive impact on cardiac structure and function in obese individuals due to weight loss and metabolic control, promoting significant regression of LV myocardial hypertrophy. Serial echocardiographic follow-up is recommended, which can show reduced LV mass, improved diastole, reduced LA mass, and increased GLS, without significant changes in LVEF.^177–179^ CMR is also recommended for monitoring structural changes following bariatric surgery.^180^

The performance of cardiac imaging examinations in patients with diabetes mellitus and obesity should be individualized, based on symptoms, cardiovascular risk, and clinical changes. There is no recommendation for routine cardiac imaging in asymptomatic patients solely due to the presence of diabetes or obesity, according to guidelines from the American Diabetes Association and the American Heart Association.^133,182,183^

Echocardiography (including myocardial strain) may be repeated according to clinical evolution, generally at intervals of 6 to 12 months in patients with established disease or persistent symptoms.^133,183,184^ In asymptomatic patients, echocardiography is not routinely recommended, but may be considered in high-risk cases or in the presence of biomarker abnormalities.^137,185^

Cardiac magnetic resonance and scintigraphy (including myocardial perfusion) are not recommended for periodic screening in asymptomatic patients with diabetes and obesity. These examinations are indicated in symptomatic patients for the evaluation of structural heart disease, or when echocardiography is inconclusive due to technical limitations related to excess adipose tissue.^137,165,186^ The frequency of imaging depends on the clinical indication, and there is no fixed interval recommended for follow-up in asymptomatic patients.

**Key messages**

- Ultrasound contrast agents are recommended to improve the diagnostic accuracy of TTE and pharmacological stress echocardiography in patients with insufficient acoustic windows, a condition frequently associated with obesity.^165,168^
- LV GLS is superior to LVEF for detecting subclinical systolic dysfunction in obesity and should be performed whenever the acoustic window allows.^171^
- In obese patients, allometric indexing of LA volumes by height is more accurate and should replace body surface indexing.^173,174^
- Epicardial adipose tissue, which is measurable by echocardiography, can be monitored since it is associated with myocardial stiffness, diastolic dysfunction, and increased cardiovascular risk.^175,176^
- High-quality CMR is possible regardless of the degree of obesity.
- In obese patients, impaired perfusion and increased LE in CMR are predictors of mortality.
- Cardiac ventricular mass and cardiac function are more accurately assessed through CMR, making it a suitable method for detecting early changes and assessing improvement after bariatric surgery.

## 4. Applications of Multimodal Cardiac Imaging in Rheumatological Diseases

### 4.1. Systemic Lupus Erythematosus

Systemic lupus erythematosus (SLE) is a chronic, multisystemic, inflammatory autoimmune disease that frequently presents with cardiovascular manifestations, which are an important cause of morbidity and mortality in this patient population.

Pericardial disease, valve disease, PH, and myocarditis are the main cardiovascular manifestations in SLE, and echocardiography plays a role in their diagnosis and prognosis.^1^

Pericardial disease, the most common cardiovascular condition in SLE, is often its first manifestation and is present in up to 50% of cases. The vast majority of cases present with mild pericardial effusion, without major hemodynamic repercussions. Careful assessment of the clinical picture is essential to determine whether the presentation represents polyserositis or isolated pericardial effusion. In lupus pericarditis, which is frequently associated with a disease flare, patients may present with a characteristic clinical picture, including the classic triad of chest pain, fever, and pericardial friction rub, along with electrocardiographic changes and varying degrees of pericardial effusion. Complicated pericarditis is rare, and in only 1% to 2% of patients does the effusion become significant enough to result in cardiac tamponade. Constrictive or infectious pericarditis is rare.^187^

Valvular involvement in SLE is very frequent, reaching up to 75% in autopsy studies. Common lesions include nonspecific valvular thickening and Libman-Sacks endocarditis, which has a peculiar pathophysiology. These sterile verrucous lesions (deposits formed by immune complexes, fibrin, and platelet thrombus) are more common when SLE is accompanied by antiphospholipid antibodies. TTE aids in the differential diagnosis between Libman-Sacks endocarditis and infective endocarditis, an important consideration in immunocompromised patients. The mitral valve is more commonly affected in Libman-Sacks, and the lesions are generally located on its atrial surface and are less mobile. They can cause valvular disturbances (usually regurgitation) (Figure 13), embolic phenomena, and can serve as a substrate for infective endocarditis.^188^Figure 13 illustrates the case of a patient with a diagnosis of SLE presenting with aortic valvulitis and severe aortic regurgitation, as well as myocarditis, as assessed by echocardiography.

**Figure 13:** Echocardiographic images of a patient diagnosed with systemic lupus erythematosus with aortic valvulitis and significant aortic regurgitation (A, B, and C), as well as myocarditis, which is evidenced by a reduction (-7.9%) in left ventricle global longitudinal strain in the 3-chamber view (D). In (A), transesophageal echocardiography shows moderate thickening and a slight retraction of the aortic valve opening (assessed in the long axis), associated with (B) significant regurgitation in color Doppler. In (C), continuous Doppler spectral curve shows a dense jet, associated with significant regurgitant volume and reduced pressure half-time (237 ms).

The prevalence of PH among patients with SLE varies between studies, with estimates ranging from 0.5% to 17.5%. Typically, these are female patients of childbearing age and are classified as group I by the PH consensus.^189^ TTE is fundamental in estimating pulmonary artery systolic pressure, which is calculated using the maximum velocity of tricuspid regurgitation. TTE can also be used to assess direct and/or indirect signs of PH, such as a hypertrophied and dilated RV, an enlarged right atrium, a displaced LV (ie, lost sphericity, assuming a "D" shape), and a notch pattern in the RV outflow tract (suggesting high pulmonary vascular resistance). It is important to highlight that: 1) there is no specific pattern of PAH in SLE; 2) TTE allows for a probabilistic diagnosis, with right heart catheterization remaining the gold standard.^190^

The prevalence of myocarditis in SLE is still around 10%, despite significant advances in immunomodulatory drugs.^191^ In such cases, TTE plays a role in detecting segmental anomalies and determining the degree of systolic dysfunction (Figure 14). LVEF is sometimes preserved, but with GLS changes, frequently resulting in subclinical dysfunction.^192^ Additional factors can negatively affect myocardial function, as these patients often present with accelerated atherosclerosis and arrhythmias.

**Figure 14:** Echocardiographic images of a patient diagnosed with systemic lupus erythematosus with significant ventricular dysfunction, evident in the apical 4-chamber view by the small variation in diastolic (A) and systolic (B) left ventricle diameters, with signs of spontaneous contrast in the left ventricular cavity. In (C) and (D), left ventricular ejection fraction (0.22) was calculated using Simpson's method; the apical 4-chamber view shows significant systolic dysfunction.

In systemic diseases, recommendations for echocardiography depend on the prevalence of associated heart disease, the specific characteristics of cardiac involvement in each situation, and clinical suspicion of cardiac involvement.^3^

CMR has become the main noninvasive tool for early detection and tissue characterization of myocardial involvement in patients with SLE.^193^ Studies have found a high prevalence of myocardial fibrosis in these patients compared to controls, even in the absence of clinical symptoms. One of the largest cohorts involving CMR with LE found a significant frequency of myocardial fibrosis, which was often located in the inferolateral and septal regions and was associated with longer disease duration and the presence of multisystem involvement.^194^ Moreover, the location and extent of LE were associated with worse ventricular function and poorer clinical prognosis, suggesting its potential role as a prognostic marker.^195^

Quantitative techniques, such as T1 and T2 mapping, have also contributed to the assessment of inflammatory activity and tissue remodeling. Recent studies have shown that patients with SLE have elevated T1 and T2 values, particularly those who have not been treated with antimalarials.^196^ CMR with tissue tracking has also allowed subclinical biventricular dysfunction characterization in asymptomatic patients, helping identify those with increased cardiovascular risk who may benefit from early treatment.^197^

A recent systematic review and meta-analysis of 18 studies (955 patients) comprehensively investigated the role of quantitative CMR biomarkers in patients with SLE, finding that individuals with SLE had significantly higher native T1, ECV, and T2 values than healthy controls. Furthermore, LV GLS was significantly lower in patients with SLE, suggesting myocardial inflammation and fibrosis, which was related to cardiovascular involvement and had prognostic implications.^193^

In addition to direct myocardial involvement, coronary microvascular dysfunction is a concern in patients with SLE, frequently associated with systemic inflammatory activity. A cohort study with a 5-year follow-up found that coronary microvascular dysfunction (assessed in perfusion-weighted MRI) and atherosclerotic plaques (assessed in CT angiography) were associated with future cardiovascular events, even in patients without significant coronary artery obstruction. These findings reinforce the importance of early screening for myocardial ischemia and vascular inflammation in patients with SLE.^198^

In addition, CMR has emerged as the most sensitive modality for diagnosing pericarditis in the autoimmune context, allowing the detection of pericardial thickening, effusion, and, above all, active inflammation through radiofrequency ablation and increased signals in T2-weighted sequences.^194^ A recent review on autoimmune pericarditis, highlighted CMR as essential not only for accurate diagnosis, but also for monitoring treatment response and detecting complications, such as transient or irreversible constrictive pericarditis.^199^ In patients with SLE, early identification of active pericardial involvement in CMR can directly affect therapeutic management, allowing for earlier immunosuppressive interventions and potentially preventing progression to chronic forms of pericardial fibrosis.

Therefore, CMR offers a comprehensive and sensitive assessment of cardiac involvement in patients with SLE, having potential not only for diagnosis but also for prognosis and therapeutic monitoring. Systematic incorporation of CMR in follow-up could result in the early detection of complications and better risk stratification for this patient population.

Additionally, nuclear medicine can contribute to assessment in the context of perimyocarditis and CAD.^200 18^F-FDG PET/CT has been used to assess acute pericarditis, confirming the presence of inflammation, demonstrating diffuse radiotracer uptake in the pericardium, generally at a low-to-moderate intensity. However, radiotracer activity in the pericardium is not specific, and cannot differentiate the etiology of different inflammatory or infectious processes, nor even neoplastic involvement.^201,202^ Because no studies have focused on ^18^F-FDG PET/CT in patients with SLE, this method should only be recommended as a complementary assessment in situations of diagnostic uncertainty.^202^

Similarly, ^18^F-FDG PET has been studied in relation to myocarditis in various clinical settings.^203^ In SLE, the evidence is limited to case reports, in which it aided diagnosis and treatment response assessment.^204^ Unfortunately, the procedure is expensive, not widely available, and involves radiation.

Patients with SLE have a high prevalence of CAD, and MPS may indicate myocardial ischemia, even without significant epicardial vessel obstruction or symptoms, which are associated with an increased occurrence of coronary events.^205–207^ Thus, MPS is useful for identifying myocardial ischemia and estimating cardiovascular risk in patients with SLE, which can be considered an equivalent of CAD.^2^ Myocardial perfusion studies using PET/CT with radiotracers (such as 13N-ammonia and ^15^O-H_2_O) have demonstrated changes in coronary flow reserve in patients with SLE, even without signs of systemic inflammation or cardiovascular symptoms, indicating the presence of persistent microvascular dysfunction in up to 57% of cases.^208–210^ Unfortunately, these radiopharmaceuticals are not produced in Brazil. Here, coronary flow reserve can be assessed through myocardial scintigraphy with a cadmium zinc telluride camera. However, no studies have used this method specifically in SLE (Table 10).

**Table 10:** Recommendations on cardiac imaging methods in patients with systemic lupus erythematosus

Recommendation	Strength of recommendation	Certainty of evidence
Transthoracic echocardiography should be performed in patients with SLE to investigate pericardial disease, valve dysfunction, pulmonary hypertension, and myocarditis.^1,187–190^	Weak	Moderate
Use cardiac magnetic resonance imaging with cinematic rendering, late enhancement, and T1/T2 mapping to assess cardiovascular involvement in patients with SLE.^188^	Weak	Moderate
Use ^18^F-FDG PET to diagnose pericarditis and myocarditis.^191^	Weak	Low
Use myocardial perfusion scintigraphy to detect myocardial ischemia.^3,192,193^	Strong	Moderate
Perform coronary flow reserve assessment.^195–197^	Weak	Moderate

^18^F-FDG PET: 18F-fluorodeoxyglucose positron emission tomography; SLE: systemic lupus erythematosus.

Despite the wide range of cardiac manifestations in patients with SLE, there is still no standard routine for cardiac imaging screening in this population. However, the expert panel of this document recommends multimodal cardiac imaging evaluation for the early detection of cardiovascular abnormalities and risk stratification in this patient population, which carries a very high cardiovascular risk. The frequency of imaging examinations should be individualized based on clinical assessment of symptoms and disease activity, with annual echocardiography recommended in higher-risk patients.^211–214^

**Key messages**

- The main cardiac manifestations in SLE, in which TTE plays a diagnostic and a prognostic role, are pericardial disease, valve disease, pulmonary hypertension, and myocarditis.
- Valvular involvement is quite common in SLE, and the most common type of lesion is nonspecific valve thickening.
- The prevalence of PH in patients with SLE varies between studies, ranging from 0.5% to 17.5%. TTE is fundamental in estimating PA systolic pressure and assessing direct and/or indirect signs of PH.
- TTE can provide a probable diagnosis of PH, with right heart catheterization remaining the gold standard.
- In patients with SLE, myocardial involvement can be identified by CMR through the presence of non-ischemic pattern myocardial fibrosis and elevated native T1/ECV results.
- Inflammation and edema can be adequately visualized in T2 mapping.
- Pericarditis is relatively common in patients with SLE, with CMR being the method of choice for assessing active disease.

### 4.2. Systemic Sclerosis

Systemic sclerosis is a rare autoimmune connective tissue disease whose pathogenesis is characterized by 3 markers: small vessel vasculopathy, autoantibody production, and fibroblast dysfunction leading to increased extracellular matrix deposition. Diagnosis is primarily clinical, supported by serological tests and imaging studies.^215^ According to the American College of Rheumatology/European League Against Rheumatism (ACR/EULAR), its main clinical features are skin thickening, Raynaud's phenomenon, and internal organ involvement.^216^ The type and severity of organ involvement will determine prognosis. Despite recent improvements in survival, systemic sclerosis has the highest morbidity and mortality of any rheumatological disease.^215^

Cardiovascular impairment is a major concern in systemic sclerosis, being one of the leading causes of mortality among these patients.^217,218^ Cardiac complications can be both primary and secondary, including arrhythmias,^219^ HF, pericardial disease, and myocardial fibrosis. Echocardiography is a widely available, noninvasive imaging method that plays a central role in assessing cardiac structure and function in systemic sclerosis. It is particularly useful for detecting both overt and subclinical cardiac involvement, as cardiac changes are often asymptomatic in the early stages of the disease. Because myocardial involvement is associated with a worse prognosis, it is important to identify early changes to prevent progression.

In patients with systemic sclerosis, TTE allows assessment of cardiac involvement, detecting systolic and diastolic dysfunction, valve abnormalities, and PH^220^ (Figure 15). Speckle-tracking echocardiography with myocardial strain assessment is especially useful for identifying subclinical myocardial dysfunction in patients with systemic sclerosis, which is often imperceptible through conventional echocardiography. Patients with systemic sclerosis present significantly lower myocardial strain parameters than healthy controls, indicating myocardial dysfunction involving the ventricles and the atria.^221–226^ Even when LVEF is preserved, GLS may be reduced in both ventricles,^225^ suggesting that this method may be more sensitive than traditional echocardiography in identifying early cardiac involvement.

**Figure 15:** IEchocardiographic images of a patient diagnosed with systemic sclerosis and interstitial pneumonia. The images show normal systolic function (A) and increased left atrial volume associated with moderate diastolic dysfunction (B, C, and D). Right ventricle assessment shows preserved dimensions and systolic function through tricuspid annular plane systolic excursion and s’ (E, F, and G), but increased pulmonary systolic pressure (assessed with tricuspid regurgitation: flow velocity = 3.34 m/s) (H and I).

Additionally, echocardiography is a valuable noninvasive method for initial assessment of pulmonary arterial pressure, especially considering that PH is associated with higher mortality in patients with systemic sclerosis.^227^ Pulmonary arterial pressure measurement by echocardiography can indicate the presence of interstitial lung disease, left heart involvement with increased pulmonary capillary wedge pressure, and pulmonary vasculopathy with associated PAH.^227^ However, although useful for PH screening, the diagnostic accuracy of echocardiography is considered moderate, with sensitivity and specificity around 83% and 72%, respectively.^228^ Due to its limitations, echocardiography should not be the sole measure for estimating pulmonary arterial pressure; it must be combined with other methods: right heart catheterization is considered the most reliable test for diagnosing and monitoring PH.^228,229^

RV monitoring is crucial for patients with systemic sclerosis and PH, as RV dysfunction is associated with increased morbidity and mortality. Echocardiographic measurements, such as TAPSE, fractional area change, and tissue Doppler systolic velocity (S’) are routinely used to assess RV systolic function (Figure 15), together with 3D echocardiography and RV strain measurement.^190^ Additionally, inadequate RV adaptation to increased pulmonary arterial afterload is a determining factor in adverse outcomes in systemic sclerosis. Measurement of the RV-pulmonary arterial coupling, determined through noninvasive indices such as TAPSE/pulmonary artery systolic pressure and fractional area change/pulmonary artery systolic pressure, has prognostic value in identifying unfavorable clinical outcomes.^230^ It is important to highlight that the echocardiographic parameters used to assess ventricle function are limited due to the anatomical complexity of this chamber and the influence of loading conditions. Therefore, these parameters should not be used in isolation, but rather in conjunction with other indicators for more accurate assessment.^190^

Echocardiography can also detect asynchronous septal movement, which is related to the myocardial involvement observed in CMR. This echocardiographic finding antecedes abnormal MRI results, such as increased ECV and myocardial fibrosis, indicating cardiac involvement in systemic sclerosis.^231^

In addition to TTE, stress echocardiography can be used in systemic sclerosis, allowing detection of increased pulmonary artery systolic pressure^229,232^ and/or LV filling pressure^233^ during exercise, indicating HFpEF, which is associated with unfavorable clinical outcomes.^232^ PAH diagnosis should not be based solely on elevated pulmonary artery systolic pressure during exercise, since diastolic dysfunction, assessed by E/e’, is frequently observed in these patients.^235^ During stress echocardiography in patients with systemic sclerosis, it is crucial to monitor pulmonary artery systolic pressure, RV and LV function, and signs of pulmonary congestion. Complications, although rare, should be carefully monitored.

Finally, individualized management of systemic sclerosis, including cardiac complications, should be based on patient characteristics and disease severity, considering the extent of organ involvement. Treatment may include immunosuppressive therapy, vasodilators, and specific medications for cardiac complications (antiarrhythmics or HF protocols), as well as endothelin receptor antagonists or phosphodiesterase-5 inhibitors for managing PH.^235,236^ Regular follow-up with TTE is essential to monitor treatment side effects, assess cardiac function, and optimize clinical outcomes.^235^

In patients with systemic sclerosis, CMR allows for detailed tissue characterization, enabling early detection of myocardial inflammation and fibrosis, which may be present even without clinical manifestations.

A non-ischemic LE pattern is a common CMR finding in patients with systemic sclerosis and indicates the presence of myocardial fibrosis, even in the absence of symptoms or evident ventricular dysfunction. LE is associated with a worse cardiovascular prognosis (arrhythmia and sudden death). In the multicenter Scleroderma Arrhythmia Clinical Utility Study (SAnCtUS), LE was an independent predictor of ventricular arrhythmia, reinforcing its prognostic value in this population.^237^ Advanced techniques, such as LE entropy analysis, have also shown additional prognostic value by quantifying the heterogeneity of fibrosis, which is associated with worse clinical outcomes.^238^

In addition to the LE technique, CMR allows assessment of diffuse fibrosis using native T1 maps and ECV. These parameters have been found to be elevated in patients with systemic sclerosis, even in early stages and without conventional changes, indicating subclinical myocardial involvement.^239^ T2-weighted imaging has also stood out in inflammatory activity assessment, being particularly useful in differentiating between active inflammation and chronic fibrosis. In a recent study, T2-weighted imaging demonstrated greater sensitivity than visual analysis in detecting myocardial inflammation associated with systemic sclerosis, even in asymptomatic patients.^240^

In another study that compared patients with systemic sclerosis to healthy controls, overall myocardial T1 and T2 values were significantly higher in patients with systemic sclerosis. Notably, 62% of patients with normal CMR findings (no LE or apparent edema) had elevated native T1 and/or T2 values. Moreover, 40% of patients with increased T1 values had larger LV diastolic volumes, larger biventricular systolic volumes, elevated global T2, and a more frequent history of digital ulcers.^240^

The emerging role of CMR in treatment response assessment is also important. In a prospective study, patients with myocarditis associated with systemic sclerosis were re-evaluated after immunosuppressive treatment, and a reduction in signs of myocardial inflammation was observed in T2 mapping and LE, demonstrating that CMR has potential in treatment response monitoring.^242^

A recent systematic review assessed the prognostic role of CMR in patients with systemic sclerosis. The main CMR parameters identified as consistent predictors of all-cause mortality were LE, native T1, ECV, and ventricular strain indices. Although less investigated, other markers, such as LA strain, perfusion measurements, and parameters obtained through stress CMR, have also demonstrated prognostic value but still lack robust validation.^243^

Evidence supports the use of nuclear medicine in cardiovascular evaluation of patients diagnosed with systemic sclerosis. Although limited to small clinical studies, the results are interesting. For more than 4 decades, MPS, first with thallium-201 chloride and later with ^99m^Tc-sestamibi, has shown that approximately 80% of patients with systemic sclerosis have perfusion defects, even in the absence of atherosclerotic CAD. An association has been found between Raynaud's phenomenon and myocardial ischemia during pharmacological stress testing with dipyridamole or exposure to cold in approximately 50% of cases. These findings indicate that microvascular dysfunction and vasospasm are an important pathophysiological substrate for myocardial damage in this context.^244–248^

PET/CT coronary flow reserve assessment with ^82^rubidium has demonstrated that systemic sclerosis is an independent predictor of reduced flow reserve, which occurred in 89% of cases, being a marker of microvascular dysfunction in these patients.^249^

Diastolic dysfunction has also been observed in MPS with ^99m^Tc-sestamibi gated SPECT imaging: those with a high total skin thickness score had a lower peak LV filling rate than those with a low score or the control group. This change was identified even in the absence of perfusion defects. The authors emphasized that this result might correlate with the severity of systemic sclerosis and, hence, it could serve as an early indicator of myocardial involvement. One study also found alterations in cardiac sympathetic function in 123I-MIBG scintigraphy, corroborating the hypothesis that autonomic dysfunction is common among in this patient population.^250,251^

Finally, abnormal myocardial radiotracer uptake in ^18^F-FDG PET/CT, an indication of subclinical myocarditis, has been observed in half of systemic sclerosis patients without cardiovascular symptoms, which may point to early cardiac involvement in the disease.^252^

More robust studies are needed to strengthen these findings and determine their prognostic impact. However, it seems reasonable to perform MPS to identify myocardial ischemia in patients with systemic sclerosis, especially symptomatic ones (Table 11).

**Table 11:** Recommendations on multimodal cardiac imaging in patients with systemic sclerosis

Recommendation	Strength of recommendation	Certainty of evidence
Use TTE with tissue Doppler to assess LV systolic and diastolic function.^217,220,221^	Strong	High
Use TTE with GLS analysis for early assessment of LV dysfunction.^223,225,226^	Weak	Moderate
Detect pulmonary hypertension through TTE by estimating tricuspid regurgitation jet velocity.^227,228^	Strong	High
Use exercise stress echocardiography to assess pulmonary pressure in patients with dyspnea without pulmonary hypertension in TTE.^232,233^	Weak	High
Use TTE with TAPSE, S' and RV GLS and RV FAC measurement to monitor RV systolic function.^190,230^	Strong	High
Use TTE with tissue Doppler to assess treatment response.	Weak	Low
Use CMR LE techniques, T1 and T2 mapping to diagnose cardiac involvement and prognostic stratification in patients with systemic sclerosis.^221^	Weak	Moderate
Use myocardial perfusion scintigraphy to detect myocardial ischemia.^244–249^	Strong	High
^18^F-FDG PET/CT to assess myocardial inflammation.^252^	Weak	Low
Use ^123^I-metaiodobenzylguanidine myocardial scintigraphy to assess sympathetic function.^251^	Weak	Low

^18^F-FDG: [18 F]-fluorodeoxyglucose; CMR: cardiac magnetic resonance imaging; CT: computed tomography; FAC: fractional area change; GLS: global longitudinal strain; LE: late enhancement; PET: positron emission tomography; RV: right ventricle; TTE: transthoracic echocardiography; TAPSE: tricuspid annular plane systolic excursion.

Annual echocardiographic evaluation is recommended (including myocardial strain analysis, if available), cardiac magnetic resonance according to clinical indication every 2–3 years in high-risk or symptomatic patients, and myocardial scintigraphy only in selected cases.^253–255^

**Key messages**

- Cardiovascular impairment is common and is a leading cause of mortality in systemic sclerosis.
- TTE is essential for assessing cardiac function and screening for PH.
- Strain assessment with speckle-tracking echocardiography can detect early myocardial dysfunction, even with preserved EF.
- RV assessment in patients with systemic sclerosis and PH is essential for therapeutic management and risk stratification.
- Although TTE is important for PH screening, right heart catheterization is the gold standard for diagnosis.
- Stress echocardiography can identify diastolic dysfunction, HFpEF, and PH.
- Regular follow-up with echocardiography is important to guide treatment and monitor complications.
- CMR can be used to detect cardiac involvement in patients with active systemic sclerosis.
- In systemic sclerosis, morphofunctional assessment and tissue characterization with CMR is valuable for diagnosis and prognosis.

### 4.3. Ankylosing Spondylitis

Ankylosing spondylitis (AS) is a chronic and progressive seronegative, inflammatory, and autoimmune spondyloarthropathy associated with human leukocyte antigen class I molecule B27. It is characterized by sacroiliac joint and spine involvement. The disease is also associated with enthesitis, uveitis, inflammatory bowel disease, and psoriasis.^256–258^

Patients with AS have a higher risk of cardiovascular diseases than the general population, including aortic and mitral valve insufficiency, aortic disease, HF, involvement of the conduction system, and CAD.^257,259^

Myocardial and valvular involvement is explained by the inflammatory and fibrotic process triggered by disease activity.^257,259^ Fibroblast activation and platelet aggregation have been demonstrated specifically in the aortic valve, resulting in valvular fibrosis.^258^

Table 12 shows the pathophysiology of cardiac and aortic involvement and its prevalence in individuals with AS.

**Table 12:** Cardiac and aortic involvement in ankylosing spondylitis^257,260–262^

Structure	Pathophysiology	Prevalence
Aortic root and proximal ascending aorta	Inflammation (aortitis), resulting in stiffness and dilation.	20-60%
Aortic valve	Inflammation and fibrosis, with thickening and retraction of the valves.	40%
Mitral valve	Inflammation and fibrosis, with thickening of the mitral-aortic junction, resulting in reduced mobility or incomplete/asymmetrical coaptation of the cusps.	50%
Myocardium	Inflammation and fibrosis, compromising left ventricle diastolic filling.	ND
Conduction system	Extension of subaortic fibrosis, including involvement of the atrioventricular node, proximal His bundle and bundle branches and fascicles, resulting in conduction disturbances.	ND

ND: no data.

Echocardiography is the imaging modality of choice for initial assessment and long-term follow-up of valvular heart disease. It is also the ideal tool for assessing diastolic function.^3^

Through LV GLS assessment, echocardiography can also show subclinical systolic dysfunction, revealing myocardial involvement, which may be present from the early stages of AS.^259^

In addition to evaluating ventricular function and heart valves, CMR can characterize myocardial tissue, demonstrating the presence of edema and/or fibrosis. In AS, myocardial edema can be observed, which is associated with disease activity, and focal LE, reflecting myocardial fibrosis.^257^ CMR can identify global LV dysfunction and focal areas of LE as definitive markers of cardiac involvement. ECV, quantified through myocardial T1 mapping, shows an association with the degree of disease activity, highlighting its potential as a biomarker for monitoring disease activity.^257^ Both CMR and CT can assess the aorta to detect dilation, stenosis, and signs of inflammation.

Although AS is associated with premature atherosclerosis and an increased risk of cardiovascular events, little has been published on the role of nuclear medicine in this scenario.

A study of 24 patients with AS concluded that, in young individuals, ^18^F-FDG PET/CT can show vascular wall inflammation and reduced inflammatory activity under the effect of statins.^256^ A study of 28 patients with AS used MPS with ^99m^Tc-sestamibi, finding altered segmental motility through gated SPECT imaging in 8 patients, which is suggestive of microvascular dysfunction.^257^

Given the scarcity of evidence, no recommendations can be made regarding nuclear medicine methods specifically in the context of AS. However, this inflammatory disease shares pathophysiological factors with other systemic diseases, such as rheumatoid arthritis (RA) and SLE, in which MPS and ^18^F-FDG PET/CT have been used more extensively and have more established evidence and indications (Table 13).

**Table 13:** Recommendations on multimodal imaging for ankylosing spondylitis assessment

Recommendation	Strength of recommendation	Certainty of evidence
Use echocardiography to determine aortic root and proximal ascending aorta involvement.^258–260^	Strong	Low
Use echocardiography to assess the mitral and aortic valves.^258–260^	Strong	Low
Use echocardiography to assess diastolic function.^258–265^	Weak	High
Use echocardiography to determine LV global longitudinal strain and detect subclinical dysfunction.^258–265^	Weak	Moderate
Use CMR to determine aortic root and ascending aorta involvement.^257^	Weak	Low
Use CMR to assess the mitral and aortic valves in patients with echocardiographic limitations and/or to determine the timing of valve intervention.^257^	Weak	Low
Use CMR to assess LV function in patients with echocardiographic limitations.^257^	Weak	Low
Use CMR for myocardial tissue characterization, with myocardial extracellular volume as a marker of disease activity.^257^	Weak	Low
Use ^18^F-FDG PET/CT to assess vascular inflammation.^263^	Weak	Low
Use MPS to detect myocardial ischemia.^264^	Weak	Low

^18^F-FDG PET/CT: 18F- fluorodeoxyglucose positron emission tomography/computed tomography; CMR: cardiac magnetic resonance imaging; LV: left ventricle.

Currently, annual echocardiographic evaluation is recommended (including myocardial strain analysis, if available), cardiac magnetic resonance according to clinical indication every 2–3 years in high-risk or symptomatic patients, and myocardial scintigraphy only in selected cases. Cardiac imaging examinations in asymptomatic and clinically stable patients should not be performed at intervals shorter than one year, except in the presence of clinical findings.^1,257,266–269^

**Key messages**

- Patients with AS have a higher risk of cardiovascular diseases, including aortic and mitral valve insufficiency, aortic disease, HF, conduction system impairment, and CAD.
- TTE is the method of choice for initial assessment and long-term follow-up of valvular heart disease and diastolic function.
- LV GLS analysis may indicate subclinical systolic dysfunction, which can be present in the early stages of AS.
- In addition to evaluating ventricular function and heart valves, CMR can characterize myocardial tissue, demonstrating the presence of edema and/or fibrosis.
- Myocardial ECV is associated with the degree of disease activity, highlighting its potential as a biomarker for monitoring disease activity.
- CMR and CT are the tests of choice for thoracic aorta assessment.

### 4.4. Rheumatoid Arthritis

RA, an autoimmune, inflammatory, chronic disease with periods of remission and relapse, mainly affects the joints of the hands, wrists, feet, and knees. For reasons not yet fully understood, the immune system begins to attack the body's own tissues, especially the synovial membrane that lines the joints, causing pain, swelling, stiffness, and, over time, deformities and joint destruction. The disease can also affect other organs and systems such as the lungs (rheumatoid nodules and pulmonary fibrosis), the eyes (scleritis and keratoconjunctivitis), the skin, and blood vessels (vasculitis).^265^ In the heart, the main manifestations of acute RA are pericarditis, myocarditis, and endothelial injury, leading to increased cardiovascular risk.^270^

The diagnosis is clinical, based on signs and symptoms, supported by laboratory and imaging tests, including rheumatoid factor, anti-cyclic citrullinated peptide autoantibody levels, erythrocyte sedimentation rate, C-reactive protein, radiography, and ultrasound for joint damage.. Although joint manifestations are the main clinical focus, cardiovascular complications, which often occur subclinically, are a major cause of morbidity and mortality in these patients.^271^

Thus, echocardiography is a fundamental diagnostic tool in the evaluation of patients with RA.

TTE allows for a noninvasive, safe, and effective assessment of cardiac structures and is indicated in patients with RA for detecting early changes such as diastolic dysfunction, pericardial thickening and effusions, valvular heart disease (especially aortic and mitral regurgitation), and alterations in ventricular contractility. Tissue Doppler echocardiography also contributes to a more sensitive assessment of myocardial function, even in the absence of evident clinical symptoms.^272^

Transesophageal echocardiography may also be indicated in more specific cases, when endocarditis or more detailed valve involvement is suspected, particularly in patients with new heart murmurs or unexplained embolisms. Thus, regular echocardiographic assessment can aid in the early diagnosis of cardiac complications, influence therapeutic management, and improve prognosis (Figure 16).^273^

**Figure 16:** Transthoracic echocardiogram of a patient diagnosed with rheumatoid arthritis, showing mild aortic valve thickening in the parasternal longitudinal (A) and transverse (B) views with mild regurgitation on color Doppler (C) and (D).

In RA, several prognostic echocardiographic parameters have been identified, meaning they are associated with a higher risk of cardiovascular events, worse clinical outcomes, or mortality. These parameters allow for the diagnosis of subclinical cardiac involvement, facilitating earlier and more effective interventions.^270^

LVEF < 50% is associated with worse prognosis, but it is a late finding in most cases. Grade II or higher diastolic dysfunction, which is linked to an increased risk of HF and cardiovascular events, is prevalent even in early stages of acute RA, especially in women.

Patients with RA often present with reduced GLS, even when LVEF is preserved, indicating early myocardial damage, possibly related to chronic inflammation, fibrosis, or microangiopathy.^274^ Studies have shown a correlation between inflammatory marker levels (such as C-reactive protein and tumor necrosis factor-α) and GLS changes.^275^ This suggests that GLS may reflect the cardiovascular impact of inflammatory activity in RA, being useful both in baseline assessment and longitudinal monitoring. Reduced GLS in asymptomatic patients with RA could indicate a higher risk of future cardiovascular events, aiding in risk stratification and decisions about early intervention, such as intensified disease control or the use of cardioprotective agents. RV and atrial function, which can be affected by RA, can be assessed with GLS. This is particularly useful in cases with suspected pulmonary hypertension or chronic pericardial involvement.^276^

A meta-analysis demonstrated that increased myocardial mass is associated with progressive myocardial dysfunction and a higher risk of HF. In RA, it can occur even without hypertension, reflecting chronic inflammation.^277^

Another meta-analysis investigated case-control studies on valve and pericardial involvement in RA, finding that pericardial thickening or effusion is common in patients with long-standing RA, indicating chronic or acute pericarditis, even if asymptomatic. Persistent findings are associated with greater disease activity and the risk of complications, such as tamponade or constrictive pericarditis.^278^

Assessing pulmonary artery systolic pressure through tricuspid regurgitation velocity (> 35-40 mm Hg) also has prognostic value and is associated with increased mortality in RA, possibly reflecting advanced lung disease.^272^ Furthermore, RV dysfunction, whether assessed by traditional methods (TAPSE, s’, fractional area change) or RV GLS, is predictive of worse outcomes, especially in the presence of PH. A recent speckle-tracking study found that pulmonary ventriculo-arterial coupling can be used as a marker of early myocardial injury in RA.^279^

Three-dimensional echocardiography is a significant technological advancement in cardiac evaluation, offering real-time images with greater accuracy and anatomical detail than 2D echocardiography. In RA, this modality can provide specific benefits for both morphological and functional cardiac evaluation, especially regarding common subclinical cardiovascular manifestations in this patient population. Three-dimensional echocardiography provides realistic images of the valves, facilitating assessment of thickening, retraction, calcification, and functional changes.^280^

Several observational studies have also found increased cardiovascular risk in RA. A recent meta-analysis validated the use of carotid intima-media thickness as a marker of elevated cardiovascular risk in patients with RA.^281^ Carotid intima-media thickness is significantly higher in individuals with RA than healthy controls, reinforcing the role of vascular ultrasound, a widely available, low-cost method that allows early detection of plaques and thickening of the intima-media layer.^2,211,212,282,283^ Thus, vascular ultrasound is recommended as a screening method for subclinical carotid and femoral atherosclerosis.

Although echocardiography remains the initial examination of choice, cardiac CT and CMR broaden the assessment of cardiovascular involvement in acute RA, especially when greater anatomical precision, tissue characterization, or objective quantification of changes is needed, such as in subclinical cardiovascular disease, suspected myocarditis or fibrosis, early atherosclerotic risk assessment, and in cases where echocardiography is inconclusive. These examinations are particularly valuable in specialized centers and can contribute to more precise therapeutic decisions.^284^

Several studies have demonstrated the usefulness of CMR in early characterization of myocardial changes, even in patients newly diagnosed with RA and without a history of established cardiovascular disease. A recent study showed elevated T1 and ECV values in patients with active RA and the presence of autoantibodies, suggesting inflammation and early myocardial remodeling.^285^ Elevated T2 mapping values, suggestive of myocardial edema, are also found in patients with RA associated with disease activity.^286^ Furthermore, there is evidence that immunobiological treatment can positively affect the functional parameters of CMR LV assessment, reducing myocardial mass and improving LVEF.^287–289^

In addition to traditional risk factors, patients with RA have increased cardiovascular risk, partly due to chronic systemic inflammation. Cardiac CT, including coronary calcium score assessment and CT angiography, has proven an important tool in the early risk stratification and monitoring of subclinical atherosclerosis progression. A recent study demonstrated that patients with RA have significantly higher coronary calcium scores than healthy controls, highlighting the importance of cardiac CT in the early assessment of subclinical atherosclerosis.^290^ Furthermore, cardiac CT can also detect valve and artery calcification more frequently in these patients than in individuals without autoimmune diseases.^291^

The progression of coronary atherosclerosis in patients with RA also appears to be associated with the degree of cumulative inflammation, cardiovascular risk factors, and drug exposure. A prospective study using CT angiography found that patients with higher inflammatory activity and lower clinical control had greater atherosclerotic plaque progression, even after adjusting for traditional risk factors.^292^

However, therapeutic strategies aimed at rigorous inflammatory control have proven effective in reducing cardiovascular risk. Randomized clinical trials comparing immunomodulators have demonstrated that, in addition to controlling joint activity, they had a positive impact on vascular outcomes, such as reduced arterial inflammation and adequate control of the lipid profile.^293,294^ These effects can be monitored through noninvasive imaging methods, such as cardiac CT, and techniques associated with vascular inflammation detection, such as ^18^F-FDG-PET.

These findings highlight the relevance of CMR and cardiac CT as diagnostic and prognostic tools in patients with RA. They offer a detailed morphofunctional assessment and show potential in treatment response monitoring. The routine incorporation of CMR and cardiac CT into cardiovascular screening strategies in RA may allow for earlier and more individualized interventions.^295^

Nuclear medicine can contribute to the investigation of pericarditis and myocarditis in patients with RA, demonstrating the disease's inflammatory activity through ^18^F-FDG-PET/CT. The inflammation of large and medium-sized blood vessels and its relationship to cardiovascular risk has also been assessed with ^18^F-FDG PET/CT and PET/MR. Another application is myocardial ischemia assessment by myocardial perfusion scintigraphy or PET/CT using perfusion radiotracers.^2^

An interesting study found pathological uptake of ^18^F-FDG in the myocardium in 39% of individuals with RA. This finding correlated with clinical and laboratory criteria of disease activity, suggesting that the imaging method could be considered a marker of subclinical myocardial inflammation. The examination was repeated 6 months after treatment, showing a significant reduction in uptake in those who used tumor necrosis factor inhibitors, indicating adequate treatment response.^296^ It is important to mention that abnormal myocardial ^18^F-FDG results are not characteristic of any specific process; they only identify the presence of active inflammation. Definitive diagnosis depends on the integration of clinical, laboratory, and other imaging information, as well as the possibility of endomyocardial biopsy in selected cases, depending on the context.

Reports indicate that patients with active RA have increased ^18^F-FDG uptake in the carotid and aortic arteries, which is a sign of vascular inflammation. There is also evidence that RA treatment can reduce uptake intensity.^297,298^ However, it has also been shown that patients with chronic RA may continue to show signs of increased vascular inflammatory activity, even during remission.^299,300^

The most significant evidence regarding the impact of RA in the context of coronary artery disease demonstrated a reduction in coronary flow reserve on PET/CT with rubidium-82 or N-13-ammonia in patients with RA, which is similar to diabetics without a prior CAD diagnosis. In follow-up studies of up to 10 years, the mortality rate was higher in patients with altered coronary flow reserve. The results corroborate that RA, like diabetes, is a determinant of microvascular dysfunction and is associated with a higher risk of cardiovascular and all-cause mortality.^301^ However, these radiopharmaceuticals are not produced in Brazil. In our country, it is possible to assess coronary flow reserve through myocardial scintigraphy. However, no studies have specifically used this method in RA.

In all patients with RA, coronary risk should be assessed every 5 years or when changes occur in symptoms or anti-inflammatory therapy. Noninvasive tests to investigate myocardial ischemia and/or atherosclerosis may also be useful in this context (Table 14).^2^

**Table 14:** Recommendations on echocardiographic parameters, magnetic resonance imaging, and cardiac computed tomography for cardiovascular assessment in rheumatoid arthritis

Recommendation	Strength of recommendation	Certainty of evidence
Use TTE for GLS analysis to detect early subclinical ventricular dysfunction.^286^	Weak	Moderate
Use TTE to calculate LVEF for global systolic function analysis.^274^	Strong	High
Use TTE to calculate the myocardial performance index in analysis of systolic and diastolic function.^287^	Weak	Moderate
Use TTE to analyze early LV diastolic dysfunction by evaluating E/A waves and the E/e' ratio.^274^	Strong	Moderate
Use TTE to analyze LA diameter, which reflects chronic diastolic dysfunction.^274^	Strong	Moderate
Use TTE to analyze indexed LA volume, which reflects chronic diastolic dysfunction.^288^	Weak	Moderate
Use TTE to analyze pericardial thickening/effusion, which indicates inflammatory activity and the risk of complications.^279^	Weak	Moderate
Use TTE to analyze RV GLS, which may be reduced in the presence of inflammation or pulmonary hypertension.^280^	Weak	Low
Use TTE to analyze LV mass to investigate cardiac remodeling due to chronic inflammation.^274,278^	Weak	Moderate
Use TTE to calculate pulmonary artery systolic pressure to investigate RA-associated pulmonary hypertension.^274^	Strong	High
Use TTE to assess valve thickening/reflux, an identifier of inflammatory valvular heart disease.^273^	Weak	Moderate
Use carotid Doppler ultrasound to assess intima-media thickness and cardiovascular risk.^284,289^	Strong	High
Use three-dimensional echocardiography to assess LV, LVEF, and valve volume.^281^	Weak	Moderate
Use CMR in prognostic assessment of cardiovascular changes after therapeutic interventions.^290,291^	Weak	Moderate
Use cardiac computed tomography to assess atherosclerotic disease.^285,292^	Strong	High

RV: right ventricle; LV: left ventricle; TTE: transthoracic echocardiogram; LVEF: LV ejection fraction; CMR: cardiac magnetic resonance imaging; GLS, global longitudinal strain.

The frequency of cardiac imaging examinations in patients with RA should be guided by symptoms, clinical course, risk factors, and therapeutic changes, with no protocol for periodic screening in asymptomatic patients.^2,211,212^

**Key messages**

- The main cardiac manifestations of RA are pericarditis, myocarditis, and endothelial injury, leading to increased cardiovascular risk.
- Reduced LVEF (< 50%) is associated with worse prognosis, but it is a late finding in most cases.
- Grade II or higher diastolic dysfunction is linked to an increased risk of HF and cardiovascular events.
- GLS is often reduced in patients with RA, even with preserved LVEF, indicating early myocardial damage.
- Ventricular hypertrophy, or increased myocardial mass, is associated with progressive myocardial dysfunction and a higher risk of HF, and may occur even in the absence of arterial hypertension, reflecting chronic inflammation.
- Nuclear medicine techniques, such as 18F-FDG-PET/CT, can be used in patients with RA to assess myocardial, aortic, and carotid vascular inflammation, as well as to detect myocardial ischemia using MPS.
- Pericardial thickening or effusion is common in patients with long-standing acute RA, indicating chronic or acute pericarditis, and persistent findings are associated with increased disease activity and complications.
- CMR morphofunctional assessment and tissue characterization are useful for detecting myocardial involvement, and the protocol should incorporate cinematic MRI, LE, and T1/T2 mapping techniques.
- When indicated, atherosclerotic disease can be accurately investigated through coronary CT angiography and calcium score.

### 4.6. Takayasu's Arteritis

Takayasu's arteritis (TA), a rare form of inflammatory vasculitis, was initially described in 1908. It preferentially affects large blood vessels, especially the aorta and its branches, and may also affect the coronary and pulmonary arteries to a lesser extent.^302^ Its incidence is higher in women and individuals < 40 years of age.^303^

The most common clinical manifestations in the initial phase of the disease include fever, arthralgia, myalgia, night sweats, loss of appetite, and weight loss. However, diagnosis usually occurs in more advanced stages, when vascular fibrosis is already observed, resulting in peripheral pulse changes and blood pressure differences > 10 mm Hg between limbs. At this stage, a murmur and thrill along the arteries may also be detected. In addition, the patient frequently presents with hypertension, which is one of the most common causes of diagnosis of this condition.^304^ Therefore, all young patients with hypertension of unknown cause should be investigated for TA.

The American College of Rheumatology diagnostic criteria^305^ are described below. Patients must present at least 3 of the 6 indicated parameters:

1. Age < 40 years.
2. Decreased brachial pulse.
3. Limping in the extremities.
4. Difference of 10 mm Hg in systolic blood pressure between the upper limbs.
5. Bruits over the aorta and subclavian arteries.
6. Angiographic changes in the aorta and its main arches.

Comprehensive assessment with laboratory tests is recommended, especially markers of inflammatory activity (polymerase chain reaction and erythrocyte sedimentation rate), which can be useful in disease monitoring. Although diagnosis is based on clinical criteria, PET/CT has been used to assist to confirm diagnosis and monitor inflammatory activity, whether in treatment response assessment or in suspected inflammatory process relapse, especially when the symptoms are nonspecific and other tests are inconclusive.^306,307^

Therefore, in patients with TA, assessment of the entire aorta is recommended as early as possible by magnetic resonance imaging or computed tomography, with or without 18F-FDG, in order not to delay immunosuppression (level of evidence 1C).^307–309^ Tomography associated with ^18^F-FDG has high specificity in large cell arteritis and may be beneficial in patients with TA for determining inflammatory activity as well as the degree of vascular involvement (Figure 17).^310^ Activated inflammatory cells have an affinity for ^18^F-FDG, a glucose analogue used in PET/CT scanning, which can detect inflammatory and/or infectious diseases and is applicable in the context of TA.^306,311^

**Figure 17:** Patient with suspected Takayasu arteritis, presenting paresthesia in the right upper limb during exertion. Doppler ultrasound of the upper limbs demonstrated right subclavian artery stenosis. A coronal PET image with 18F-FDG shows heterogeneous uptake of the radiotracer in the ascending aorta and carotid arteries, suggesting vasculitis.

PET/CT has advantages over other imaging methods, including combining anatomical and functional images, estimating the intensity of the inflammatory process through the degree of radiotracer uptake, obtaining whole-body images in a single examination to identify the extent of the involvement, no impairment to renal function when the examination is performed without iodinated contrast media, and a low incidence of allergic reaction to the radiopharmaceutical. Potential disadvantages include high cost, low availability, and the use of ionizing radiation.^312^

A 2018 meta-analysis (10 studies; 301 patients) found a sensitivity of 81% and a specificity of 71% for identifying TA compared to clinical criteria.^307^ Another meta-analysis investigated the efficacy of PET/CT for monitoring treatment response in large vessel vasculitis, including giant cell arteritis (4 studies; 111 patients), finding a sensitivity of 77% and specificity of 71% for distinguishing active disease and remission.^308^ Some limitations that affect the diagnostic accuracy of PET/CT in TA are the small number of included patients, the diverse inclusion criteria, and the different image parameters analyzed in the studies.^313–315^

CT is less invasive than catheter arteriography and can clearly demonstrate thickening of the vessel wall, which may not be visible with catheterization^316^ (Figure 18). MRI can also be used to determine the degree of vessel wall involvement, with its main advantage over CT being the lack of ionizing radiation, which can be beneficial for monitoring young patients due to the need for frequent repeat assessments of the aorta.^317^ Both CT (with or without ^18^F-FDG) and MRI reveal lesions from inflammation due to the disease, a tortuous aorta, alternating segments of stenosis with dilation or occlusion, and aortic branch involvement, especially the carotid, left subclavian, and mesenteric arteries.^318^ Disease activity can be monitored through inflammatory function tests and imaging studies (MRI or CT angiography).

**Figure 18:** Echocardiographic (A, B, and D) and thoracic aorta CT angiography (C) images of a patient diagnosed with Takayasu's arteritis. The patient presents with significant concentric myocardial hypertrophy (A and B) secondary to marked stenosis of the aortic arch, descending thoracic aorta, as well as occlusion of the brachiocephalic trunk and marked stenosis of the left common carotid artery and left subclavian artery (C). Continuous Doppler ultrasound showed a maximum gradient of 202 mm Hg in the suprasternal view of the descending aorta. Image provided by Dr. Viviane Hotta.

Although using vascular ultrasound to assess thoracic aorta involvement in TA involves technical limitations, the method is important for detecting involvement of the subclavian arteries, common carotid arteries (sparing of the carotid branches), vertebral arteries, and abdominal branches (Figure 19). In addition to aiding in the diagnosis of atrial fibrillation, vascular ultrasound can monitor disease progression and treatment response.^319^ During periods of inflammatory activity, vascular ultrasound can visualize arterial segments with concentric thickening, which are characterized by homogeneity and low or intermediate echogenicity and may involve hypoechoic areas related to edema and/or neovascularization.^320^ In addition to neovascularization, artery diameter increases > 10 mm suggest disease activity.^321^ Concentric thickening can lead to arterial obstructions and occlusions, including ischemia in target organs. However, when vascular ultrasound detects arterial segments with hyperechoic concentric thickening due to a greater presence of fibrotic tissue, there is a stronger correlation with chronic stages of TA.^319,322^

**Figure 19:** Stages of concentric thickening in Takayasu's arteritis. In a patient during the active phase of the disease, transverse (A) and longitudinal (B) B-mode sections demonstrating large concentric thickening of the common carotid artery wall with low echogenicity and echolucent intervals. Longitudinal B-mode sections demonstrating less pronounced and more echogenic thickening of the common carotid artery wall (C) with sparing of the internal branch (D). Image provided by Dr. Cláudia Maria Vilas Freire.

It is recommended that:

- Vascular ultrasound can be used to detect mural inflammation and/or luminal changes in the aortic branches of patients with suspected TA, but it has limited value for thoracic aorta assessment;
- Vascular ultrasound can be used for long-term monitoring of structural damage, particularly to detect stenosis, occlusion, dilation, and/or aneurysms. Both the screening frequency and the imaging method should be decided on a case-by-case basis.

Echocardiography, which plays a secondary role in clinical management, can be used to detect changes secondary to segmental stenosis of the aorta and its branches, arterial hypertension (ventricular hypertrophy) (Figure 18), and even the direct involvement of other vessels, such as the coronary arteries (up to 25% of patients may present with stenosis) and the pulmonary arteries, which may be dilated. Accelerated flow in the abdominal aorta (in the subxiphoid plane) and mitral or aortic valve insufficiency (which can occur in up to 55% of patients) may also be observed (Table 15).^323^

**Table 15:** Recommendations on the use of ^18^F-FDG PET/CT in Takayasu's arteritis

Recommendation	Strength of recommendation	Certainty of evidence
Use ^18^F-FDG PET/CT to diagnose active vascular inflammation and the intensity and extent of involvement.^307–309^	Weak	Low
Use ^18^F-FDG PET/CT to monitor treatment response.^307–309^	Weak	Low

^18^F-FDG PET/CT: 18F- fluorodeoxyglucose positron emission tomography/computed tomography.

Although not well established, the frequency of imaging examinations can vary from 3 to 6 months depending on disease activity and the clinical evaluation of individual patients.^316^ However, according to the European League Against Rheumatism (EULAR) recommendations, patients in remission should undergo vascular ultrasound, MRI, or CT annually, even in the absence of inflammatory activity after medication has been introduced, due to the possibility of subclinical vascular involvement.^308^

**Key messages**

- TA is a rare inflammatory vasculitis that frequently causes arterial hypertension and vascular fibrosis, resulting in bruits over the aorta and subclavian arteries, changes in peripheral pulses, and a blood pressure difference greater than 10 mm Hg between the limbs.
- In patients with TA, evaluation of the entire aorta is recommended using MRI or CT, with or without 18F-FDG.
- 18F-FDG PET has high specificity for GCA and may be beneficial in patients with TA for determining inflammatory activity as well as the degree of vascular involvement.
- CTA and MRA can be used to determine the degree of vessel wall involvement, demonstrating lesions related to TA, including a tortuous aorta with alternating segments of stenosis and dilation or occlusion, as well as involvement of aortic branches, particularly the carotid, left subclavian, and mesenteric arteries.
- Vascular ultrasound is useful for detecting involvement of aortic branches, such as the subclavian arteries and common carotid arteries, while allowing evaluation of the carotid branches, vertebral arteries, and abdominal branches.
- Vascular ultrasound can be used to monitor disease progression and staging, as well as therapeutic response.

## 5. Applications of multimodal cardiac imaging in storage diseases

### 5.1. Iron Overload Cardiomyopathy

The term iron overload cardiomyopathy was introduced to describe cardiomyopathy resulting from iron accumulation in the myocardium, primarily due to genetically-determined iron metabolism disorders or multiple transfusions.^324^ In addition to being a major cause of morbidity, iron overload cardiomyopathy is responsible for one-third of deaths in hereditary hemochromatosis and is a leading cause of death in other conditions associated with secondary iron overload.^325,326^ Iron overload can be primary or secondary (Table 16).

**Table 16:** Main conditions that lead to iron overload

Primary iron overload	Secondary iron overload	Other conditions
Hereditary hemochromatosis	Hereditary anemias	Chronic liver disease
➢ Type I: related to the HFE gene	Hemoglobinopathies	Friedreich ataxia
➢ Type II: juvenile	Thalassemia	Aceruloplasminemia
Subtype A: related to the HJV gene	Sickle cell anemia	Congenital atransferrinemia
Subtype B: related to the HAMP gene	Diamond-Blackfan anemia	Increased food intake
➢ Type III: Related to the TfR2 gene	Congenital dyserythropoietic anemia	
➢ Type IV: related to ferroportin	Sideroblastic anemia	
	Acquired anemias	
	Myelodysplastic syndromes	
	Myelofibrosis	
	Aplastic anemia	
	Leukemias	
	Myeloproliferative diseases	
	Stem cell transplantation	
	Chronic kidney disease	

The primary form of iron overload is called hereditary hemochromatosis, an autosomal recessive genetic disease resulting from mutations in the genes that encode proteins involved in iron metabolism, causing increased iron absorption by the intestinal epithelium and further disruption of its metabolism, which leads to iron accumulation and consequent damage to various organs.^327,328^

Secondary iron overload is generally caused by high parenteral iron administration. It is mainly observed in association with hereditary or acquired anemias dependent on repetitive blood transfusions, which saturate the cells of the reticuloendothelial system with iron, subsequently spreading to other parenchymal cells (Table 16).^329^

Iron deposition occurs initially in the ventricular myocardium, followed by the atrial myocardium. It also affects the conduction system, especially the atrioventricular node, but to a lesser degree than the functional myocardium.^329^ It should be noted that myocardial iron overload, although a triggering factor in the development of iron overload cardiomyopathy, is not the only mechanism involved. Iron overload cardiomyopathy is a specific type of cardiomyopathy with a complex pathophysiology involving additional immunoinflammatory and genetic factors that affect pathogenesis. Two phenotypes of iron overload cardiomyopathy have been identified:^325^

- Dilated phenotype: characterized by LV remodeling and leading to chamber dilation and reduced LVEF;
- Restrictive phenotype: characterized by diastolic LV dysfunction with restrictive filling, preserved LVEF, pulmonary hypertension, and subsequent RV dilation.

These phenotypes are followed by several other manifestations, including conduction system abnormalities, tachyarrhythmias, and perimyocarditis. If the cause of iron overload persists and no adequate iron chelation therapy is initiated, LV remodeling occurs in most patients with iron overload cardiomyopathy, in advanced stages leads to LV dilation and reduced LVEF (dilated phenotype).^325,330^

Approximately 10% of cases are characterized by severe iron overload and mild LV dysfunction, particularly in advanced stages, PH, RV dilation, and right HF without LV remodeling and with preserved LVEF, even in the final stages (restrictive phenotype).^330–332^

Iron overload cardiomyopathy, which presents distinct clinical characteristics and requires a specific diagnostic and therapeutic approach, should be considered in any patient with HF of unknown etiology. Patients may be asymptomatic in the early stages of the disease, although once HF develops, rapid deterioration follows.

In the initial assessment patients with iron overload, echocardiography is the main modality used to screen for heart disease, although its findings are nonspecific.^325^ Abnormalities in ventricular systolic and diastolic function, as well as pericardial and valvular involvement, can be detected. In early stages, slight dilation of the cardiac chambers and a minimal reduction in LVEF may be evident. In these patients, analysis of LV GLS can help confirm incipient myocardial dysfunction (Figure 20).

**Figure 20:** Echocardiographic images of a 25-year-old patient with hereditary hemochromatosis. Left ventricular volumes and ejection fraction assessed by Simpson's method in 2-dimensional echocardiography (A) and 3-dimensional echocardiography (B and C) showing systolic function at the lower limit of normal and slight ventricular dilation. There is a slight reduction in left ventricular global longitudinal strain (-17.1%) and global circumferential strain (-24.2%) (D and E). LVEF: left ventricular ejection fraction; GCS: global circumferential strain; GLS: global longitudinal strain; RV: right ventricle; EDV: left ventricular end-diastolic volume; LV: left ventricle; ESV: left ventricular end-systolic volume.

High cardiac output with chamber dilation, eccentric LVH, and normal or increased LVEF may also be observed.^333^ It should be noted that diastolic LV dysfunction and reduced LVEF can be masked by an anemia-induced high output state in patients with hemoglobinopathies and other hematological conditions. Thus, a pseudonormal pattern can often be found in transmitral flow,^325^ which can be exposed by refining the diastolic function analysis through investigation of pulmonary vein flow patterns or mitral annulus movement in tissue Doppler imaging.

Although sophisticated imaging methods, such as LV GLS, may be more sensitive markers of subclinical LV dysfunction, their results have been controversial in populations with hemochromatosis.^334,335^ prospective studies are needed to better evaluate subclinical LV dysfunction in individuals with chronically-treated hereditary hemochromatosis.

Although echocardiography can identify the consequences of iron overload on myocardial structure and function, it cannot accurately predict myocardial iron levels. Nevertheless, it is a simple method for screening asymptomatic patients and monitoring patients with known pathologies.^325^

CMR with T2* relaxometry is shortened in the presence of iron due to the metal's paramagnetic effect. T2* values < 20 ms indicate clinically relevant overload, and values < 10 ms are associated with a high risk of cardiac dysfunction and serious arrhythmias.^336,337^ There is a strong correlation between T2* values and iron concentration measured in biopsies, validating CMR as a reliable method for noninvasive assessment.^338,339^ The entire ventricle can also be mapped with the technique, avoiding sampling errors that can occur during biopsy.

Another promising tool is T1 mapping. In established cardiac iron overload, T1 and T2* are concordant. In CSF, native T1 values are reduced. However, in the 20–30 ms T2* range, T1 mapping appears to detect iron earlier.^340–342^

In advanced cases, LE can identify areas of myocardial fibrosis, which are associated with worse prognosis.^340–342^

In advanced cases, myocardial late enhancement can identify areas of myocardial fibrosis, which are associated with a worse prognosis.^343^ Although fibrosis is not a common finding in primary hemochromatosis, its presence in patients with secondary iron overload (such as thalassemias) may indicate more advanced disease.

Another significant advantage of CMR is its ability to monitor treatment response. Therapies such as phlebotomy or iron chelation (with desferrioxamine, deferasirox, or deferiprone) can reduce myocardial iron load, which can be monitored serially through CMR. Longitudinal thalassemia studies have shown that post-therapy improvement in T2* values is associated with cardiac function recovery and fewer adverse events.^331,344^ The main CMR changes in patients with hemochromatosis are described in Table 17.

**Table 17:** Main changes in hemochromatosis assessed by CMR

Morphology	Late enhancement	T1 map	T2 or T2*	ECV
Symmetrical LVH	Rare in the early stages	Reduced	T2* reduced	N/A

CMR: cardiac magnetic resonance imaging; ECV: extracellular volume; LVH: left ventricular hypertrophy; N/A: not applicable.

LV dysfunction and PH can lead to impaired RV function.^325^ More recently, CMR data analysis in patients with thalassemia major showed that RVEF progressively decreased with increasing myocardial iron load, following a similar pattern to LVEF.^345^

Finally, it should be noted that serum ferritin levels do not correlate with myocardial iron overload severity, ie, high myocardial iron deposition can occur despite low serum ferritin levels.^346^ Therefore, diagnosis of iron overload cardiomyopathy is based on evidence of heart disease, particularly when LV diastolic dysfunction^324,347^ with restrictive LV filling or remodeling with chamber dilation and reduced LVEF co-occurs with iron overload (serum ferritin 300 ng/mL, transferrin saturation 55%) and cardiac siderosis (cardiac T2* < 20 ms), as outlined in Figure 21.

**Figure 21:** Diagnosis flowchart for iron overload cardiomyopathy. Diagnosis requires: (1) iron overload (serum ferritin > 300 ng/mL, transferrin saturation > 55%); (2) cardiac siderosis (T2* < 20 ms); and (3) evidence of heart disease. LV: left ventricle; LVEF: LV ejection fraction; RV: right ventricle. Adapted from Kremastinos & Farmakis.^331^

Table 18 presents practical recommendations (following the GRADE system) on echocardiography in patients with known or suspected iron overload cardiomyopathy.

**Table 18:** Recommendations on echocardiography for patients with suspected or confirmed iron overload cardiomyopathy

Recommendation	Strength of recommendation	Certainty of evidence
Transthoracic echocardiography, including geometric and biventricular systolic function analysis, should be performed as a first-line assessment in suspected or confirmed iron overload conditions.^324,325,331,332^	Strong	Moderate
Transthoracic echocardiography with left-ventricular diastolic function analysis, including tissue Doppler imaging of the medial and lateral mitral annulus, should be performed as a first-line assessment for suspected or confirmed iron overload.^331,332,338^	Strong	Moderate
Global longitudinal strain can be used to diagnose subclinical left-ventricular systolic dysfunction in conditions of suspected or confirmed iron overload.^334,335^	Weak	Low
Transthoracic echocardiography should be performed every 6 to 12 months if the CMR T2* relaxation time is < 20 ms, and every 6 months or less if the patient becomes symptomatic.^326,331,334,336^	Weak	Moderate
CMR analysis of biventricular volumes and systolic function should be performed for suspected or confirmed iron overload.	Strong	Moderate
CMR measurement of T2* relaxation time should be performed in all patients with suspected or confirmed iron overload.^336–339^	Weak	Moderate
CMR with T1 relaxation time measurement can be performed in patients evaluated for suspected or confirmed iron overload.^340–342^	Weak	Low
Follow-up CMR measurement of T2* relaxation time should be performed in patients with iron overload who are undergoing treatment (chelating agents and phlebotomy), since improved T2* values correlate with recovered cardiac function and fewer adverse events.^331,344^	Weak	Moderate

CMR: cardiac magnetic resonance imaging.

In patients with CSF, echocardiography should be performed every 6 to 12 months if the T2 relaxation time measured by CMR is less than 20 ms, and every 6 months or less if the patient becomes symptomatic. Periodic transthoracic echocardiography with assessment of diastolic function every 1–2 years is recommended, even in asymptomatic patients.^324,347^

**Key messages**

- Early LV diastolic dysfunction occurs in CSF and can be detected by echocardiography with tissue Doppler.
- If LV diastolic dysfunction and/or a reduction in the maximum tissue systolic velocity of mitral annulus motion is detected by echocardiography, T2* relaxation time should be measured in CMR.
- In all patients with idiopathic cardiomyopathy, T2* relaxation time should be measured in CMR.
- Myocardial iron concentration can be determined through the T2* relaxation technique in CMR.
- T1 mapping is complementary to T2* and can detect iron deposition at earlier stages, especially at T2* values between 20 and 30 ms.
- When monitoring therapy with phlebotomy or chelating agents, improved T2* values correlate with recovered cardiac function and fewer adverse events.

### 5.2. Fabry Disease

Anderson-Fabry disease (FD), a rare condition resulting from pathogenic variants in the *GLA* gene, leads to a deficiency of α-galactosidase, an enzyme responsible for the metabolism of glycosphingolipids (GL-3 and lyso-Gb3) in lysosomes. This results in the formation of cellular deposits that can be observed as early as intrauterine life. These deposits cause an intense inflammatory process and oxidative stress, evolving to cellular apoptosis and multisystem organ failure.^348^

Clinical manifestations depend on the genotype, since certain pathogenic variants determine the classic phenotype and others cause the alternate (late onset) phenotype. In the first subgroup, enzymatic activity < 5% determines very early symptoms in children as young as 2 years of age. Notable symptoms include the appearance of angiokeratomas, hypohidrosis, gastrointestinal disorders, corneal changes (verticillata), painful crises due to peripheral neuropathy, and proteinuria of varying intensity. Supraventricular and ventricular arrhythmias, exercise intolerance, and mild valve insufficiency may occur in these early stages. These alterations tend to worsen in adolescence in cases not treated with specific therapy, whether enzyme replacement or oral chaperone therapy. In adulthood, significant changes may occur in the renal system (renal failure), cardiac system (marked increase in myocardial thickness, arrhythmias, myocardial ischemia, and a hypertrophic cardiomyopathy pattern with HFpEF), and central nervous system (cerebrovascular disease), with rapid progression to advanced disease and the need for replacement therapies such as dialysis.^349,350^

However, due to the presence of residual levels of enzymatic activity, symptoms in the alternate phenotype manifest later, generally in adulthood, and are more restricted to organs such as the heart and kidney. Nevertheless, this rules out neither cardiomyopathy nor nephropathy, and the potential for progression is as severe as in the classic form. Thus, the cardiac and renal changes are the same as in the classic form.^349,350^

Definitive diagnosis is based on identifying the pathogenic variant associated with α-galactosidase activity, along with the presence of clinical manifestations, typical histological changes, elevated levels of specific biomarkers (Gb3 and lyso-Gb3), and a family history of the disease.^351^

The complexity of the disease's clinical presentation reinforces the need for multidisciplinary research, including medical geneticists and specialists such as cardiologists, neurologists, and nephrologists, among others. Therapy that integrates nursing care, nutrition, and psychology is also important for optimizing follow-up and improving quality of life.^352^

Cardiologists must play a leading role in this, since cardiovascular and renal manifestations are the main causes of FD morbidity and mortality in Brazil. Clinical complaints and abnormalities on complementary cardiological examinations can determine the stage of disease progression and provide diagnostic and prognostic information that indicates the need for specific treatment.^353^

Electrocardiography should be performed for the initial evaluation of cardiac involvement in FD and should be repeated annually. The most frequent electrocardiographic findings are a short PR interval, signs of LV overload, and a corrected QT interval < 440 ms.^354^ Echocardiography is fundamental, especially in asymptomatic individuals or in cases with nonspecific symptoms. Furthermore, its low cost and availability allow heart disease progression to be assessed in the index patient, as well as phenotypic diagnosis of potentially affected family members. The main cardiac imaging findings are described in Table 19, Figure 22 and Figure 23.

**Table 19:** Main findings regarding echocardiography and cardiac magnetic resonance imaging in Fabry disease

Echocardiography	CMR
LV thickness > 11 mm (women) or > 12 mm (men)	Symmetrical and concentric LV hypertrophy
Increased thickness of the papillary muscle	Subepicardial, inferolateral mesocardial, and septal LE
Diastolic dysfunction	Reduced T1 values (watch for pseudonormalization)
Altered segmental contractility (basal inferolateral segment)	Elevated T2 values
Reduced GLS and GRS (basal inferolateral segment)	

CMR: cardiac magnetic resonance imaging; GLS: global longitudinal strain; GRS: global radial strain; LE: late myocardial enhancement; LV: left ventricle; T1: longitudinal relaxation time; T2: transverse relaxation time.

**Figure 22:** Echocardiographic findings in Fabry disease. A) Parasternal longitudinal view showing increased LV wall thickness. B) Apical 4-chamber view showing the endocardial binary signal (myocardial birefringence aspect) in the inferior septal wall. C) Doppler transmitral flow velocity pattern showing significant diastolic dysfunction (E/A ratio > 2). D) Apical 4-chamber view showing mild mitral regurgitation. E) Apical 3-chamber view showing increased papillary muscle thickness (yellow arrow). F) Representation of segmental longitudinal strain curves and parametric mapping showing reduced segmental strain in the basal segment of the inferior wall, a change characteristic Fabry disease.

**Figure 23:** Cardiac magnetic resonance images of a patient diagnosed with Fabry disease. Cine MRI images show concentric biventricular myocardial wall thickening. Native T1 mapping sequences demonstrate reduced native T1 values (yellow arrow) and pseudonormalization of the T1 signal in an area of late gadolinium enhancement. Late gadolinium enhancement images demonstrate fibrosis in the inferolateral wall (yellow arrows). Increased T2 mapping values in the area of late gadolinium enhancement indicate the presence of edema and inflammation. Images provided by Dr. Sílvia Aguiar Rosa.

Increased LV and/or RV thickness (30%-40% of cases), usually in a concentric pattern and associated with preserved EF and diastolic function abnormalities, should raise suspicion of FD. The incidence of these changes increases with age, and the prevalence is similar in men and women.^354,355^

The use of 3D imaging techniques increases the accuracy of cardiac mass without the need for geometric inferences. In young individuals, abnormal diastolic function that is unexplained by coexisting diseases is usually the earliest echocardiographic manifestation of the disease and precedes hypertrophy. LVEF usually remains preserved throughout the natural course of the disease, and worsening systolic function is associated with the appearance of fibrosis and/or alterations such as renal dysfunction. The presence of endocardial binary sign shows a histological correlation with endocardial Gb3 deposits. These deposits can also affect valve tissue, causing mild valvular regurgitation. Ectasia of the initial portion of the thoracic aorta may occur, which generally does not evolve into aneurysmal forms with the potential for dissection.

GLS analysis aids in differential diagnosis from phenocopies and can identify segmental contractility changes in the basal segment of the inferolateral wall that may go unnoticed in conventional echocardiographic evaluation (Figure 22). Segmental strain dysfunction may indicate fibrotic changes, which are markers of chronic disease and poor cardiovascular prognosis. RV free wall strain may be reduced, even when other systolic function markers, such as TAPSE and S-wave velocity, are normal.^356–358^

In addition to characterizing myocardial tissue and providing prognostic information, CMR can be useful in treatment response assessment. CMR allows precise quantification of ventricular mass and can detect specific patterns of myocardial involvement that aid in differential diagnosis from phenocopies.^359–362^ One of the most significant contributions of CMR to FD management is the ability to detect and quantify myocardial fibrosis through LE. The characteristic LE pattern occurs predominantly in the basal inferolateral LV segments, typically mesocardial or subepicardial, which contrasts with the transmural enhancement pattern observed in ischemic cardiomyopathy or the diffuse subendocardial pattern seen in amyloidosis. This peculiar distribution of fibrosis reflects the underlying pathophysiological process of the disease, which results from the accumulation of globotriaosylceramide in cardiomyocytes and endothelial cells.^362^ The presence and extent of LE have important prognostic value, correlating with the risk of ventricular arrhythmias and adverse cardiac events.^362^

Native T1 mapping has become another valuable tool in CMR assessment. Due to intracellular accumulation of sphingolipids, native T1 values are reduced in FD, which contrasts with the increase observed in most other cardiomyopathies (Table 20). Special care should be taken regarding T1 pseudonormalization in areas affected by LE. This finding is particularly useful in the differential diagnosis between other causes of LVH, being considered a marker of the disease. T2 mapping may show slightly increased overall values (Figure 23).^348^

**Table 20:** Recommendations on multimodal imaging for the diagnosis of Anderson-Fabry disease

Recommendation	Strength of recommendation	Certainty of evidence
TTE should be performed as a first-line imaging method in FD diagnosis and should be repeated annually or if new cardiac symptoms appear.^355,356^	Strong	High
Altered GLS, characterized by decreased myocardial strain in the basal segment of the inferolateral wall, is characteristic of FD and serves as a differential diagnosis for other diseases with a similar phenotype.^355,356^	Weak	Moderate
When first-degree relatives are diagnosed and genetic testing is not possible, echocardiography should be used for family screening.^355,357^	Weak	Moderate
CMR is the gold standard for cardiac investigation because it allows for better characterization of myocardial damage, provides prognostic information, and can assess treatment response.^358–360^	Weak	Low
^99m^Tc-sestamibi SPECT/CT can provide additional information about microcirculation.^360^	Weak	Low

^99m^Tc-PYP: 99m-technetium; FD: Anderson-Fabry disease; CMR: cardiac magnetic resonance imaging; CT: computed tomography; GLS: global longitudinal strain; SPECT: single-photon emission computed tomography; TTE: transthoracic echocardiogram.

CMR also plays a crucial role in assessing the response to enzyme replacement therapy or molecular chaperone therapy. Longitudinal studies have demonstrated that effective treatment can lead to stabilization or even regression of ventricular hypertrophy, as well as prevent the progression of myocardial fibrosis.^362^ CMR allows for objective and reproducible monitoring of these changes, providing quantitative parameters that aid in therapeutic decision-making. T1 mapping has proven especially sensitive to changes in myocardial globotriaosylceramide content during treatment and may serve as an early biomarker of treatment response. In addition to morphological assessment and tissue characterization, CMR provides valuable information about cardiac function and prognosis^356,362^ (Table 20).

Although its diagnostic and prognostic role is still under investigation, SPECT/CT can also provide information about microcirculation through the radiotracer ^99m^Tc-sestamibi. Complaints of ischemic chest pain associated with electrocardiographic changes and typical biomarkers are frequent in the absence of macrovascular coronary obstruction. Coronary flow reserve can also be assessed in these cases by CT or CMR.^360,361^

Table 20 presents the main recommendations on multimodal cardiac imaging assessment in FD according to the GRADE system.

The recommended frequency of cardiac imaging examinations in patients with FD is based on expert consensus and practices from reference centers. A publication from the University of Washington recommends annual echocardiography in men from 18 years of age and every 2 years in women aged 18 to 35 years; after 35 years of age, the frequency may be adjusted according to the clinical scenario.^350^

Multicenter studies and reviews suggest that, in clinical practice, echocardiography (including myocardial strain) should be performed every 6 to 12 months, especially in higher-risk or symptomatic patients, and may be performed earlier in cases of clinical worsening.^363^ Cardiac magnetic resonance is recommended every 1 to 2 years, depending on availability and disease stage.^335,362,363^

Thus, the frequency of imaging examinations should be individualized according to disease severity, symptoms, response to treatment, and availability of imaging modalities, with intensified follow-up in cases of higher risk or progression of cardiac involvement.^363,364^

**Key messages**

- The most typical echocardiographic findings in FD are increased myocardial thickness of the ventricular wall and papillary muscle, with preserved systolic function, diastolic dysfunction, binary endocardial appearance, and mild valvular regurgitation.
- Reduced GLS in the basal segment of the inferolateral wall is characteristic of FD and helps differentiate it from other diseases of a similar phenotype.
- CMR is useful for distinguishing ventricular hypertrophy in FD from that of other cardiomyopathies due to the symmetrical concentric thickening pattern, fibrosis in inferolateral basal segments, and typically reduced native T1 values in the septum.
- Through CMR, the effects of enzyme replacement therapy or molecular chaperone therapy for FD can be assessed, including monitoring for hypertrophy regression and LE stabilization.

### 5.3. Glycogen Storage Diseases

Glycogen storage diseases (GSD) are a heterogeneous group of genetic metabolic diseases caused by mutations in the genes that encode enzymes involved in glucose metabolism, leading to intracellular glycogen accumulation in various organs. There are more than 12 types of GSD, and here we highlight those that affect the heart and skeletal muscle: Pompe disease (GSD type IIa) (autosomal recessive with pathogenic variants in the gene encoding α-glucosidase acid),^365^ Danon disease (GSD type IIb) (X-linked inheritance and mutations in the *LAMP2* gene, which encodes lysosome-associated membrane protein-2),^366^ and *PRKAG2* syndrome (autosomal dominant with mutations in the *PRKAG2* adenosine monophosphate-activated protein kinase gamma-2 regulatory subunit).^367^

GSD present different cardiac phenotypes, ranging from asymptomatic conditions to sudden cardiac death. Despite their broad range of clinical presentations, these diseases predominantly manifest in children (as early as the first year of life in Pompe disease) or young adults and have early increased LV wall thickness in common.

Echocardiography, the first-line imaging method, can reveal a hypertrophic, restrictive, or dilated phenotype.^366,368,369^ Hypertrophy is often severe (> 30 mm), biventricular, and rapidly progressive, especially in male Danon disease patients^366^ (Figure 24). Female Danon disease patients present with asymmetrical LVH, RVH, or, in 30% to 50% of cases, dilated or non-dilated hypokinetic phenotypes with slower progression. Dynamic obstruction of the LV outflow tract may also occur in GSD, especially Danon disease (up to 25% of the cases),^366^ and should be actively investigated in patients with dyspnea.

**Figure 24:** Transthoracic echocardiogram images of a patient with PRKAG2 syndrome. Significant concentric left ventricular hypertrophy is observed in the parasternal longitudinal view (A) and the apical longitudinal view (B), in addition to significant right ventricular hypertrophy in the apical view (right chambers) (C). Polar map of a patient with moderate left ventricular systolic function impairment demonstrating reduced global longitudinal strain (-5.9%) and a striped appearance.

Despite the crucial role of echocardiography, the clinical context is fundamental for the differential diagnosis with other hypertrophic phenocopies, especially sarcomeric hypertrophic cardiomyopathy, helping to guide the indication for genetic testing and/or biopsy.^368^ Electrocardiographic changes, such as a short PR interval, ventricular pre-excitation (Wolff-Parkinson-White syndrome), atrial fibrillation, intraventricular conduction delays, and sinoatrial or atrioventricular blocks, which require early pacemaker implantation, are considered warning signs of GSD.^366^ Unlike infiltrative cardiomyopathies, QRS voltage is normal or increased, which aids in the differential diagnosis with amyloidosis. Multisystem involvement (mainly myopathies and neurocognitive changes), LV diastolic dysfunction, and cardiopathy are the classic triad of Danon disease, which occurs more rarely in other GSD.^366^ The ventricular hypertrophy pattern can be identified by CMR, as discussed below.

Although advanced echocardiographic techniques, including speckle tracking and 3D myocardial strain imaging, are recommended in other hypertrophic or restrictive phenotypes,^5,105^ they lack robust validation in GSD. Reduced GLS with an apical sparing pattern (similar to that of CA) has been described in small retrospective case series on Danon disease, in which it was associated with worse outcomes.^370,371^

In 18 of 30 patients with *PRKAG2* syndrome, Pena et al. observed a peculiar regional striped pattern (varying from light to dark pink) in the polar GLS map (Figure 24).^372^ In the same series, these authors found that RV function, assessed by GLS and 3D echocardiography, was below normal in more than half of the patients, showing a significant positive correlation between the RV free wall longitudinal strain and RVEF. These parameters have greater sensitivity in detecting myocardial dysfunction than traditional echocardiographic indices, such as TAPSE.^373^ In another study by the same group that included 7 children of parents with *PRKAG2* syndrome, the echocardiographic findings indicated a trend towards cardiac hypertrophy.^374^

Table 21 summarizes the main echocardiographic characteristics of the GSD described above.

**Table 21:** Main echocardiographic characteristics of glycogen storage diseases

**Danon disease**	Rapidly progressive biventricular hypertrophy, with a severe phenotype in men or a variable phenotype in women, ranging from hypertrophy to dilation and systolic dysfunction. LV outflow tract obstruction occurs in 25% of patients and should be actively investigated in patients with dyspnea. Strain may present with an apical sparing polar map pattern, similar to amyloidosis.
**Pompe disease**	Concentric hypertrophy will rapidly progress to severe diffuse hypokinesia if left untreated. LV outflow tract obstruction may occur.
***PRKAG2*** **syndrome**	LV hypertrophy occurs in varying degrees, generally with a concentric pattern; RV hypertrophy is not uncommon; LV outflow tract obstruction is infrequent. In polar maps, strain may appear with a striped pattern.

LV: left ventricle; right ventricle.

The prognosis for hypertrophic phenocopies associated with GSD is generally worse than that of hypertrophic cardiomyopathy caused by variants of the sarcomeric protein gene. Of these, specific enzyme replacement therapy exists only for Pompe disease.^365^ However, early diagnosis facilitates therapy for HF and arrhythmias. Nevertheless, the benefits of enzyme replacement therapy in Pompe disease remain uncertain. Considering the severity and rapid progression of GSD, as well as its therapeutic implications, it is reasonable to recommend, even without robust evidence, echocardiography for disease screening every 1-2 years in symptomatic or asymptomatic children and adolescents with pathogenic variants and/or children of family members with early onset of GSD signs and symptoms. In individuals without the above described conditions, echocardiography is recommended every 3 to 5 years.^366^

CMR allows the detection of characteristic myocardial changes that can distinguish GSD from other forms of hypertrophic cardiomyopathy, enabling early diagnosis and monitoring of disease progression.^374^ This is particularly valuable considering the rapid progression of some of these diseases (such as Danon disease and infantile Pompe disease) and the need for early intervention. LE patterns, native T1 mapping, and ECV calculation aid in differential diagnosis with other hypertrophic phenotypes, as well as in determining disease stage (Table 22). In Danon disease, the extent of LE correlates with the degree of cardiac dysfunction and has important prognostic value, being a risk marker for malignant ventricular arrhythmias and sudden cardiac death. LE frequently presents a transmural or subepicardial distribution and involves multiple ventricular segments. CMR also allows for more accurate quantification of ventricular mass and wall thickness, as well as the detection of intracavitary thrombi secondary to ventricular dysfunction, overcoming some limitations of echocardiography, particularly the difficulty of visualizing cardiac segments, such as the apical region, the lateral wall, and the RV. Studies have shown that hypertrophy progresses more rapidly in LV diastolic dysfunction than in other cardiomyopathies, and CMR provides a sensitive method for monitoring it. The main CMR findings in patients with GSD are shown in Table 23.

**Table 22:** Main systemic changes in glycogen storage diseases observed in CMR

	Morphology	Late enhancement	T1 mapping	T2 or T2*	ECV
**Normal**	Normal	Absent	< 1050 ms	> 40 ms	< 40%
**Danon disease**	Marked symmetric LVH associated with LV dysfunction	Diffuse transmural or subepicardial	Increased or normal	N/A	Increased or normal
**Pompe disease**	Marked LVH at early presentation	Rare, inferolateral mid-wall	Increased or normal	N/A	Increased or normal
***PRKAG2* syndrome**	Diffuse or septal LVH	Rare in early stages	Reduced in early stages	N/A	N/A

LVH: left ventricular hypertrophy; N/A: not applicable; T1: longitudinal relaxation recovery; T2: transverse relaxation decay; LV: left ventricle; ECV: extracellular volume.

**Table 23:** Recommendations on multimodal imaging in patients with suspected or confirmed GSD

Recommendation	Strength of recommendation	Certainty of evidence
TTE should be used as a first-line imaging method when investigating suspected or known GSD.^105^	Weak	Moderate
In patients with GSD and an insufficient acoustic window, consider using ultrasound contrast agents for better resolution of endocardial borders and to increase ventricular opacification, facilitating the detection of hypertrophy or thrombi and increasing the accuracy of LVEF measurement.^105^	Weak	Low
In appropriate acoustic windows, LV global longitudinal strain can be used as an additional tool for detecting LV and RV subclinical systolic dysfunction in patients with GSD; the polar map pattern can aid in differential diagnosis with phenocopies.^370–372^	Weak	Low
Provided an adequate acoustic window, TTE with 3D imaging can be used to more accurately assess LVEF in patients with GSD.^105^	Weak	Low
For acute neurological events in patients with GSD, TEE should be recommended to detect intracardiac thrombi in the context of LV dysfunction or arrhythmias.^105^	Weak	Moderate
TTE with provocation maneuvers (Valsalva, orthostasis) is recommended for dyspneic patients with LV hypertrophy to investigate dynamic obstruction of the LV outflow tract, provided the resting LV outflow tract gradient is not ≥ 50 mm Hg.^366,368^	Weak	Moderate
Stress echocardiography is recommended in dyspneic patients with LV hypertrophy to investigate dynamic obstruction of the LV outflow tract, provided they do not present with a LV outflow tract gradient ≥ 50 mm Hg at rest or during a provocation maneuver.^366,368^	Weak	Moderate
When first-degree relatives are diagnosed and genetic testing is not possible, TTE should be used for family screening.^366^	Weak	Moderate
CMR should be considered when investigating GSD because it allows for better tissue characterization and phenotypic differentiation, in addition to providing prognostic information on treatment response.^365,374^	Weak	Moderate

ARU: ultrasound enhancing agents; DDG: glycogen storage disease; FEVE: left ventricular ejection fraction; LV: left ventricle; LVOT: left ventricular outflow tract; mm Hg: millimeters of mercury; RV: right ventricle; TEE: transesophageal echocardiography; TTE: transthoracic echocardiography; 3D: three-dimensional.

In the context of family counseling and carrier screening, CMR is a sensitive tool for detecting cardiac involvement in at-risk relatives, even in the absence of clinical symptoms. Considering the X-linked inheritance pattern, early identification of cardiac abnormalities in heterozygous women is particularly important, as they may develop cardiac manifestations later than men, but with equal severity.

Table 23 presents the main recommendations for multimodal cardiac imaging assessment in GSD according to the GRADE system.

The expert panel of this position statement recommends transthoracic echocardiography for clinical follow-up of patients diagnosed with GSD and for screening of cardiac involvement every 1 to 2 years in symptomatic or asymptomatic children and adolescents with pathogenic variants and/or offspring of family members with early onset of signs and symptoms of GSDs. Cardiac magnetic resonance may be performed every 1 or 2 years in symptomatic patients, in the presence of echocardiographic abnormalities, and in those with risk factors such as arrhythmias, HF, and clinical complications.^184,375^

**Key messages**

- GSD typically manifest in early life as LVH or biventricular hypertrophy in echocardiography.
- For echocardiograms showing LVH or biventricular hypertrophy, the clinical context is crucial for differential diagnosis, especially age of onset, mode of inheritance, association with ventricular pre-excitation, and multisystem involvement.
- Advanced echocardiography techniques, such as GLS and 3D, are promising for early detection of LV or biventricular dysfunction in patients with suspected or known GSD. The strain pattern in polar mapping can aid in the differential diagnosis with hypertrophic phenocopies
- CMR helps distinguish GSD from other cardiomyopathies through LVH distribution, native T1 and ECV values associated with dilation, and early loss of ventricular systolic function.

## 6. Applications of Multimodal Cardiac Imaging in Neuromuscular Diseases

Neuromuscular diseases are a heterogeneous group of rare degenerative conditions that affect motor, respiratory, and even swallowing function. The main conditions, Duchenne muscular dystrophy (DMD), Becker muscular dystrophy (BMD), and Friedreich ataxia (FA), are of genetic origin. DMD and BMD are X-linked genetic conditions, characterized by differing degrees of changes in dystrophin. Impairment may include cardiac function, which is a significant cause of mortality in these patients. There is no clear temporal correlation between skeletal muscle and myocardial involvement in any of these conditions, which represents a major challenge for cardiac dysfunction screening. Myocardial function assessment through noninvasive imaging methods, including their capabilities and recommendations, will be discussed below.

### 6.1. Duchenne Muscular Dystrophy

DMD is the most severe dystrophinopathy, leading to progressive motor function impairment, generally resulting in wheelchair dependence before 12 years of age.^376^ Although common, cardiomyopathy usually develops later in the course of the disease, with a dilated pattern that evolves to reduced LVEF around 14 years of age.^377^ About 33% of patients will develop dilated cardiomyopathy by 14 years of age, 50% between 14 and 18 years of age, and likely 100% by 18 years of age.^378^ Although there is still a scarcity of clinical studies with robust evidence, early diagnosis of cardiac involvement may allow for early cardioprotective therapy (with angiotensin-converting enzyme inhibitors or beta-blockers).^378^ A retrospective study found that, in < 4 years of follow-up, administering cardioprotective agents led to stabilized or even improved myocardial function in a significant percentage of the 69 patients.^379^

Due to its low cost and ease of access, TTE is the preferred method for evaluating and monitoring patients with DMD. Assessment is recommended at the time of diagnosis or beginning at 6 years of age, with evaluations every 2 years until 10 years of age, at which point annual evaluation is recommended.^380,381^ Myocardial involvement may begin regionally, primarily in the LV inferolateral wall (Figure 25). Advanced echocardiographic techniques for assessing ventricular function, such as multi-axis speckle-tracking strain analysis, including GLS and global circumferential strain parameters, has proven sensitive for early diagnosis of myocardial involvement.^382,383^ Initial studies point to the efficacy of regional strain as a means of identifying impaired myocardial strain in the LV inferolateral region.^384^

**Figure 25:** Bulls-eye image and curves for each myocardial segment in apical projections of longitudinal myocardial strain measurement in a patient with Duchenne muscular dystrophy, showing reduced strain in the posterolateral segments despite preserved left ventricular segmental contractility.

Studies comparing CMR with TTE in patients with DMD have shown that 20% to 40% of patients are underdiagnosed by TTE when using LVEF criteria.^385,386^ Reports of insufficient acoustic windows are also frequent in this patient population.^387^ In a recent cohort of 38 patients with DMD, Prakash et al. found that 52.6% had LVEF < 55% according to CMR and only 12% had abnormal GLS.^388^ A 2018 position statement recommended using TTE until around 6 to 7 years of age in patients with DMD and, as soon as possible, CMR for early detection of heart disease.^389^ It is important to consider that CMR is a more expensive and less readily available method, often requiring sedation in children or those with positioning difficulties, which can be common in this population.

### 6.2. Becker Muscular Dystrophy

Unlike DMD, which is the more severe form, BMD has slower progression and generally milder symptoms.^377^ Histopathological studies have shown that cardiac damage can precede clinical symptoms, beginning as early as adolescence, even in asymptomatic individuals or those with little motor impairment.^390^ As in DMD, myocardial involvement generally includes the inferolateral LV wall (Figures 26 and 27). Cardiological follow-up should begin during childhood or adolescence, with periodic evaluations even in asymptomatic individuals (an annual frequency is suggested). Detecting early functional changes that indicate a need for therapeutic intervention is still a clinical challenge, although echocardiography is playing an increasingly important role.

**Figure 26:** Echocardiographic images of a patient diagnosed with Becker muscular dystrophy, showing slight left ventricle dilation in the parasternal longitudinal view and little variation between diastolic (A) and systolic (B) diameters, with moderate systolic dysfunction. During systole, there is no thickening of the inferolateral wall (B). Image provided by Dr. Viviane Hotta.

**Figure 27:** Cardiac magnetic resonance images of a patient with Becker muscular dystrophy showing left ventricular dilation with significant left ventricular systolic dysfunction. Thinning of the basal and middle segments of the inferior and inferolateral walls is observed (A to F). Extensive heterogeneous late enhancement is observed in the basal and middle segments of the anterior lateral and mediobasal inferolateral walls (C, E, and F), with an appearance usually found in muscular dystrophy.

Although cardiac function analysis should be based on LVEF quantification, there is also evidence that advanced techniques, such as speckle-tracking myocardial strain assessment, allow for early diagnosis of global or regional myocardial dysfunction. An observational study^391^ found that 77.5% of patients with BMD presented with abnormal GLS (≥ −18%), while only 60% had reduced LVEF. Notably, GLS was altered in 17.5%, despite normal LVEF. After 2 years of follow-up, GLS had deteriorated significantly, while LVEF remained stable, demonstrating the role of strain analysis as an early and sensitive marker of disease progression. GLS was also significantly correlated with troponin I and NT-proBNP levels, but not with muscle or respiratory function. Tissue Doppler and LA function assessment, although less explored in BMD, can provide additional information on early diastolic dysfunction, especially when associated with atrial strain analysis. Early recognition of these changes is essential for disease-modifying therapies, such as cardioprotective agents, which can affect disease progression, morbidity, and mortality.

CMR is the diagnostic tool of choice for evaluating cardiac abnormalities like myocardial edema and fibrosis. Different techniques, such as T1 and T2 sequencing, LE, and multiparametric mapping are fundamental for assessing these patients, as is contractile function assessment and LVEF and RVEF calculation. Recent studies have found that LE increases with age (from 17% at < 10 years of age to 59% in those > 15 years of age with reduced LVEF).^392^ There is a linear correlation between the presence and number of segments affected by fibrosis and LVEF decrease. Typically, the fibrosis pattern in DMD and BMD affects the subepicardium or mesocardium, progressing with disease progression to transmural fibrosis, most frequently in the inferolateral LV wall.^393^ Recent studies have suggested that adipose tissue infiltration into skeletal muscle plays an important role in myocardial disease progression, and myocardial LE may result from the combination of fibrosis and adipose tissue.^394^ Thus, CMR plays a fundamental role not only in assessing the presence of fibrosis and ventricular function, but also in evaluating progression at a cellular level through the transformation of adipose tissue in heart muscle.

### 6.3. Friedreich Ataxia

Friedreich Ataxia (FA), a neurodegenerative disease with an autosomal recessive inheritance pattern, is caused by repeat expansion in the *FXN* gene, resulting in frataxin protein deficiency. Clinically, it is characterized by progressive ataxia, cerebellar dysfunction, scoliosis, DM and, often, cardiomyopathy, which is one of the most severe and lethal manifestations of the disease.^376,395^

FA-associated cardiomyopathy is characterized by LVH, primarily septal, which can progress to fibrosis, ventricular dysfunction, arrhythmias, and HF, the latter being the leading cause of death among patients. Although the relationship between atrial fibrillation-associated cardiomyopathy and neurological degeneration is unclear, regular assessment of cardiac involvement is essential in clinical practice. FA-associated cardiomyopathy can be classified into 4 categories based on interventricular septum thickness in diastole and LVEF, as shown in Table 24. However, with disease progression, LV mass may reduce due to LVH regression and increased myocardial fibrosis. These parameters can be assessed by serial 2D echocardiography. LVEF estimation is crucial, since decreased values are associated with a higher risk of mortality.^396^

**Table 24:** Classification of Friedreich ataxia-associated cardiomyopathy

Classification	Diagnostic criteria	Characteristics
Mild CMP-FA	Increased IVSd* (≤18% of predicted) and LVEF ≥ 50%	Mild LVH and preserved systolic function
Moderate CMP-FA	Increased IVSd* (≥ 18% of predicted) and LVEF ≥ 50%	Moderate LVH, possible onset of remodeling.
Severe CMP-FA	LVEF ≤ 50% (independent of IVSd)	Ventricular dilation, decreased myocardial GLS, possible signs of fibrosis.

* AInterventricular septum thickness in diastole considered normal based on Henry et al.'s normogram^.397^ CMP-FA: Friedreich ataxia-associated cardiomyopathy; GLS: global longitudinal strain; IVSd: interventricular septum thickness in diastole; LVEF: left ventricular ejection fraction; LVH: left ventricular hypertrophy.

Advanced echocardiographic techniques like LV GLS analysis can indicate early changes in relation to LVEF and are useful for detecting subclinical dysfunction. The most common alteration is diastolic dysfunction (84%), which is mainly pseudonormalized and can be determined through the transmitral E/A ratio, mitral flow deceleration rate, and the E/e’ ratio.^398^

Given the scarcity of controlled clinical trials and systematic reviews focused on diagnosing FA-associated cardiomyopathy, we suggest a systematic evaluation of the TTE parameters listed in Table 25.

**Table 25:** Key echocardiographic parameters for cardiac evaluation of Friedreich ataxia

Echocardiographic parameter	Associated cardiac abnormality
IVSd	Concentric hypertrophy
Posterior wall thickness	Hypertrophy and geometry
Relative wall thickness	Concentric remodeling
LV mass index	Ventricular hypertrophy (mortality predictor)
LVEF	Global systolic function (survival predictor)
Global longitudinal strain	Early subclinical myocardial dysfunction
Mitral E/A ratio	Diastolic function (pseudonormalized)
E/e' ratio	Ventricular filling pressure
Isovolumetric relaxation time	Early diastolic dysfunction

IVSd: interventricular septum thickness in diastole; LV: left ventricular; LVEF: LV ejection fraction.

CMR, a valuable tool for characterizing fibrosis and myocardial remodeling in patients with FA, even in the absence of clinical HF, can detect LVH and myocardial fibrosis.^399^ CMR can also reveal early myocardial remodeling, characterized by hypertrophy and increased ECV, even in patients with preserved LVEF.^399^ The technique also allows detection of abnormalities in myocardial perfusion reserve, which are indicative of myocardial ischemia in the absence of epicardial CAD.^400^

Thus, in light of evidence in the literature, for patients with suspected cardiomyopathy related to neuromuscular diseases (mainly DMD, BMD, and FA), we recommend a cardiomyopathy screening strategy involving TTE, taking accessibility and cost into consideration. According to GRADE criteria,^401^ this recommendation is strong, based on moderate-certainty evidence (mainly for DMD) regarding early diagnosis, which could beneficially affect the progression of myocardial dysfunction (although the level of certainty about its impact on survival is still low) (Table 26).

**Table 26:** Recommendations on echocardiography in patients with neuromuscular degenerative diseases

Recommendation	Strength of recommendation	Certainty of evidence
Transthoracic echocardiography should be performed as a first-line assessment in patients with neuromuscular diseases to screen for ventricular dysfunction.	Strong	Moderate

In patients with DNM, evaluation is recommended at the time of diagnosis or from 6 years of age, with assessments every 2 years until 10 years of age, when annual evaluation is recommended. In patients with evidence of reduced LVEF or in the presence of abnormalities detected by GLS, annual evaluation is recommended.^380,381^

## 7. Perspectives

In cardiology, as in all medical specialties, multimodal integration of imaging techniques is a clear necessity for patients. Each cardiac imaging method has its advantages and disadvantages, and no single method can provide all of the required information about the complex functioning of the cardiovascular system.

Given the particularities and the interface between inflammatory factors and traditional cardiovascular risk factors in each specific disease, recommendations for the indication and frequency of each cardiac imaging examination should be individualized for each disease and patient. The purpose of these examinations is to reduce the risk of cardiometabolic and inflammatory complications related to traditional risk factors and to facilitate early diagnosis of abnormalities, including HF (with preserved or reduced EF), as well as coronary, valvular, myocardial, pericardial, and aortic diseases associated with systemic diseases. Thus, given the complexity and cardiac complications of each disease, a structured multimodal approach for the diagnosis, prognosis, and monitoring of these abnormalities is critically necessary.^4,9–11,402,403^

Despite the well-established value of new technologies for the early detection of subclinical changes in cardiac function, echocardiographic myocardial strain analysis is not yet routinely used. Because this method depends on specific equipment, examiner training, and requires more time to perform, it demands adequate remuneration, thus limiting its use in clinical practice. Nevertheless, in clinical practice, myocardial strain assessment should be systematically integrated into conventional echocardiographic analysis.^5,6^ New cardiac CT-associated techniques for investigating myocardial ischemia, such as fractional flow reserve and perfusion assessment, can improve diagnostic accuracy, but they remain underutilized.^9,10,403^ However, 4D CMR allows for 3D visualization of vascular flow over time and quantitative measurement of transvalvular and intracavitary flow.^11,403^

Additionally, the integration of precision medicine and artificial intelligence in cardiovascular practice has transformed the assessment and management of systemic diseases with cardiac involvement.^404,405^ Precision medicine is an effort to tailor diagnosis and treatment based on the specific biological, clinical, and imaging characteristics of each patient. Artificial intelligence enhances this approach by processing large volumes of data, identifying subtle patterns and predicting disease trajectories with greater accuracy. When applied to multimodal cardiac imaging, artificial intelligence can integrate data on functional, structural, and tissue characterization, promoting more precise phenotypic stratification and aiding in the early detection of myocardial involvement.^406,407^

In systemic diseases, early and accurate characterization of cardiac changes is crucial for risk stratification and treatment planning. In diseases such as amyloidosis or sarcoidosis, for example, combining CMR and PET or SPECT increases diagnostic sensitivity and can identify subclinical myocardial inflammation or infiltration. Similarly, in autoimmune rheumatic diseases, integrating echocardiography, nuclear medicine, and CMR can reveal early signs of ischemia, myocarditis, and fibrosis, which often go unnoticed in the early stages.

Thus, the convergence of precision medicine, artificial intelligence, and multimodal cardiac imaging fosters a deeper understanding of the cardiovascular impact of systemic diseases, enabling screening and early diagnosis of cardiovascular complications in patients with generalized systemic diseases, as well as personalized monitoring of cardiac function and associated changes during follow-up.

**Key messages**

- Impaired cardiac function, a significant cause of mortality, may occur in neuromuscular diseases such as DMD, BMD, and FA.
- DMD is the most severe form of dystrophinopathy. Although common, cardiomyopathy (with a dilated pattern) usually develops later, evolving to reduced LVEF at around 14 years of age.
- TTE is the preferred method for assessing and monitoring patients with neuromuscular diseases.
- In DMD and BMD, the pattern of myocardial involvement can begin regionally, primarily in the LV inferolateral wall.
- LV GLS has proven to be a sensitive parameter for early diagnosis of myocardial involvement in neuromuscular diseases.
- FA-associated cardiomyopathy is characterized by LVH, primarily septal, which can progress to fibrosis, ventricular dysfunction, arrhythmias, and HF.
- Patients with FA and advanced heart disease may experience LVH regression and increased myocardial fibrosis. LVEF analysis with TTE is crucial, since reduced values are associated with a higher mortality risk.

## 8. Conclusions

Due to the wide variety of cardiovascular manifestations in systemic diseases, routine requests for multimodal cardiac imaging in clinical follow-up must be standardized. However, no routine for requesting cardiac imaging examinations has been established. Advances in cardiac imaging techniques, the growing evidence of their usefulness in patients with systemic diseases, and multidisciplinary work among cardiologists, rheumatologists, pulmonologists, neurologists, etc. will contribute to the development of a standardized routine for multimodal imaging in these patients.

## Data Availability

The data underlying the text of the Position Statement are included in the manuscript.

## List of abbreviations and acronyms

18F-FDG: 18F-fluorodeoxyglucose
2D: Two-dimensional
3D: Three-dimensional
99mTc: 99m-technetium
AL: Light chain amyloidosis
AS: Ankylosing spondylitis
ATTR: Transthyretin amyloidosis
BMD: Becker muscular dystrophy
CA: Cardiac amyloidosis
CAD: Coronary artery disease
CMR: Cardiac magnetic resonance imaging
CS: Cardiac sarcoidosis
CT: Computed tomography
DM: Diabetes mellitus
DMD: Duchenne muscular dystrophy
ECV: Extracellular volume
EF: Ejection fraction
FA: Friedreich ataxia
FD: Fabry disease / Anderson-Fabry disease
GLS: Global longitudinal strain
GRADE: Grading of Recommendations, Assessment, Development and Evaluation
GSD: Glycogen storage diseases
HF: Heart failure
HFpEF: Heart failure with preserved ejection fraction
LA: Left atrium
LE: Late enhancement
LV: Left ventricle
LVEF: Left ventricular ejection fraction
LVH: Left ventricular hypertrophy
MPS: Myocardial perfusion scintigraphy
MRI: Magnetic resonance imaging
PAH: Pulmonary arterial hypertension
PET: Positron emission tomography
PH: Pulmonary hypertension
PYP: Pyrophosphate
RA: Rheumatoid arthritis
RV: Right ventricle
SLE: Systemic lupus erythematosus
SPECT: Single photon emission computed tomography
T2DM: *Type 2 diabetes mellitus*
TA: Takayasu's arteritis
TAPSE: Tricuspid annular plane systolic excursion
TTE: Transthoracic echocardiography
